# First-line zolbetuximab plus mFOLFOX6 and nivolumab in unresectable CLDN18.2-positive gastric or gastroesophageal junction adenocarcinoma: a phase 2 trial

**DOI:** 10.1038/s41591-026-04306-9

**Published:** 2026-03-16

**Authors:** Kohei Shitara, Hirokazu Shoji, Nicola Fazio, Sara Lonardi, Keun-Wook Lee, Li-Yuan Bai, Kensei Yamaguchi, Jean-Philippe Metges, Gianluca Masi, Denis Smith, Tae-Yong Kim, Maria Matsangou, Archita Shrivastava, Miaomai Zhou, Jason Hill, Abraham Guerrero, Xuewei Wang, Aziz Zaanan, Samuel J. Klempner

**Affiliations:** 1 Department of Gastrointestinal Oncology, National Cancer Center Hospital East, Kashiwa City, Japan; 2 Department of Gastrointestinal Medical Oncology, National Cancer Center Hospital, Tokyo, Japan; 3 Division of Gastrointestinal Medical Oncology and Neuroendocrine Tumors, European Institute of Oncology, IEO, IRCCS, Milan, Italy; 4 Medical Oncology 1, Veneto Institute of Oncology IOV-IRCCS, Padua, Italy; 5 Division of Hematology and Medical Oncology, Department of Internal Medicine, Seoul National University Bundang Hospital, Seoul National University College of Medicine, Seongnam, South Korea; 6 Division of Hematology and Oncology, Department of Internal Medicine, China Medical University Hospital, and College of Medicine, China Medical University, Taichung, Taiwan; 7 Department of Gastroenterological Chemotherapy, The Cancer Institute Hospital of JFCR, Tokyo, Japan; 8 Institut de Cancerologie et d’Hematologie, ARPEGO Network CHU Morvan, Brest, France; 9 Department of Translational Research and New Technologies in Medicine and Surgery, University of Pisa and Division of Medical Oncology, Azienda Ospedaliero-Universitaria Pisana, Pisa, Italy; 10 Department of Digestive Oncology, CHU Bordeaux, Pessac, France; 11 Department of Internal Medicine, Seoul National University Hospital, Seoul, South Korea; 12 Astellas Pharma Global Development, Inc., Northbrook, IL USA; 13 Department of Gastroenterology and Digestive Oncology, Institut du Cancer Paris Carpem, Assistance Publique des Hôpitaux de Paris, European Georges Pompidou Hospital, University of Paris Cité, Paris, France; 14 Department of Medicine, Division of Hematology-Oncology, Massachusetts General Hospital Center, Boston, MA USA

**Keywords:** Tumour biomarkers, Gastric cancer

## Abstract

There is an unmet need for effective and safe treatments for patients with metastatic gastric/gastroesophageal junction (mG/GEJ) adenocarcinoma. Targeting claudin 18 isoform 2 (CLDN18.2) and programmed death ligand 1 (PD-L1), represents a promising strategy. Zolbetuximab, a CLDN18.2-targeting antibody, plus chemotherapy improved survival outcomes in patients with CLDN18.2-positive, human epidermal growth factor receptor 2 (HER2)-negative mG/GEJ adenocarcinoma. Cohort 4 of the global, open-label, phase 2 ILUSTRO study examined first-line zolbetuximab plus mFOLFOX6 and nivolumab (a PD-L1 inhibitor). Here we report results from cohorts 4A (safety lead-in phase) and 4B (expansion phase). The primary endpoint of ILUSTRO was specific to cohort 1 and was previously published; the main efficacy endpoint of interest for cohort 4 was progression-free survival (PFS), as assessed by the investigators per Response Evaluation Criteria in Solid Tumors version 1.1. At data cutoff (2 September 2025) for this final analysis, 77 patients were enrolled in 4A + 4B (85.5% with CLDN18.2-high tumors). Cohort 4B median follow-up was 11.5 months, and median PFS (95% confidence interval (CI)) was 14.8 months (8.3–not estimable) overall (*n* = 71) and 18.0 months (11.1–not estimable) in patients with CLDN18.2-high tumors (*n* = 59). Objective response rate (measurable disease; 95% CI) was 62.1% (48.4–74.5) in 4B overall (*n* = 58) and 68.1% (52.9–80.9) in CLDN18.2-high (*n* = 47). In 4A + 4B, the most common treatment-emergent adverse events were nausea (80.5%) and decreased appetite (72.7%). Efficacy and safety data support randomized evaluation of zolbetuximab plus chemoimmunotherapy in patients with CLDN18.2-positive and PD-L1-positive mG/GEJ adenocarcinoma in the ongoing phase 3 LUCERNA study. ClinicalTrials.gov: NCT03505320.

## Abstract (web summary)

In cohort 4 of the ILUSTRO trial, combination of anti-CLDN18.2 zolbetuximab plus mFOLFOX6 and nivolumab in patients with CLDN18.2-positive, HER2-negative metastatic gastric or gastroesophageal junction adenocarcinoma led to encouraging clinical efficacy, supporting the testing of this combination in a phase 3 trial.

## Main

Gastric/gastroesophageal junction (G/GEJ) adenocarcinoma is a leading cause of cancer mortality^1^. In 2022, gastric cancer ranked fifth among cancers for worldwide incidence and mortality, with almost 1 million new cases and more than 650,000 deaths^1^. In modern phase 3 trials, many studies demonstrate median overall survival (OS) of approximately 14 months in patients with mG/GEJ adenocarcinoma^2–9^.

The standard of care for first-line therapy for mG/GEJ adenocarcinoma is platinum/fluoropyrimidine-based chemotherapy^10–14^. The inclusion of biomarker-directed treatments in combination with platinum/fluoropyrimidine-based chemotherapy improves outcomes^2,4,5,15,16^. Testing for biomarkers including PD-L1, HER2 and CLDN18.2 expression is endorsed by international guidelines and supported by phase 3 trial data^2,4,5,11,15–17^. The biomarker CLDN18.2 is a tight junction protein normally expressed in gastric mucosa cells; these lose epithelial polarity during malignant transformation, exposing CLDN18.2 epitopes to the cell surface and enabling therapeutic targeting via antibody binding for patients with mG/GEJ adenocarcinoma^18–22^. Although estimates for CLDN18.2 positivity vary depending on assessment criteria, geographical region, age and other parameters, approximately 35–45% of patients with gastric cancer are CLDN18.2 positive^18,23^.

Zolbetuximab is a first-in-class, chimeric, IgG1 monoclonal antibody that targets CLDN18.2 and mediates the death of CLDN18.2-positive G/GEJ adenocarcinoma cells via antibody-dependent cellular cytotoxicity and complement-dependent cytotoxicity^20,21^. Zolbetuximab plus chemotherapy has demonstrated clinically meaningful and statistically significant improvements in PFS and OS compared with chemotherapy alone in the phase 3 SPOTLIGHT and GLOW studies in patients with CLDN18.2-positive, HER2-negative mG/GEJ adenocarcinoma^6,8,16^. In the pooled analysis of these trials, median PFS was 9.2 months with zolbetuximab plus chemotherapy and 8.2 months with chemotherapy alone (hazard ratio = 0.71, 95% CI: 0.61–0.83), and median OS was 16.4 months versus 13.7 months (hazard ratio = 0.77, 95% CI: 0.67–0.89), respectively^16^.

Building on the clinical efficacy established separately for zolbetuximab plus chemotherapy and for chemotherapy plus immunotherapy, preclinical and translational observations suggest complementary biology between CLDN18.2 targeting and PD-1 pathway blockade. Zolbetuximab-mediated cytotoxicity can increase antigen release and dendritic cell priming, and co-targeting the PD-1 pathway potentially preserves and amplifies tumor-specific T cell response, thus leveraging innate and adaptive immune responses^18,24,25^. In a CLDN18.2-expressing syngeneic mouse model, triplet therapy with zolbetuximab, chemotherapy and PD-1 inhibition achieved greater tumor control than any of the three separate doublet therapies^25^. Exploratory analyses combining data from a phase 1 study (NCT03528629) and cohorts 1 and 2 from the phase 2 ILUSTRO study used limited on-treatment tissue samples from patients who had received zolbetuximab (alone or in combination with mFOLFOX6) and showed increased CD8^+^ T cells and CD163^+^ macrophages, potentially suggesting immune microenvironment remodeling; however, a correlation with clinical efficacy was not investigated^24^. Combined, these studies provide support for evaluating the triplet therapeutic regimen of zolbetuximab plus chemotherapy and PD-1-based immunotherapy.

The phase 2 multicohort ILUSTRO study (NCT03505320) investigated efficacy and safety of zolbetuximab as monotherapy or in combination with chemotherapy and/or immunotherapy (including blockade of PD-1) in patients with mG/GEJ. Results from cohort 1 (third-line or later zolbetuximab monotherapy), cohort 2 (first-line zolbetuximab plus mFOLFOX6) and cohort 3 (third-line or later zolbetuximab plus pembrolizumab) have been published^3^. Cohort 4 of ILUSTRO evaluated first-line zolbetuximab plus mFOLFOX6 and nivolumab in patients with HER2-negative, locally advanced unresectable or mG/GEJ adenocarcinoma whose tumors had either high or intermediate CLDN18.2 positivity.

## Results

### Patient characteristics

**Fig. 1:**
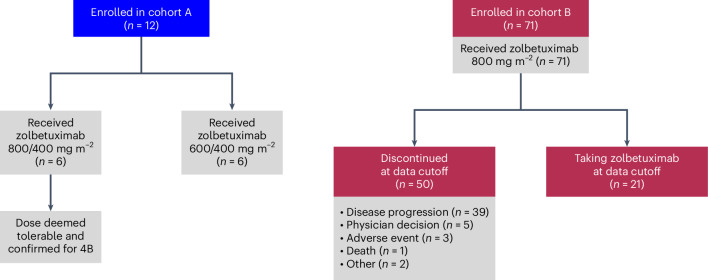
CONSORT diagram for cohort 4 of the ILUSTRO study. Patient disposition at the data cutoff date of September 2, 2025.

Here we report the results from the final analysis of ILUSTRO cohort 4, which comprised two subcohorts: 4A (safety lead-in phase) and 4B (expansion phase). Patients were screened and enrolled between 2 February and 5 September 2022 for cohort 4A and between 29 May 2023 and 9 October 2024 for cohort 4B. ILUSTRO is an ongoing study without database lock, but the data cutoff date for this analysis was 2 September 2025, when the prespecified number of PFS events defined in the statistical analysis protocol had been reached. As of the data cutoff date, 77 patients were enrolled in cohort 4 (cohort 4A, *n* = 6; cohort 4B, *n* = 71) who had received a loading dose of zolbetuximab 800 mg m^−^^2^ (Fig. 1). Six patients in cohort 4A who received a loading dose of zolbetuximab 600 mg m^−^^2^ were not included in the analyses. All analyses described herein were conducted in patients who received identical therapy (that is, a loading dose of zolbetuximab 800 mg m^−^^2^). Baseline characteristics and safety analyses were conducted in cohort 4A + 4B, and primary efficacy analyses were conducted in cohort 4B only; efficacy analyses for cohort 4A + 4B were exploratory and are presented as extended data items. The safety analysis set comprised all patients who had received at least one dose of zolbetuximab (cohort 4A, 6/6 who received zolbetuximab 800 mg m^−^^2^; cohort 4B, 71/71) and, per the study protocol, was used for PFS and OS analyses and for safety analyses. The full analysis set included 76 of 77 patients (98.7%; cohort 4A, 6/6 (100%); cohort 4B, 70/71 (98.6%)) who had received at least one dose of study drug and had at least one posttreatment disease assessment; this set was used for tumor response analyses. Ad hoc analyses for tumor responses were conducted including only patients with measurable disease (cohort 4B, *n* = 58; cohort 4A + 4B, *n* = 62).

In cohort 4A + 4B (*n* = 77), median age was 61.0 years (range, 37.0–86.0), and 62.3% of patients were male (Table 1). Of 76 enrolled patients with valid tumor CLDN18 immunohistochemistry (IHC) results, 65 (85.5%) had tumors with high CLDN18.2 expression (moderate-to-strong membranous CLDN18 staining in ≥75% of tumor cells), and 11 (14.5%) had tumors with intermediate CLDN18.2 expression (moderate-to-strong membranous CLDN18 staining in ≥50% and <75% of tumor cells). Sixty-three (81.8%) patients had measurable disease at baseline, and the stomach was the primary tumor site in most patients (86.5%). Among patients with available tumor PD-L1 status (*n* = 75), 34.7% had a PD-L1 combined positive score (CPS) < 1 and 65.3% of patients had a CPS ≥ 1. Lauren classification was diffuse in 53 patients (70.7%), intestinal in 10 patients (13.3%), mixed in three patients (4.0%) and other in nine patients (12.0%).

**Table 1:** Baseline demographic and clinical characteristics in the safety analysis set

Parameter, *n* (%)^a,b^	Cohort 4B (*n* = 71)	Cohort 4A + 4B (*n* = 77)
Age, median (range)	61.0 (37.0–86.0)	61.0 (37.0–86.0)
Sex		
Male	44 (62.0)	48 (62.3)
Female	27 (38.0)	29 (37.7)
Race^b^		
Asian	47 (77.0)	53 (79.1)
White	14 (23.0)	14 (20.9)
Previous treatment^b^		
Surgery alone	3 (4.7)	3 (4.3)
Surgery followed by chemotherapy	2 (3.1)	3 (4.3)
Chemotherapy followed by surgery	1 (1.6)	1 (1.4)
Primary site^b^		
Stomach	58 (85.3)	64 (86.5)
Gastroesophageal junction	10 (14.7)	10 (13.5)
Number of metastatic sites		
0–2	49 (69.0)	54 (70.1)
≥3	22 (31.0)	23 (29.9)
Lauren classification^b^		
Diffuse	49 (71.0)	53 (70.7)
Intestinal	9 (13.0)	10 (13.3)
Mixed	2 (2.9)	3 (4.0)
Other	9 (13.0)	9 (12.0)
ECOG PS		
0	46 (64.8)	51 (66.2)
1	25 (35.2)	26 (33.8)
Measurable disease		
Yes	59 (83.1)	63 (81.8)
No	12 (16.9)	14 (18.2)
CLDN18.2 expression^b^		
High^c^	59 (84.3)	65 (85.5)
Intermediate^d^	11 (15.7)	11 (14.5)
PD-L1 status^b^		
CPS ≥ 1	47 (68.1)	49 (65.3)
CPS < 1	22 (31.9)	26 (34.7)

^a^Unless otherwise specified.

^b^Percentages were calculated out of patients with non-missing data; percentages may not add up to 100% due to rounding.

^c^Defined as ≥75% of tumor cells demonstrating moderate-to-strong membranous CLDN18 staining as determined by central IHC.

^d^Defined as ≥50% to <75% of tumor cells demonstrating moderate-to-strong membranous CLDN18 staining as determined by central IHC.

ECOG PS, Eastern Cooperative Oncology Group performance status.

### Efficacy

**Fig. 2:**
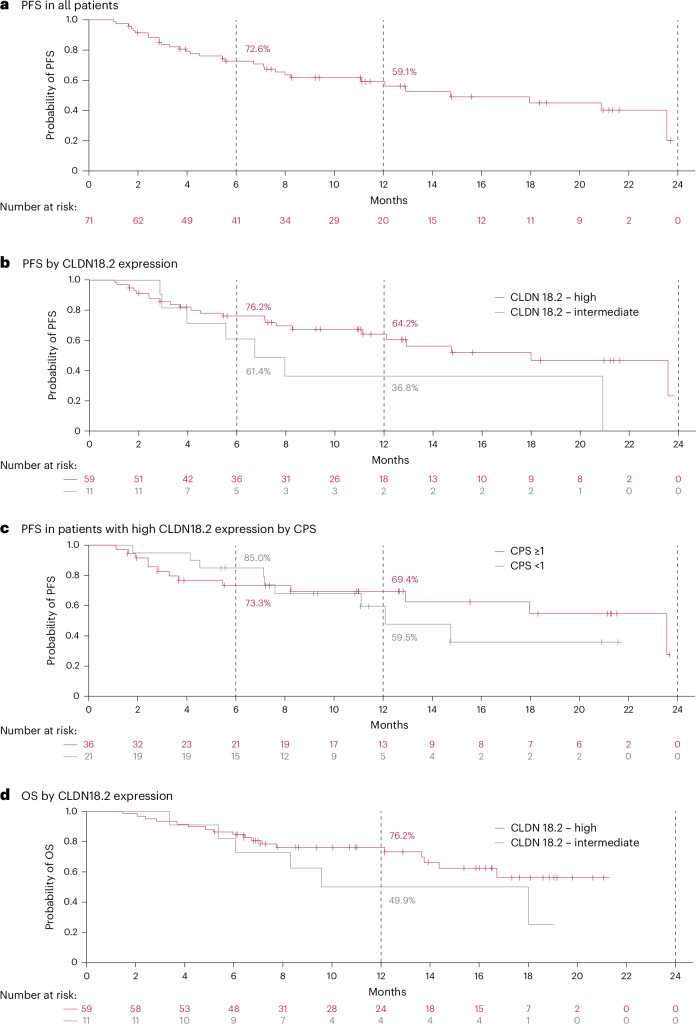
Survival outcomes in cohort 4B in the safety analysis set. **a**, Kaplan-Meier curves of PFS in all patients. **b**, Kaplan-Meier curves of PFS by CLDN18.2 expression. **c**, Kaplan-Meier curves of PFS in patients with high CLDN18.2 expression by PD-L1 CPS. **d**, Kaplan-Meier curves of OS by CLDN18.2 expression. PFS was assessed by investigators per RECIST v1.1. PFS and OS were estimated using the Kaplan−Meier method with corresponding 95% CIs. One patient was missing a value for CLDN18.2 expression and is, therefore, excluded from **b** and **d**. Two patients were missing PD-L1 CPS values and are, therefore, excluded from **c**.

**Extended Data Fig. 1:**
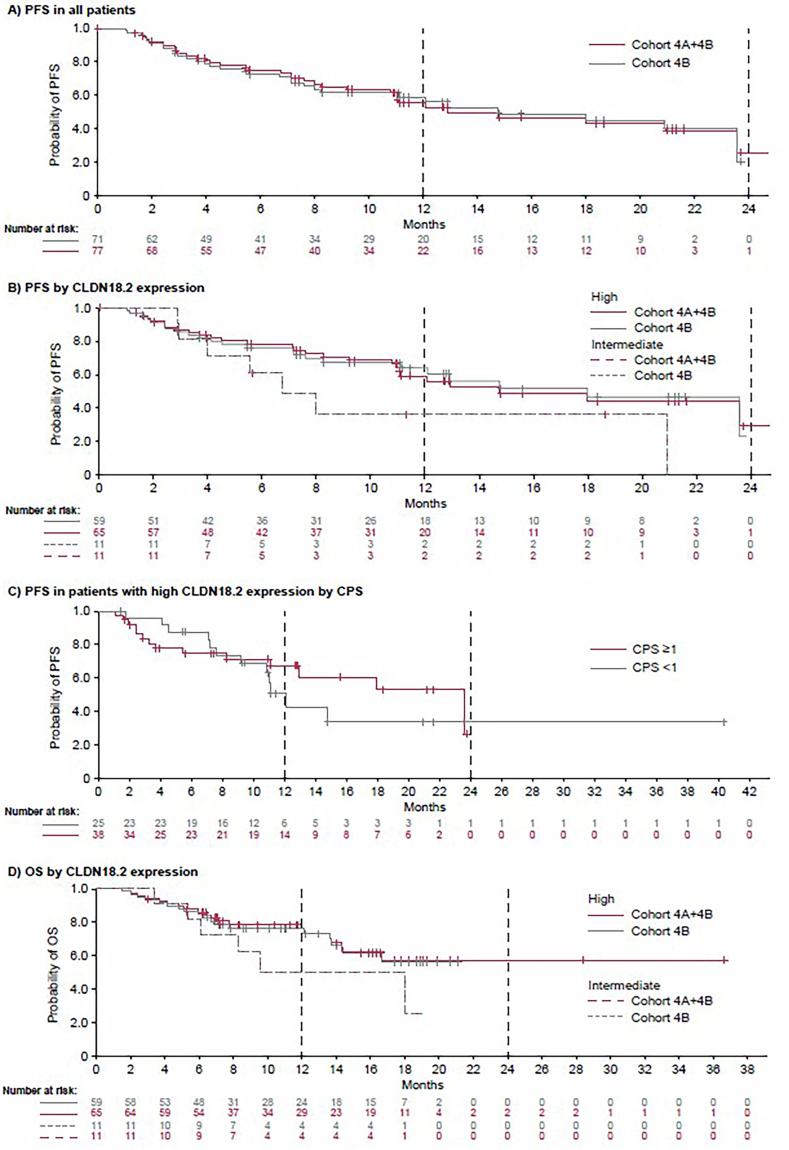
Survival outcomes in cohort 4B and exploratory analysis cohort 4A + 4B in the safety analysis set. **a**, Kaplan-Meier curves of PFS in all patients. **b**, Kaplan-Meier curves of PFS by CLDN18.2 expression. **c**, Kaplan-Meier curves of PFS in patients in cohort 4A + 4B with high CLDN18.2 expression by PD-L1 CPS. **d**, Kaplan-Meier curves of OS by CLDN18.2 expression. PFS was assessed by investigators per RECIST v1.1. PFS and OS were estimated using the Kaplan-Meier method with corresponding 95% CIs. One patient was missing a value for CLDN18.2 expression. Two patients were missing PD-L1 CPS values. Cohort 4 A had no patients with intermediate CLDN18.2 expression, and therefore cohorts 4 A + B and 4B are identical. CLDN18.2, claudin 18 isoform 2; CPS, combined positive score; OS, overall survival; PFS, progression-free survival; RECIST v1.1, Response Evaluation Criteria in Solid Tumors version 1.1.

In the final statistical analysis plan, investigator-assessed PFS was designated as the main efficacy endpoint based on statistical assumptions for cohort 4B, and this determined the sample size and timing of the analysis (see the study protocol and statistical analysis plan in the Supplementary Information). In cohort 4B, median follow-up time for PFS was 11.5 months, and median PFS was 14.8 months (95% CI: 8.3–not estimable) (Fig. 2a and Extended Data Table 1). At 6 months and 12 months, PFS rates were 72.6% and 59.1%, respectively. Post hoc analyses according to tumor CLDN18.2 expression showed that median PFS in patients whose tumors had high CLDN18.2 expression (*n* = 59) was 18.0 months (95% CI: 11.1–not estimable) (Fig. 2b and Extended Data Table 1), and the lower bound of the 95% CI exceeded the prespecified threshold of 8.5 months. Kaplan−Meier landmark estimates at 6 months and 12 months showed longer PFS in patients whose tumors had high versus intermediate CLDN18.2 expression, although the number of patients categorized as intermediate was small (Extended Data Table 1). In cohort 4B, 36 of 57 patients (63.2%) with high CLDN18.2 expression had PD-L1 CPS ≥ 1, with a median PFS of 23.6 months; those with high CLDN18.2 expression and PD-L1 CPS < 1 (*n* = 21) had a median PFS of 12.1 months (Fig. 2c and Extended Data Table 2). OS data were immature at the time of analysis; 25 of 71 patients (35.2%) in the cohort 4B safety analysis set had died by the data cutoff. With a median follow-up time of 15.4 months, median OS was 18.0 months (95% CI: 13.6–not estimable) in all patients in cohort 4B and not estimable (95% CI: 13.7–not estimable) among those whose tumors had high CLDN18.2 expression (Fig. 2d and Extended Data Table 1). Exploratory efficacy analyses of cohort 4A + 4B (*n* = 77) are included in Extended Data Fig. 1 and Extended Data Table 1.

**Fig. 3:**
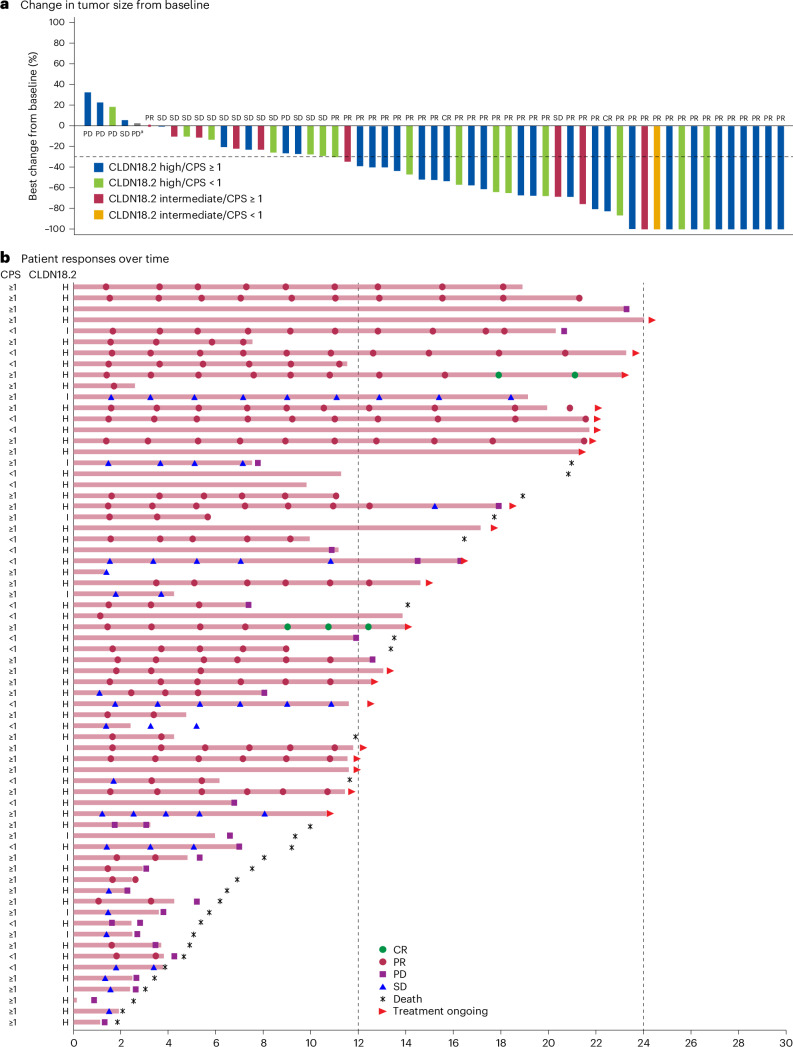
Tumor responses in cohort 4B in the full analysis set. **a**, Waterfall plot of change in tumor size from baseline. **b**, Swimmer plot of patient responses over time. Patients included in the waterfall plot (**a**) were those with baseline and post-baseline measurements; patients classified as non-CR/non-PD are not included (*n* = 57). One patient had no evaluable CLDN18.2 status and is not shown in either plot. Two patients were missing PD-L1 CPS values and are not shown in the swimmer plot (**b**). ^a^This patient had no evaluable CPS status. CR, complete response; H, CLDN18.2 high; I, CLDN18.2 intermediate; PD, progressive disease; PR, partial response; SD, stable disease.

**Extended Data Fig. 2:**
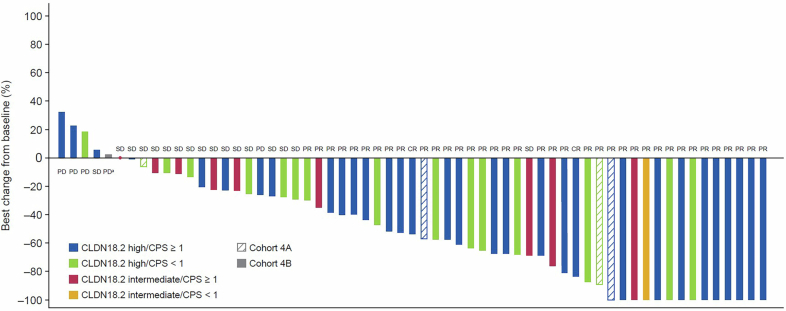
Change in tumor size from baseline in cohort 4A + 4B in the full analysis set. Patients included were those with baseline and post-baseline measurements (*n* = 61); patients classified as non-CR/non-PD are not included. One patient had no evaluable CLDN18.2 status. ^a^This patient had no evaluable CPS status. CLDN18.2, claudin 18 isoform 2; CR, complete response; PD, progressive disease; PR, partial response; SD, stable disease.

**Extended Data Fig. 3:**
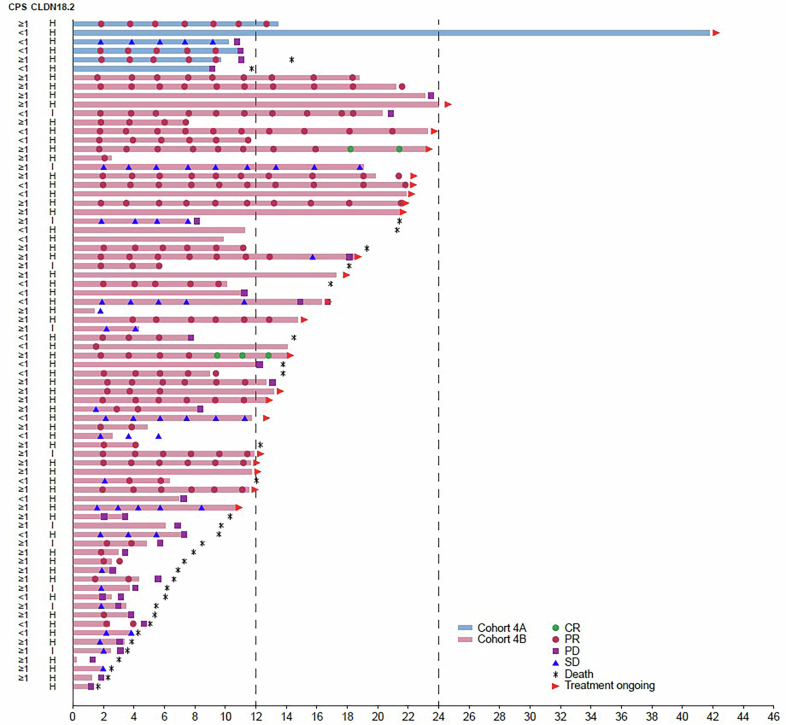
Patient responses over time in cohort 4A + 4B in the full analysis set. One patient had no evaluable CLDN18.2 status and is therefore excluded from the plot (total *n* = 75). The last patient is missing a CPS value. CLDN18.2, claudin 18 isoform 2; CPS, combined positive score; CR, complete response; H, CLDN18.2-high; I, CLDN18.2 intermediate; PD, progressive disease; PR, partial response; SD, stable disease.

Change in tumor size from baseline in patients in the full analysis set in cohort 4B, not including patients classified as non-complete response/non-progressive disease, is shown in Fig. 3a. In ad hoc analyses of patients in cohort 4B with measurable disease at baseline (*n* = 58), objective response rate (ORR) was 62.1% (95% CI: 48.4–74.5), with two complete responses (3.4%), 34 partial responses (58.6%) and a disease control rate (DCR) of 89.7% (95% CI: 78.8–96.1) (Table 2). Among 47 patients with measurable disease whose tumors had high CLDN18.2 expression, ORR was 68.1% (95% CI: 52.9–80.9); among 10 patients with measurable disease whose tumors had intermediate CLDN18.2 expression, ORR was 40.0% (95% CI: 12.2–73.8). Of the patients with measurable disease whose tumors had high CLDN18.2 expression, ORR was 74.2% (95% CI: 55.4–88.1) for patients with PD-L1 CPS ≥ 1 (*n* = 31) and 60.0% (95% CI: 32.3–83.7) for patients with PD-L1 CPS < 1 (*n* = 15) (Extended Data Table 3). Responses were generally durable (Fig. 3b); median duration of response (DOR) for patients in cohort 4B with measurable disease was 19.1 months (95% CI: 10.8–not estimable) and in patients in cohort 4B with high CLDN18.2 expression was not estimable (10.8–not estimable). Tumor response results from the exploratory analyses of patients in cohort 4A + 4B with measurable disease and in the full analysis set are included in Extended Data Figs. 2 and 3 and in Extended Data Tables 4 and 5, respectively. ORR results confirmed by a second scan at least 4 weeks after the first documented response are shown in Extended Data Table 6.

**Table 2:** Tumor responses per RECIST v1.1 in patients in cohort 4B with measurable disease

Parameter	Overall (*n* = 58)^a^	CLDN18.2 high^b^ (*n* = 47)	CLDN18.2 intermediate^c^ (*n* = 10)
BOR, *n* (%)			
CR	2 (3.4)	2 (4.3)	0
PR	34 (58.6)	30 (63.8)	4 (40.0)
SD	16 (27.6)	10 (21.3)	6 (60.0)
PD	6 (10.3)	5 (10.6)	0
ORR, *n* (%)			
*n* (%)	36 (62.1)	32 (68.1)	4 (40.0)
95% CI	(48.4–74.5)	(52.9–80.9)	(12.2–73.8)
DCR, *n* (%)			
*n* (%)	52 (89.7)	42 (89.4)	10 (100)
95% CI	(78.8–96.1)	(76.9–96.5)	(69.2–100)
DOR			
Months, median (95% CI)	19.1 (10.8–NE)	NE (10.8–NE)	19.1 (3.5–NE)

^a^One patient was missing a value for CLDN18.2 expression.

^b^Defined as ≥75% of tumor cells demonstrating moderate-to-strong membranous CLDN18 staining as determined by central IHC.

^c^Defined as ≥50% to <75% of tumor cells demonstrating moderate-to-strong membranous CLDN18 staining as determined by central IHC.

CR, complete response; NE, not estimable; PD, progressive disease; PR, partial response; SD, stable disease.

### Safety

Safety and dose-limiting tolerability were specifically assessed in cohort 4A as part of the protocol-defined safety analysis. Safety analyses for the full cohort were conducted in the safety analysis set, comprising all 77 patients who received zolbetuximab 800 mg m^−^^2^ in cohort 4A + 4B. At data cutoff, 22 patients (28.6%) remained on zolbetuximab; of the 55 patients who discontinued zolbetuximab, the most common reason was disease progression, observed in 44 patients; five patients discontinued due to physician decision, three due to an adverse event, two due to other reasons and one due to patient death. The median duration of treatment was 288.5 days (range, 1–1,271) for zolbetuximab and 226.0 days (range, 1–872) for nivolumab. Mean relative dose intensity of zolbetuximab was 97.1% (s.d. 11.6) and of nivolumab was 97.0% (s.d. 7.1).

Nearly all patients (98.7%) experienced at least one treatment-emergent adverse event (TEAE), and events of grade 3 or higher severity occurred in approximately two-thirds of patients (66.2%) (Table 3). The most frequently reported events of any grade were nausea (80.5%), decreased appetite (72.7%), neutrophil count decreased (45.5%), peripheral sensory neuropathy (45.5%) and vomiting (37.7%). In general, high-grade gastrointestinal events were infrequent: grade 3 or higher vomiting occurred in 3.9% of patients during the study, and no grade 3 or higher nausea was reported. Additionally, immune-related events (that is, drug hypersensitivity, contrast media allergy and hypersensitivity) were observed in 9.1% of patients. TEAEs considered related to study drugs are described in Extended Data Table 7.

**Table 3:** TEAEs in the safety analysis set

Event, *n* (%)	Cohort 4A + 4B (*n* = 77)	
Any grade	76 (98.7)	
Grade ≥3	51 (66.2)	
Serious	29 (37.7)	
Leading to discontinuation of any drug	38 (49.4)	
TEAEs in ≥10% of patients by preferred terms	Any grade	Grade ≥3
Nausea	62 (80.5)	0
Decreased appetite	56 (72.7)	6 (7.8)
Neutrophil count decreased	35 (45.5)	25 (32.5)
Peripheral sensory neuropathy	35 (45.5)	2 (2.6)
Vomiting	29 (37.7)	3 (3.9)
Diarrhea	28 (36.4)	1 (1.3)
Pyrexia	24 (31.2)	0
Anemia	19 (24.7)	2 (2.6)
Constipation	17 (22.1)	0
Asthenia	15 (19.5)	1 (1.3)
Abdominal pain upper	13 (16.9)	0
Aspartate aminotransferase increased	13 (16.9)	2 (2.6)
Stomatitis	13 (16.9)	1 (1.3)
Alanine aminotransferase increased	11 (14.3)	2 (2.6)
Edema peripheral	11 (14.3)	0
Abdominal pain	10 (13.0)	0
Dysgeusia	10 (13.0)	0
White blood cell count decreased	10 (13.0)	5 (6.5)
Gastritis	9 (11.7)	0
Paresthesia	9 (11.7)	0
Platelet count decreased	9 (11.7)	2 (2.6)
Rash	9 (11.7)	0
Weight decreased	9 (11.7)	0
COVID-19	8 (10.4)	0
Hepatic function abnormal	8 (10.4)	2 (2.6)
Hypoalbuminemia	8 (10.4)	5 (6.5)
Malaise	8 (10.4)	0
Neutropenia	8 (10.4)	5 (6.5)

TEAEs leading to discontinuation of any study drug occurred in 38 patients (49.4%), to discontinuation of zolbetuximab in four patients (5.2%) and to discontinuation of nivolumab in six patients (7.8%) (Extended Data Table 7). The most common TEAEs leading to discontinuation of any study drug were neutrophil count decreased and peripheral sensory neuropathy (both *n* = 15, 19.5%). During the study, five patients (6.5%) experienced a total of six TEAEs leading to death. Reasons for death were malignant neoplasm progression (*n* = 2, 2.6%) and sudden death, pneumonia, pneumonia aspiration and vascular device infection (all *n* = 1, 1.3%).

Unreported secondary endpoints for cohort 4 of ILUSTRO include evaluation of pharmacokinetics and immunogenicity of zolbetuximab in combination with mFOLFOX6 and nivolumab, assessment of health-related quality of life and ORR and PFS measurements by central reviewer.

## Discussion

The multicenter, global ILUSTRO trial was designed to examine zolbetuximab in combination with other anticancer treatments in patients with CLDN18.2-positive mG/GEJ adenocarcinoma. Cohort 4B included patients with previously untreated, CLDN18.2-positive, HER2-negative mG/GEJ adenocarcinoma who received the triplet combination of zolbetuximab, mFOLFOX6 and nivolumab and demonstrated a median PFS of 14.8 months, accompanied by an ORR of 62.1% in ad hoc analyses of patients with measurable lesions. Analyses stratified by CLDN18.2 expression in cohort 4B lend support to targeting this biomarker: patients with high CLDN18.2 expression experienced longer PFS (median, 18.0 months) and, in patients with measurable disease, a higher ORR (68.1%) than those with intermediate expression (6.7 months and 40.0%, respectively), consistent with results from the phase 2 FAST trial of zolbetuximab plus chemotherapy^26^. This pattern was also observed in patients with measurable disease with dual biomarker expression (that is, high CLDN18.2 expression and PD-L1 CPS ≥ 1), where median PFS was 23.6 months and ORR was 74.2%, suggesting that use of both biomarkers as enrollment criteria may achieve optimal patient selection in future trials. Although the descriptive design and absence of a control group prevent definitive assignment of benefit to PD-1 blockade, the expression response gradient and sustained disease control support preclinical observations that targeting both CLDN18.2 and PD-1 may optimally engage the antitumor immune response, particularly in patients with CPS ≥ 1 whose tumors have high CLDN18.2 expression^24,25^. Biomarkers are being further explored in blood and tumor samples that were collected during the study to explore additional response predictors and define immunologic readouts.

PFS was selected as the main endpoint for cohort 4B to align with previous phase 3 zolbetuximab trials, where treatment benefit was most consistently reflected in PFS and OS rather than in ORR. The statistical power was based on an assumption of a median PFS of 12.0 months with the triplet regimen versus 8.5 months with zolbetuximab plus mFOLFOX6, and the median PFS in ILUSTRO among patients with high CLDN18.2 expression compares favorably with this. Notably, this 8.5-month assumption of median PFS for doublet therapy with zolbetuximab plus mFOLFOX6 is longer than the median PFS reported in pivotal trials of immunotherapy plus chemotherapy for mG/GEJ adenocarcinoma^2,4,5^. These immunotherapy plus chemotherapy trials enrolled patients irrespective of CLDN18.2 expression^2,4,5^, and available data suggest that CLDN18.2 status is not prognostic for outcomes with immunotherapy in combination with chemotherapy^27–29^. In cohort 4 of ILUSTRO, the numerically favorable efficacy in patients whose tumors had high CLDN18.2 expression and PD-L1 CPS ≥ 1 versus patients with intermediate expression or CPS < 1 therefore potentially points to a target-driven contribution that complements, rather than duplicates, inhibition of PD-1-mediated activity. Nevertheless, further investigation in randomized, placebo-controlled settings is needed to establish superiority of the triplet strategy.

The safety profile of zolbetuximab plus mFOLFOX6 and nivolumab was manageable and consistent with the known toxicities of the component agents^2,3,16^. Nausea and vomiting were common and generally low grade; however, some grade 3 or higher events were observed. Although the incidence of individual grade 3 or higher events was low, such toxicities can affect quality of life and drug exposure, thereby warranting proactive management. These observations are consistent with the safety profile of zolbetuximab plus chemotherapy, in which nausea and vomiting are typically concentrated in early cycles and can be mitigated with guideline-concordant antiemetic prophylaxis and infusion rate management^6,8,30^. A recent exploratory analysis of the phase 3 SPOTLIGHT and GLOW trials of zolbetuximab plus chemotherapy suggested that prophylactic antiemetic regimens containing three or more drug classes were most effective for the management of these adverse events^31^. This exploratory analysis also explored PFS and OS after censoring patients who experienced nausea and vomiting that led to inadequate dose exposure or early discontinuation and found that median PFS and median OS were numerically longer after censoring^31^.

In cohort 4 of the ILUSTRO study, beyond nausea and vomiting, peripheral sensory neuropathy was observed in 45.5% of patients and, with neutrophil count decreased, was one of the most common TEAEs leading to discontinuation of any study drug. Peripheral neuropathy likely reflected cumulative oxaliplatin exposure, which can be addressed through dose and/or schedule modification^32,33^. Notably, relative dose intensity of zolbetuximab was maintained throughout the ILUSTRO study. Immune-related TEAEs were infrequent and manageable, and no new safety signals emerged with the addition of nivolumab^3,6^. Of note, there was no apparent increase in most gastrointestinal toxicities or immune-mediated gastrointestinal events with triplet therapy compared to previous studies of zolbetuximab plus chemotherapy^6,8,16^.

Limitations of this study include its non-randomized design and the modest sample size. Another key limitation is the immaturity of OS data, with only 35.2% of patients in cohort 4B experiencing an OS event by data cutoff. Although median PFS was longer than the median follow-up, this was driven by censoring related to early treatment discontinuation rather than data immaturity. Censoring events included discontinuation due to adverse events, clinical disease progression and initiation of subsequent treatments and were not considered PFS events according to the protocol. Nevertheless, these data should be interpreted with caution. Additionally, the small number of patients with intermediate CLDN18.2 expression limits the robustness of comparisons between high and intermediate subgroups. In this analysis, efficacy endpoints were assessed by investigators, which introduced potential for bias; however, the historical control was likewise based on investigator-assessed PFS. Notably, in the pivotal phase 3 studies of zolbetuximab plus chemotherapy, investigator-assessed median PFS was numerically shorter than centrally assessed PFS^6,8^.

The results from cohort 4 of ILUSTRO have informed the design of the ongoing, global, randomized phase 3 LUCERNA trial evaluating triplet therapy with zolbetuximab plus chemotherapy with PD-1 inhibition in patients with HER2-negative, locally advanced unresectable or mG/GEJ adenocarcinoma whose tumors are CLDN18.2 positive and have a PD-L1 CPS ≥ 1 (NCT06901531). Pending positive findings from LUCERNA, a triplet therapy approach may represent a rational, biomarker-guided first-line strategy for patients with CLDN18.2-positive, PD-L1-positive, HER2-negative, locally advanced mG/GEJ adenocarcinoma.

## Methods

### Ethical approval and consent

The protocol and all amendments were approved by the appropriate ethics committee or institutional review board at each participating institution (see the list of study sites in the Supplementary Information). The study was conducted in accordance with Declaration of Helsinki and Council for International Organizations of Medical Sciences International ethical guidelines, applicable International Council for Harmonization Good Clinical Practice guidelines and applicable laws and regulations. All patients provided written informed consent.

### Study design and participants

This international trial is registered with ClinicalTrials.gov (NCT03505320); materials were first submitted on 29 March 2018. The study protocol and statistical analysis plan are included in the Supplementary Information. The final analysis of cohort 4 of ILUSTRO included two subcohorts: the safety lead-in (4A) and the extension phase (4B). Cohort 4A was designed to confirm the tolerability and safety of the triplet regimen over a 2-week dose-limiting toxicity period. The tolerability of the applied dose was assessed during a tolerability evaluation meeting before proceeding to cohort 4B. Baseline characteristics, demographics and safety were assessed in all patients in cohort 4A + 4B who received the loading dose of zolbetuximab 800 mg m^−^^2^. Primary efficacy analyses were conducted in cohort 4B, and exploratory efficacy analyses were conducted in cohort 4A + 4B. Subgroup analyses included outcomes by CLDN18.2 expression (high versus intermediate). Within cohort 4, the safety analysis set included all patients who received at least one dose of zolbetuximab and was used for summaries of demographic and baseline characteristics, PFS, OS and all safety assessments, as outlined in the study protocol and statistical analysis plan. The full analysis set included all patients who received at least one dose of zolbetuximab and who had at least one posttreatment disease assessment and was used to assess tumor response. Ad hoc analyses of patients with measurable disease per Response Evaluation Criteria in Solid Tumors version 1.1 (RECIST v1.1)^34^ were also conducted.

Eligible patients were adults (according to local regulation at the time of signing informed consent) with radiographically confirmed locally advanced unresectable or mG/GEJ adenocarcinoma (per RECIST v1.1) who had an imaging scan to determine eligibility within 28 days before the first dose of study treatment. Patients were recruited from hospitals and cancer centers, and a list of study sites is included in the Supplementary Information. Male and female patients were included, and sex was collected as a demographic variable and recorded in the electronic case report form. Only biological sex (male or female) was captured; gender identity was not collected in this study. The study was a single-arm trial and was not designed to evaluate treatment effects by sex or gender. Therefore, no sex-based or gender-based stratification or subgroup analyses were performed. Patients had high or intermediate CLDN18.2 expression, defined as moderate-to-strong membranous CLDN18 staining in ≥75% of tumor cells (high) or in ≥50% and <75% of tumor cells (intermediate), as determined by central IHC testing, using the investigational VENTANA CLDN18 (43-14A) RxDx Assay (Roche Diagnostics Solutions), and were HER2 negative based on local or central testing results. Patients were evaluated for PD-L1 status based on centrally assessed CPS as determined by the Dako PD-L1 IHC 28-8 pharmDx assay. Patients had not received previous systemic anticancer therapy for advanced disease, including previous checkpoint inhibitor therapy. Patients may have received neoadjuvant and/or fluorouracil-containing adjuvant chemotherapy that had been completed at least 6 months before the first dose of study treatment. Systemic immunosuppressive therapy, including systemic corticosteroids, 14 days before first dose of study treatment was prohibited. Finally, patients should not have had allergic reactions to any of the treatments or their components. A complete list of inclusion and exclusion criteria is included in the Supplementary Information.

### Treatments

Treatments were administered every 2 weeks on days 1, 15 and 29 of every 42-day cycle in the following sequence: zolbetuximab, nivolumab (approximately 30 minutes after the end of the zolbetuximab infusion) and mFOLFOX6. Antiemetic premedication was given before zolbetuximab administration.

Zolbetuximab was administered as a minimum 2-hour intravenous infusion that could be interrupted or slowed to manage toxicity as needed. Based on the results from cohort 4A, a loading dose of zolbetuximab 800 mg m^−^^2^ followed by subsequent doses of 400 mg m^−^^2^ every 2 weeks was considered tolerable in combination with mFOLFOX6 and nivolumab; this dosing regimen was, therefore, used for cohort 4B. The mFOLFOX6 and nivolumab regimen was based on CheckMate 649 (ref. ^2^). Nivolumab was administered intravenously at a dose of 240 mg infused over 30 minutes, and dose reductions were not permitted. Individual components of mFOLFOX6 were as follows: oxaliplatin: 85 mg m^−^^2^ intravenous infusion over 2 hours; leucovorin: 400 mg m^−2^intravenous infusion over 2 hours (or levofolinic acid given either at the protocol-recommended doses or as deemed appropriate by the investigator in accordance with institutional standard of care); and 5-fluorouracil (5-FU): 400 mg m^−^^2^ intravenous bolus infused over 5–15 minutes followed by a continuous 46–48-hour 5-FU infusion of 2,400 mg m^−^^2^. Administration of mFOLFOX6 continued for ≤12 treatments (four cycles), and, beginning at cycle 5, patients could remain on 5-FU and leucovorin or folinic acid along with zolbetuximab plus nivolumab for the remainder of the study per the investigator’s discretion.

### Endpoints and assessments

The primary endpoint of ILUSTRO was ORR of zolbetuximab as a single agent, as assessed by an independent central reader. These data were determined as part of cohort 1 and were previously published^3^. The main efficacy endpoint of interest for ILUSTRO cohort 4 was PFS, and other secondary endpoints of interest were ORR, DCR, DOR, OS and safety and tolerability. Although ORR was initially defined as the primary endpoint in cohort 4 during early study planning, the endpoint was amended to PFS through a prospectively implemented protocol amendment prior to dosing in cohort 4B. This amendment aimed to use the more appropriate endpoint of PFS after the primary readout of the phase 3 SPOTLIGHT trial, in which zolbetuximab plus chemotherapy had shown an effect on PFS but not on ORR. Accordingly, the sample size calculation in cohort 4B was based on investigator-assessed PFS of zolbetuximab plus mFOLFOX6, as specified in the most recent version of the study protocol and statistical analysis plan (Supplementary Information). Responses and disease progression were evaluated using RECIST v1.1 and were radiologically assessed by investigators; confirmation of response required repeat assessment at least 4 weeks after the initial documentation. Imaging was evaluated every 8 weeks (±1) from cycle 1 day 1 for the first 56 weeks and then every 12 weeks (±2) thereafter, until disease progression or study discontinuation.

TEAEs and serious TEAEs, regardless of causality, were collected from the time of informed consent through 90 days after the last dose of any study drug (except for inpatient hospitalization for planned procedures) and were graded according to National Cancer Institute Common Terminology Criteria for Adverse Events 4.03 guidelines. Patients who discontinued zolbetuximab, mFOLFOX6 (all components) and/or nivolumab had a study discontinuation visit and safety follow-up visits 30 days and 90 days after their last dose of any study drug.

### Statistical analysis

The sample size of cohort 4A was not based on a statistical power calculation but was expected to provide safety information to determine the tolerability of the dose level of interest. The expected sample size of approximately 65 patients for cohort 4B was not based on strict statistical consideration but was expected to yield 50 patients whose tumors had high CLDN18.2 expression based on population prevalence estimates. For patients with high CLDN18.2 expression, assuming an accrual period of 12 months and a follow-up period of 3–6 months and 20–25 PFS events, the sample size of 50 was expected to provide 70.37–76.13% power to detect the difference in PFS with the assumption of a 12-month median PFS for the triplet therapeutic regimen versus an 8.5-month median PFS (zolbetuximab plus mFOLFOX6 and nivolumab versus zolbetuximab and mFOLFOX6) using a one-sided 15% type I error. It was assumed that the survival time distributions of both groups were approximated reasonably well by the Weibull distribution with a shape parameter of 1 (exponential distribution).

PFS was defined as the time from date of treatment start until the date of radiographic disease progression or until death due to any cause, whichever was earliest. The survival curve, median PFS and PFS rates at 6 months, 12 months, 24 months and 36 months were estimated using the Kaplan−Meier method with corresponding 95% CIs. OS was defined as the time from the date of treatment start until the documented date of death from any cause. All events of death were included, regardless of whether the event occurred while the patient was still taking the study drug. Patients who were still alive at the time of analysis were censored on the last day known to be alive. OS was analyzed using the Kaplan−Meier method. Best overall response (BOR) was determined once all tumor response data for the patient were available, and patients were classified by best response on study as outlined in RECIST v1.1. ORR was defined as the proportion of patients with complete response or partial response based on BOR and summarized using an exact 95% CI. The DCR was defined as the proportion of patients with complete response, partial response or stable disease based on BOR and summarized using the same method as for ORR, including exact 95% CI. DOR was defined as the time from the date of the first complete response/partial response (whichever was first recorded) to the date of radiographic progression/death or date of censoring; distribution was estimated using Kaplan−Meier methodology with median and 95% CI. No formal hypothesis testing was prespecified; analyses were descriptive and exploratory.

### Study protocol amendment history

The ILUSTRO study protocol underwent several amendments with substantial changes. The first of these was amendment 4, which added cohorts 4A and 4B and removed cohort 3B, approved on 25 February 2021. Amendment 5 was approved on 22 August 2022; the substantial changes were to clarify the dosing and structure of the dose-limiting toxicity analysis and to adjust the statistical parameters for the sample size calculation and for the efficacy endpoints. The next substantial amendment (6) was approved on 2 August 2023; substantial changes were to add cohort 5 and to revise cohort 4 to use PFS to determine the sample size calculation. Substantial amendment 7 removed the enrollment cap and was approved on 23 May 2024. Finally, amendment 8 was approved on 22 November 2024, with substantial changes to update the classification of selected study drugs and to update the adverse reactions that would require dose modifications of nivolumab.

### Reporting summary

Further information on research design is available in the Nature Portfolio Reporting Summary linked to this article.

## Online content

Any methods, additional references, Nature Portfolio reporting summaries, source data, extended data, supplementary information, acknowledgements, peer review information; details of author contributions and competing interests; and statements of data and code availability are available at 10.1038/s41591-026-04306-9.

## Supplementary information

Supplementary Information Inclusion/exclusion criteria, List of study sites, Study protocol and Study statistical analysis plan.

Reporting Summary

## Figures and Tables

**Extended Data Table 1:**
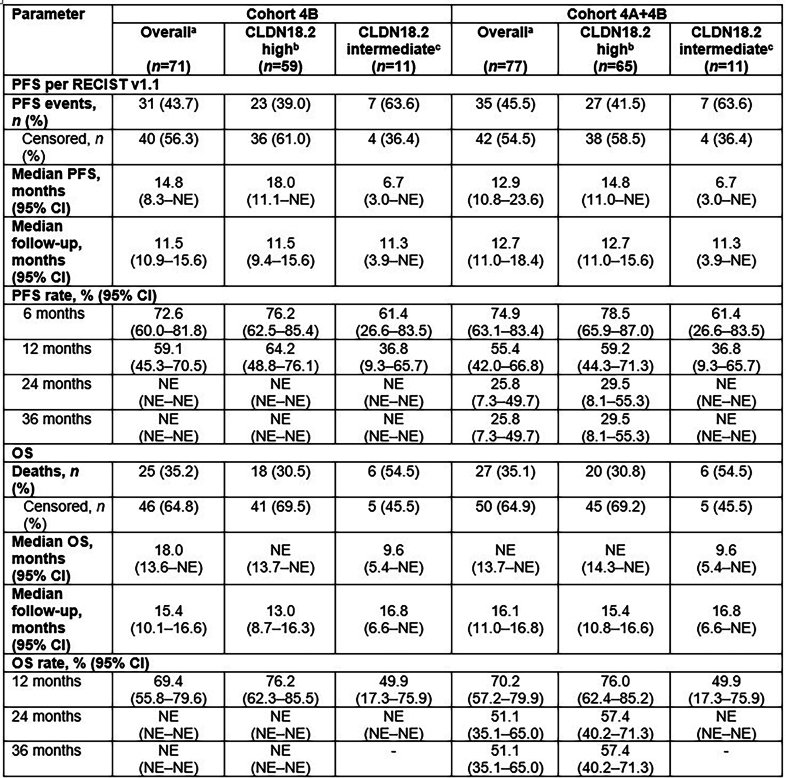
Survival outcomes in the safety analysis set

^a^One patient was missing a value for CLDN18.2 expression.

^b^Defined as ≥75% of tumor cells demonstrating moderate-to-strong membranous CLDN18 staining as determined by central IHC.

^c^Defined as ≥50% but <75% of tumor cells demonstrating moderate-to-strong membranous CLDN18 staining as determined by central IHC.

Efficacy was assessed as an exploratory analysis in cohort 4 A + 4B.

CI, confidence interval; CLDN18, claudin 18; CLDN18.2, claudin 18 isoform 2; IHC, immunohistochemistry; NE, not estimable; OS, overall survival; PFS, progression-free survival; RECIST v1.1, Response Evaluation Criteria in Solid Tumors version 1.1.

**Extended Data Table 2:**
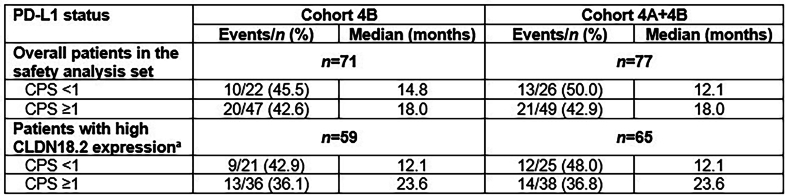
PFS by PD-L1 CPS in the safety analysis set

Efficacy was assessed as an exploratory analysis in cohort 4 A + 4B. One patient was missing a value for CLDN18.2 expression. Two patients were missing PD-L1 CPS values.

^a^Defined as ≥75% of tumor cells demonstrating moderate-to-strong membranous CLDN18 staining as determined by central IHC.CLDN18.2, claudin 18 isoform 2; CPS, combined positive score; IHC, immunohistochemistry; PD-L1, programmed death-ligand 1; PFS, progression-free survival.

**Extended Data Table 3:**
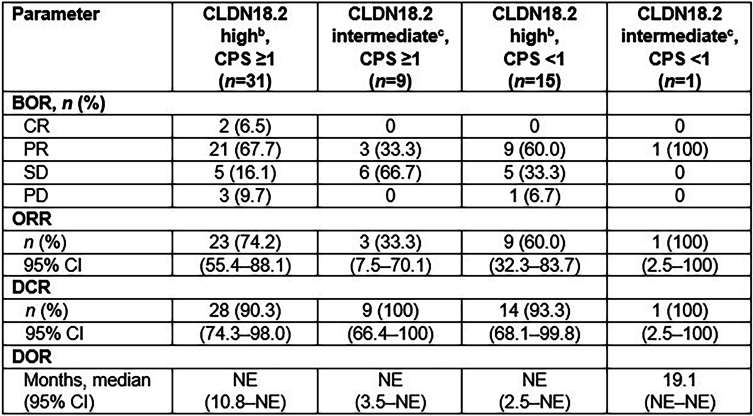
Ad hoc analysis of tumor responses per RECIST v1.1 in patients with measurable disease in cohort 4B by CLDN18.2 expression^a^ and PD-L1 CPS

^a^One patient was missing a value for CLDN18.2 expression. Two patients were missing PD-L1 CPS values.

^b^Defined as ≥75% of tumor cells demonstrating moderate-to-strong membranous CLDN18 staining as determined by central IHC.

^c^Defined as ≥50% to <75% of tumor cells demonstrating moderate-to-strong membranous CLDN18 staining as determined by central IHC.

BOR, best overall response; CI, confidence interval; CLDN18, claudin 18; CLDN18.2, claudin 18 isoform 2; CR, complete response; DCR, disease control rate; DOR, duration of response; IHC, immunohistochemistry; NE, not estimable; ORR, objective response rate; PD, progressive disease; PR, partial response; RECIST v1.1, Response Evaluation Criteria in Solid Tumors version 1.1; SD, stable disease.

**Extended Data Table 4:**
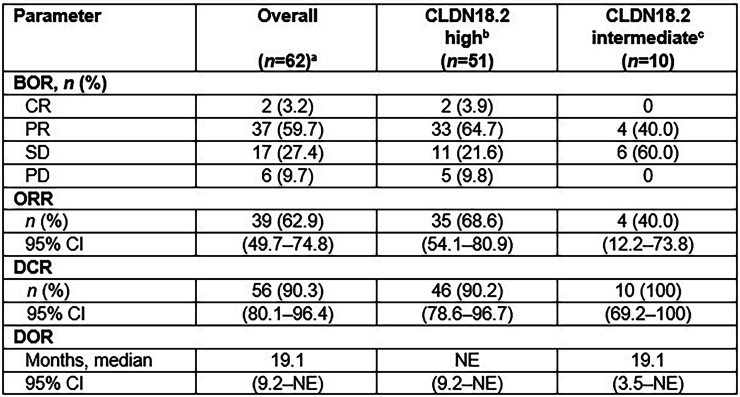
Ad hoc analysis of tumor responses per RECIST v1.1 in patients with measurable disease in cohort 4A + 4B

^a^One patient was missing a value for CLDN18.2 expression.

^b^Defined as ≥75% of tumor cells demonstrating moderate-to-strong membranous CLDN18 staining as determined by central IHC.

^c^Defined as ≥50% to <75% of tumor cells demonstrating moderate-to-strong membranous CLDN18 staining as determined by central IHC.

BOR, best overall response; CI, confidence interval; CLDN18, claudin 18; CLDN18.2, claudin 18 isoform 2; CR, complete response; DCR, disease control rate; DOR, duration of response; IHC, immunohistochemistry; NE, not estimable; ORR, objective response rate; PD, progressive disease; PR, partial response; RECIST v1.1, Response Evaluation Criteria in Solid Tumors version 1.1; SD, stable disease.

**Extended Data Table 5:**
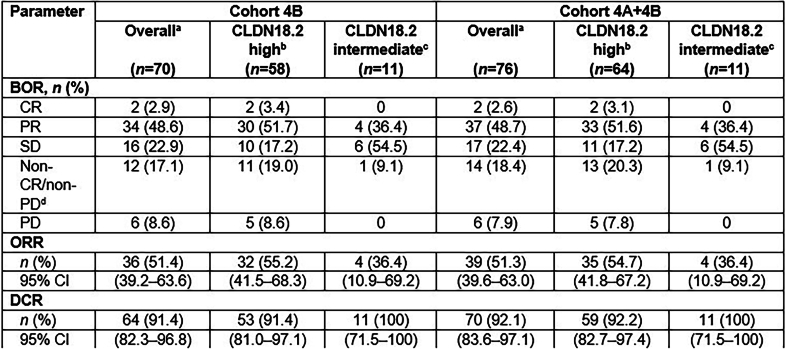
Tumor responses per RECIST v1.1 in the full analysis set

^a^One patient was missing a value for CLDN18.2 expression.

^b^Defined as ≥75% of tumor cells demonstrating moderate-to-strong membranous CLDN18 staining as determined by central IHC.

^c^Defined as ≥50% to <75% of tumor cells demonstrating moderate-to-strong membranous CLDN18 staining as determined by central IHC.

^d^Based on RECIST v1.1 definitions and included patients who could not classified as CR, PR, SD, or PD.

Efficacy was assessed as an exploratory analysis in cohort 4 A + 4B.

BOR, best overall response; CI, confidence interval; CLDN18, claudin 18; CLDN18.2, claudin 18 isoform 2; CR, complete response; DCR, disease control rate; IHC, immunohistochemistry; NE, not estimable; ORR, objective response rate; PD, progressive disease; PR, partial response; RECIST v1.1, Response Evaluation Criteria in Solid Tumors version 1.1; SD, stable disease.

**Extended Data Table 6:**
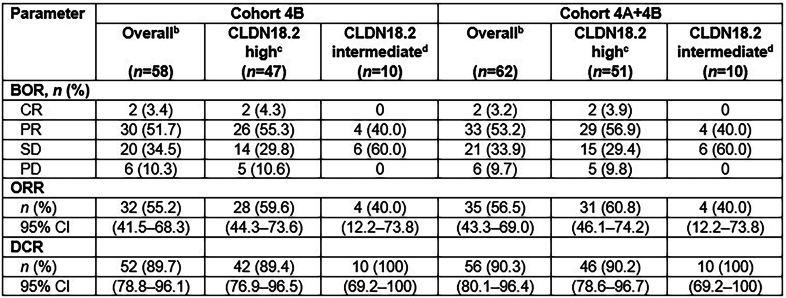
Ad hoc analysis of confirmed tumor responses^a^ per RECIST v1.1 in patients with measurable disease

^a^Defined as responses with a second scan ≥4 weeks after the first documentation of response.

^b^One patient was missing a value for CLDN18.2 expression.

^c^Defined as ≥75% of tumor cells demonstrating moderate-to-strong membranous CLDN18 staining as determined by central IHC.

^d^Defined as ≥50% to <75% of tumor cells demonstrating moderate-to-strong membranous CLDN18 staining as determined by central IHC.

Efficacy was assessed as an exploratory analysis in cohort 4 A + 4B.

BOR, best overall response; CI, confidence interval; CLDN18, claudin 18; CLDN18.2, claudin 18 isoform 2; CR, complete response; DCR, disease control rate; IHC, immunohistochemistry; NE, not estimable; ORR, objective response rate; PD, progressive disease; PR, partial response; RECIST v1.1, Response Evaluation Criteria in Solid Tumors version 1.1; SD, stable disease.

**Extended Data Table 7:**
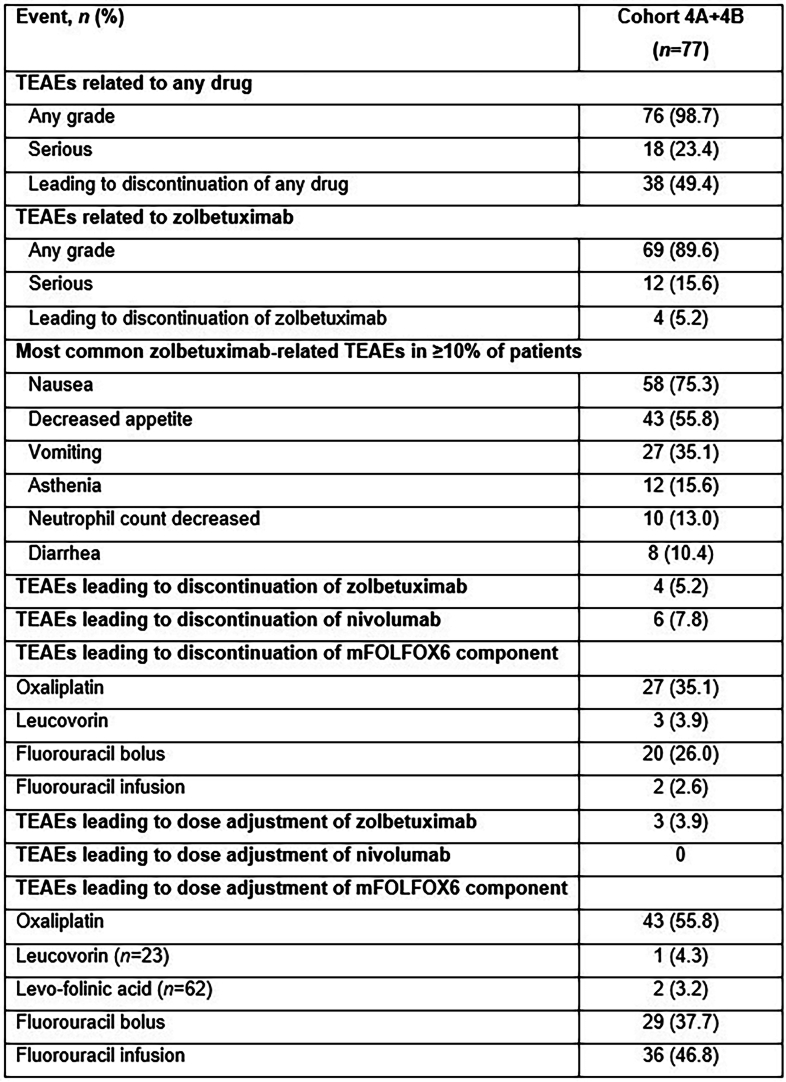
Additional safety parameters in the safety analysis set

TEAE, treatment-emergent adverse event.

## Data Availability

The data underlying this paper can be requested from the study sponsor, Astellas Pharma. Details for how researchers may request access to information from Astellas-sponsored clinical trials can be found at https://www.clinicaltrials.astellas.com/transparency/. Subject to compliance with the applicable laws and regulations relevant to protection of personal data, Astellas provides a platform (https://vivli.org/) where researchers may request access to participant-level data, trial-level data and protocols from Astellas-sponsored clinical trials with a medicinal product conducted in patients that are completed after 1 January 2010. Access to these data is granted for medicinal products and indications approved in any country after the request has been reviewed and approved by an independent panel of experts (‘Scientific Review Board’) based on scientific merit and the qualifications of the researcher. Requestors must be affiliated with an accredited research institution, have relevant education and qualifications, disclose conflicts of interest, submit a detailed research proposal and statistical analysis plan, include a qualified statistician on the team and sign a data use agreement before access is granted. Access is given by Astellas after review and approval by the Scientific Review Board and execution of a data-sharing agreement. If certain data elements cannot be made available due to privacy, ethical or regulatory limitations, this will be communicated to the requester. Before participant-level data are shared, they are anonymized to respect the rights of the clinical trial patients to privacy and to protect their personal health information. Anonymized data will be shared in the Vivli secure research environment. On average, it takes a few months to access data after submitting a request, but the timeline can vary based on the number of data contributors, studies involved and the requester’s responsiveness to comments. Further details on Astellas’ data-sharing criteria and process for requesting access can be found on the Astellas Member Page at https://vivli.org/.
