# Supplementary material for: First-line zolbetuximab plus mFOLFOX6 and nivolumab in unresectable CLDN18.2-positive gastric or gastroesophageal junction adenocarcinoma: a phase 2 trial
Source: Nat Med. 2026 Mar 16;32(6):2182–90. doi: 10.1038/s41591-026-04306-9 (PMC13278966; doi:10.1038/s41591-026-04306-9)
Supplement: Supplementary file 1 — Inclusion/exclusion criteria, List of study sites, Study protocol and Study statistical analysis plan. [file 41591_2026_4306_MOESM1_ESM.pdf]

nature medicine

Supplementary information https://doi.org/10.1038/s41591-026-04306-9

First-line zolbetuximab plus mFOLFOX6 and nivolumab in unresectable CLDN18.2-positive gastric or gastroesophageal junction adenocarcinoma: a phase 2 trial

In the format provided by the authors and unedited

SUPPLEMENTARY INFORMATION

Inclusion/exclusion criteria from version 8.0 of the ILUSTRO study protocol

ILUSTRO inclusion criteria

1. Institutional Review Board (IRB)/Independent Ethics Committee (IEC) approved written informed consent and privacy language as per national regulations (e.g., Health Insurance Portability and Accountability Act [HIPAA] Authorization for US sites) must be obtained from the patient or legally authorized representative prior to any study-related procedures (including withdrawal of prohibited medication, if applicable).
2. Patient is considered an adult according to local regulation at the time of signing informed consent.
3. Female patient eligible to participate if she is not pregnant and at least one of the following conditions applies:

   a. Not a woman of child-bearing potential (WOCBP) as defined in Appendix 12.6 Contraception Requirements

   OR

   b. WOCBP who agrees to follow the contraceptive guidance as defined in Appendix 12.6 Contraception Requirements throughout the treatment period and for at least 9 months after the final oxaliplatin administration and 6 months after the final administration of all other study drugs
4. Female patient must agree not to breastfeed starting at screening and throughout the study period, and for 6 months after the final study drug administration.
5. Female patient must agree not to donate ova starting at screening and throughout the study period, and for 9 months after the final oxaliplatin administration and 6 months after the final administration of all other study drugs.
6. A sexually active male patient with a female partner(s) who is of child-bearing potential must agree to use contraception during the treatment period and for at least 6 months after the final study drug administration.
7. Male patient must agree not to donate sperm starting at screening and throughout the study period, and for 6 months after the final study drug administration.
8. Male patient with a pregnant or breastfeeding partner(s) must agree to remain abstinent or use a condom for the duration of the pregnancy or time partner is breastfeeding throughout the study period and for 6 months after the final study drug administration.
9. Patient has histologically confirmed gastric or GEJ adenocarcinoma.
10. Patient has radiographically confirmed, locally advanced, unresectable or metastatic disease within 28 days prior to the first dose of study treatment.
11. Patient’s tumor is positive for CLDN18.2 expression demonstrating moderate to strong membranous staining as determined by central IHC testing.
12. Patient agrees not to participate in another interventional study while on treatment.
13. Patient has ECOG performance status 0 to 1.
14. Patient has predicted life expectancy ≥ 12 weeks in the opinion of the investigator.

15. Patient must meet all of the following criteria based on the centrally or locally analyzed laboratory tests collected within 14 days prior to the first dose of study treatment. In case of multiple central laboratory data within this period, the most recent data should be used.

- Hemoglobin (Hgb) ≥ 9 g/dL (transfusion is allowed, but post-transfusion Hgb [24 hours or later following transfusion] must be ≥ 9 g/dL)
- Absolute neutrophil count (ANC) ≥ 1.5 × 10^9/L
- Platelets ≥ 100 × 10^9/L
- Albumin ≥ 2.5 g/dL
- Total bilirubin ≤ 1.5 × upper limit of normal (ULN)
- Aspartate aminotransferase (AST) and alanine aminotransferase (ALT) ≤ 2.5 × ULN in patients without liver metastases (≤ 5 × ULN if liver metastases are present)
- Estimated creatinine clearance ≥ 30 mL/min
- Prothrombin time/international normalized ratio and partial thromboplastin time ≤ 1.5 × ULN (except for patients receiving anticoagulation therapy)

Inclusion criteria specific to cohort 4

1. Patient has radiologically evaluable disease (measurable and/or non-measurable) according to RECIST 1.1, per local assessment, ≤ 28 days prior to the first dose of study treatment. For patients with only 1 evaluable lesion and prior radiotherapy ≤ 3 months before enrollment, the lesion must either be outside the field of prior radiotherapy or must have documented radiographic progression following radiation therapy.
2. Patient has not received prior systemic anti-cancer therapy for their advanced disease (patient may have received neoadjuvant and/or fluorouracil-containing adjuvant chemotherapy as long as it has been completed ≥ 6 months before the first dose of study treatment).
3. Patient has a gastric or GEJ tumor that is HER2-negative as determined by local or central testing.
4. Patient has not received prior checkpoint inhibitor therapy.

Specific to cohort 4B only:

1. Patient must have an additional available tumor specimen collected within 3 months prior to the first dose of study treatment.
2. Patient must be an appropriate candidate for a tumor biopsy and is amenable to undergo a tumor biopsy during the screening period (if applicable) and treatment period as indicated in the Schedule of Assessments.

ILUSTRO exclusion criteria

1. Patient has had prior severe allergic reaction or intolerance to known ingredients of zolbetuximab or other monoclonal antibodies, including humanized or chimeric antibodies.

2. Patient has known immediate or delayed hypersensitivity or contraindication to any component of study treatment.
3. Patient has received other investigational agents or devices concurrently or within 28 days prior to first dose of study treatment.
4. Patient has received systemic immunosuppressive therapy, including systemic corticosteroids 14 days prior to first dose of study treatment. Patients using a physiologic replacement dose of hydrocortisone or its equivalent (defined as up to 30 mg per day of hydrocortisone or up to 10 mg per day of prednisone), receiving a single dose of systemic corticosteroids or receiving systemic corticosteroids as pre-medication for radiologic imaging contrast use are allowed.
5. Patient has a complete gastric outlet syndrome or a partial gastric outlet syndrome with persistent recurrent vomiting.
6. Per investigator judgment, patient has significant gastric bleeding and/or untreated gastric ulcers that would preclude the patient from participation per investigator judgment.
7. Patient has history of central nervous system metastases and/or carcinomatous meningitis from gastric/GEJ cancer.
8. Patient has a known history of a positive test for human immunodeficiency virus (HIV) infection or known active hepatitis B (positive hepatitis B surface antigen [HBsAg]) or hepatitis C infection. NOTE: Screening for these infections should be conducted per local requirements.
   - For patients who are negative for HBs Ag, but HBc Ab positive, an HB DNA test will be performed and if positive the patient will be excluded.
   - Patients with positive hepatitis C virus (HCV) serology, but negative HCV RNA test results are eligible.
   - Patients treated for HCV with undetectable viral load results are eligible.
9. Patient has had within 6 months prior to first dose of study treatment any of the following: unstable angina, myocardial infarction, ventricular arrhythmia requiring intervention or hospitalization for heart failure.
10. Patient has active infection requiring systemic therapy that has not completely resolved within 7 days prior to the start of study treatment.
11. Patient has active autoimmune disease that has required systemic treatment within the past 3 months prior to the start of study treatment.
12. Patient has a clinically significant disease or co-morbidity that in the opinion of the investigator may adversely affect the safe delivery of treatment within this study or make the patient unsuitable for study participation.
13. Patient has psychiatric illness or social situations that would preclude study compliance per investigator’s judgment.
14. Patient has had a major surgical procedure ≤ 28 days before start of study treatment.
15. Patient is without complete recovery from a major surgical procedure ≤14 days before start of study treatment.

16. Patient has received radiotherapy for locally advanced unresectable or metastatic gastric or GEJ adenocarcinoma ≤ 28 days prior to start of study treatment and has NOT recovered from any related toxicity.

17. Patient has another malignancy for which treatment is required, per investigator's clinical judgment.

Exclusion criteria specific to cohort 4

18. Patient has any of the following:
    - Prior severe allergic reaction or intolerance to any component of mFOLFOX6 or FLOT chemotherapeutics in this study.
    - Known dihydropyrimidine dehydrogenase (DPD) deficiency (screening for DPD deficiency should be conducted per local requirements).
    - Known peripheral neuropathy > grade 1 (absence of deep tendon reflexes as the sole neurological abnormality does not render the patient ineligible).
    - Sinusoidal obstruction syndrome, formerly known as veno-occlusive disease, if present, should be stable or improving per investigator's judgment.
    - History of clinically significant ventricular arrhythmias (i.e., sustained ventricular tachycardia, ventricular fibrillation, or Torsades de Pointes).
    - QTc interval > 450 msec for male patients; QTc interval > 470 msec for female patients.
    - History or family history of congenital long QT syndrome.
    - Cardiac arrhythmias requiring anti-arrhythmic medications (Patients with rate controlled atrial fibrillation for > 1 month prior to first dose of study treatment are eligible).
19. Patient has any of the following:
    - Patient with ongoing or previous autoimmune disease or interstitial lung disease, active diverticulitis or gastrointestinal ulcerative disease, or solid organ or stem cell transplant, or other uncontrolled or clinically significant medical disorders
    - Patient with type 1 diabetes mellitus, endocrinopathies stably maintained on appropriate replacement therapy or skin disorders (e.g., vitiligo, psoriasis, or alopecia) not requiring systemic treatment are allowed.
    - Patient has known history of serious hypersensitivity reaction to a known ingredient of pembrolizumab or nivolumab.
    - Cohort 4B Only: Patient with known microsatellite instability-high or mismatch repair deficient tumors

List of study sites

Site name	Principal investigator name	Committee that approved the protocol
Hopital Morvan - CHU Brest	Jean Philippe Metges	
Hopital Haut Leveque	Denis Smith	Comite de Protection des Personnes Sud-Est 1 CHU de Saint-Etienne – Hopital de Bellevue
Hopital Europeen Georges Pompidou	Aziz Zaanan	
Seconda Universita degli Studi di Napoli, AOU	Ferdinando De Vita	
SO S.Chiara, AOU Pisana	Gianluca Masi	Comitato Etico Degli IRCCS Istituto Europeo di Oncologia e Centro Cardiologico Monzino
Istituto Oncologico Veneto	Sara Lonardi	
Istituto Europeo di Oncologia	Nicola Fazio	
National Cancer Center Hospital East	Kohei Shitara	National Cancer Central Institutional Review Board
National Cancer Center Hospital	Hirokazu Shoji	National Cancer Central Institutional Review Board
The Cancer Institute Hospital of JFCR	Kensei Yamaguchi	Cancer Institute Hospital of JFCR Institutional Review Board
Seoul National University Hospital	Tae Yong Kim	Seoul National University College of Medicine / Seoul National University Hospital Institutional Review Board
Seoul National University Bundang Hospital	Keun Wook Lee	Seoul National University Bundang Hospital institutional review Board
China Medical University Hospital	Li Yuan Bai	Research Ethics Committee, China Medical University & Hospital

A Phase 2 Study of Zolbetuximab (IMAB362) as Monotherapy and in Combination with Chemotherapy and/or Immunotherapy in Subjects with Metastatic or Locally Advanced Unresectable Gastric or Gastroesophageal Junction (GEJ) Adenocarcinoma and Locoregional Gastric or GEJ Adenocarcinoma Whose Tumors are Claudin (CLDN) 18.2-Positive

A Study of Zolbetuximab (IMAB362) in Adults with Gastric Cancer

ISN/Protocol 8951-CL-0103

Version 8.0

Amendment 7 [Substantial]

23 May 2024

ILUSTRO

IND 129598

EU CT 2024-511649-21

Astellas Pharma Global Development Inc. (APGD)

Northbrook, IL 60062

Protocol History:
Version 1.0 [13 Dec 2017]
Version 2.0 [08 Feb 2018] Incorporating Substantial Amendment 1
Version 3.0 [03 Jul 2018] Incorporating Substantial Amendment 2
Version 4.0 [10 Mar 2020] Incorporating Substantial Amendment 3
Version 5.0 [25 Feb 2021] Incorporating Substantial Amendment 4
Version 5.1 [21 Oct 2021] Incorporating Nonsubstantial Amendment 1
Version 6.0 [22 Aug 2022] Incorporating Substantial Amendment 5
Version 7.0 [02 Aug 2023] Incorporating Substantial Amendment 6
Version 8.0 [23 May 2024] Incorporating Substantial Amendment 7

The information contained in this document is supplied as a background for clinical investigations. This document contains confidential information which is the intellectual property of Astellas. By accepting or reviewing this document, you agree to hold this information in confidence and not copy or disclose it to others or use it for unauthorized purposes except (1) as otherwise agreed to in writing; (2) where required by applicable law; (3) where disclosure is directly related to the care and safety of the research participant; and (4) where disclosure of such information is made to a member of the investigator's team who agrees to hold this information in confidence.

Table of Contents

I. SIGNATURES ... 11
II. CONTACT DETAILS OF KEY SPONSOR'S PERSONNEL ... 13
III. LIST OF ABBREVIATIONS AND DEFINITION OF KEY TERMS ... 14
IV. PROTOCOL AMENDMENT SUMMARY OF CHANGES ... 19
V. SYNOPSIS ... 24
VI. FLOW CHART AND SCHEDULE OF ASSESSMENTS ... 48
1 INTRODUCTION ... 113
1.1 Background ... 114
1.2 Nonclinical and Clinical Data ... 115
1.2.1 Nonclinical Data ... 115
1.2.2 Clinical Data ... 116
1.3 Summary of Key Safety Information for Study Drugs ... 117
1.4 Efficacy ... 118
1.4.1 Efficacy Results from Study GM-IMAB-001-02 (MONO) ... 118
1.4.2 Efficacy Results from Study GM-IMAB-001-03 (FAST) ... 118
1.4.3 Efficacy of mFOLFOX6 ... 119
1.4.4 Efficacy of mFOLFOX6 and Nivolumab ... 119
1.4.5 Efficacy of FLOT ... 120
1.5 Risk Benefit Assessment ... 120
2 STUDY OBJECTIVE(S), DESIGN AND ENDPOINTS ... 120
2.1 Study Objectives ... 120
2.1.1 Primary Objective ... 120
2.1.2 Secondary Objectives ... 120
2.1.3 Exploratory Objective ... 121
2.2 Study Design and Dose Rationale ... 121
2.2.1 Study Design ... 121
2.2.2 Dose Rationale ... 130
2.3 Endpoints ... 131
2.3.1 Primary Endpoints ... 131
2.3.2 Secondary Endpoints ... 131
2.3.3 Exploratory Endpoints ... 132
3 STUDY POPULATION ... 133

3.1 Selection of Study Population 133
3.1.1 Cohort 1A 133
3.1.2 Cohort 2 133
3.1.3 Cohort 3A 134
3.1.4 Cohort 4 134
3.1.5 Cohort 5 134
3.2 Inclusion Criteria 134
3.3 Exclusion Criteria 138
4 TREATMENT 140
4.1 Identification of Investigational Medicinal Product 140
4.1.1 Study Drug 140
4.1.2 Other Drugs 140
4.1.2.1 mFOLFOX6 (Cohorts 2 and 4 Only) 140
4.1.2.2 Pembrolizumab (Cohort 3 Only) 140
4.1.2.3 Nivolumab (Cohort 4 Only) 141
4.1.2.4 FLOT (Cohort 5 Only) 141
4.1.3 Comparative Drug(s) 141
4.2 Packaging and Labeling 141
4.3 Study Drug Handling 141
4.3.1 Zolbetuximab 142
4.3.1.1 Observation Period Following Zolbetuximab Infusion 143
4.3.2 0.9% Sodium Chloride Injection 143
4.3.3 mFOLFOX6 (Cohort 2) 143
4.3.3.1 Oxaliplatin 143
4.3.3.2 Leucovorin 144
4.3.3.3 Fluorouracil 144
4.3.4 Pembrolizumab (Cohort 3) 144
4.3.5 mFOLFOX6 and Nivolumab (Cohort 4) 144
4.3.6 FLOT (Cohort 5) 145
4.4 Blinding 145
4.5 Assignment and Allocation 145
5 TREATMENTS AND EVALUATION 145
5.1 Dosing and Administration of Study Drug(s) and Other Medication(s) 145
5.1.1 Dose/Dose Regimen and Administration Period 145

5.1.1.1 Zolbetuximab 145
5.1.1.2 Antiemetic Premedication 146
5.1.1.3 mFOLFOX6 (Cohorts 2, 4A and 4B Only) 147
5.1.1.4 Pembrolizumab (Cohort 3A Only) 147
5.1.1.5 Nivolumab (Cohorts 4A and 4B Only) 148
5.1.1.6 FLOT (Cohort 5 Only) 148
5.1.2 Treatment Delays or Discontinuation 148
5.1.2.1 Zolbetuximab Delay 148
5.1.2.2 mFOLFOX6 Delay (Cohorts 2 and 4) 149
5.1.2.3 Pembrolizumab Delay (Cohort 3) 149
5.1.2.4 Nivolumab Delay (Cohort 4) 150
5.1.2.5 FLOT Delay (Cohort 5) 150
5.1.2.6 Discontinuation of mFOLFOX6 Components 150
5.1.2.7 Continuation of mFOLFOX6 (or Some Components, with or without Nivolumab) if Zolbetuximab is Discontinued First 150
5.1.2.8 Continuation of Zolbetuximab if mFOLFOX6 (with or without Nivolumab) or Pembrolizumab is Discontinued First 150
5.1.2.9 Continuation of Pembrolizumab if Zolbetuximab is Discontinued First 151
5.1.2.10 Continuation of Nivolumab if Zolbetuximab is Discontinued First 151
5.1.2.11 Discontinuation of Both Zolbetuximab and mFOLFOX6 (All Components, with or without Nivolumab) or Pembrolizumab 151
5.1.2.12 Continuation of FLOT (or Some Components) if Zolbetuximab is Discontinued First 151
5.1.3 Increase or Reduction in Dose of the Study Drug 151
5.1.3.1 Zolbetuximab 152
5.1.3.2 mFOLFOX6 (Cohorts 2 and 4) 155
5.1.3.3 Oxaliplatin-Induced Neurotoxicity 158
5.1.3.4 Dose Modifications for Pembrolizumab 160
5.1.3.5 Dose Modifications for Nivolumab 161
5.1.3.6 Dose Modifications for FLOT 163
5.1.3.7 Dose Modifications for FLOT Due to Hematologic Toxicity 163
5.1.3.8 Dose Modifications for FLOT Due to Non-hematologic Toxicity 165
5.1.3.9 Dose Adjustment for FLOT in Case of Oxaliplatin-related Neurotoxicity 166
5.1.3.10 Dose Adjustments for FLOT in Case of Other Toxicities 166
5.1.4 Previous and Concomitant Treatment (Medication and Non-medication Therapy) 166

5.1.5 Treatment Compliance 168
5.1.6 Criteria for Continuation of Treatment 168
5.2 Demographics and Baseline Characteristics 168
5.2.1 Demographics 168
5.2.2 Medical History 168
5.2.3 Diagnosis of the Target Disease, Severity and Duration of Disease 168
5.3 Efficacy Assessments 168
5.4 Safety Assessment 170
5.4.1 Vital Signs 170
5.4.2 Adverse Events 170
5.4.2.1 Adverse Events of Possible Hepatic Origin 170
5.4.3 Laboratory Assessments 170
5.4.4 Physical Examination 172
5.4.5 Electrocardiogram 172
5.5 Adverse Events and Other Safety Aspects 173
5.5.1 Definition of Adverse Events 173
5.5.1.1 Abnormal Laboratory Findings 173
5.5.1.2 Potential Cases of Drug-induced Liver Injury 174
5.5.1.3 Disease Progression and Study Endpoints 174
5.5.2 Definition of Serious Adverse Events 174
5.5.3 Criteria for Causal Relationship to Study Drug 175
5.5.4 Criteria for Defining the Severity of an Adverse Event 176
5.5.5 Reporting of Serious Adverse Events 177
5.5.6 Follow-up of Adverse Events 179
5.5.7 Monitoring of Common Serious Adverse Events 179
5.5.8 Adverse Events of Special Interest 179
5.5.8.1 Guidelines for Infusion-related Reactions 179
5.5.9 Special Situations 180
5.5.9.1 Pregnancy 180
5.5.9.2 Medication Error, Overdose and “Off-label Use” 181
5.5.9.3 Misuse/Abuse 181
5.5.9.4 Occupational Exposure 182
5.5.9.5 (Suspicion of) Transmission of Infectious Agent 182
5.5.9.6 Suspected Drug-Drug Interaction 182
5.5.10 Supply of New Information Affecting the Conduct of the Study 182

5.5.11 Urgent Safety Measures 183
5.5.12 Reporting Urgent Safety Measures 183
5.6 Test Drug Concentration 183
5.7 Other Measurements, Assessments or Methods 184
5.7.1 Biomarkers 184
5.7.2 Blood/Serum/Plasma Samples/PBMCs for Exploratory Biomarkers 184
5.7.3 Tumor Tissue Samples 184
5.7.4 Immunogenicity Assessment 188
5.7.5 Optional Samples for Future Pharmacogenomics Analysis 188
5.7.6 Quality of Life and Patient Reported Outcomes 189
5.7.6.1 Quality of Life Questionnaire 189
5.7.6.2 Oesophago-gastric Module 189
5.7.6.3 Global Pain 189
5.7.6.4 EuroQOL Five Dimensions Questionnaire 189
5.7.6.5 Health Resource Utilization 189
5.8 Total Amount of Blood 189
6 DISCONTINUATION 190
6.1 Discontinuation of Individual Subject(s) 190
6.2 Discontinuation of the Site 191
6.3 Discontinuation of the Study 191
7 STATISTICAL METHODOLOGY 192
7.1 Sample Size 192
7.2 Analysis Sets 193
7.2.1 Full Analysis Set 193
7.2.2 Safety Analysis Set 193
7.2.3 Pharmacokinetic Analysis Set 193
7.2.4 Biomarker Analysis Set 193
7.2.5 DLT Evaluation Analysis Set 193
7.3 Demographics and Baseline Characteristics 193
7.3.1 Subject Disposition 193
7.3.2 Previous and Concomitant Medications 194
7.3.3 Medical History 194
7.4 Analysis of Efficacy 194
7.4.1 Analysis of Primary Endpoint 194

7.4.2 Analysis of Secondary Endpoints 194
7.4.2.1 Disease Control Rate 194
7.4.2.2 Duration of Response 194
7.4.2.3 Clinical Response and Pathological Response 194
7.4.2.4 Progression-free Survival 195
7.4.2.5 Disease-free Survival 195
7.4.2.6 Overall Survival 195
7.4.2.7 HRQoL 195
7.4.2.8 Minimal Residual Disease 195
7.4.3 Analysis of Exploratory Endpoints 195
7.4.3.1 Biomarkers 195
7.4.4 Imaging 196
7.5 Analysis of Safety 196
7.5.1 Adverse Events 196
7.5.2 Laboratory Assessments 196
7.5.3 Vital Signs 196
7.5.4 Physical Examination 196
7.5.5 Routine 12-lead Electrocardiograms 196
7.5.6 Other Safety Aspects 197
7.6 Analysis of Pharmacokinetics 197
7.6.1 Concentrations 197
7.6.2 Estimation of Pharmacokinetic Parameters 198
7.6.3 Statistical Analysis of Pharmacokinetic Parameters 198
7.6.4 Immunogenicity 198
7.7 Major Protocol Deviations 198
7.8 Interim Analysis (and Early Discontinuation of the Clinical Study) 198
7.9 Handling of Missing Data, Outliers, Visit Windows and Other Information 198
8 OPERATIONAL AND ADMINISTRATIVE CONSIDERATIONS 199
8.1 Procedure for Clinical Study Quality Control 199
8.1.1 Data Collection 199
8.1.1.1 Collection of Data Via Electronic Source and SAS Transfer 199
8.1.1.2 Electronic Patient Reported Outcome 200
8.1.1.3 Data Quality Assurance 200
8.1.2 Specification of Source Documents 200
8.1.3 Clinical Study Monitoring 202

8.1.4 Direct Access to Source Data/Documents 202
8.1.5 Data Management 202
8.1.6 Major Protocol Deviations 202
8.1.7 Start and End of Trial in All Participating Countries 203
8.2 Ethics and Protection of Subject Confidentiality 204
8.2.1 Institutional Review Board/Independent Ethics Committee/Competent Authorities 204
8.2.2 Ethical Conduct of the Study 204
8.2.3 Informed Consent of Subjects 204
8.2.3.1 Subject Information and Consent 204
8.2.3.2 Supply of New and Important Information Influencing the Subject’s Consent and Revision of the Written Information 206
8.2.4 Data Protection 206
8.3 Administrative Matters 208
8.3.1 Arrangement for Use of Information and Publication of the Clinical Study 208
8.3.2 Documents and Records Related to the Clinical Study 208
8.3.3 Protocol Amendment and/or Revision 209
8.3.4 Insurance of Subjects and Others (ex-US sites only) 210
8.3.5 Dissemination of Clinical Study Data 211
8.3.5.1 Disclosure of Study Information and Results 211
8.3.5.2 Access to Study Data 212
9 QUALITY ASSURANCE 212
10 STUDY ORGANIZATION 212
10.1 Independent Data-Monitoring Committee/Data and Safety Monitoring Board 212
10.2 Other Study Organization 212
11 REFERENCES 213
12 APPENDICES 216
12.1 List of Excluded Concomitant Medications 216
12.2 Liver Safety Monitoring and Assessment 217
12.3 Common Serious Adverse Events 220
12.4 Eastern Cooperative Oncology Group (ECOG) Performance Status 221
12.5 Retrospective PGx Sub-Study (Optional) 222
12.6 Contraception Requirements 224
12.7 Clinical Study Continuity 226

13 COORDINATING INVESTIGATOR'S SIGNATURE ···· 268

List of In-text Tables

Table 1 Schedule of Assessments for Cohort 1A ···· 65
Table 2 Cohort 1A – Zolbetuximab Pharmacokinetic, Immunogenicity and Triplicate Electrocardiogram Schedule ···· 71
Table 3 Schedule of Assessments for Cohort 2 Cycle 1 ···· 73
Table 4 Schedule of Assessments for Cohort 2 Cycles ≥ 2 ···· 79
Table 5 Cohort 2 – Zolbetuximab Pharmacokinetic and Immunogenicity Schedule ···· 84
Table 6 Cohort 2 – mFOLFOX6 Pharmacokinetic Schedule ···· 85
Table 7 Schedule of Assessments for Cohort 3A ···· 86
Table 8 Cohort 3A– Zolbetuximab and Pembrolizumab Pharmacokinetic/Immunogenicity Schedule ···· 93
Table 9 Schedule of Assessments for Cohort 4 ···· 94
Table 10 Cohorts 4A and 4B – Zolbetuximab and Nivolumab Pharmacokinetic/Immunogenicity Schedule ···· 104
Table 11 Schedule of Assessments for Cohort 5 ···· 105
Table 12 Cohort 5 – Zolbetuximab Pharmacokinetic/Immunogenicity Schedule ···· 111
Table 13 Cohort 5 – Exploratory Biomarkers Collection Schedule ···· 112
Table 14 Recommended Action Based on the Number of Subjects for DLT Assessment ···· 126
Table 15 Infusion Rates Recommended for Each Zolbetuximab Infusion ···· 146
Table 16 Infusion-related Reactions ···· 152
Table 17 Guidelines for Zolbetuximab Treatment Modification Due to Non-hematologic Toxicity ···· 153
Table 18 Zolbetuximab: Dose Modifications for Hematologic Toxicity ···· 154
Table 19 Recommended Dose Adjustment Levels for Oxaliplatin and 5-FU ···· 155
Table 20 mFOLFOX6 Dose Modification Due to Hematologic Toxicity ···· 156
Table 21 mFOLFOX6 Dose Modification Due to Non-hematologic Toxicity ···· 158
Table 22 Oxaliplatin Dose Modification for Associated Neurotoxicity ···· 159
Table 23 Comparison of the Symptoms and Treatment of Laryngopharyngeal and Platinum Hypersensitivity Reactions ···· 160
Table 24 Recommended Dose Modifications for Adverse Reactions – Pembrolizumab ···· 161
Table 25 Recommended Dose Modifications – Nivolumab ···· 162
Table 26 Recommended Dose Modifications – FLOT ···· 163
Table 27 Recommended Dose Modifications for FLOT Due to Hematologic Toxicity ···· 163
Table 28 Recommended Dose Modifications for FLOT Due to Non-hematologic Toxicity ···· 165
Table 29 Recommended Dose Adjustment for FLOT in Case of Oxaliplatin-related Neurotoxicity ···· 166
Table 30 Clinical Laboratory Tests ···· 171
Table 31 Tumor Tissue Requirements Cohort 1 ···· 185
Table 32 Tumor Tissue Requirements Cohort 2 ···· 186
Table 33 Tumor Tissue Requirements Cohort 3 ···· 186

Table 34 Tumor Tissue Requirements Cohort 4 187
Table 35 Tumor Tissue Requirements Cohort 5 188
Table 36 Alternative Schedule of Assessments in Response to a Crisis, Cohort 2 Cycle ≥ 2 228
Table 37 Alternative Schedule of Assessments in Response to a Crisis, Cohort 4 243
Table 38 Alternative Schedule of Assessments in Response to a Crisis, Cohort 5 259

List of In-text Figures

Figure 1 Flow Chart for Cohort 1A 48
Figure 2 Cohort 1A Treatment Period Dosing Schedule 49
Figure 3 Study Drug Dosing Schematic (Cohort 1A) 50
Figure 4 Flow Chart for Cohort 2 51
Figure 5 Cohort 2 Treatment Period Dosing Schedules 52
Figure 6 Study Drug Dosing Schematics (Cohort 2) 54
Figure 7 Flow Chart for Cohort 3A 56
Figure 8 Cohort 3 Treatment Period Dosing Schedules 57
Figure 9 Flow Chart for Cohort 4 58
Figure 10 Cohort 4 Treatment Period Dosing Schedules 59
Figure 11 Flow Chart for Cohort 5 62
Figure 12 Cohort 5 Treatment Period Dosing Schedules 63

I. SIGNATURES

1. SPONSOR'S SIGNATURES

Required sponsor signatures as required by ICH GCP 4.5.1 are located in the first attachment.

**Attachment 1**	Electronic Sponsor Signatures

2. INVESTIGATOR'S SIGNATURE

A Phase 2 Study of Zolbetuximab (IMAB362) as Monotherapy and in Combination with Chemotherapy and/or Immunotherapy in Subjects with Metastatic or Locally Advanced Unresectable Gastric or Gastroesophageal Junction (GEJ) Adenocarcinoma and Locoregional Gastric or GEJ Adenocarcinoma Whose Tumors are Claudin (CLDN) 18.2-Positive

ISN/Protocol 8951-CL-0103

Version 8.0 Incorporating Substantial Amendment 7

23 May 2024

I have read all pages of this clinical study protocol for which Astellas is the sponsor. I agree to conduct the study as outlined in the protocol and to comply with all the terms and conditions set out therein. I confirm that I will conduct the study in accordance with International Council for Harmonisation (ICH) Good Clinical Practice (GCP) guidelines and applicable local regulations. I will also ensure that subinvestigator(s) and other relevant members of my staff have access to copies of this protocol and the ICH GCP guidelines to enable them to work in accordance with the provisions of these documents.
**Principal Investigator**:<br>Signature: --<br>Date (DD Mmm YYYY)<br><br>Printed Name: --<br>*<Insert name and qualification of the investigator>*<br><br>Address: --<br>--

II. CONTACT DETAILS OF KEY SPONSOR'S PERSONNEL

**24h-Contact for Serious Adverse Events (SAEs)**<br><br>See [Section 5.5.5 Reporting of Serious Adverse Events] for SAE Fax Number and Email	**Please fax or email the SAE Worksheet to:**<br>**Astellas Pharma Global Development, Inc.**<br>**Pharmacovigilance**<br>**Fax number North America: 888-396-3750**<br>**North America Alternate Fax: 847-317-1241**<br>**International Fax Number: +44-800-471-5263**<br>**Email: safety-us@astellas.com**<br><br>**Japan site only:**<br>**Please fax or email the SAE Worksheet to:**<br>**Astellas Pharma Inc. Japan**<br>**Pharmacovigilance**<br>**Fax number: +81-(0)3-3243-5747**<br>**Email: rk-safety-jp@astellas.com**
Medical Monitor/Study Physician:	*PPD*<br>Astellas Pharma Global Development Inc.<br>Northbrook, Illinois 60062<br>*PPD*<br><br>PAREXEL International<br>Herman Heijermansweg 20<br>1077 WL Amsterdam, Netherlands<br>*PPD*
Clinical Research Contact:	*PPD*
Clinical Research Contact Japan:	Corporate Name: Astellas Pharma Inc.<br>Location: 2-5-1, Nihonbashi-Honcho, Chuo-ku, Tokyo<br>Office phone: 03-3244-1097<br>Fax: 03-3243-5737<br>Sponsor's personnel: *PPD*<br>Contact numbers during non-business hours and for emergency:<br>*PPD*

III. LIST OF ABBREVIATIONS AND DEFINITION OF KEY TERMS

List of Abbreviations

Abbreviations	Description of abbreviations
5-FU	fluorouracil
ADA	anti-drug antibody
ADCC	antibody-dependent cellular cytotoxicity
AE	adverse event
ALP	alkaline phosphatase
ALT	alanine aminotransferase (GPT)
ANC	absolute neutrophil count
APGD	Astellas Pharma Global Development Inc.
AST	aspartate aminotransferase (GOT)
AUC	area under the concentration-time curve
AUC_{inf}	AUC from the time of dosing extrapolated to time infinity
AUC_{inf} (%extrap)	percentage of AUC_{inf} due to extrapolation from the last measurable concentration to time infinity
AUC_{last}	AUC from the time of dosing up to the time of the last measurable concentration
AUC_{tau}	auc from the time of dosing to the start of the next dosing interval at multiple dose conditions
AxMP	auxiliary medicinal product
βHCG	beta human chorionic gonadotropin (HCG)
BMAS	biomarker analysis set
BSA	body surface area
C_{max}	maximum concentration
C_{trough}	concentration immediately prior to dosing at multiple dosing
C1D1	cycle 1 day 1
CDC	complement-dependent cytotoxicity
CIOMS	council for international organizations of medical sciences
CL	total clearance after intravenous dosing
CLDN	claudin
CPS	combined positive score
CR	complete response
CRO	contract research organization
CT	computerized tomography
CTCAE	Common Terminology Criteria for Adverse Events
ctDNA	circulating tumor DNA
CV	coefficient of variation

Abbreviations	Description of abbreviations
DCR	disease control rate
DEAS	DLT Evaluation Analysis Set
DFS	disease-free survival
DILI	drug-induced liver injury
DLT	dose-limiting toxicity
DOR	duration of response
DPD	dihydropyrimidine dehydrogenase
ECG	electrocardiogram
ECF/ECX	epirubicin and cisplatin plus fluorouracil or capecitabine
ECOG	Eastern Cooperative Oncology Group
eCRF	electronic case report form
EEA	European Economic Area
EORTC	European Organisation for Research and Treatment of Cancer
EOT	end of treatment
EOX	epirubicin, oxaliplatin and capecitabine
EQ-5D	EuroQOL five dimensions questionnaire
EUS	endoscopic ultrasound
FAS	full analysis set
FFPE	formalin-fixed paraffin-embedded
FIM	first-in-human
FLOT	fluorouracil, leucovorin or folinic acid, oxaliplatin and docetaxel
GCP	Good Clinical Practice
GEJ	gastroesophageal junction
GMP	Good Manufacturing Practice
GP	global pain
HBsAg	hepatitis B surface antigen
HCG	human chorionic gonadotropin
HCV	hepatitis C
Hgb	hemoglobin
HER2	human epidermal growth factor receptor 2
HIV	human immunodeficiency virus
HR	hazard ratio
HRQoL	health-related quality of life
HRU	health resource utilization
HSR	hypersensitivity reactions
IB	Investigator's Brochure

Abbreviations	Description of abbreviations
ICF	informed consent form
ICH	International Council for Harmonisation of Technical Requirements for Pharmaceuticals for Human Use
IEC	independent ethics committee
IHC	immunohistochemistry
IMAB	ideal monoclonal antibody
IMP	investigational medicinal product
INR	international normalized ratio
IRB	institutional review board
IRR	infusion related reaction
IRT	interactive response technology
ISN	international study number
ITT	intent to treat
IUD	intrauterine device
IUS	intrauterine system
IV	intravenous
LA-CRF	liver abnormality case report form
LFT	liver function tests
mFOLFOX6	5-fluorouracil, leucovorin or folinic acid and oxaliplatin
MRI	magnetic resonance imaging
NCCN	National Comprehensive Cancer Network
NCI	National Cancer Institute
NCI-CTCAE	National Cancer Institute Common Terminology Criteria for Adverse Events
nIMP	noninvestigational medicinal product
NSAID	nonsteroidal anti-inflammatory drugs
OG-25	Oesophago-Gastric Module (EORTC QLQ-OG-25)
ORR	objective response rate
OS	overall survival
PBMC	peripheral blood mononuclear cells
PD	progressive disease
PD-L1	programmed death-ligand 1
PFS	progression-free survival
PGx	pharmacogenomics
PKAS	pharmacokinetic analysis set
PR	partial response
PRES	posterior reversible encephalopathy syndrome
PT	prothrombin time

**Abbreviations**	**Description of abbreviations**
PTT	partial thromboplastin time
QLQ-C30	Quality of Life Questionnaire - Core Questionnaire
QTc	corrected QT
QTcF	Fridericia-corrected QT interval
RSI	reference safety information
SAE	serious adverse event
SAF	safety analysis set
SAP	statistical analysis plan
SOC	system organ class
SOP	standard operating procedure
SPC	summary of product characteristics
SUSAR	suspected unexpected serious adverse reactions
$t_{1/2}$	terminal elimination half-life
t_{max}	time of maximum concentration
TBL	total bilirubin
TEAE	treatment-emergent adverse event
TEM	tolerability evaluation meeting
TNM	tumor/node/metastasis
ULN	upper limit of normal
USM	urgent safety measure
V_z	volume of distribution after intravenous dosing during the terminal elimination phase
VEGF	vascular endothelial growth factor
WFI	water for injection
WOCBP	woman of child-bearing potential

Definition of Key Study Terms

**Terms**	**Definition of terms**
Baseline	Assessments of subjects as they enter a trial before they receive any treatment.
Endpoint	Variable that pertains to the efficacy or safety evaluations of a trial.
Enroll	To register or enter a subject into a clinical trial. NOTE: Once a subject has received the study drug or placebo, the clinical trial protocol applies to the subject.
Intervention	The drug, device, therapy or process under investigation in a clinical study that is believed to have an effect on outcomes of interest in a study. (e.g., health-related quality of life, efficacy, safety and pharmacoeconomics).
Investigational period	Period of time where major interests of protocol objectives are observed, and where the test drug or comparative drug (sometimes without randomization) is usually given to a subject, and continues until the last assessment after completing administration of the test drug or comparative drug.
Post investigational period	Period of time after the last assessment of the protocol. Follow-up observations for sustained adverse events and/or survival are done in this period.
Screening	A process of active consideration of potential subjects for enrollment in a trial.
Screen failure	Potential subject who did not meet 1 or more criteria required for participation in a trial.
Screening period	Period of time before entering the investigational period, usually from the time when a subject signs the consent until just before the test drug or comparative drug is given to a subject.
Study period	Period of time from the first site initiation date to the last site completing the study.
Variable	Any entity that varies; any attribute, phenomenon or event that can have different qualitative or quantitative values.

IV. PROTOCOL AMENDMENT SUMMARY OF CHANGES

**DOCUMENT HISTORY**	
**Document**	**Date**
Amendment 7	23 May 2024
Amendment 6	02 Aug 2023
Amendment 5	22 Aug 2022
Amendment [Nonsubstantial] 1	21 Oct 2021
Amendment 4	25 Feb 2021
Amendment 3	10 Mar 2020
Amendment 2	03 Jul 2018
Amendment 1	08 Feb 2018
Original Protocol	13 Dec 2017

Amendment 7 [Substantial] 23 May 2024

This amendment is considered to be substantial based on the criteria set forth in Regulation (EU) No 536/2014 of the European Parliament.

Overall Rationale for the Amendment:

The primary rationale for this amendment is to allow competitive enrollment and remove the enrollment cap initially set by region and to clarify and specify that disease progression should be confirmed radiographically to distinguish from and avoid confusion with clinical progression. Updates are made to maintain consistency within the protocol and with phase 3 study protocols and to align with the current EU CTR requirements.

Summary of Changes

Substantial Changes

Section Number	Description of Change	Brief Rationale
V, 2.2.1, 3.1	The requirement of the minimum enrollment of at least 50% of the subjects in Cohort 4B from the US or Europe is removed.	To allow competitive enrollment and remove the enrollment cap initially set by region. This will help more patients who are eligible to participate to have access to study drugs and receive treatment on study.

Nonsubstantial Changes

Section Number	Description of Change	Brief Rationale
Title Page	Short title and EU clinical trial number are added as protocol identifiers; EudraCT number is removed.	To align with current EU CTR requirements.
II	Clinical Research Contact: Clinical Study Manager updated to Clinical Trial Lead.	To align with change of designation.
IV	The text was updated to reflect that "this amendment is considered to be substantial based on the criteria set forth in Regulation (EU) No 536/2014 of the European Parliament".	To add new EU regulation, whose criteria are the basis for the current amendment.
V	Short title is added to the synopsis.	To align with current EU CTR requirements.
V and 2.2.1	Text is updated to reflect a reduction in the number of sites in North America and Japan for Cohort 5 from 13 to 5 sites.	This is a very small cohort to assess safety. Limiting to small number of sites can be more effective and reduce burden to sites.
V, VI (Figures 9, 11 and Tables 9, 11), 2.2.1, 3.2, 5.1.2.7, 5.1.2.8, 5.1.2.10, 5.1.2.11, 5.1.2.12, 5.3, 5.7.3 (Tables 34, 35), 6.1, 12.7 (Tables 37, 38)	Text is updated to add 'radiographic' or delete 'or clinical' to disease progression and progressive disease (PD).	To specify that progression of disease should be confirmed by radiographic assessment. This is to add clarification as well as distinguish from and avoid confusion with clinical progression. If a subject discontinues treatment due to clinical progression, then they need to enter post-treatment period and continue radiographic assessment of disease until radiographic progression of disease is confirmed. However, this change is not implemented for Cohorts 1, 2 and 3A, where recruitment is complete.
V, 2.2.1	The text describing exploratory biomarker collection is updated to add 'cycle 1 day 1, 14-30 days after last preoperative dose'.	To align the exploratory biomarker sample collection timepoints with those in Table 13.
V and 4.1	'Investigational product(s)' was updated to 'investigational medicinal product(s) (IMPs)'.	To keep terminology consistent throughout the protocol.

Section Number	Description of Change	Brief Rationale
V, 4.1.2, 4.1.2.1, 4.1.2.2, 4.1.2.3, and 4.1.2.4	Details regarding marketing status of approved products used in the study are added.<br>Text is added to characterize mFOLFOX6 and FLOT used in this clinical trial as auxiliary medicinal products (AxMP)/noninvestigational medicinal products (nIMP). Additionally, text is updated to characterize nivolumab and pembrolizumab as 'AxMP/nIMP' instead of 'IMP'.	To align with current EU CTR requirements.
V and 5.1.1.2	Text is added to state 'Antiemetics are authorized products and will be used in this study according to the marketing authorizations'.	To align with current EU CTR requirements.
4.2 and 4.3.1	Language for the final release of medication is updated.	To align with current EU CTR requirements.
V, 5.1.1.3 and 5.1.1.6	For levofolinate mode of administration specific to Japan, 'or longer' is removed from the phrase '2 hours or longer' and the text 'or per institutional standard of care' is added.	Removal of 'or longer' is based on approved dosage and administration in Japan. Addition of 'or per institutional standard of care' is for consistency within the protocol.
5.1.2	Section title updated to 'Treatment Delays or Discontinuation'.	To clarify the content of the section.
VI (Figure 12)	The dose of folinic acid for Cohort 5 is changed from 400 mg/m^2 to 200 mg/m^2.	To align the folinic acid dose for Cohort 5 with that in sections V and 5.1.1.6.
VI (Table 9)	The text was added to footnote 34 to reflect that survival follow-up will also be performed following the start of another anti-cancer therapy.	To maintain consistenty throughout the document.
VI (Tables 1, 3, 4, 7, 9 11) and 12.7 (Tables 36, 37, 38)	The text 'or up to 48 hours prior to treatment' is added for HRQoL and HRU assessments.	To align the assessment timing with that in phase 3 study protocols.
VI (Table 11), 5.3, 12.7 (Table 38)	For Cohort 5 image assessment, text is revised to state 'Imaging at restaging (after preoperative chemotherapy prior to surgery) and after a subject discontinues or completes all study treatment (every 3 [± 1] months during the first year, then every 4 [± 2] months during the second year in post-treatment follow-up period'.	To provide clarity in the protocol text.

Section Number	Description of Change	Brief Rationale
VI (Table 11)	12-lead ECG assessments are added to the Schedule of Assessments for Cohort 5 on Day 2 for cycles 1 and 5 which is clarified in footnote 20. The footnote is also updated to include the exact timepoints of single ECG and to clarify that cycles 2-4 and 6-8 ECG are to be performed on FLOT administration days.	To clarify the ECG timepoints and to align with Section 5.1.1.6.
5.1.2.1, 5.1.3.1 (Table 17)	Information that one case of PRES was reported in subjects receiving zolbetuximab, and instruction to discontinue zolbetuximab if PRES is suspected, are added. PRES diagnosis should be confirmed by brain imaging, preferably by MRI.	To add instructions on discontinuation of zolbetuximab in case PRES is suspected.
5.1.3.3	Information that cases of PRES have been reported in subjects receiving oxaliplatin combination chemotherapy, and instruction to discontinue oxaliplatin if PRES is suspected, were added. PRES diagnosis should be confirmed by brain imaging, preferably by MRI.	To add instructions on discontinuation of oxaliplatin in case PRES is suspected.
5.1.3 (Table 27)	Added the following footnote to Table 27:<br>† At the investigator's discretion, growth factors may be used according to standard practice guidelines.	To clarify that in case of febrile neutropenia investigators may use growth factors at their discretion.
5.4.5	ECG section is updated to add the phrase '(Cohort 5 only: and preoperative follow-up visits [7-14 days and 30 days after last preoperative dose] and postoperative follow-up visits [EOT and 30-day follow-up])'.	To align with the ECG assessments reflected in Table 11.
6.1	A bullet "Subject declines further study participation" is added to the list of criteria for subject discontinuation from the treatment period.	To align with section V.
8.1.1	The sentence "The monitor should verify the data in the eCRFs with source documents and confirm that there are no inconsistencies between them" is removed.	The requirement is removed as it is no longer relevant.

Section Number	Description of Change	Brief Rationale
8.1.3	Text is added to state that clinical study monitoring at EU sites should be as per EU CTR regulations.	To align with current EU CTR requirements.
8.1.7	Text is added to define 'first act of recruitment' and 'completion of study for a participant'.	To align with current EU CTR requirements.
8.2.3.1	Following sentence "Subject or his/her guardian or legal representative will be required to sign a statement of informed consent that meets the requirements of 21 CFR 50, EU CTR 536/2014 (including Article 29), local regulations, ICH GCP guidelines, the Declaration of Helsinki, HIPAA requirements, where applicable, and the IRB/IEC or study center'' is added.	To align with current EU CTR requirements.
8.2.4	Section title is updated from 'Subject Confidentiality and Privacy' to 'Data Protection'.<br>Text is added on use of personal data of participants throughout the development program.<br>Text is added to specify that non-medical personnel acting on behalf of Astellas may be given access to recorded data to verify that the study is carried out in compliance with legal and quality requirements.	To align with current EU CTR requirements and to specify access of recorded data provided to non-medical personnel acting on behalf of Astellas.
8.3.2	Language for record retention is updated.	To align with current EU CTR requirements.
8.3.5	Section title is updated from 'Signatory Investigator for Clinical Study Report' to 'Dissemination of Clinical Study Data;' language is updated. Additional information for disclosure of study information and results and access to study data is added in two newly added subsections.	To align with current EU CTR requirements.
Throughout	Minor administrative-type changes, e.g., typos, format, numbering, consistency throughout the protocol.	To provide clarifications to the protocol and to ensure complete understanding of study procedures.

V. SYNOPSIS

**Date and Version No. of Protocol Synopsis:**	23 May 2024, Version 8.0
**Sponsor:**<br>Astellas Pharma Global Development Inc. (APGD)	**Protocol Number:**<br>8951-CL-0103
**Name of Study Drug:**<br>Zolbetuximab (IMAB362)	**Phase of Development:**<br>2

Title of Study:
A Phase 2 Study of Zolbetuximab (IMAB362) as Monotherapy and in Combination with Chemotherapy and/or Immunotherapy in Subjects with Metastatic or Locally Advanced Unresectable Gastric or Gastroesophageal Junction (GEJ) Adenocarcinoma and Locoregional Gastric or GEJ Adenocarcinoma Whose Tumors are Claudin (CLDN) 18.2-Positive

Short Title: A Study of Zolbetuximab (IMAB362) in Adults with Gastric Cancer

Planned Study Period:
From 2Q2018 to 1Q2026

Study Objective(s):

Primary

- To determine the objective response rate (ORR) of zolbetuximab as a single agent as assessed by an independent central reader

Secondary

- To evaluate pharmacokinetics of zolbetuximab as a single agent, in combination with 5-fluorouracil (5-FU), leucovorin or folinic acid and oxaliplatin (mFOLFOX6), in combination with pembrolizumab, in combination with mFOLFOX6 and nivolumab, and in combination with fluorouracil, leucovorin or folinic acid, oxaliplatin and docetaxel (FLOT)
- To evaluate pharmacokinetics of oxaliplatin and 5-FU in combination with zolbetuximab
- To assess the safety and tolerability of zolbetuximab as a single agent, in combination with mFOLFOX6 (with or without nivolumab), in combination with pembrolizumab, and in combination with FLOT
- To assess the immunogenicity of zolbetuximab as a single agent, in combination with mFOLFOX6 (with or without nivolumab), in combination with pembrolizumab, and in combination with FLOT
- To evaluate health-related quality of life (HRQoL)
- To assess ORR of zolbetuximab in combination with pembrolizumab and in combination with mFOLFOX6 as assessed by an independent central reader
- To assess ORR of zolbetuximab as a single agent, in combination with mFOLFOX6 (with or without nivolumab) based on investigator assessment
- To evaluate disease control rate (DCR), duration of response (DOR) and progression-free survival (PFS) of zolbetuximab as a single agent and in combination with mFOLFOX6 based on independent central reader assessment
- To evaluate DCR, DOR and PFS of zolbetuximab as a single agent and in combination with mFOLFOX6 (with or without nivolumab) based on investigator assessment
- To assess overall survival (OS) of zolbetuximab as a single agent, in combination with mFOLFOX6 and nivolumab, and in combination with FLOT
- Cohort 5: To evaluate antitumor activity of zolbetuximab and FLOT as measured by radiological response (restaging) and pathological response (ypTNM)

- Cohort 5: To assess disease-free survival (DFS) of zolbetuximab in combination with FLOT
- Cohort 5: To assess minimal residual disease and disease recurrence

Exploratory

- To evaluate potential genomic and/or other biomarkers that may correlate with treatment outcome of zolbetuximab as a single agent, in combination with mFOLFOX6 (with or without nivolumab), and in combination with FLOT
- To assess the effects of zolbetuximab as a single agent and in combination with mFOLFOX6 (with or without nivolumab) on Claudin (CLDN) 18.2 expression
- To assess the immunomodulatory effects of zolbetuximab as a single agent, in combination with mFOLFOX6 (with or without nivolumab), and in combination with FLOT
- To evaluate the pharmacokinetics of pembrolizumab in combination with zolbetuximab
- To evaluate the pharmacokinetics of nivolumab in combination with zolbetuximab and mFOLFOX6.

Planned Total Number of Study Centers and Location(s):

Cohorts 1-4: Up to 25 centers in North America, Europe and Asia Pacific

Cohort 5: Up to 5 centers in North America and Japan

Study Population:

Subjects with metastatic or locally advanced unresectable gastric or gastroesophageal junction (GEJ) adenocarcinoma and locoregional gastric or GEJ adenocarcinoma whose tumors are CLDN18.2-positive by central immunohistochemistry (IHC) testing, defined as follows:

- High CLDN18.2 expression has ≥ 75% of tumor cells demonstrating moderate to strong membranous staining as determined by central IHC testing.
- Intermediate CLDN18.2 expression has ≥ 50%, but < 75% of tumor cells demonstrating moderate to strong membranous staining as determined by central IHC testing.

Subjects in Cohorts 2 and 4 must be human epidermal growth factor receptor 2 (HER2) negative based on local or central testing results.

Number of Subjects to be Enrolled:

Approximately 143 subjects will be enrolled into the study as outlined in the table below:

	Treatment	No. of Subjects	Line of Treatment	CLDN18.2 Expression	HER2 Status
**Cohort 1A****	Zolbetuximab	30 enrolled*	≥ 3rd Line	High	Not Applicable
**Cohort 2****	Zolbetuximab + mFOLFOX6	21 enrolled	1st Line	High	HER2 Negative
**Cohort 3A****	Zolbetuximab + Pembrolizumab	3 enrolled	≥ 3rd Line	High or Intermediate	Not Applicable
**Cohort 4A****	Zolbetuximab + mFOLFOX6 + Nivolumab	12 enrolled	1st Line	High or Intermediate	HER2 Negative
**Cohort 4B**	Zolbetuximab + mFOLFOX6 + Nivolumab	Approximately 65	1st Line	High or Intermediate	HER2 Negative
**Cohort 5**	Zolbetuximab + FLOT	Approximately 12	Perioperative	High	Not Applicable

CLDN18.2: claudin 18.2; HER2: human epidermal growth factor receptor 2

*Subjects with measurable disease at screening based on RECIST v 1.1 per independent central reader.

**Closed to enrollment.

At least 50% of the subjects enrolled in Cohorts 1A and 2 must be from the US or Europe to ensure the study is regionally balanced.

Study Design Overview:

This is a phase 2, open-label, multi-arm, non-randomized, multicenter study to assess the antitumor activity of zolbetuximab, an IgG1 chimeric monoclonal antibody directed against CLDN18.2, in subjects with metastatic or locally advanced unresectable gastric or GEJ adenocarcinoma and locoregional gastric or GEJ adenocarcinoma whose tumors are CLDN18.2-positive. For each cohort, the study consists of the following periods: pre-screening, screening, treatment and follow-up for radiographic disease progression (or post-treatment follow-up for disease recurrence, which will be conducted for Cohort 5). In addition, there will be a survival follow-up period for Cohorts 1A, 4B and 5 subjects only.

Pre-Screening:

After the pre-screening informed consent has been obtained, an archival tumor sample will be submitted for each subject to determine CLDN18.2 status (in addition to HER2 status for Cohorts 2 and 4 if required). If available, the most recent tumor sample is preferred. If archival tumor tissue is insufficient or unavailable for pre-screening, the subject will forego the pre-screening period and directly enter the screening period and biopsy will be performed to obtain the tumor sample for eligibility needed to determine CLDN18.2 status and HER2 status as required. While not required for eligibility, programmed death-ligand 1 (PD-L1) status will be determined based on the tumor sample submitted.

Screening and Enrollment:

Enrollment for Cohorts 1A, 2, 3A and 4A has been completed. Enrollment for Cohorts 4B and 5 is ongoing. After the screening main informed consent has been obtained, screening will take place up to 28 days prior to subject enrollment on cycle 1 day 1. Subjects may be rescreened once.

Treatment Period:

Cohort 1A:

Subjects in Cohort 1A will be treated with zolbetuximab on a 21-day cycle in which zolbetuximab will be administered as a single agent every 3 weeks until disease progression, toxicity requiring cessation, start of another anti-cancer treatment or other treatment discontinuation criteria are met. Cohort 1A will enroll approximately 20 subjects with measurable disease at baseline. Imaging in Cohort 1A will be performed every 6 weeks from cycle 1 day 1 for the first 24 weeks and then every 12 weeks thereafter.

Cohort 2:

Subjects in Cohort 2 will be treated with zolbetuximab and mFOLFOX6 on a 42-day cycle in which zolbetuximab is administered on days 1 and 22, and mFOLFOX6 is administered on days 1, 15 and 29; however, for the first cycle, zolbetuximab will be administered on day 3 (instead of day 1) to allow for pharmacokinetic collection. Subjects will receive up to 12 mFOLFOX6 treatments (4 cycles). Beginning at cycle 5, subjects may continue on 5-FU and leucovorin or folinic acid along with zolbetuximab for the remainder of the study per the investigator’s discretion. Imaging in Cohort 2 will be performed every 9 weeks for the first 54 weeks, and then every 12 weeks thereafter.

Cohort 3A:

Subjects in Cohort 3A will be treated with zolbetuximab and pembrolizumab on a 21-day cycle in which zolbetuximab and pembrolizumab are administered on day 1 of each cycle. This cohort is to determine safety and tolerability. Imaging in Cohort 3A will be performed every 6 weeks for the first 24 weeks and then every 12 weeks thereafter.

The tolerability and safety of zolbetuximab in combination with pembrolizumab will be evaluated during the 3-week dose-limiting toxicity (DLT) assessment period. Initially, 3 subjects will be

enrolled into Cohort 3A and receive the zolbetuximab loading dose of 800 mg/m^2 in combination with pembrolizumab 200 mg on cycle 1 day 1, followed by zolbetuximab 600 mg/m^2 in combination with pembrolizumab 200 mg on day 1 of each subsequent cycle. Any of the zolbetuximab and/or pembrolizumab related adverse events (AEs) specified as the DLTs will be assessed.

<u>Cohort 4:</u>

In Cohort 4A, the tolerability and safety of zolbetuximab in combination with mFOLFOX6 and nivolumab will be evaluated during the 2-week DLT assessment period. Initially, 3 subjects will be enrolled into Cohort 4A and receive the zolbetuximab loading dose of 800 mg/m^2 in combination with nivolumab 240 mg and mFOLFOX6 on cycle 1 day 1, followed by zolbetuximab 400 mg/m^2 in combination with nivolumab 240 mg and mFOLFOX6 every 2 weeks [days 15 and 29] (1 cycle = 6 weeks). Zolbetuximab will be administered first, followed by the nivolumab and then mFOLFOX6. Any of the zolbetuximab, mFOLFOX6 and/or nivolumab related AEs specified as the DLTs will be assessed. Subjects will receive up to 12 mFOLFOX6 treatments (4 cycles).

In Cohort 4B, subjects will be treated with the combination of zolbetuximab, mFOLFOX6 and nivolumab at the dose deemed tolerable in Cohort 4A. Subjects will receive up to 12 mFOLFOX6 treatments (4 cycles).

For Cohorts 4A and 4B, beginning at cycle 5, subjects may continue on 5-FU and leucovorin or folinic acid along with zolbetuximab and nivolumab for the remainder of the study per investigator's discretion. Imaging in Cohort 4A and 4B will be performed every 8 weeks for the first 56 weeks, and then every 12 weeks thereafter.

<u>Cohort 5:</u>

Subjects in Cohort 5 will be treated with zolbetuximab in combination with FLOT for a total of eight 2-week cycles. Four cycles will be administered preoperatively and 4 cycles will be administered postoperatively. The tolerability and safety of treatment with zolbetuximab in combination with FLOT will be evaluated for DLTs during the 2-week DLT assessment period following the first preoperative treatment with zolbetuximab. Any of the zolbetuximab and/or FLOT related AEs considered DLTs will be assessed.

Preoperative Treatment

Subjects will be enrolled into Cohort 5 and receive the zolbetuximab loading dose of 800 mg/m^2 on cycle 1 day 1, followed by FLOT on cycle 1 day 2. For cycles 2-4, subjects may receive zolbetuximab 400 mg/m^2 in combination with FLOT, dosed on day 1 of each cycle. When dosing both treatments on the same day, zolbetuximab should be administered first, followed by FLOT. However, dosing may continue to be split over 2 days with zolbetuximab administration on day 1 and FLOT administration on day 2, based on investigator judgment. Subjects should undergo surgery within 4-8 weeks after completion of 4 cycles of preoperative zolbetuximab + FLOT.

If the zolbetuximab 800 mg/m^2 loading dose is assessed as <u>not</u> tolerable based on the DLT assessment, then 3 to 6 subjects will be treated with zolbetuximab 600 mg/m^2 loading dose on cycle 1 day 1 in combination with FLOT on cycle 1 day 2, followed by zolbetuximab 400 mg/m^2 in combination with FLOT on day 1 (or zolbetuximab on day 1 with FLOT on day 2) of cycles 2-4 (2-week cycles).

Postoperative Treatment

Subjects who have undergone complete surgical resection (R0) should receive 4 cycles of postoperative treatment with zolbetuximab + FLOT, starting 6-12 weeks after surgery.

Subjects will receive the zolbetuximab loading dose of 800 mg/m^2 on cycle 5 day 1, followed by FLOT on cycle 5 day 2. For subjects who experience a DLT during preoperative treatment on the loading dose of 800 mg/m^2, the postoperative loading dose may be reduced to 600 mg/m^2 based on the judgment of the investigator. However, for subjects who experience a DLT during preoperative

treatment on a loading dose of 600 mg/m^2, the postoperative loading dose may not be omitted, but dose reduction/modification can be considered for FLOT based on investigator judgment.<br>For cycles 6-8, subjects may receive zolbetuximab 400 mg/m^2 in combination with FLOT dosed on day 1 of each cycle. When dosing both treatments on the same day, zolbetuximab will be administered first, followed by FLOT. However, dosing may continue to be split over 2 days with zolbetuximab administration on day 1 and FLOT administration on day 2, based on investigator judgment.<br>Tolerability evaluation meeting: Cohorts 3A, 4A and 5<br>The tolerability evaluation meeting (TEM) will be held after the end of the 3-week (Cohort 3A) or 2-week (Cohorts 4A and 5) DLT assessment period for the first 3 or 6 total DLT-evaluable subjects enrolled in a cohort. The sponsor will comprehensively assess the data (including AEs reported for subjects who are unevaluable for DLTs) generated from the cohort to be assessed and discuss the tolerability of the current dose with the investigators at the TEM. Based on the results of the discussion, the sponsor will decide the tolerability of the current dose level.
For Cohort 3A, it has been determined that zolbetuximab 800/600 mg/m^2 in combination with pembrolizumab 200 mg is tolerable.<br>For Cohort 4A, it has been determined that zolbetuximab 800/400 mg/m^2 in combination with mFOLFOX6 and nivolumab is tolerable.<br>For Cohort 5, the starting dose of the preoperative combination therapy is zolbetuximab 800 mg/m^2 loading dose on cycle 1 day 1 in combination with FLOT on cycle 1 day 2, followed by zolbetuximab 400 mg/m^2 in combination with FLOT on day 1 (or zolbetuximab on day 1 with FLOT on day 2) of cycles 2-4 (2-week cycles).<br>• If zolbetuximab 800 mg/m^2 loading dose is assessed as tolerable based on the DLT assessment, then Cohort 5 may proceed to complete enrollment.<br>• If zolbetuximab 800 mg/m^2 loading dose is assessed as not tolerable based on the DLT assessment, then 3 to 6 subjects will be treated with zolbetuximab 600 mg/m^2 loading dose on cycle 1 day 1 in combination with FLOT on cycle 1 day 2, followed by zolbetuximab 400 mg/m^2 in combination with FLOT on day 1 (or zolbetuximab on day 1 with FLOT on day 2) of cycles 2-4 (2-week cycles).<br>• If zolbetuximab 600 mg/m^2 loading dose, based on the DLT assessment, is assessed as:<br>A) Tolerable, then:<br>o Cohort 5 may proceed to complete enrollment, OR<br>o Re-escalation to 800 mg/m^2 loading dose may occur based on the Bayesian Optimal Interval Design [Liu & Yuan, 2015] criteria shown in the Dose Limiting Toxicities section below.<br>B) Not tolerable, then:<br>o Cohort 5 will cease enrollment.<br>In following the recommendations by the Bayesian Optimal Interval Design, the sponsor may choose a more conservative pathway based on safety evaluations conducted regularly, as well as the totality of safety data available. Based on these, the sponsor may decide to further assess safety on a lower loading dose level and enroll additional subjects.<br>Dose Limiting Toxicities:<br>Quantitative assessment of DLTs will be performed according to the criteria shown in the table below referring to the concept of Bayesian Optimal Interval Design [Liu & Yuan, 2015] with the target DLT rate of 33% with the maximum number of subjects limited to 6 in each dose level.

- 0 of 3 initial evaluable subjects experience DLTs: If this is not the highest available dose level, 3 subjects may be enrolled at the next available higher dose level. If this is the highest available dose level, this dose may be considered tolerable based on the totality of the data.
- 1 of 3 initial evaluable subjects experience DLTs: Additional 3 evaluable subjects will be enrolled at the current dose level and the assessment is continued.
    - Less than 2 of the 6 evaluable subjects experience DLTs: If this is not the highest available dose level, 3 subjects may be enrolled at the next available higher dose level. If this is the highest available dose level, this dose may be considered tolerable based on the totality of the data.
    - 2 of 6 evaluable subjects experience DLTs: The tolerability of this dose level will be determined based on a comprehensive assessment of the incidence of DLTs and other safety data at the TEM (the totality of the data).
    - 3 of 6 evaluable subjects experience DLTs: If this is not the lowest available dose level, 3 subjects will be enrolled at next available lower dose level. If this is the lowest available dose level, then this dose will not be considered tolerable.
    - 4 or more of 6 evaluable subjects experience DLTs: If this is not the lowest available dose level, 3 subjects will be enrolled at the next available lower dose level, and the current dose level will be eliminated from the available dose levels. If this is the lowest available dose level, this dose will not be considered tolerable.
- 2 of 3 initial evaluable subjects experience DLTs: If this is not the lowest available dose level, 3 subjects will be enrolled at the next available lower dose level and this dose level may be considered available for re-escalation. If this is the lowest available dose level, this dose will not be considered tolerable.
- 3 of 3 initial evaluable subjects experience DLTs: If this is not the lowest available dose level, 3 subjects will be enrolled at the next lower dose level and the current dose level will be eliminated from the available dose levels. If this is the lowest available dose level, this dose will not be considered tolerable.

Recommended Action Based on the Number of Subjects for DLT Assessment

	**Number of Subjects for DLT Assessment**			
Recommended Action by Bayesian Criteria				
Number of Subjects for DLT Assessment	3	4*	5*	6
If the number of subjects with DLTs is equal to the number in the cell to the right or less:<br>1) If this is not the highest available dose level, 3 subjects may be enrolled at next available higher dose level.<br>2) If this is the highest available dose level, this dose level is considered tolerable.	0	1	1	1
If the number of subjects with DLTs is equal to the number in the cell to the right and:<br>1) If the current dose level has reached 3 subjects, enroll additional 3 subjects at this dose level.<br>2) If the current dose level has reached 6 subjects, the tolerability of this dose level will be determined based on the totality of the data.	1	-	-	2
If the number of subjects with DLTs is equal to the number in the cell to the right and:<br>1) If this is not the lowest available dose level, enroll 3 subjects at the next available lower dose level. This dose level may be considered available for re-escalation.<br>2) If this is the lowest available dose level, this dose level will not be considered tolerable.	2	2	2 or 3	3

If the number of subjects with DLTs is the number in the cell to the right or more:<br>1) If this is not the lowest available dose level, enroll 3 subjects at the next available lower dose level, and this dose will be eliminated from the available dose levels for re-escalation.<br>2) If this is the lowest available dose level, this dose level will not be considered tolerable.	3	3	4	4

DLT: dose-limiting toxicity

* In case the number of subjects for DLT assessment is 4 or 5, it is considered exceptional, including, for example, if informed consent is obtained from more than 3 subjects in the process of enrolling 3 subjects in the 1st dose level. In such a case, these subjects will be handled as subjects for DLT assessment in the 1st cohort by deciding that the planned cohort size has been modified from 3 to 4 or 5 subjects.

A DLT is defined as any of the following AEs (graded using National Cancer Institute Common Terminology Criteria for Adverse Events [NCI-CTCAE] version 4.03) or laboratory findings that the investigator (or sponsor) cannot clearly attribute to a cause other than study drugs (zolbetuximab, mFOLFOX6, pembrolizumab, nivolumab and FLOT) occurring during the 3-week (Cohort 3A) or 2-week (Cohorts 4A and 5) DLT assessment period:

- Grade 4 neutropenia (afebrile)
- Grade ≥ 3 febrile neutropenia
- Grade 4 thrombocytopenia
- Grade 3 thrombocytopenia accompanied by bleeding that requires any transfusion (platelets or red blood cells)
- Grade 4 anemia
- Grade 3 anemia requiring red blood cell transfusion
- Grade ≥ 3 non-hematologic AE

  (NOTE: Grade 3 nausea, vomiting, diarrhea, fatigue or abdominal pain that improves to ≤ grade 2 with appropriate treatment within 7 days will not be assessed as a DLT)
- Grade ≥ 2 pneumonitis
- Grade ≥ 2 encephalopathy or meningitis
- Guillain-Barré syndrome or myasthenic syndrome/myasthenia gravis
- Aspartate aminotransferase (AST) or alanine aminotransferase (ALT) > 5 × upper limit of normal (ULN; grade ≥ 3) in subjects without liver metastases
- AST or ALT > 8 × ULN in subjects with liver metastases
- AST or ALT > 3 × ULN and total bilirubin > 2 × ULN (in subject with Gilbert syndrome: AST or ALT > 3 × ULN and direct bilirubin > 1.5 × ULN) in subjects without liver metastases
- Total bilirubin > 3 × ULN (grade ≥ 3)
- Amylase or lipase > 2 × ULN (grade ≥ 3)
- Grade ≥ 3 infusion-related reaction (other than nausea, vomiting or abdominal pain) including hypersensitivity reactions and reactions with features of anaphylaxis
- Grade 5 toxicity

Replacement of Subjects:

In Cohorts 3A, 4A and 5, subjects who meet the following criterion during the 3-week or 2-week DLT assessment period, respectively, will be considered unevaluable for DLT and will be replaced by another subject:

- For Cohorts 3A and 4A, a subject without a DLT who receives less than the planned dose on cycle 1 day 1, or does not complete cycle 1 evaluation for a reason other than DLT (e.g., consent withdrawal)
- For Cohort 5, a subject without a DLT who receives less than the planned dose of zolbetuximab on cycle 1 day 1 and/or of FLOT by cycle 1 day 2, or does not complete cycle 1 evaluation for a reason other than DLT (e.g., consent withdrawal)

Tolerability Evaluation for Japanese Subjects:

Cohort 3: Tolerability of zolbetuximab in combination with pembrolizumab in Japanese subject(s) will be evaluated in the Cohort 3A DLT assessment. Quantitative assessment of any tolerability issues observed in the 3-week assessment period will be performed according to the criteria referring to the Bayesian Optimal Interval Design with the target rate of 0.33.

Cohort 4: Tolerability of zolbetuximab in combination with mFOLFOX6 and nivolumab in Japanese subject(s) will be evaluated in Cohort 4B, if Japanese subjects are not enrolled in the Cohort 4A DLT assessment. Tolerability and safety will be evaluated during a 2-week assessment period for one Japanese subject enrolled in Cohort 4B (if no DLT is observed in Cohort 4A) or 3 Japanese subjects (if 1 or more DLTs are observed in Cohort 4A). The Japanese subject(s) will be assessed for tolerability in Cohort 4B, using the same DLT criteria as those used for the Cohort 4A DLT assessment, but the subject(s) will not be part of the formal DLT assessment already completed in Cohort 4A. Quantitative assessment of any tolerability issues observed in the 2-week assessment period will be performed according to the criteria referring to the Bayesian Optimal Interval Design with the target rate of 0.33. Additional Japanese subjects may be enrolled in Cohort 4B, by up to 6 evaluable Japanese subjects, to continue the tolerability assessment. The details of tolerability evaluation procedure in Cohort 4B will be documented in the Japan Tolerability Guidelines.

Cohort 5: If Japanese subjects are enrolled and assigned to Cohort 5, a separate Japanese tolerability evaluation at the same dose level will not occur because the tolerability of Japanese subjects will have been evaluated, as well as non-Japanese subjects.

Cohorts 1-4 Discontinuation:

Following discontinuation from zolbetuximab, subjects will have a study drug discontinuation visit, and safety follow-up visits 30 days and 90 days after their last dose of zolbetuximab. For subjects in Cohort 2, following mFOLFOX6 (all components) discontinuation, Cohort 3 following pembrolizumab discontinuation or Cohort 4 following discontinuation of mFOLFOX6 (all components) and/or nivolumab, subjects will have a study discontinuation visit and safety follow-up visits 30 and 90 days after their last dose of mFOLFOX6, pembrolizumab or nivolumab. If mFOLFOX6, pembrolizumab, or nivolumab are discontinued at the same time as zolbetuximab, 1 discontinuation visit for both zolbetuximab and mFOLFOX6 or zolbetuximab and pembrolizumab or zolbetuximab and nivolumab may be performed.

If a subject discontinues mFOLFOX6 (or components of mFOLFOX6), pembrolizumab or nivolumab due to any reason other than radiographic progressive disease (PD) as confirmed by the independent central reader (or investigator assessment for Cohort 4), they may continue on zolbetuximab at the discretion of the investigator provided that all of the following have been met:

- The subject completed at least 1 cycle (42 days [3 treatments]) of mFOLFOX6 (with or without nivolumab) or 2 cycles of pembrolizumab (42 days) treatment;
- The subject will not receive or has not received other chemotherapy;
- And in the investigator's opinion the subject continues to derive clinical benefit with acceptable toxicity.

Subjects should continue to follow the Study Treatment Period schedule of assessments.

Cohort 5 Discontinuation:

Zolbetuximab and FLOT will be discontinued after completion of the 4 planned postoperative cycles, with an end of treatment visit taking place up to 7 days after treatment discontinuation. Additionally, subjects will have safety follow-up visits at 30 and 90 days after completion of postoperative treatment or after early discontinuation, if applicable. If zolbetuximab and FLOT are discontinued separately, the safety follow-up visits should take place relative to each treatment discontinuation.

Post-Treatment Follow-up Period for Radiographic Disease Progression (Cohorts 1A, 2, and 4 only)

If a subject discontinues all study treatment prior to radiographic disease progression, the subject will enter the post-treatment follow-up period and continue to undergo imaging assessments until radiographic disease progression or until the subject starts another anti-cancer treatment, whichever occurs first.

Post-Treatment Follow-up Period for Disease Recurrence (Cohort 5 only)

If a subject discontinues or completes all study treatment, the subject will enter the post-treatment follow-up period for up to 2 years. Subjects will undergo imaging assessments every 3 months (± 1 month) during the first year, and then every 4 months (± 2 months) or according to local guidelines/standard clinical practice during the second year until disease recurrence (or radiographic disease progression) or until the subject starts another anticancer treatment, whichever happens first. Subjects will also have exploratory biomarker samples collected at cycle 1 day 1, 14-30 days after last preoperative dose, cycle 5 day 1 and cycle 8 day 1, postoperative 30-day and 90-day follow-up, and at 6, 9, 12, 16, 20 and 24 months from the date of surgery.

<u>*Survival Follow-up Period (Cohorts 1A and 4B only)*</u>

Following radiographic disease progression or the start of another anti-cancer therapy (whichever occurs first), subjects in Cohorts 1A and 4B will enter the survival follow-up period and be followed every 12 weeks. Survival follow-up will be performed via telephone.

<u>*Survival Follow-up Period (Cohort 5 only)*</u>

Following completion of the post-treatment follow-up period, disease recurrence (or radiographic disease progression) or the start of another anticancer therapy (whichever occurs first), subjects in Cohort 5 will enter the survival follow-up period for the remainder of the 3-year period following last dose of study treatment. Survival follow-up will be performed via telephone every 3 months.

<u>*Imaging Assessment*</u>

All radiologically evaluable disease (measurable and/or non-measurable) must be documented at screening and re-assessed at each subsequent tumor evaluation. Computerized tomography (CT) scans of the chest, abdomen, and pelvis, or other areas as clinically indicated (brain, bone, etc.), should be performed using contrast media unless clinically contraindicated. If CT scan is medically not feasible with contrast, magnetic resonance imaging (MRI) may be used for imaging. Bone scans (or focal X-ray) or brain imaging should be performed if metastatic disease is suspected. Disease must be evident by radiology, measurable lesions only. If the sole lesion lies within the field of prior radiotherapy, there must be evidence of radiographic disease progression prior to inclusion in the study. Imaging for Cohorts 1A, 2 and 3A will be sent to an independent central reader within 7 days for the assessment of PFS and ORR based on RECIST 1.1. Central radiographic review assessment will be conveyed to the investigator if the central reader determines that the subject has met RECIST 1.1 defined radiographic progression. Imaging for Cohorts 4 and 5 should also be sent to the independent central reader within 7 days. The central radiographic review for these cohorts will occur at the end of study or at a time point to be specified by the sponsor.

Imaging for Restaging

For subjects in Cohort 5, restaging will be conducted after completion of 4 cycles of preoperative treatment and prior to surgery. Radiological restaging may include imaging, endoscopy and/or laparoscopy.

<u>*Safety Assessments*</u>

Safety will be evaluated based on AEs, electrocardiogram (ECG), vital signs, Eastern Cooperative Oncology Group (ECOG) performance status and laboratory assessments based on NCI-CTCAE version 4.03. Pharmacokinetic-matched, centrally read ECG measurements (Cohorts 1A and 2 only) and locally read ECG measurements (Cohorts 3A, 4 and 5) will be collected per the Schedule of Assessments to evaluate the potential of zolbetuximab to induce QT/corrected QT (QTc) interval prolongation.

Additional safety assessments for Cohort 5 will include surgical complications, surgical mortality defined as death within 30 days of surgery, percentage of subjects able to complete preoperative chemotherapy, perioperative mortality and morbidity at 30 days and 90 days post last dose, percentage of subjects able to start postoperative chemotherapy and percentage of subjects able to complete postoperative chemotherapy.

<u>*Biomarkers*</u>

In order to assess the effects of any study drug treatment on CLDN18.2 expression, immune activity and other biomarkers in the tumor microenvironment, tumor specimens may be analyzed for biomarkers. Tumor sample collection is summarized below.

Tumor Tissue Specimens (FFPE) to be sent to Central Laboratory

	Tumor Sample for Eligibility	Baseline Tumor Sample	On-Treatment Biopsy	Post-Progression Biopsy**
	Archival or newly obtained* to determine eligibility	Recent (≤ 3 months prior to start of study drug) for biomarker analyses	For biomarker analyses	For biomarker analyses
**Cohort 1A**	Required	Required	Required	Optional
**Cohort 2**	Required	Required	Required	Optional
**Cohort 3A**	Required	Optional	Optional	Optional
**Cohort 4A**	Required	Optional	Optional	Optional
**Cohort 4B**	Required	Required	Required	Optional

	Tumor Sample for Eligibility	Postoperative (Surgical) Tumor Sample	Post-Progression Biopsy**
	Archival or newly obtained* to determine eligibility	For biomarker analyses	For biomarker analyses
**Cohort 5**	Required	Required	Optional

FFPE: formalin-fixed paraffin-embedded

* If the subject does not have archival tissue to determine eligibility, the subject must enter the screening period and undergo a biopsy to obtain the tumor sample needed to determine CLDN18.2 status and human epidermal growth factor receptor 2 status as required.

**An optional post-progression tumor tissue sample may be collected for subjects who sign a separate informed consent form.

Additionally, serum, plasma and whole blood samples will be collected for analysis of biomarkers that may be related to treatment outcome. An optional blood sample for future pharmacogenomics (PGx) analysis may be collected for subjects who sign a separate informed consent form (ICF).

HRQoL

HRQoL questionnaires and Health Resource Utilization (HRU) questionnaires will be assessed during the visit prior to study treatment or physician assessment at screening, every 3 weeks (Cohorts 1A, 2 and 3A) and every 2 weeks (Cohorts 4 and 5) during the treatment period, at the 30-day safety follow-up visit and the 90-day follow-up visit. HRQoL will be measured by Quality of Life Questionnaire – Core Questionnaire (European Organisation for Research and Treatment of Cancer [EORTC] QLQ-C30), Oesophago-gastric Module (EORTC OG-25), Global Pain (GP), EuroQOL five dimensions questionnaire (EQ-5D) and HRU questionnaires.

Primary Analysis

The primary analysis will occur once all subjects have progressed, died, discontinued from the study or provided at least 1 post-baseline disease assessment, whichever occurs first. Primary endpoint, secondary endpoints and other endpoints will be analyzed at the time of the primary analysis.

Inclusion/Exclusion Criteria:

Inclusion:

Waivers to the inclusion criteria will **NOT** be allowed. Laboratory results obtained during screening should be used to determine eligibility criteria. In situations where laboratory results are outside of the permitted range, the investigator may opt to retest the subject and subsequent within-range screening results may be used to confirm eligibility. CT scans and MRI conducted as part of a subject's routine clinical management (i.e., standard of care) obtained before signing the ICF may be utilized for screening or baseline purposes, provided the procedures met the protocol-specified criteria and were performed within the Screening period.

Subject is eligible for the study if all of the following apply:

1. Institutional Review Board (IRB)/Independent Ethics Committee (IEC) approved written informed consent and privacy language as per national regulations (e.g., Health Insurance Portability and Accountability Act [HIPAA] Authorization for US sites) must be obtained from the subject or legally authorized representative prior to any study-related procedures (including withdrawal of prohibited medication, if applicable).
2. Subject is considered an adult according to local regulation at the time of signing informed consent.
3. Female subject eligible to participate if she is not pregnant [See Appendix 12.6 Contraception Requirements] and at least one of the following conditions applies:
    a. Not a woman of child-bearing potential (WOCBP) as defined in Appendix 12.6 Contraception Requirements
    OR
    b. WOCBP who agrees to follow the contraceptive guidance as defined in Appendix 12.6 Contraception Requirements throughout the treatment period and for at least 9 months after the final oxaliplatin administration and 6 months after the final administration of all other study drugs
4. Female subject must agree not to breastfeed starting at screening and throughout the study period, and for 6 months after the final study drug administration.
5. Female subject must agree not to donate ova starting at screening and throughout the study period, and for 9 months after the final oxaliplatin administration and 6 months after the final administration of all other study drugs.
6. A sexually active male subject with a female partner(s) who is of child-bearing potential must agree to use contraception as detailed in Appendix 12.6 Contraception Requirements during the treatment period and for at least 6 months after the final study drug administration.
7. Male subject must agree not to donate sperm starting at screening and throughout the study period, and for 6 months after the final study drug administration.
8. Male subject with a pregnant or breastfeeding partner(s) must agree to remain abstinent or use a condom for the duration of the pregnancy or time partner is breastfeeding throughout the study period and for 6 months after the final study drug administration.
9. Subject has histologically confirmed gastric or GEJ adenocarcinoma.
10. Cohorts 1-4: Subject has radiographically confirmed, locally advanced, unresectable or metastatic disease within 28 days prior to the first dose of study treatment.
11. Subject's tumor is positive for CLDN18.2 expression demonstrating moderate to strong membranous staining as determined by central IHC testing.
12. Subject agrees not to participate in another interventional study while on treatment.
13. Subject has ECOG performance status 0 to 1.
14. Subject has predicted life expectancy ≥ 12 weeks in the opinion of the investigator.
15. Subject must meet all of the following criteria based on the centrally or locally analyzed laboratory tests collected within 14 days prior to the first dose of study treatment. In case of multiple central laboratory data within this period, the most recent data should be used.
    - Hemoglobin (Hgb) ≥ 9 g/dL (transfusion is allowed, but post-transfusion Hgb [24 hours or later following transfusion] must be ≥ 9 g/dL)
    - Absolute neutrophil count (ANC) ≥ 1.5 × 10^9/L
    - Platelets ≥ 100 × 10^9/L
    - Albumin ≥ 2.5 g/dL
    - Total bilirubin ≤ 1.5 × upper limit of normal (ULN)

- Aspartate aminotransferase (AST) and alanine aminotransferase (ALT) ≤ 2.5 × ULN in subjects without liver metastases (≤ 5 × ULN if liver metastases are present)
- Cohorts 1-4: Estimated creatinine clearance ≥ 30 mL/min
- Cohort 5: Serum creatinine ≤ 1.5 × ULN, or estimated creatinine clearance ≥ 50 mL/min for subjects with serum creatinine levels > 1.5 × ULN
- Prothrombin time/international normalized ratio and partial thromboplastin time ≤ 1.5 × ULN (except for subjects receiving anticoagulation therapy)

Specific to Cohort 1A:

1. Subject has measurable disease according to RECIST 1.1 within 28 days prior to the first dose of study treatment per investigator assessment. For subjects with only 1 evaluable lesion and prior radiotherapy ≤ 3 months before enrollment, the lesion must either be outside the field of prior radiotherapy or must have documented progression following radiation therapy.
2. Subject has disease progression on or after at least 2 prior regimens for their advanced disease, including fluoropyrimidine and platinum-containing chemotherapy, and if appropriate, HER2/neu-targeted therapy and all associated side effects have resolved to grade 1 or less.
3. Subject must have an additional available tumor specimen collected within 3 months prior to the first dose of study treatment.
4. Subject must be an appropriate candidate for a tumor biopsy and is amenable to undergo a tumor biopsy during the screening period (if applicable) and treatment period as indicated in the Schedule of Assessments.

Specific to Cohort 2:

1. Subject has measurable disease according to RECIST 1.1 within 28 days prior to the first dose of study treatment per investigator assessment. For subjects with only 1 evaluable lesion and prior radiotherapy ≤ 3 months before enrollment, the lesion must either be outside the field of prior radiotherapy or must have documented progression following radiation therapy.
2. Subject has not received prior systemic anti-cancer therapy for their advanced disease (subject may have received neoadjuvant and/or fluorouracil-containing adjuvant chemotherapy as long as it has been completed ≥ 6 months before the first dose of study treatment).
3. Subject has a gastric or GEJ tumor that is HER2-negative as determined by local or central testing.
4. Subject must have an additional available tumor specimen collected within 3 months prior to the first dose of study treatment.
5. Subject must be an appropriate candidate for a tumor biopsy and is amenable to undergo a tumor biopsy during the screening period (if applicable) and treatment period as indicated in the Schedule of Assessments.

Specific to Cohort 3A:

1. Subject has radiologically evaluable disease (measurable and/or non-measurable) according to RECIST 1.1, per local assessment, ≤ 28 days prior to the first dose of study treatment. For subjects with only 1 evaluable lesion and prior radiotherapy ≤ 3 months before enrollment, the lesion must either be outside the field of prior radiotherapy or must have documented progression following radiation therapy.
2. Subject has disease progression on or after at least 2 prior regimens for their advanced disease, including fluoropyrimidine and platinum-containing chemotherapy, and if appropriate, HER2/neu-targeted therapy.
3. Subject has not received prior checkpoint inhibitor therapy.

<u>Specific to Cohorts 4A and 4B:</u>

1. Subject has radiologically evaluable disease (measurable and/or non-measurable) according to RECIST 1.1, per local assessment, ≤ 28 days prior to the first dose of study treatment. For subjects with only 1 evaluable lesion and prior radiotherapy ≤ 3 months before enrollment, the lesion must either be outside the field of prior radiotherapy or must have documented radiographic progression following radiation therapy.
2. Subject has not received prior systemic anti-cancer therapy for their advanced disease (subject may have received neoadjuvant and/or fluorouracil-containing adjuvant chemotherapy as long as it has been completed ≥ 6 months before the first dose of study treatment).
3. Subject has a gastric or GEJ tumor that is HER2-negative as determined by local or central testing.
4. Subject has not received prior checkpoint inhibitor therapy.

<u>Specific to Cohort 4B Only:</u>

1. Subject must have an additional available tumor specimen collected within 3 months prior to the first dose of study treatment.
2. Subject must be an appropriate candidate for a tumor biopsy and is amenable to undergo a tumor biopsy during the screening period (if applicable) and treatment period as indicated in the Schedule of Assessments.

<u>Specific to Cohort 5 Only:</u>

1. Subject has new histologically confirmed primary gastric or GEJ adenocarcinoma that are amenable to curative resection.
2. Subject has locoregional, resectable gastric or GEJ adenocarcinoma. GEJ may include type I-III Siewert classification. Clinical stage will be determined by endoscopic ultrasound (EUS) and/or CT or MRI. Diagnostic laparoscopy may be used as per institutional guidelines and clinical practices.
3. Subject meets one of the following criteria of locoregional disease by clinical TNM staging:
   a. GEJ: cT2,N0 (high-risk lesions: ≥3 cm, poorly differentiated), cT1b–cT2,N+ or cT3–cT4a,Any N.
   b. Gastric: T2 to T4a, and/or N1-3,M0.
4. Subject's tumor expresses CLDN18.2 in ≥ 75% of tumor cells demonstrating moderate to strong membranous staining as determined by central IHC testing.

Exclusion:

Waivers to the exclusion criteria will **NOT** be allowed.

Subject who meets any of the following exclusion criteria prior to enrollment is not eligible for enrollment:

1. Subject has had prior severe allergic reaction or intolerance to known ingredients of zolbetuximab or other monoclonal antibodies, including humanized or chimeric antibodies.
2. Subject has known immediate or delayed hypersensitivity or contraindication to any component of study treatment.
3. Subject has received other investigational agents or devices concurrently or within 28 days prior to first dose of study treatment.
4. Subject has received systemic immunosuppressive therapy, including systemic corticosteroids 14 days prior to first dose of study treatment. Subjects using a physiologic replacement dose of hydrocortisone or its equivalent (defined as up to 30 mg per day of hydrocortisone or up to 10 mg per day of prednisone), receiving a single dose of systemic corticosteroids or receiving systemic corticosteroids as pre-medication for radiologic imaging contrast use are allowed.

5. Subject has a complete gastric outlet syndrome or a partial gastric outlet syndrome with persistent recurrent vomiting.
6. Per investigator judgment, subject has significant gastric bleeding and/or untreated gastric ulcers that would preclude the subject from participation per investigator judgment.
7. Subject has history of central nervous system metastases and/or carcinomatous meningitis from gastric/GEJ cancer.
8. Subject has a known history of a positive test for human immunodeficiency virus (HIV) infection or known active hepatitis B (positive hepatitis B surface antigen [HBsAg]) or hepatitis C infection. NOTE: Screening for these infections should be conducted per local requirements.
   - For subjects who are negative for HBs Ag, but HBc Ab positive, an HB DNA test will be performed and if positive the subject will be excluded.
   - Subjects with positive hepatitis C virus (HCV) serology, but negative HCV RNA test results are eligible.
   - Subjects treated for HCV with undetectable viral load results are eligible.
9. Subject has had within 6 months prior to first dose of study treatment any of the following: unstable angina, myocardial infarction, ventricular arrhythmia requiring intervention or hospitalization for heart failure.
10. Subject has active infection requiring systemic therapy that has not completely resolved within 7 days prior to the start of study treatment.
11. Subject has active autoimmune disease that has required systemic treatment within the past 3 months prior to the start of study treatment.
12. Subject has a clinically significant disease or co-morbidity that in the opinion of the investigator may adversely affect the safe delivery of treatment within this study or make the subject unsuitable for study participation.
13. Subject has psychiatric illness or social situations that would preclude study compliance per investigator's judgment.
14. Subject has had a major surgical procedure ≤ 28 days before start of study treatment.
15. Subject is without complete recovery from a major surgical procedure ≤ 14 days before start of study treatment.
16. Subject has received radiotherapy for locally advanced unresectable or metastatic gastric or GEJ adenocarcinoma ≤ 14 days (Cohorts 1 and 3A) and ≤ 28 days (Cohorts 2 and 4A or 4B) prior to start of study treatment and has NOT recovered from any related toxicity.
17. Subject has another malignancy for which treatment is required, per investigator's clinical judgment.

18. Cohorts 2, 4 and 5 Only, subject has any of the following:
    - Prior severe allergic reaction or intolerance to any component of mFOLFOX6 or FLOT chemotherapeutics in this study.
    - Known dihydropyrimidine dehydrogenase (DPD) deficiency (screening for DPD deficiency should be conducted per local requirements).
    - Known peripheral neuropathy > grade 1 (absence of deep tendon reflexes as the sole neurological abnormality does not render the subject ineligible).
    - Sinusoidal obstruction syndrome, formerly known as veno-occlusive disease, if present, should be stable or improving per investigator's judgment.
    - History of clinically significant ventricular arrhythmias (i.e., sustained ventricular tachycardia, ventricular fibrillation, or Torsades de Pointes).
    - QTc interval > 450 msec for male subjects; QTc interval > 470 msec for female subjects;
    - History or family history of congenital long QT syndrome.
    - Cardiac arrhythmias requiring anti-arrhythmic medications (Subjects with rate controlled atrial fibrillation for > 1 month prior to first dose of study treatment are eligible).
19. Cohorts 3A, 4A and 4B Only, subject has any of the following:
    - Subject with ongoing or previous autoimmune disease or interstitial lung disease, active diverticulitis or gastrointestinal ulcerative disease, or solid organ or stem cell transplant (for Cohort 4), or other uncontrolled or clinically significant medical disorders.
    - Subject with type 1 diabetes mellitus, endocrinopathies stably maintained on appropriate replacement therapy or skin disorders (e.g., vitiligo, psoriasis, or alopecia) not requiring systemic treatment are allowed.
    - Subject has known history of serious hypersensitivity reaction to a known ingredient of pembrolizumab or nivolumab.
    - Cohort 4B Only: Subject with known microsatellite instability-high or mismatch repair deficient tumors.
20. Cohort 5 Only, subject has either of the following:
    - Subject cannot undergo curative resection per the investigator's judgment
    - Subject meets the following criterion of locoregional disease by clinical TNM staging: cT1N0.

**Investigational Medicinal Product:**<br>**Zolbetuximab**<br>The investigational medicinal product (IMP), zolbetuximab, is a sterile lyophilized powder for solution for infusion. Chimeric monoclonal antibody zolbetuximab is the active pharmaceutical ingredient. Each vial contains 105 mg of zolbetuximab and has to be reconstituted with 5.0 mL water for injection per vial to a concentration of 20 mg/mL. Further dilution with sterile 0.9% sodium chloride to a final concentration of 2 mg/mL is required.<br>**Other Drugs:**<br>mFOLFOX6, pembrolizumab, nivolumab, and FLOT are authorized products and will be used in this study according to the marketing authorizations.<br>**mFOLFOX6**<br>mFOLFOX6 components are auxiliary medicinal products (AxMP)/noninvestigational medicinal products (nIMP) and will be administered in combination with zolbetuximab.<br>**Pembrolizumab**<br>Pembrolizumab is an AxMP/nIMP and will be administered in combination with zolbetuximab.<br>**Nivolumab**<br>Nivolumab is an AxMP/nIMP and will be administered in combination with zolbetuximab and mFOLFOX6.<br>**FLOT**<br>FLOT components are AxMP/nIMP and will be administered in combination with zolbetuximab.
**Dose:**<br>Cohorts 1A and 2: 800 mg/m² loading dose followed by subsequent doses of 600 mg/m² every 3 weeks.<br>Cohort 3A: 800 mg/m² loading dose followed by subsequent doses of 600 mg/m² every 3 weeks. May be de-escalated to 600 mg/m² followed by subsequent doses of 600 mg/m² every 3 weeks if DLTs are observed.<br>Cohort 4A: 800 mg/m² loading dose followed by subsequent doses of 400 mg/m² every 2 weeks. May be de-escalated to 600 mg/m² loading dose followed by subsequent doses of 400 mg/m² every 2 weeks if DLTs are observed.<br>Cohort 4B: Will be dependent on the recommended regimen determined in Cohort 4A.<br>Cohort 5: 800 mg/m² loading dose followed by subsequent doses of 400 mg/m² every 2 weeks for 4 cycles preoperatively and 4 cycles postoperatively. If the zolbetuximab 800 mg/m² loading dose administered on cycle 1 day 1 is assessed as not tolerable based on the DLT assessment, then 3 to 6 subjects will be treated with zolbetuximab 600 mg/m² loading dose on cycle 1 day 1, followed by zolbetuximab 400 mg/m² on day 1 of cycles 2-4. For subjects who experience a DLT during preoperative treatment on the loading dose of 800 mg/m², the postoperative loading dose may be reduced to 600 mg/m². However, for subjects who experience a DLT during preoperative treatment on a loading dose of 600 mg/m², the postoperative loading dose may not be omitted, but dose reduction/modification can be considered for FLOT based on investigator judgment.
**Mode of Administration:**<br>Zolbetuximab will be administered as a minimum 2-hour intravenous (IV) infusion. IV infusion may be interrupted or slowed down to manage toxicity. Please refer to the Pharmacy Manual and Infusion Guidelines for more detailed information.

**mFOLFOX6 (Cohorts 2, 4A and 4B Only)**<br>**Doses and Modes of Administration:**<br>• Oxaliplatin: 85 mg/m^2 IV infusion over 2 hours or per institutional guidelines every 2 weeks for a maximum of 4 cycles (3 treatments in each cycle). NOTE: ECG is required to be performed and assessed locally prior to every oxaliplatin infusion (before any antiemetic treatment) and following completion of every oxaliplatin infusion. ECG should be performed 48 hours *prior* to and up to 6 hours *following* every oxaliplatin infusion. Oxaliplatin administration and electrolyte levels should be managed according to the investigator's judgment for subjects with grade 1 or 2 hypokalemia, hypomagnesemia and/or hypocalcemia.<br>• Leucovorin: 400 mg/m^2 IV infusion over 2 hours every 2 weeks (or levofolinic acid given at either the protocol-recommended doses or as deemed appropriate by the investigator in accordance with institutional standard of care).<br>***SPECIFIC TO JAPAN:*** Or, levofolinate 200 mg/m^2 IV infusion over 2 hours or per institutional standard of care every 2 weeks for 4 cycles. Levofolinate can be continued beyond 4 cycles based on investigator's judgment.<br>• 5-FU Bolus: 400 mg/m^2 IV bolus over 5 to 15 minutes or infused per institutional guidelines every 2 weeks.<br>• 5-FU Infusion: 2400 mg/m^2 continuous IV infusion over 46 to 48 hours or per institutional guidelines every 2 weeks.<br>• Cohort 4 Only: Infusion should begin after the completion of nivolumab.
**Pembrolizumab (Cohort 3A Only)**<br>**Dose:**<br>Pembrolizumab will be administered at a dose of 200 mg intravenously over 30 minutes on day 1 of every 21-day cycle and will be infused 1 hour after the zolbetuximab infusion is completed. Dose reductions are not permitted.<br>**Mode of Administration:**<br>Pembrolizumab should be given intravenously according to institutional guidelines, published guidelines and the respective product prescribing information, and dosed according to this protocol.
**Nivolumab (Cohorts 4A and 4B Only)**<br>**Dose:**<br>Nivolumab will be administered at a dose of 240 mg intravenously over 30 minutes on day 1 of every 2-week cycle and will be infused 30 minutes after the zolbetuximab infusion is completed. Dose reductions are not permitted.<br>**Mode of Administration:**<br>Nivolumab should be given intravenously according to institutional guidelines, published guidelines and the respective product prescribing information, and dosed according to this protocol.

**FLOT (Cohort 5 Only)**<br>**Doses and Modes of Administration:**<br>• Docetaxel: 50 mg/m^2 IV infusion over 1 hour on cycles 1 and 5 day 2 and cycles 2-4 and 6-8 days 1 or 2.<br>• Oxaliplatin: 85 mg/m^2 IV infusion over 2 hours or per institutional guidelines on cycles 1 and 5 day 2 and cycles 2-4 and 6-8 days 1 or 2.<br>○ **NOTE**: ECG is required to be performed and assessed locally prior to every oxaliplatin infusion (before any antiemetic treatment) and following completion of every oxaliplatin infusion. ECG should be performed 48 hours prior to and up to 6 hours following every oxaliplatin infusion. Oxaliplatin administration and electrolyte levels should be managed according to the investigator's judgment for subjects with grade 1 or 2 hypokalemia, hypomagnesemia and/or hypocalcemia.<br>• Leucovorin: 200 mg/m^2 IV infusion over 2 hours on cycles 1 and 5 day 2 and cycles 2-4 and 6-8 days 1 or 2 (or levofolinic acid given as deemed appropriate by the investigator in accordance with institutional standard of care).<br>○ ***SPECIFIC TO JAPAN:*** Or, levofolinate 100 mg/m^2 IV infusion over 2 hours or per institutional standard of care on cycles 1 and 5 day 2 and cycles 2-4 and 6-8 days 1 or 2.<br>• Fluorouracil (5-FU): 2600 mg/m^2 IV infusion over a 24-hour period on cycles 1 and 5 day 2 and cycles 2-4 and 6-8 days 1 or 2.
**Antiemetic Premedication**<br>Prophylactic antiemetics should be given according to institutional standards, published guidelines and the respective product package insert(s). Antiemetic premedication should be started or taken at least 30 minutes prior to each dose of zolbetuximab, mFOLFOX6 (with or without nivolumab), pembrolizumab and FLOT.<br>On days when subjects receive both zolbetuximab and mFOLFOX6 (Cohorts 2 and 4 only), pembrolizumab (Cohort 3 only), or FLOT (Cohort 5), antiemetic premedication will be given prior to zolbetuximab administration. It is recommended that the prophylactic antiemetic regimen include (but is not limited to) the following agents:<br>• NK-1 receptor blockers<br>• 5-HT3 receptor blockers*<br>Antiemetics are authorized products and will be used in this study according to the marketing authorizations.
* To minimize the risk of Torsades de Pointes, administer 5-HT3 receptor blockers with caution to subjects who have or may develop QTc prolongation.<br>The impact of corticosteroids on the potential efficacy of zolbetuximab, pembrolizumab or nivolumab, is not known. Therefore, use corticosteroids with caution if necessary as clinically indicated.

Duration of Treatment:

Cohort 1 subjects will receive zolbetuximab until disease progression, toxicity requiring cessation, start of another anti-cancer treatment or other treatment discontinuation criteria are met.

Cohort 2 subjects will receive zolbetuximab until disease progression, toxicity requiring cessation, start of another anti-cancer treatment or other treatment discontinuation criteria are met. Subjects will also receive mFOLFOX6 (or some of its components, as described below) until disease progression, toxicity requiring cessation, start of another anti-cancer treatment or other treatment discontinuation criteria are met.

Cohort 3 subjects will receive zolbetuximab and pembrolizumab until disease progression, toxicity requiring cessation, start of another anti-cancer treatment or other treatment discontinuation criteria are met for either drug. Subjects without disease progression will receive pembrolizumab for up to 24 months.

Cohort 4 subjects will receive zolbetuximab until radiographic disease progression, toxicity requiring cessation, start of another anti-cancer treatment or other treatment discontinuation criteria are met. Subjects will also receive mFOLFOX6 (or some of its components, as described below) and nivolumab until radiographic disease progression, toxicity requiring cessation, start of another anti-cancer treatment or other treatment discontinuation criteria are met.

Cohort 5 subjects will receive zolbetuximab in combination with FLOT for four 2-week cycles preoperatively and four 2-week cycles postoperatively.

Study Treatment Discontinuation Criteria

Discontinuation from treatment is defined as a subject who enrolled in the study and for whom all study treatment (zolbetuximab, mFOLFOX6 [all components], nivolumab, pembrolizumab and/or FLOT) is permanently discontinued for any reason.

The subject will be discontinued from the treatment period if any of the following occur:

- Investigator determines it is in the subject's best interest to discontinue study treatment.
- Subject develops radiographic disease progression per RECIST 1.1 criteria based on assessment by an independent central reader (Cohort 4 only: by investigator assessment).
    - If there is radiographic evidence of PD; however, the investigator believes the subject is continuing to derive clinical benefit (asymptomatic and/or without worsening of performance status or overall health) from study drug and an increase in tumor burden is not likely to affect vital organ function, the subject may remain on study drug until the next scheduled radiographic assessment.
        - If the next radiographic assessment indicated PD per RECIST 1.1, which is confirmed by the independent central reader, then the subject must be discontinued from study drug.
        - In the rare event where PD suspected on initial assessment, but is not confirmed on subsequent scan by the investigator or central reviewer, the subject may continue in the study.
    - The investigator should make every effort to immediately submit radiographic assessments for central review when PD is either suspected or confirmed or uncertainty exists.
- Subject develops clinical progression per investigator assessment and radiographic assessment is not medically feasible due to the subject's condition.
- Subject starts another systemic chemotherapy, immunotherapy, radiotherapy or other treatment intended for antitumor activity.
- Subject starts another investigational agent or device.
- Subject develops unacceptable toxicity that results in discontinuation of zolbetuximab (Subjects in Cohort 2, Cohort 3A, Cohort 4 or Cohort 5 may continue on mFOLFOX6/5-FU and leucovorin or folinic acid, pembrolizumab, nivolumab or FLOT, as applicable, per investigator discretion.).
- Subject has a delay of zolbetuximab, mFOLFOX6, nivolumab, pembrolizumab or FLOT treatment for > 28 days from when the next zolbetuximab, mFOLFOX6, pembrolizumab, nivolumab or FLOT treatment was scheduled to be administered (> 49 days from when the last dose began if a 3-week cycle [Cohorts 1A, 2 and 3A] and > 42 days from when the last dose began if a 2-week cycle [Cohorts 4 and 5]).
- Inter-current illness that the investigator determines may jeopardize the subject's safety if the subject continues to receive study treatment.
- Female subject becomes pregnant.
- Significant deviation from the protocol or eligibility criteria as determined by the sponsor.
- Subject declines further treatment.
- Subject declines further study participation
- Subject is lost to follow-up despite reasonable efforts by the investigator to locate the subject
- Death
- Sponsor discontinues the study

Endpoints for Evaluation:

Primary:

- ORR of zolbetuximab as a single agent as assessed by an independent central reader

Secondary:

- Pharmacokinetics of zolbetuximab (Cohorts 1A, 2, 3A, 4 and 5): AUC_{inf}, AUC_{inf} [%extrap], AUC_{last}, AUC_{tau}, C_{max}, C_{trough}, t_{max}, $t_{1/2}$, $t_{last,}$ CL, V_z, as appropriate
- Pharmacokinetics of oxaliplatin (Cohort 2) and 5-FU (Cohort 2): AUC_{inf}, AUC_{inf} [%extrap], AUC_{last}, C_{max}, t_{max}, $t_{1/2}$, $t_{last,}$ CL, V_z, as appropriate
- Safety and tolerability of single agent zolbetuximab, in combination with mFOLFOX6 (with or without nivolumab), in combination with pembrolizumab, and in combination with FLOT evaluated by AEs, ECG, vital signs, ECOG performance status and laboratory assessments (NCI-CTCAE version 4.03)
- Safety and tolerability of zolbetuximab + FLOT in Cohort 5 include the following additional assessments:
  - Surgical complications
  - Surgical mortality as defined by death within 30 days of surgery
  - Percentage of subjects able to complete preoperative chemotherapy
  - Perioperative mortality and morbidity at 30 days and 90 days post last dose
  - Percentage of subjects able to start postoperative chemotherapy
  - Percentage of subjects able to complete postoperative chemotherapy
- Immunogenicity of zolbetuximab as a single agent, in combination with mFOLFOX6 (with or without nivolumab), in combination with pembrolizumab, and in combination with FLOT as measured by the frequency of anti-drug antibody (ADA) positive subjects
- HRQoL measured by the QLQ-C30, OG-25, GP, EuroQOL Five Dimensions Questionnaire (EQ-5D) and the HRU questionnaires
- ORR of zolbetuximab in combination with pembrolizumab and in combination with mFOLFOX6 as assessed by an independent central reader
- ORR of zolbetuximab as a single agent, in combination with mFOLFOX6 (with or without nivolumab) and in combination with pembrolizumab by investigator assessment
- DCR, DOR and PFS of zolbetuximab as a single agent and in combination with mFOLFOX6 as assessed by an independent central reader
- DCR, DOR and PFS of zolbetuximab as a single agent and in combination with mFOLFOX6 (with or without nivolumab) as assessed by the investigator
- OS of zolbetuximab as a single agent, in combination with mFOLFOX6 and nivolumab, and in combination with FLOT
- Cohort 5 only: Antitumor activity of zolbetuximab and FLOT as measured by radiological response (restaging) and pathological response ypTNM (pCR)
- Cohort 5 only: DFS
- Cohort 5 only: Minimal residual disease and disease recurrence as measured by circulating tumor DNA (ctDNA)

Exploratory:

- Potential genomic and/or other biomarkers that may be related to treatment outcome of zolbetuximab as a single agent, in combination with mFOLFOX6 (with or without nivolumab) and in combination with FLOT
- Changes in tumor expression of CLDN18.2
- Changes in immune-related biomarkers in tumor tissue and blood samples
- Pharmacokinetics of pembrolizumab (predose and end-of-infusion concentrations) in combination with zolbetuximab
- Pharmacokinetics of nivolumab (predose and end-of-infusion concentrations) in combination with zolbetuximab and mFOLFOX6

Statistical Methods: ***See [Section 7 Statistical Methodology]***

Sample Size Justification:

The sample size of 20 for Cohort 1A is not based on a strict statistical power calculation but is expected to provide adequate early efficacy and safety information.

The sample size of 12 for Cohort 2 is to provide sufficient pharmacokinetic information of zolbetuximab, oxaliplatin and 5-FU.

The sample size of 12 for Cohort 3A is not based on a statistical power calculation but is expected to provide safety information to determine the tolerability of the dose level of interest.

The sample size of 12 for Cohort 4A is not based on a statistical power calculation but is expected to provide safety information to determine the tolerability of the dose level of interest.

The sample size of approximately 65 for Cohort 4B is not based on a strict statistical consideration. The sample size of approximately 65 is expected to yield 50 subjects with high CLDN18.2 expression. For these subjects with high CLDN18.2 expression, assuming an accrual period of 12 months and a follow-up period of 3 to 6 months and 20 to 25 PFS events, the sample size of 50 will provide 70.37% to 76.13% power to detect the difference in PFS with the assumption of a 12-month median PFS vs 8.5-month median PFS (zolbetuximab in combination of nivolumab and mFOLFOX6 vs zolbetuximab and mFOLFOX6) using a 1-sided 15% Type I error. It is assumed that the survival time distributions of both groups are approximated reasonably well by the Weibull distribution with a shape parameter of 1.

The sample size of 12 for Cohort 5 is not based on a statistical power calculation but is expected to provide safety information to determine the tolerability of the dose level of interest.

Analysis populations:

- The Full Analysis Set (FAS) consists of all subjects who were enrolled and received at least 1 dose of zolbetuximab and who have at least 1 post-treatment disease assessment. At least 1 post-treatment disease assessment is defined as, for at least one time point, for either local or central assessment, the tumor imaging overall response does not equal to NE (not evaluable). The FAS will be used for summaries of all efficacy data except PFS and OS, as well as selected demographic and baseline characteristics.
- The Safety Analysis Set (SAF) consists of all subjects who received at least 1 dose of zolbetuximab. The SAF will be used for summaries of demographic and baseline characteristics, PFS, OS and all safety and tolerability related variables.
- The Pharmacokinetic Analysis Set (PKAS) consists of the subset of the SAF for which at least 1 concentration data is available for any analyte. Additional subjects may be excluded from the PKAS at the discretion of the pharmacokineticist. The PKAS is used for all tables and graphical summaries of the pharmacokinetic data.
- The Biomarker Analysis Set (BMAS) consists of the subset of the SAF for which at least 1 pre-treatment or 1 on-treatment biomarker measurement is available. Additional subjects may be excluded from the BMAS at the discretion of the sponsor. The BMAS is used for all tables and graphical summaries of the biomarker data.
- The DLT Evaluation Analysis Set (DEAS) is defined as all subjects in the SAF excluding subjects without a DLT who receive less than the prescribed dose in cycle 1, or do not complete cycle 1 evaluations for a reason other than a DLT (e.g., consent withdrawal).

Efficacy:

ORR and DCR will be summarized using exact 95% confidence interval. The survival curve and median PFS and OS will be estimated using Kaplan-Meier method and will be reported along with corresponding 95% confidence interval. DOR will be estimated using the same method as PFS and OS only for those with CR/PR.

For zolbetuximab and FLOT (Cohort 5), the clinical response to preoperative chemotherapy (timepoints include baseline tumor assessment [prior to study treatment] to before surgery and after completion/discontinuation of preoperative chemotherapy) will be summarized using the percentage of subjects with radiological response at restaging and the percentage of subjects with pathological response (ypTNM). These percentages will be summarized using exact 95% confidence intervals. DFS will be analyzed using the same method as PFS (i.e., Kaplan-Meier method). Minimal residual disease and disease recurrence as measured by ctDNA will be summarized.

Pharmacokinetics:

Descriptive statistics will be used to summarize serum concentrations and parameters of zolbetuximab, pembrolizumab and nivolumab, and plasma concentrations and parameters of oxaliplatin (measured as total and free platinum) and 5-FU. Individual (spaghetti) and mean concentration-time curves on linear and semi-logarithmic scales will be provided as appropriate. Geometric least-squares mean ratio (cycle 2 day 1 vs cycle 1 day 1) and associated 90% confidence intervals will be provided for C_{max} and AUCs of total platinum, free platinum and 5-FU for Cohort 2.

The potential relationship between zolbetuximab immunogenicity and zolbetuximab pharmacokinetic, efficacy and safety profile in subjects may be assessed.

Biomarkers:

Biomarkers may be summarized graphically or descriptively, and summary statistics may be tabulated. Associations between biomarkers and clinical (e.g., efficacy, safety or pharmacodynamics, pharmacokinetics) measures may be performed on subjects who have sufficient baseline and on-study measurements to provide interpretable results for specific parameters.

Pharmacodynamics:

Not Applicable

Safety:

AEs will be coded using MedDRA and graded using NCI-CTCAE. The number and percentage of AEs, SAEs, AEs leading to interruption/discontinuation, AEs leading to death and AEs related to study drug will be summarized by system organ class and preferred term for each cohort. The number and percentage of AEs by toxicity grade will also be summarized for each cohort. All AEs will be listed. Summary statistics will also be provided for laboratory parameters, vital signs, drug exposure and other safety parameters. All DLT incidences will be summarized for Cohorts 3A, 4A and 5.

VI. FLOW CHART AND SCHEDULE OF ASSESSMENTS

Figure 1 Flow Chart for Cohort 1A

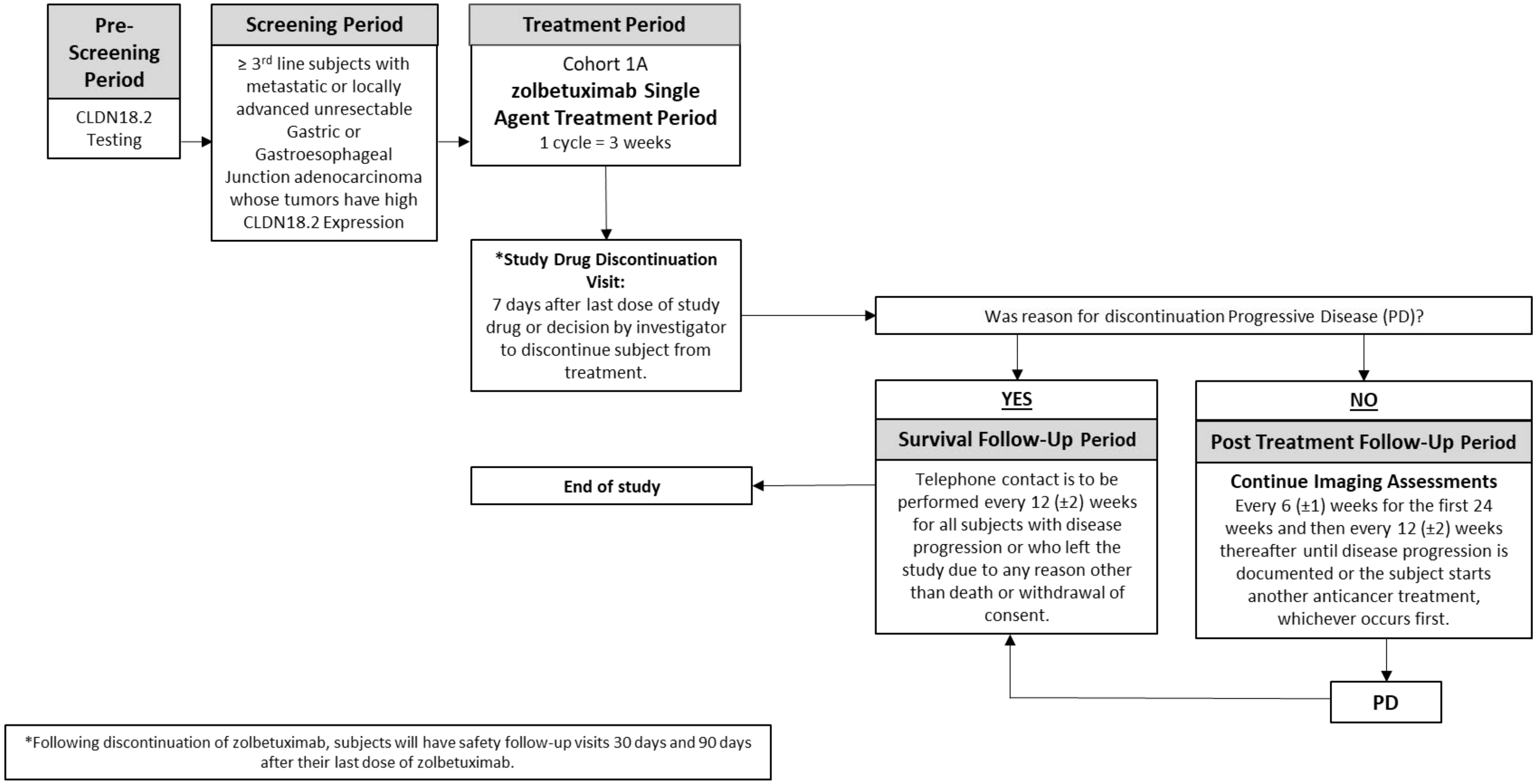

CLDN: claudin; PD: progressive disease

Figure 2 Cohort 1A Treatment Period Dosing Schedule

Treatment Period Dosing Schedule												
**Cycle 1**	**Cycle 2**	**Cycle 3**	**Cycle 4**	**Cycle 5**	**Cycle 6**	**Cycle 7**	**Cycle 8**	**Cycle 9**	**Cycle 10**	**Cycle 11**	**Cycle 12**	**Cycle 13+** (q3 weeks)
Z	Z	Z	Z	Z	Z	Z	Z	Z	Z	Z	Z	Z

Z	= **zolbetuximab (q3 weeks)**

*1 cycle = 3 Weeks

q3: once every 3

Figure 3 Study Drug Dosing Schematic (Cohort 1A)

Cohort 1A Dosing Visits

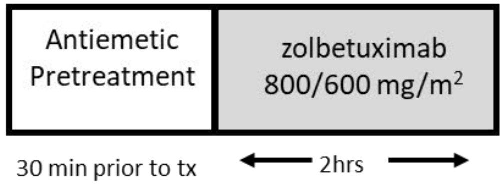

$ $800 mg/m² loading dose followed by subsequent doses of 600 mg/m² every 3 weeks**

It is recommended that zolbetuximab infusion not exceed 6 hours from start of infusion.

tx: treatment

Figure 4 Flow Chart for Cohort 2

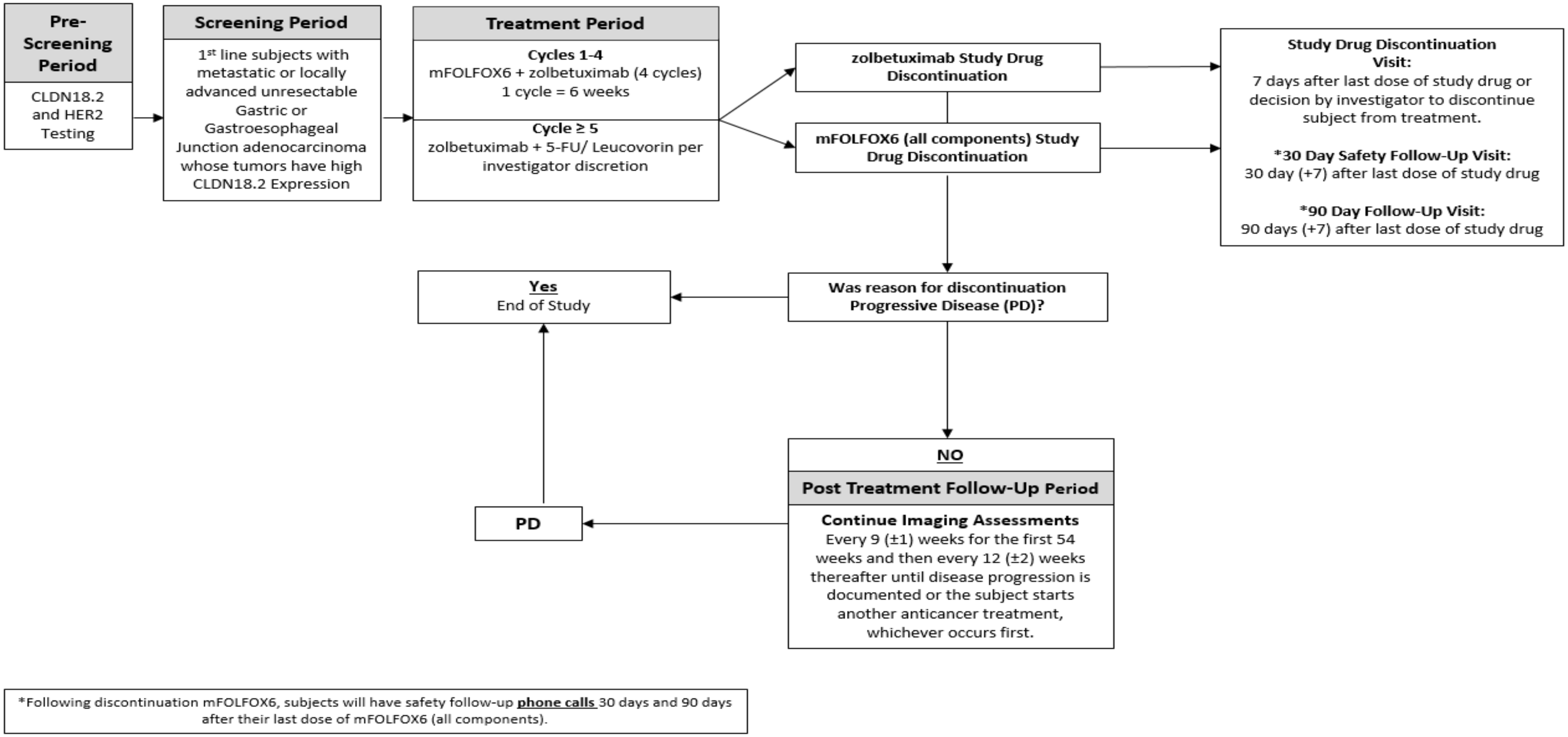

5-FU: fluorouracil; CLDN: claudin; HER2: human epidermal growth factor receptor 2; mFOLFOX6: 5-fluorouracil, leucovorin or folinic acid and oxaliplatin; PD: progressive disease.

Figure 5 Cohort 2 Treatment Period Dosing Schedules

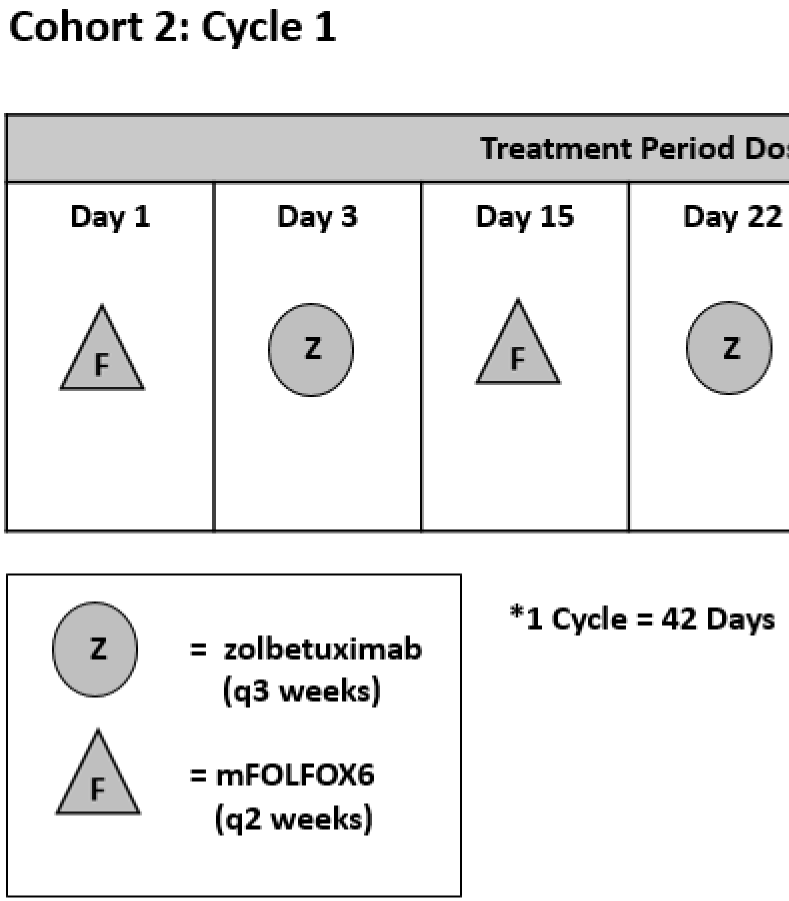

Cohort 2: Cycles ≥ 2

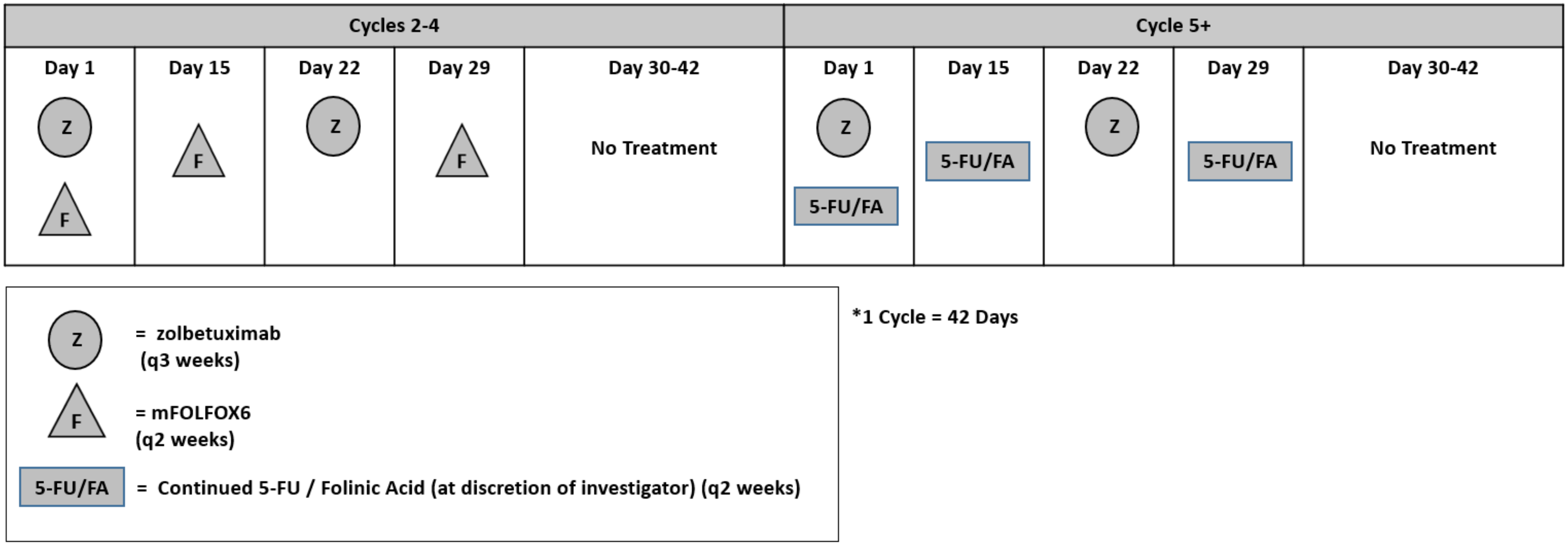

5-FU: fluorouracil; mFOLFOX6: 5-fluorouracil, leucovorin or folinic acid and oxaliplatin

Figure 6 Study Drug Dosing Schematics (Cohort 2)

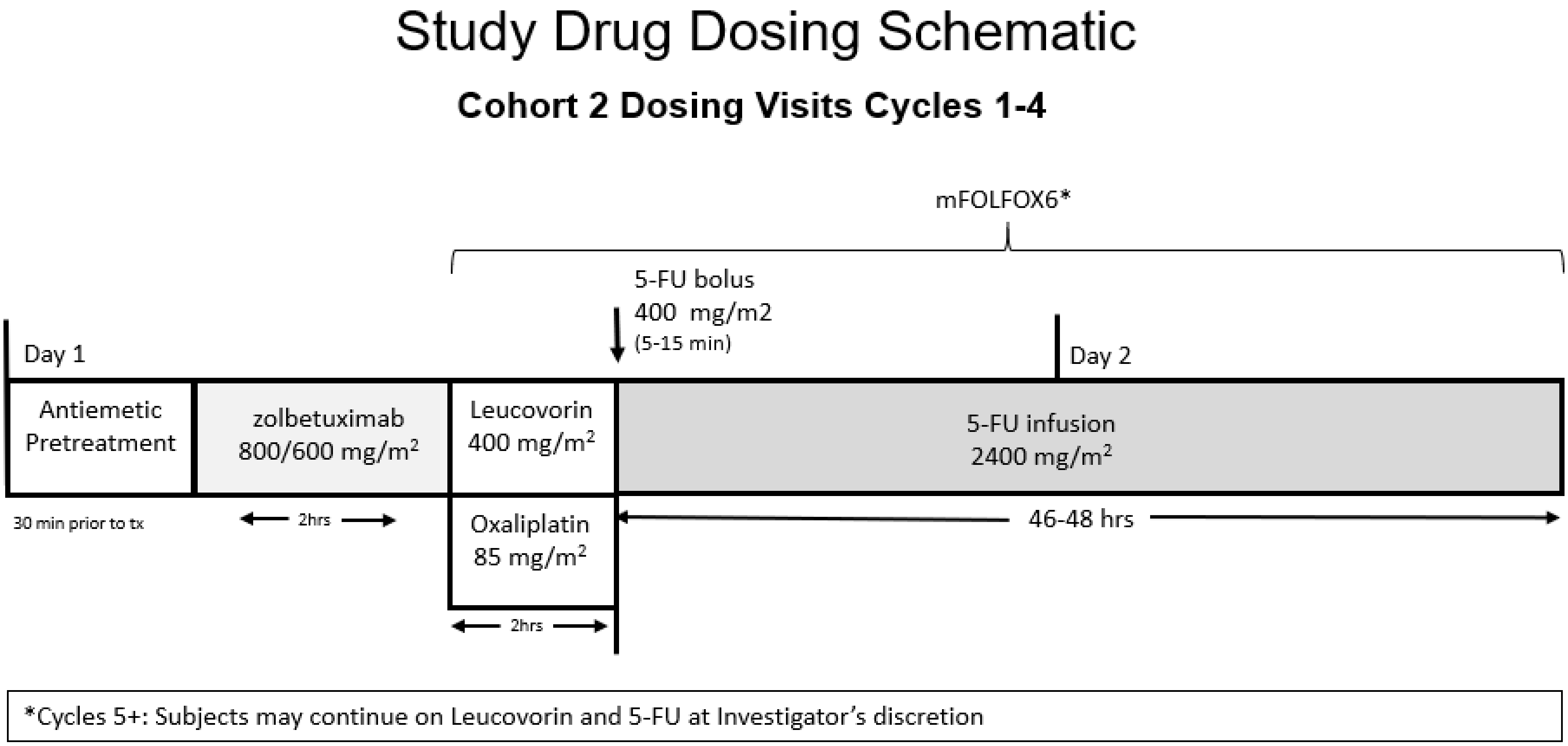

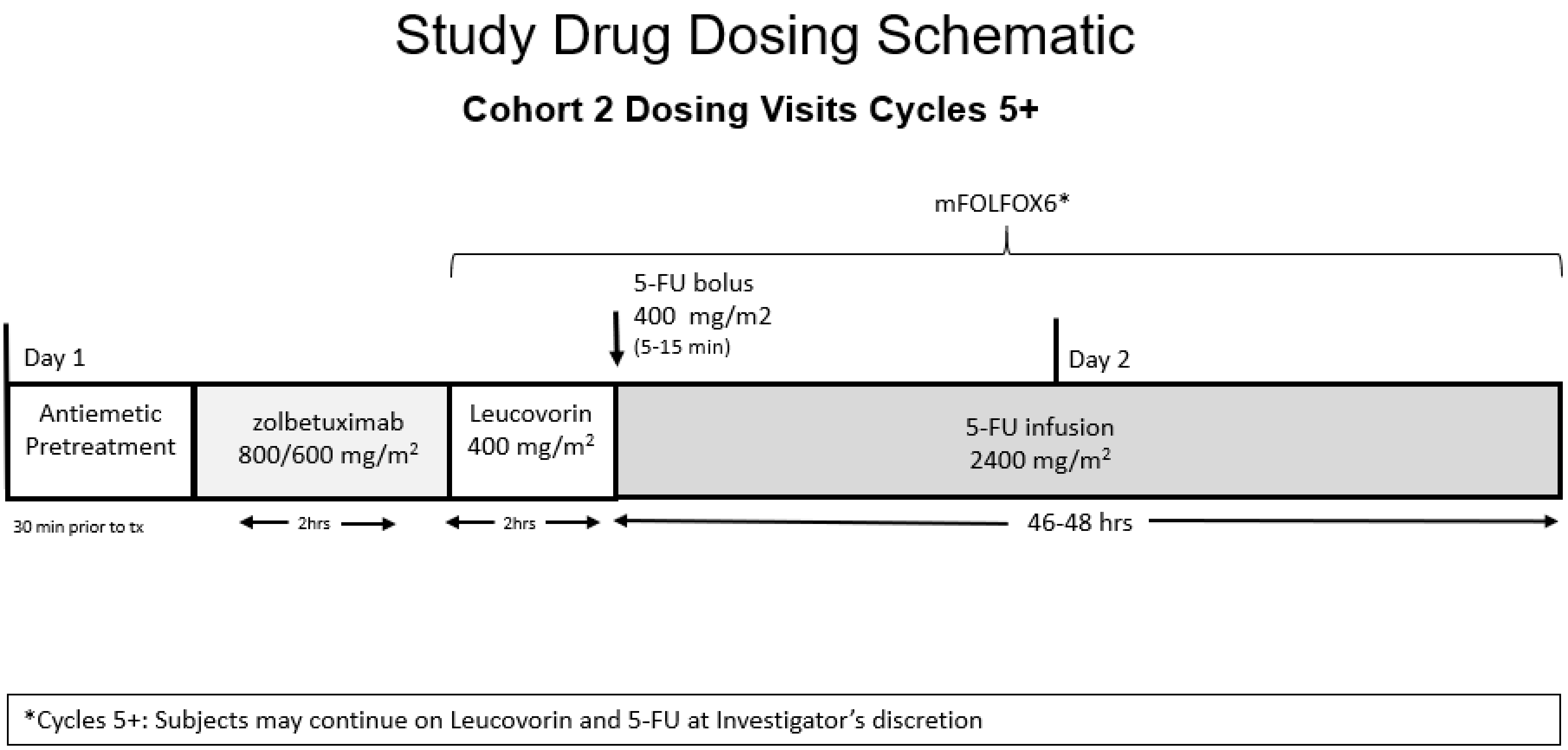

5-FU: fluorouracil; mFOLFOX6: 5-fluorouracil, leucovorin or folinic acid and oxaliplatin; tx: treatment

Figure 7 Flow Chart for Cohort 3A

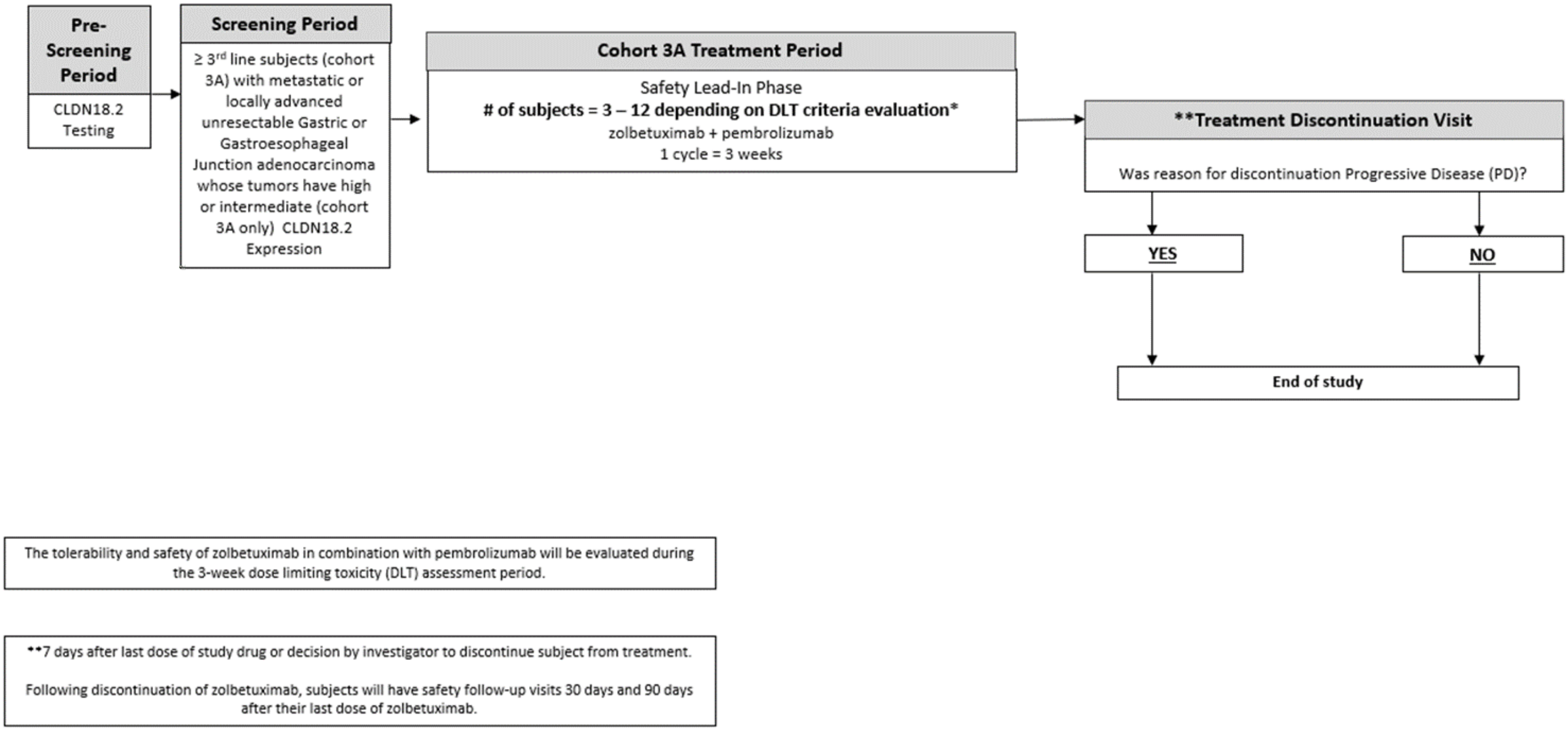

CLDN: claudin; DLT: dose-limiting toxicity; PD: progressive disease

Figure 8 Cohort 3 Treatment Period Dosing Schedules

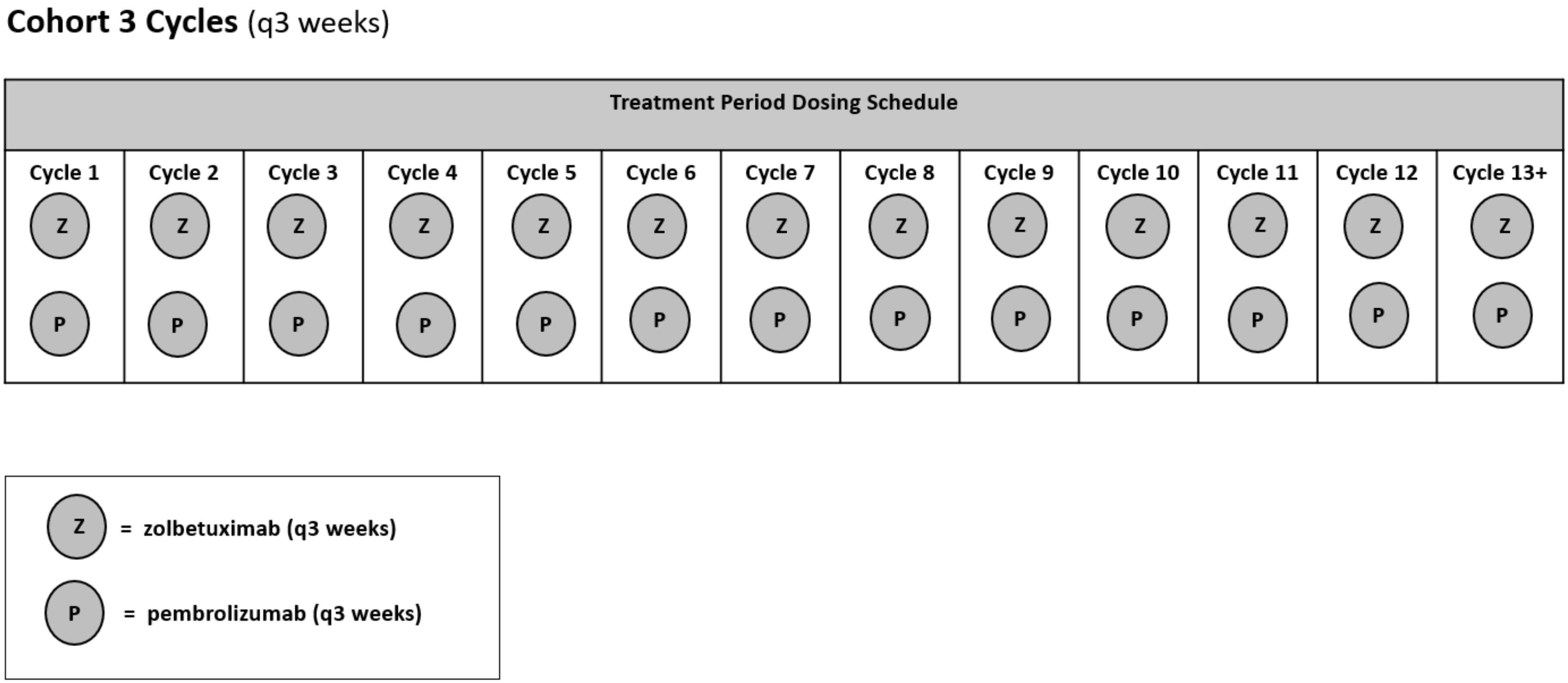

q3: once every 3

Figure 9 Flow Chart for Cohort 4

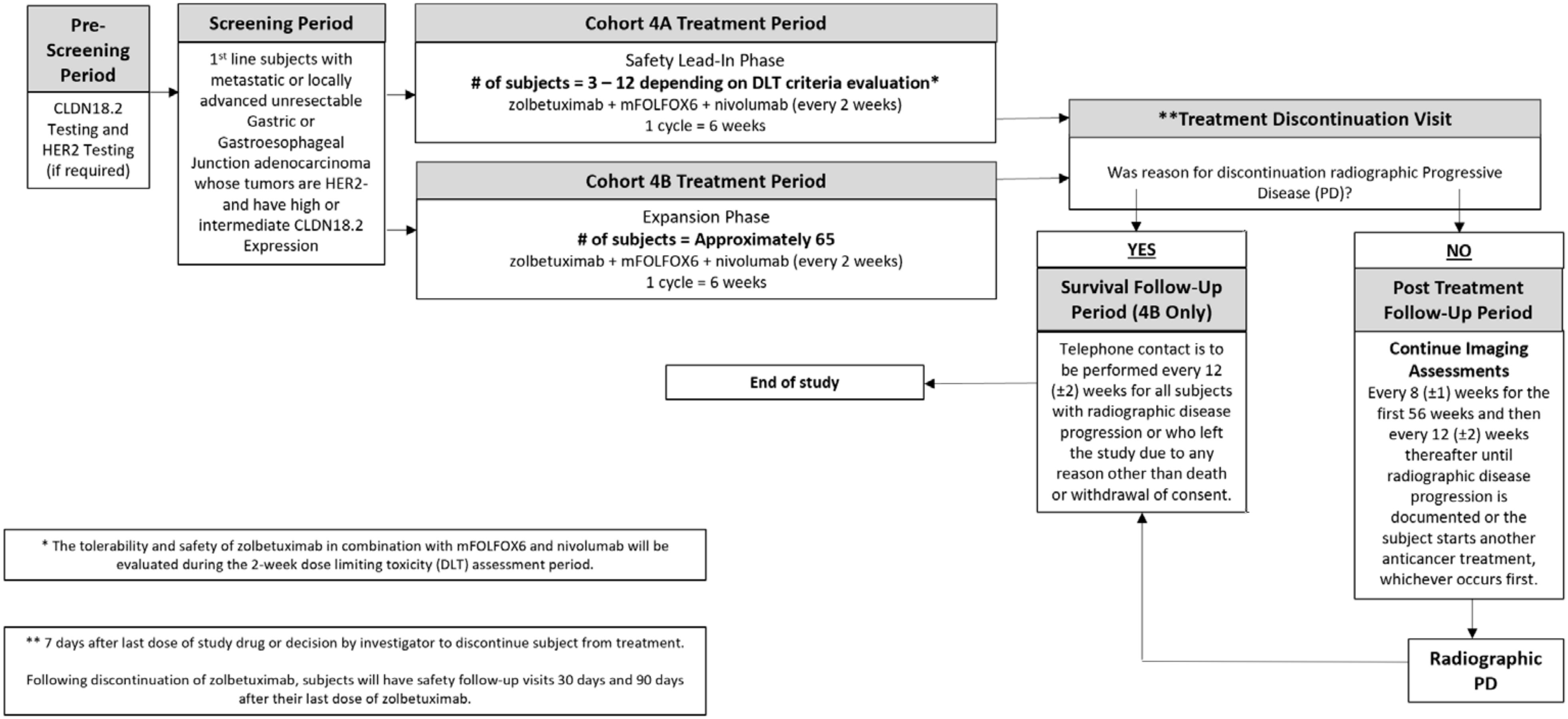

CLDN18.2: claudin-18 isoform 2; DLT: dose limiting toxicity; HER2: human epidermal growth factor receptor 2; mFOLFOX6: 5-fluorouracil, leucovorin or folinic acid and oxaliplatin; PD: progressive disease

Figure 10 Cohort 4 Treatment Period Dosing Schedules

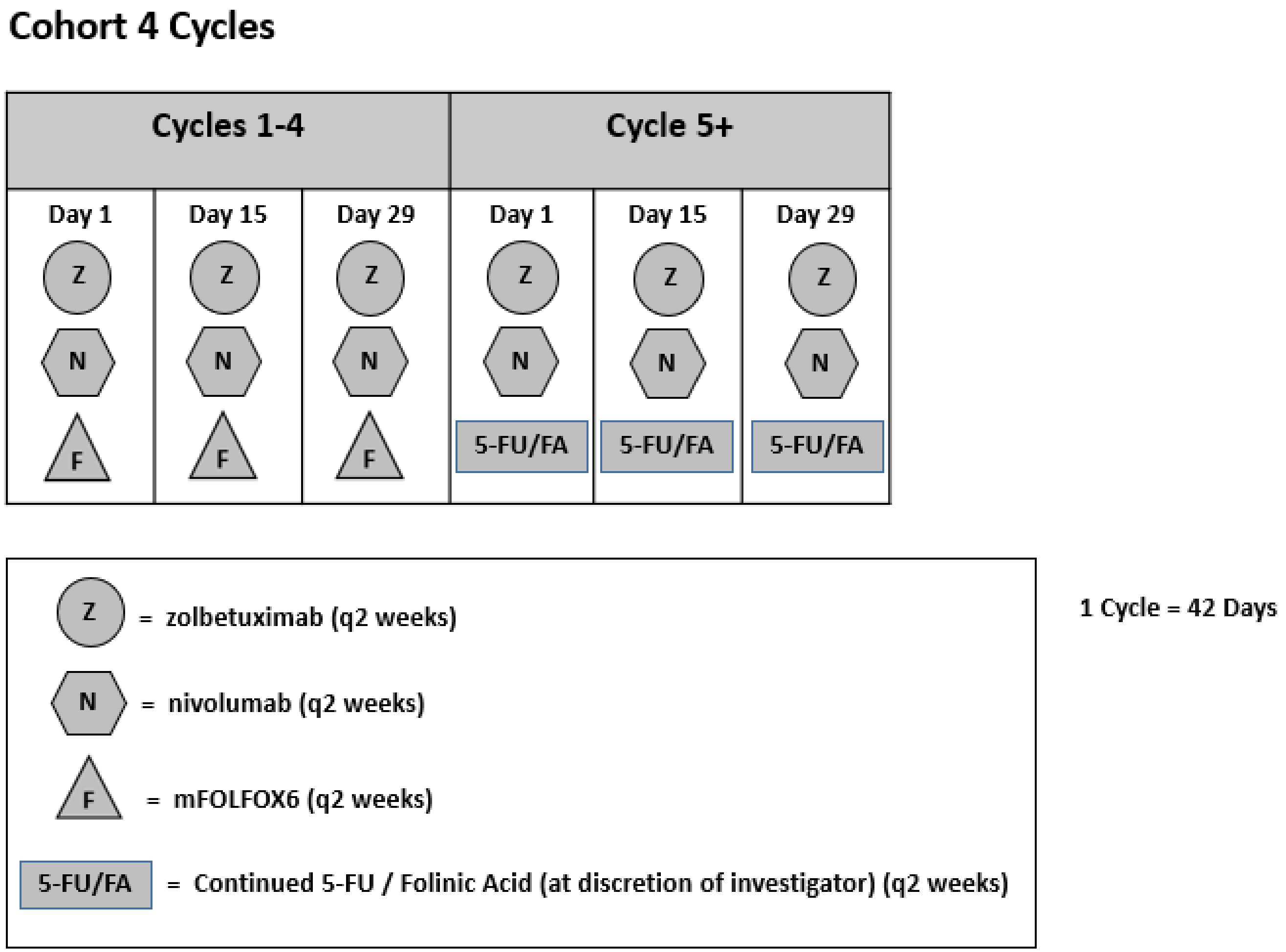

Study Drug Dosing Schematic

Cohort 4 Dosing Visits Cycles 1-4

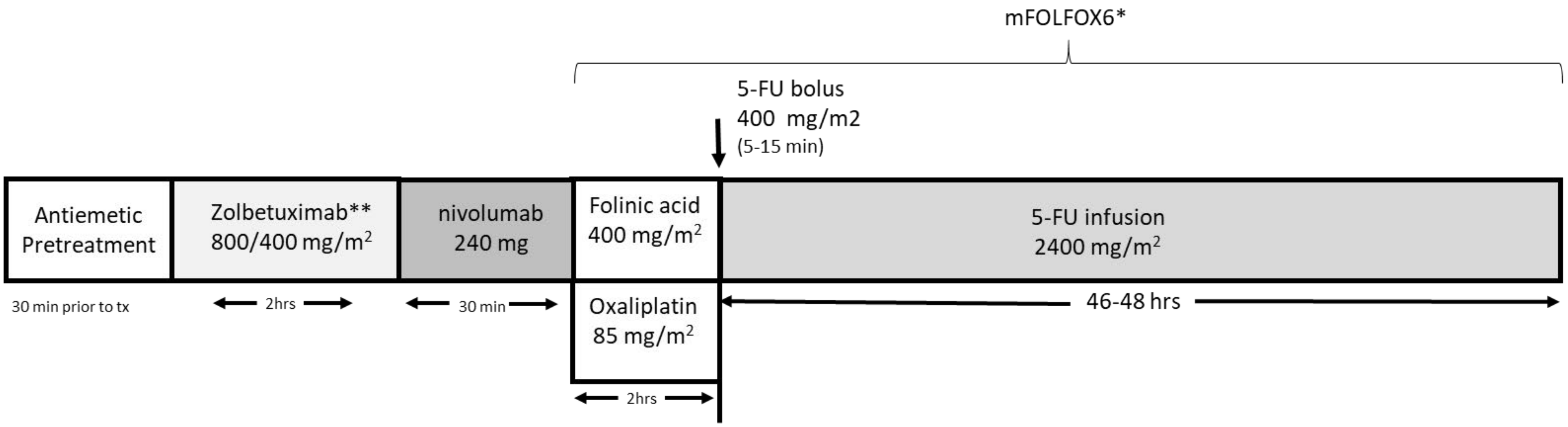

**Zolbetuximab will be administered with a loading dose of 800 mg/m² at cycle 1 day 1 followed by subsequent maintenance doses of 400 mg/m².

5-FU: fluorouracil; mFOLFOX6: 5-fluorouracil, leucovorin or folinic acid and oxaliplatin; tx: treatment

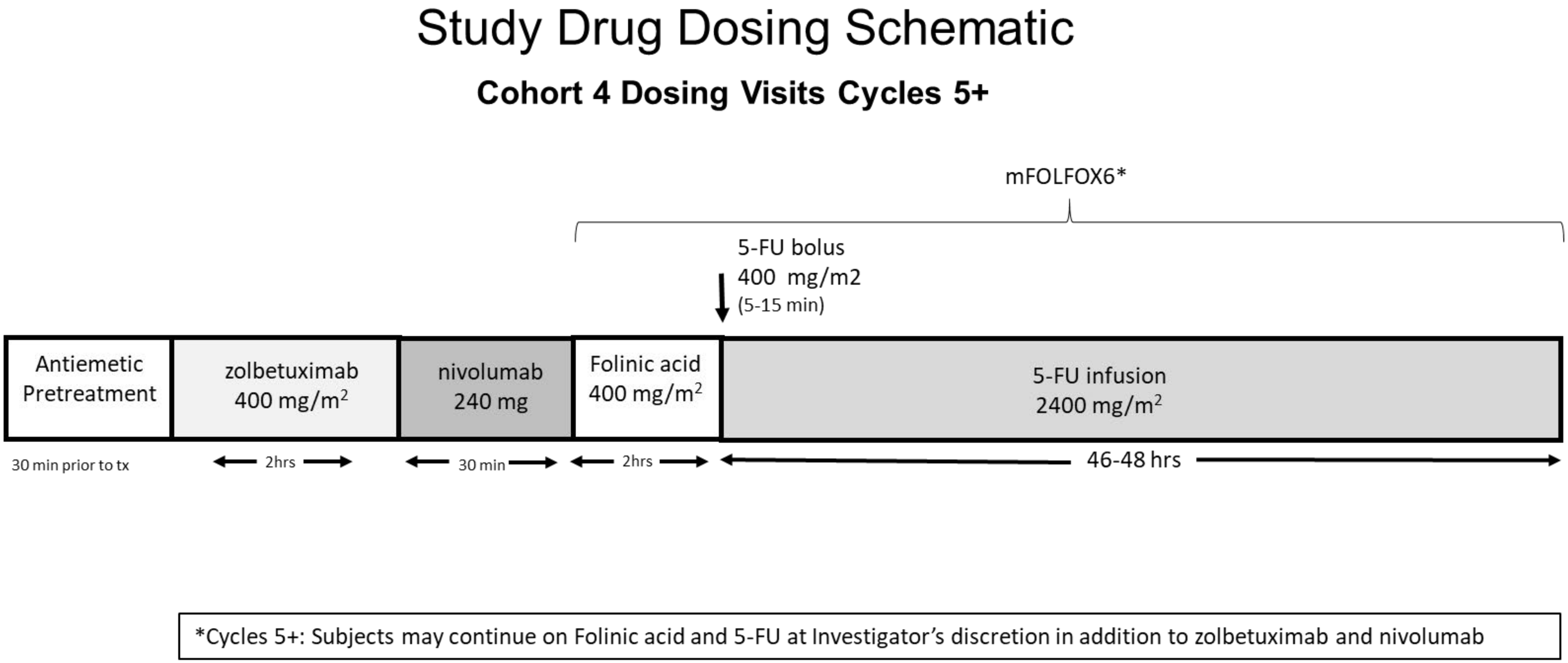

Figure 11 Flow Chart for Cohort 5

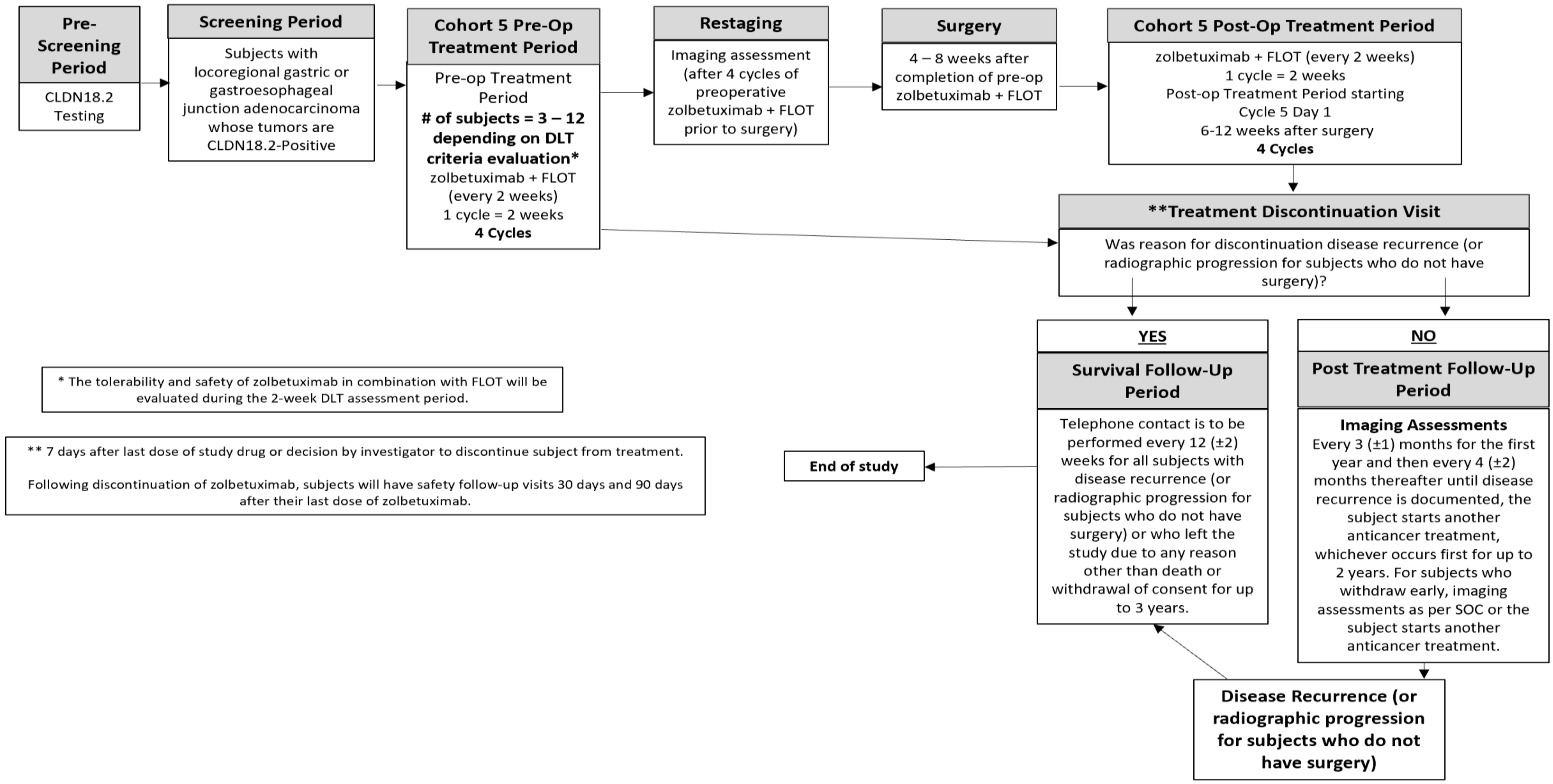

CLDN18.2: claudin-18 isoform 2; DLT: dose-limiting toxicity; FLOT: fluorouracil, leucovorin or folinic acid, oxaliplatin and docetaxel; Post-Op: postoperative; Pre-Op: preoperative; SOC: system organ class

Figure 12 Cohort 5 Treatment Period Dosing Schedules

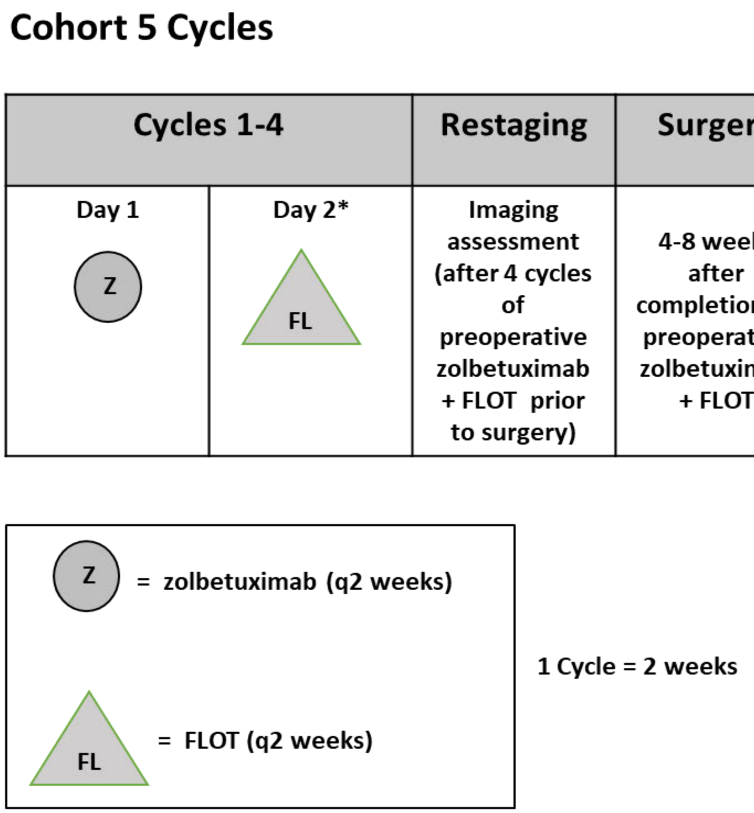

FLOT: fluorouracil, leucovorin or folinic acid, oxaliplatin and docetaxel

*For cycles 1 and 5, zolbetuximab is administered on day 1 and FLOT administered on day 2. For cycles 2-4 and 6-8, FLOT may be administered following zolbetuximab on day 1 or can be administered on day 2 as per investigator's clinical judgment.

Study Drug Dosing Schematic

Cohort 5 Dosing Visits

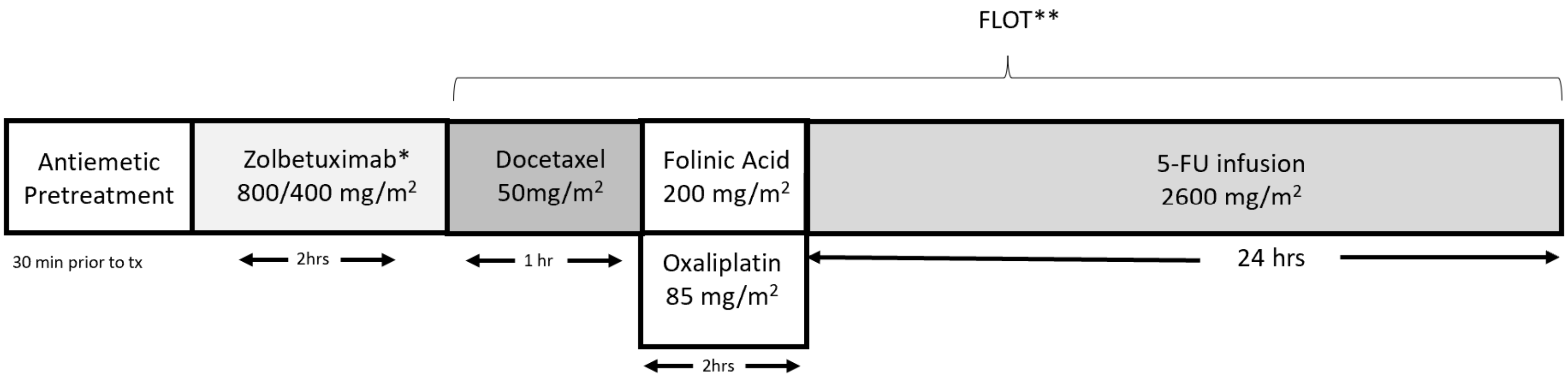

*Zolbetuximab will be administered with a loading dose of 800 mg/m² at cycle 1 day 1 followed by subsequent maintenance doses of 400 mg/m².
** For cycles 1 and 5, zolbetuximab is administered on day 1 and FLOT is administered on day 2. For cycles 2-4 and 6-8, FLOT may be administered following zolbetuximab on day 1, or can be administered on day 2 per investigator's clinical judgment.

Table 1 Schedule of Assessments for Cohort 1A

VISIT	Pre-Screening[1]	Screening[2]	Cohort 1A (single agent zolbetuximab)										Study Drug Discontinuation Visit[26]	30-Day Safety Follow-up Visit[27]	90-Day Follow-up Visit[28]	Post-treatment Follow-up[29]	Survival Follow-up[30]
**Cycle[3]**			**1**	**2**	**3**	**4**	**5**	**6**	**7**	**8**	**9 to 12**	**≥ 13**					
**Day**		-28 to -1	1	22	43	64	85	106	127	148	169 to 274	295					
Visit Window (business days)		-28 to -1	0	+ 2	+ 2	+ 2	+ 2	+ 2	+ 2	+ 2	+ 2	+ 2	+ 7	+ 7	+ 7	± 14	± 14
Pre-Screening ICF	X																
Tumor Sample for Eligibility[1]	X																
Main Study ICF		X															
Tumor Biopsy[4]		X															
Medical and Disease History		X															
Confirmation of Eligibility		X	X														
Cohort Assignment[5]			X														
**Study Drug Administration**																	
Antiemetic Pretreatment[6]			X	X	X	X	X	X	X	X	X	X					
Zolbetuximab[7]			X	X	X	X	X	X	X	X	X	X					
Post-infusion Observation Period[8]			X	X	X	X	X	X	X	X	X	X					
**Physical Examination/Assessments**																	
Physical Examination[9]		X	X	X	X	X	X	X	X	X	X	X	X	X			
Weight[9]		X	X	X	X	X	X	X	X	X	X	X	X	X			
ECOG Performance Status[9]		X	X	X	X	X	X	X	X	X	X	X	X	X			
Vital Signs[10]		X	X	X	X	X	X	X	X	X	X	X	X	X			

Table continued on next page

**VISIT**	**Pre-Screening[1]**	**Screening[2]**	**Cohort 1A (single agent zolbetuximab)**										**Study Drug Discontinuation Visit[26]**	**30-Day Safety Follow-up Visit[27]**	**90-Day Follow-up Visit[28]**	**Post-treatment Follow-up[29]**	**Survival Follow-up[30]**
**Cycle[3]**			**1**	**2**	**3**	**4**	**5**	**6**	**7**	**8**	**9 to 12**	**≥ 13**					
**Day**		-28 to -1	1	22	43	64	85	106	127	148	169 to 274	295					
Visit Window (business days)		-28 to -1	0	+ 2	+ 2	+ 2	+ 2	+ 2	+ 2	+ 2	+ 2	+ 2	+ 7	+ 7	+ 7	± 14	± 14
**Laboratory Assessments**																	
Biochemistry[11]		X	X	X	X	X	X	X	X	X	X	X	X	X			
Hematology[11]		X	X	X	X	X	X	X	X	X	X	X	X	X			
Urinalysis[12]		X	If clinically indicated										X	X			
Cytokine/Chemokine and/or Tryptase			If clinically indicated														
TSH and T4		X	If clinically indicated														
PT, PTT and INR[13]		X	If clinically indicated														
Serum Pregnancy Test[14]		X															
Urine Pregnancy Test[15]			X	X	X	X	X	X	X	X	X	X	X	X			
Exploratory Biomarkers (Serum)[16]			X	X	X	X		X		X			X				
Exploratory Biomarkers (Plasma)[16]			X	X	X	X		X		X			X				
Immune Cell Subsets (Whole blood)[16]			X	X	X	X		X		X			X				
Genetic Immune Polymorphisms (Whole blood)[16]			X														
Cryopreserved PBMC [16]			X	X	X	X		X		X			X				

Table continued on next page

VISIT	Pre-Screening[1]	Screening[2]	Cohort 1A (single agent zolbetuximab)										Study Drug Discontinuation Visit[26]	30-Day Safety Follow-up Visit[27]	90-Day Follow-up Visit[28]	Post-treatment Follow-up[29]	Survival Follow-up[30]
**Cycle[3]**			**1**	**2**	**3**	**4**	**5**	**6**	**7**	**8**	**9 to 12**	**≥ 13**					
**Day**		-28 to -1	1	22	43	64	85	106	127	148	169 to 274	295					
Visit Window (business days)		-28 to -1	0	+ 2	+ 2	+ 2	+ 2	+ 2	+ 2	+ 2	+ 2	+ 2	+ 7	+ 7	+ 7	± 14	± 14
Anti-Drug Antibodies (Immunogenicity)			See Table 2 below for sample collection schedule														
Pharmacokinetics			See Table 2 below for pharmacokinetic collection schedule														
Whole Blood Sample for PGx (optional)[17]			X														
**Cardiac Safety**																	
Single 12-lead ECG[18]		X	If clinically indicated										X	X			
Triplicate ECG		See Table 2 below for Triplicate ECG schedule (Cohort 1A only)															
**Radiology**																	
Image Assessment[19]		X	Every 6 (± 1) weeks from C1D1 for the first 24 weeks and then every 12 (± 2) weeks thereafter.														
**HRQoL**																	
HRQoL[20]		X	X	X	X	X	X	X	X	X	X	X	X	X	X		
**Tissue Samples**																	
Baseline Tumor Sample[21]		X															
On-Treatment Biopsy[22]					X												
Post-progression Tumor Sample (optional)[23]													X				
**Safety and Survival Assessment**																	
Concomitant Medication[24]		X	X	X	X	X	X	X	X	X	X	X	X	X	X		

Table continued on next page

VISIT	Pre-Screening[1]	Screening[2]	Cohort 1A (single agent zolbetuximab)										Study Drug Discontinuation Visit[26]	30-Day Safety Follow-up Visit[27]	90-Day Follow-up Visit[28]	Post-treatment Follow-up[29]	Survival Follow-up[30]
**Cycle[3]**			**1**	**2**	**3**	**4**	**5**	**6**	**7**	**8**	**9 to 12**	**≥ 13**					
**Day**		-28 to -1	1	22	43	64	85	106	127	148	169 to 274	295					
Visit Window (business days)		-28 to -1	0	+ 2	+ 2	+ 2	+ 2	+ 2	+ 2	+ 2	+ 2	+ 2	+ 7	+ 7	+ 7	± 14	± 14
AEs/SAEs[25]		X	X	X	X	X	X	X	X	X	X	X	X	X	X		
Survival Assessment[30]																	X

ADA: anti-drug antibody; AE: adverse event; βHCG: beta human chorionic gonadotropin; BSA: body surface area; C1D1: cycle 1 day 1; CLDN: claudin; CT: computerized tomography; eCRF: electronic case report form; ECG: electrocardiogram; ECOG: Eastern Cooperative Oncology Group; EQ-5D: EuroQOL five dimensions questionnaire; FFPE: formalin-fixed paraffin-embedded; GP: Global Pain; HER2: human epidermal growth factor receptor 2; HRQoL: health-related quality of life; HRU: Health Resource Utilization; ICF: informed consent form; INR: international normalized ratio; IRR: infusion related reaction; IV: intravenous; MRI: magnetic resonance imaging; OG-25: Oesophago-Gastric Module (EORTC QLQ-OG-25); ORR: objective response rate; QLQ-C30: Quality of Life Questionnaire - Core Questionnaire; PBMC: peripheral blood mononuclear cells; PET: positron emission tomography; PFS: progression-free survival; PGx: pharmacogenomics; PT: prothrombin time; PTT: partial thromboplastin time; SAE: serious adverse event; T4: thyroxine; TSH: thyroid-stimulating hormone

Laboratory tests will be performed predose according to the Schedule of Assessments and sent to a central laboratory for analysis. In case of multiple laboratory data within this period, the most recent data should be used.

1. Pre-Screening: FFPE tumor specimens will be collected for central pre-screening tests to determine CLDN18.2 status. There should be sufficient tumor tissue in the specimen used to send to the central laboratory for testing. Archival tumor tissue is preferred. A minimum of 1 FFPE tumor tissue block (preferred) OR a minimum of 9 FFPE unstained sections are required. If slides are submitted, the slides should be freshly cut from the FFPE block within the time frame described in the laboratory manual. If archival tumor tissue is insufficient or unavailable for pre-screening, the subject will enter the screening period and a biopsy will be performed to obtain the tumor sample for eligibility. If ≥ 9 slides cannot be provided, the sponsor should be contacted for further guidance.
2. Screening period is 28 days. Subjects may be rescreened once. Any procedures that fall outside of the screening period must be repeated. If more than 1 assessment is taken during the screening period, the assessment closest to enrollment date should be used for eligibility.
3. Assessments should be collected on day 1 of each corresponding cycle.

Footnotes continued on next page

4. If archival tumor tissue is insufficient or unavailable for pre-screening, the subject will enter the screening period and a biopsy will be performed to obtain the tumor sample for eligibility.
5. All laboratory results should be available and reviewed by the investigator before starting zolbetuximab study treatment.
6. Prophylactic antiemetics and, if needed, other premedications should be given according to institutional standards and the respective product package insert(s). Antiemetic premedication should be given at least 30 minutes prior to each dose of zolbetuximab. It is recommended that the prophylactic antiemetic regimen include the following agents: NK-1 receptor blockers and 5-HT3 receptor blockers.
7. Zolbetuximab will be administered as a single agent 2-hour IV infusion every 3 weeks. Zolbetuximab will be administered with a loading dose of 800 mg/m² BSA followed by 600 mg/m² BSA from cycle 2 onwards as IV infusion. Each zolbetuximab cycle is 3 weeks. Zolbetuximab IV infusion may be interrupted or slowed down to manage toxicity.
8. Post-Infusion Observation Period: Following the subject's first dose of zolbetuximab, the subject must remain at the site facility for 2 hours post infusion. If AEs are observed during this time, infusion time should be extended and subjects should continue to be observed for 2 hours post infusion. If the subject does not develop any AEs, the subject should be observed for 1 hour post infusion for their subsequent zolbetuximab infusions.
9. Physical examination and other evaluations include height (at screening only), weight and ECOG performance status. The physical exam only needs to be repeated on C1D1 if clinically significant changes from screening (in the opinion of the investigator) are observed. Target (symptom-driven) physical exams should be conducted every 3 weeks on zolbetuximab visit days.
10. Vital signs (pulse, blood pressure, temperature) should be taken at the following time points:
    a. Predose on dosing days
    b. C1D1 and C1D22: Every 15 (± 5) minutes during the zolbetuximab infusion.
    c. Subsequent zolbetuximab infusions: Every 30 (± 10) minutes if the subject did not develop any AEs during the Post-Infusion Observation Period of cycle 1
    d. Post-treatment observation period: 30 and 60 (± 10) minutes post infusion
11. See [Section 5.4.3] for list of laboratory assessments. Laboratory tests must be sent to the central laboratory for analysis. Central laboratory results must be used to confirm eligibility. The screening labs used to determine eligibility should be collected within 14 days prior to C1D1. In situations where central laboratory results are outside of the permitted range, the investigator may opt to retest the subject and subsequent within-range screening results may be used to confirm eligibility. In case of multiple laboratory data within the Screening period, the most recent central laboratory data should be used to confirm eligibility. Laboratory test results will be reviewed by the investigator prior to any study treatment. Local laboratory results may be used for treatment decisions; however, central laboratory samples must also be drawn per protocol and sent to the central laboratory. Holidays and weekends should be taken into account when scheduling these blood draws. Additional assessments may be done centrally or locally to monitor AEs or as clinically indicated. Clinical significance of out-of-range laboratory findings is to be determined and documented by the investigator/subinvestigator who is a qualified physician. CT scans and MRI conducted as part of a subject's routine clinical management (i.e., standard of care) obtained before signing the ICF may be utilized for screening or baseline purposes, provided the procedures met the protocol-specified criteria and were performed within the Screening period.
12. Urinalysis: Urinalysis should be performed at screening and is to be repeated if clinically indicated after starting study treatment. Urinalysis should also be performed at the zolbetuximab study drug discontinuation visit and the 30-day follow-up visit. Urinalysis tests will be sent to a central laboratory for analysis.
13. Ongoing evaluation should be continued for subjects who are receiving therapeutic anticoagulation according to local standard of care.
14. Serum Pregnancy Test: Will be collected for female subjects of childbearing potential only. Serum pregnancy tests are to be completed at screening. Subjects with elevated serum βHCG during screening and a demonstrated non-pregnant status through additional testing are eligible. Central laboratory must be used to confirm eligibility.
15. Urine Pregnancy Test: For female subjects of childbearing potential only. Urine pregnancy tests are to be completed at every visit prior to zolbetuximab administration. Urine pregnancy can be confirmed at a local laboratory.

Footnotes continued on next page

16. Samples should be collected predose.
17. For subjects who signed a separate ICF, an optional whole blood sample for PGx analysis will be collected at C1D1.
18. A single 12-lead ECG will be performed at screening, study drug discontinuation, 30-day follow-up visit and if clinically indicated.
19. Imaging will be evaluated at screening and every 6 (± 1) weeks counting from C1D1 for the first 24 weeks and then every 12 (± 2) weeks thereafter. Imaging assessment completed prior to consent, but within 28 days of first dose of study drug, may be used for screening. Imaging will include CT scans with contrast of the thorax, abdomen, and pelvis (if CT scan is medically not feasible with contrast, MRI may be used for imaging). Bone scans (or focal X-ray) or brain imaging should be performed if metastatic disease is suspected. Disease must be evident by radiology; measurable lesions only. Same mode of imaging should be utilized throughout the study unless medical necessity requires change. CT scan performed with PET scan can be used if it is of quality which allows accurate tumor measurement. Progression and tumor responses will be evaluated by investigator per RECIST 1.1. All imaging will be sent to a central independent radiographic review within 7 days.
20. HRQoL questionnaires and HRU are to be administered on zolbetuximab visit days before any drug treatment (or up to 48 hours prior to treatment) or other scheduled assessments are conducted and before the disease status is discussed with the subject. HRQoL will be measured by QLQ-C30, OG-25, GP and the EQ-5D questionnaires. The HRU questionnaire will not be administered at the screening visit. Questionnaires should only be administered on days when the subject receives zolbetuximab treatment.
21. Cohort 1A Only: Subjects are required to provide an additional tumor specimen collected within 3 months prior to the first dose of study treatment. There should be sufficient tumor tissue from the specimen used to send to the central laboratory for testing. If the specimen is insufficient or unavailable, a biopsy may be performed to obtain tumor. Ensure that subject meets all other study eligibility criteria prior to performing biopsy (as applicable). A minimum of 1 FFPE tumor tissue block (preferred) OR a minimum of 15 FFPE unstained sections are required. If slides are submitted, the slides should be freshly cut from the FFPE block within the time frame described in the laboratory manual. If available, the provision of additional fresh/frozen tumor samples is strongly encouraged. If ≥15 slides cannot be provided, the sponsor should be contacted for further guidance.
22. Cohort 1A Only: Subjects are required to provide an on-treatment tumor specimen collected ± 15 days of the C3D1 visit. A minimum of 1 FFPE tumor tissue block (preferred) OR a minimum of 15 FFPE unstained sections are required. If slides are submitted, the slides should be freshly cut from the FFPE block within the time frame described in the laboratory manual. If available, the provision of additional fresh/frozen tumor samples is strongly encouraged.
    If ≥15 slides cannot be provided, the sponsor should be contacted for further guidance.
23. For subjects who signed a separate ICF, an optional post-progression tumor sample for exploratory biomarker analysis should be collected following local or central confirmation of disease progression and prior to commencement of subsequent anti-cancer therapy.
24. Concomitant medications will be collected from the time of main study informed consent through 90 days following the last dose of study drug.
25. AEs/SAEs (regardless of causality) will be collected from the time of informed consent through 90 days following the last dose of study drug.
26. Study Drug Discontinuation: Visit will occur within 7 days after the last dose or decision by the investigator to discontinue subject from treatment.
27. Zolbetuximab 30-day Safety Follow-up Visit should occur 30 days (+ 7) after the last dose of zolbetuximab.
28. Zolbetuximab 90-day Safety Follow-up Visit should occur 90 days (+ 7) after the last dose of zolbetuximab.
29. Post-treatment Follow-up: if a subject discontinues study drug prior to radiographic or clinical disease progression as confirmed by the independent central reader, the subject should enter the post-treatment follow-up period and continue to undergo imaging assessments until progression is documented per the investigator, or the subject starts another cancer treatment, whichever occurs earlier.
30. Survival follow-up by telephone contact is to be performed every 12 weeks ± 2 weeks for all subjects with proven disease progression or who left the study due to any other reason than death or withdrawal of consent. Subjects will be followed to collect survival status until subject death, withdrawal of consent, or study closure. Additional follow-up contacts may be required per sponsor request for analysis purposes.

Table 2 Cohort 1A – Zolbetuximab Pharmacokinetic, Immunogenicity and Triplicate Electrocardiogram Schedule

**Study Day (1 cycle = 3 Weeks)**		**Time (Relative to Dosing in each cycle)**	**Zolbetuximab Pharmacokinetics** [a,b]	**Zolbetuximab Immunogenicity** [c]	**Triplicate ECG** [d]
Cycle 1	Day 1	Predose (0 hour)	X	X	X
		EOI	X		X
		0.5 hour after EOI	X		
		3 hours after EOI	X		
		6 hours after EOI	X		
	Day 2	24 hours after EOI	X		X
	Day 4	72 hours after EOI	X		
	Day 8	168 hours after EOI	X		
	Day 15	336 hours after EOI	X		
Cycle 2	Day 1	Predose (504 hours after C1D1 EOI)	X	X	
Cycle 3	Day 1	Predose (0 hour)	X	X	X
		EOI	X		X
		0.5 hour after EOI	X		
		3 hours after EOI	X		
		6 hours after EOI	X		
	Day 2	24 hours after EOI	X		
	Day 4	72 hours after EOI	X		
	Day 8	168 hours after EOI	X		
	Day 15	336 hours after EOI	X		
Cycle 4	Day 1	Predose (504 hours after C3D1 EOI)	X		
Cycles 5 & 9	Day 1	Predose (0 hour)	X	X	X
		EOI	X		X
Cycles 13 & 17	Day 1	Predose (0 hour)	X	X	
30-day Safety Follow-up Visit			X	X	
90-day Follow-up Visit			X	X	

C1D1: cycle 1 day 1; ECG: electrocardiogram; EOI: end of infusion

Footnotes continued on next page

a. Pharmacokinetic sampling windows are as below. The date and time of each sample collection will be recorded to the nearest minute.
   - Predose: within 60 minutes prior to dosing
   - End of infusion: within 10 minutes after the end of the infusion
   - Samples at 0.5 to 6 hours: ± 10 minutes

b. Unscheduled pharmacokinetic blood samples may be taken at any time during the study to evaluate drug exposure following a safety event.

c. Immunogenicity sampling window: Predose: within 60 minutes prior to dosing.

d. Prior to performing triplicate ECG, subjects should rest in supine position for 10 minutes and their ECGs should be recorded in triplicate with 2 minutes apart per time point. When collected on the same day, ECG should be collected prior to pharmacokinetic samples. ECGs will be read centrally.

Table 3 Schedule of Assessments for Cohort 2 Cycle 1

VISIT	Pre-Screening[1]	Screening[2]	Combination Treatment Period mFOLFOX6 + Zolbetuximab cycle 1<br>cycle = 42 days						Zolbetuximab Study Drug Discontinuation Visit[25]	mFOLFOX6 Study Drug Discontinuation Visit[25]	Zolbetuximab 30 Day Safety Follow-up Visit[26]	mFOLFOX6 30 Day Safety Follow-up Phone Call[27]	Zolbetuximab 90 Day Follow-up Visit[28]	mFOLFOX6 90 Day Follow-up Phone Call[29]	Post-treatment Follow-up[30]
**Day**			**1**	**3**	**8**	**15**	**22**	**29**							
Visit Window (business days)		-28 to -1	0	+ 2	+ 2	+ 2	+ 2	+ 2	+ 7	+ 7	+ 7	+ 7	+ 7	+ 7	± 14
Pre-Screening ICF	X														
Tumor Sample for Eligibility[1]	X														
Archival Tumor Sample-HER2[1]	X														
Main Study ICF		X													
Tumor Biopsy[3]		X													
Medical and Disease History		X													
Confirmation of Eligibility		X	X												
Cohort Assignment[4]			X												
**Study Drug Administration**															
Antiemetic Pretreatment[5]			X	X		X	X	X							
Zolbetuximab[6]				X			X								
Post-infusion Observation Period[7]				X			X								
mFOLFOX6 [8]			X			X		X							
**Physical Examination/Assessments**															
Physical Examination[9]		X		X			X		X	X	X				
Weight[9]		X		X			X		X	X	X				
ECOG Performance Status[9]		X		X			X		X	X	X				
Vital Signs [10]		X		X			X		X	X	X				
**Laboratory Assessments**															
Biochemistry[11]		X	X	X	X	X	X	X	X	X	X				
Hematology[11]		X	X	X	X	X	X	X	X	X	X				

Table continued on next page

VISIT	Pre-Screening[1]	Screening[2]	Combination Treatment Period mFOLFOX6 + Zolbetuximab cycle 1<br><br>cycle = 42 days						Zolbetuximab Study Drug Discontinuation Visit[25]	mFOLFOX6 Study Drug Discontinuation Visit[25]	Zolbetuximab 30 Day Safety Follow-up Visit[26]	mFOLFOX6 30 Day Safety Follow-up Phone Call[27]	Zolbetuximab 90 Day Follow-up Visit[28]	mFOLFOX6 90 Day Follow-up Phone Call[29]	Post-treatment Follow-up[30]
Day			1	3	8	15	22	29							
Visit Window (business days)		-28 to -1	0	+ 2	+ 2	+ 2	+ 2	+ 2	+ 7	+ 7	+ 7	+ 7	+ 7	+ 7	± 14
Urinalysis[12]		X	If clinically indicated						X	X	X				
DPD testing per local requirements		X													
Cytokine/Chemokine and/or Tryptase			If clinically indicated												
TSH and T4		X	If clinically indicated												
PT, PTT and INR[13]		X	If clinically indicated												
Serum Pregnancy Test[14]		X													
Urine Pregnancy Test[15]				X			X		X	X	X				
Pharmacokinetics of Zolbetuximab			See Table 5 below for pharmacokinetic collection schedule								X		X		
Pharmacokinetics of mFOLFOX6			See Table 6 below for pharmacokinetic collection schedule												
Anti-Drug Antibodies (Immunogenicity)			See Table 5 below for sample collection schedule								X		X		
Genetic Immune Polymorphisms – (Whole Blood)			X												
Exploratory Biomarkers (Serum)[16]			X				X		X						
Exploratory Biomarkers (Plasma)[16]			X				X		X						
Immune Cell Subsets (Whole Blood)[16]			X				X		X						
Cryopreserved PBMC[16]			X				X		X						
Whole Blood Sample for PGx (optional)[17]			X												
**Cardiac Safety**															
12-lead ECG[18]		X	X			X		X	X		X				
**Radiology**															
Image Assessment[19]		X	Every 9 (± 1) weeks from C1D1 for the first 54 weeks and then every 12 (± 2) weeks thereafter.												
Table continued on next page															

**VISIT**	**Pre-Screening**[1]	**Screening**[2]	**Combination Treatment Period mFOLFOX6 + Zolbetuximab cycle 1<br><br>cycle = 42 days**						**Zolbetuximab Study Drug Discontinuation Visit**[25]	**mFOLFOX6 Study Drug Discontinuation Visit**[25]	**Zolbetuximab 30 Day Safety Follow-up Visit**[26]	**mFOLFOX6 30 Day Safety Follow-up Phone Call**[27]	**Zolbetuximab 90 Day Follow-up Visit**[28]	**mFOLFOX6 90 Day Follow-up Phone Call**[29]	**Post-treatment Follow-up**[30]
**Day**			**1**	**3**	**8**	**15**	**22**	**29**							
Visit Window (business days)		-28 to -1	0	+ 2	+ 2	+ 2	+ 2	+ 2	+ 7	+ 7	+ 7	+ 7	+ 7	+ 7	± 14
**HRQoL**															
HRQoL[20]		X		X			X		X		X		X		
**Tissue Sample**															
Post-progression Tumor Sample (optional)[21]									X						
Baseline Tumor Sample[22]		X													
**Safety Assessment**															
Concomitant Medication[23]		X	X	X	X	X	X	X	X	X	X	X	X	X	
AEs/SAEs[24]		X	X	X	X	X	X	X	X	X	X	X	X	X	

5-FU: fluorouracil; ADA: anti-drug antibody; AE: adverse event; βHCG: beta human chorionic gonadotropin; BSA: body surface area; C1D1: cycle 1 day 1; CLDN: claudin; CT: computerized tomography; DPD: dihydropyrimidine dehydrogenase; eCRF: electronic case report form; ECG: electrocardiogram; ECOG: Eastern Cooperative Oncology Group; EQ-5D: EuroQOL five dimensions questionnaire; FFPE: formalin-fixed paraffin-embedded; GP: Global Pain; HER2: human epidermal growth factor receptor 2; HRQoL: health-related quality of life; HRU: Health Resource Utilization; ICF: informed consent form; INR: international normalized ratio; IRR: infusion related reaction; IV: intravenous; MRI: magnetic resonance imaging; OG-25: Oesophago-Gastric Module (EORTC QLQ-OG-25); ORR: objective response rate; QLQ-C30: Quality of Life Questionnaire - Core Questionnaire; PBMC: peripheral blood mononuclear cells; PET: positron emission tomography; PGx: pharmacogenomics; PFS: progression-free survival; PGx: pharmacogenomics; PT: prothrombin time; PTT: partial thromboplastin time; SAE: serious adverse event; T4: thyroxine; TSH: thyroid-stimulating hormone

Laboratory tests will be performed predose according to the Schedule of Assessments and sent to a central laboratory for analysis. In case of multiple laboratory data within this period, the most recent data should be used.

Footnotes continued on next page

1. Pre-Screening: FFPE tumor specimens will be collected for central pre-screening tests to determine CLDN18.2 and HER2 status (if necessary). There should be sufficient tumor tissue in the specimen used to send to the central laboratory for testing. Archival tumor tissue is preferred. A minimum of 1 FFPE tumor tissue block (preferred) OR a minimum of 15 FFPE unstained sections are required. If slides are submitted, the slides should be freshly cut from the FFPE block within the time frame described in the laboratory manual. If archival tumor tissue is insufficient or unavailable for pre-screening, the subject will enter the screening period and a biopsy will be performed to obtain the tumor sample for eligibility. If ≥ 15 slides cannot be provided, the sponsor should be contacted for further guidance.
   NOTE: If local HER2 results are available, a minimum of 9 slides are required. If local HER2 results are unavailable, follow guidance above.
2. Screening period is 28 days. Subjects may be rescreened once during the screening period. Any procedures that fall outside of the screening period must be repeated. If more than 1 assessment is taken during the screening period, the assessment closest to enrollment date should be used for eligibility.
3. If archival tumor tissue is insufficient or unavailable for pre-screening, the subject will enter the screening period and a biopsy will be performed to obtain the tumor sample for eligibility.
4. All laboratory results should be available and reviewed by the investigator before starting any study treatment (zolbetuximab, mFOLFOX6).
5. Prophylactic antiemetics and, if needed, other premedications should be given according to institutional standards and the respective product package insert(s). Antiemetic premedication should be given at least 30 minutes prior to each dose of zolbetuximab and mFOLFOX6. It is recommended that the prophylactic antiemetic regimen include the following agents: NK-1 receptor blockers and 5-HT3 receptor blockers. On days when subjects receive both zolbetuximab and mFOLFOX6, antiemetic premedication will be given prior to zolbetuximab administration.
6. Zolbetuximab will be administered with a loading dose of 800 mg/m² BSA as a 2-hour IV infusion on cycle 1 day 3 to allow for the mFOLFOX6 pharmacokinetic collection. Zolbetuximab IV infusion may be interrupted or slowed down to manage toxicity.
7. Post-Infusion Observation Period: Following the subject's first dose of zolbetuximab on C1D3, the subject must remain at the site facility for 2 hours post infusion. If AEs are observed during this time, infusion time should be extended and subjects should continue to be observed for 2 hours post infusion.
8. mFOLFOX6 will be administered every 2 weeks. If both zolbetuximab and mFOLFOX6 are to be administered during the same visit, zolbetuximab should be administered prior to mFOLFOX6.
9. Physical examination and other evaluations include height (at screening only), weight and ECOG performance status. The physical exam only needs to be repeated on C1D1 if clinically significant changes from screening (in the opinion of the investigator) are observed. Targeted (symptom-driven) physical exams should be conducted every 3 weeks on zolbetuximab visit days.
10. Vital signs (pulse, blood pressure, temperature) should be taken at the following time points:
    a. Predose on dosing days for zolbetuximab
    b. C1D3 and C1D22: Every 15 (± 5) minutes during the zolbetuximab infusion.
    c. Post-treatment observation period: 30 and 60 (± 10) minutes post infusion

Footnotes continued on next page

11. See [Section 5.4.3] for list of laboratory assessments. Laboratory tests must be sent to the central laboratory for analysis. Central laboratory results must be used to confirm eligibility. The screening labs used to determine eligibility should be collected within 14 days prior to C1D1. In situations where central laboratory results are outside of the permitted range, the investigator may opt to retest the subject and subsequent within-range screening results may be used to confirm eligibility. In case of multiple laboratory data within the Screening period, the most recent central laboratory data should be used to confirm eligibility. Laboratory test results will be reviewed by the investigator prior to any study treatment. Local laboratory results may be used for treatment decisions; however, central laboratory samples must also be drawn per protocol and sent to the central laboratory. Holidays and weekends should be taken into account when scheduling these blood draws. Additional assessments may be done centrally or locally to monitor AEs or as clinically indicated. Clinical significance of out-of-range laboratory findings is to be determined and documented by the investigator/subinvestigator who is a qualified physician. CT scans and MRI conducted as part of a subject's routine clinical management (i.e., standard of care) obtained before signing the ICF may be utilized for screening or baseline purposes, provided the procedures met the protocol-specified criteria and were performed within the Screening period.
12. Urinalysis: Urinalysis should be performed at screening and is to be repeated if clinically indicated after starting study treatment. Urinalysis should also be performed for the zolbetuximab and mFOLFOX6 study drug discontinuation visits and at the zolbetuximab 30-day follow-up visit. Urinalysis tests will be sent to a central laboratory for analysis.
13. Ongoing evaluation should be continued for subjects who are receiving therapeutic anticoagulation according to local standard of care.
14. Serum Pregnancy Test: Will be collected for female subjects of childbearing potential only. Serum pregnancy tests are to be completed at screening. Subjects with elevated serum βHCG during screening and a demonstrated non-pregnant status through additional testing are eligible. Central laboratory must be used to confirm eligibility.
15. Urine Pregnancy Test: For female subjects of childbearing potential only. Urine pregnancy tests are to be completed at every visit prior to zolbetuximab administration. Urine pregnancy can be confirmed at a local laboratory.
16. Biomarker (serum, plasma, whole blood and cryopreserved PBMCs) samples should be taken within 48 hours prior to dosing:
    a. C1D1: predose
    b. C1D22: predose
    c. Zolbetuximab study drug discontinuation visit
17. For subjects who signed a separate ICF, an optional whole blood sample for PGx for exploratory biomarker analysis should be collected within 48 hours prior to dosing.
18. A single ECG will be performed at the following time points:
    - Screening
    - Up to 48 hours prior to every oxaliplatin infusion (before any antiemetic treatment)†
    - Up to 6 hours following completion of every oxaliplatin infusion†
    - Zolbetuximab study drug discontinuation
    - Zolbetuximab 30-day follow-up visit
    - If clinically indicated

    †Local read only – do not transmit to central ECG laboratory.

Footnotes continued on next page

19. Imaging assessments to be collected at screening and every 9 (± 1) weeks counting from C1D1 for the first 54 weeks, and then every 12 (± 2) weeks thereafter. Imaging assessments completed prior to consent, but within 28 days of first dose of study drug, may be used for screening. Imaging will include CT scans with contrast of the thorax, abdomen, and pelvis (if CT scan is medically not feasibly with contrast, MRI may be used for imaging). Bone scans (or focal X-ray) or brain imaging should be performed if metastatic disease is suspected. Disease must be evident by radiology; measurable lesions only. Same mode of imaging should be utilized throughout the study unless medical necessity requires change. CT scan performed with PET scan can be used if it is of quality, which allows accurate tumor measurement. Progression and tumor responses will be evaluated by investigator per RECIST 1.1. All imaging will be sent to a central independent radiographic review within 7 days.
20. HRQoL questionnaires and HRU are to be administered on zolbetuximab visit days before any drug treatment (or up to 48 hours prior to treatment) or other scheduled assessments are conducted and before the disease status is discussed with the subject. HRQoL will be measured by QLQ-C30, OG-25, GP and the EQ-5D questionnaires. The HRU questionnaire will not be administered at the screening visit. Questionnaires should only be administered on days when the subject receives zolbetuximab treatment.
21. For subjects who signed a separate ICF, an optional post-progression tumor sample for exploratory biomarker analysis should be collected following confirmation of disease progression and prior to commencement of subsequent anti-cancer therapy.
22. Subjects are required to provide a tumor specimen collected within 3 months prior to the first dose of study treatment. There should be sufficient tumor tissue from the specimen used to send to the central laboratory for testing. If the specimen is insufficient or unavailable, a biopsy may be performed to obtain tumor tissue. Ensure that subject meets all other study eligibility criteria prior to performing the biopsy (as applicable). A minimum of 1 FFPE tumor tissue block (preferred) OR a minimum of 15 FFPE unstained sections are required. If slides are submitted, the slides should be freshly cut from the FFPE block within the time frame described in the laboratory manual. If available, the provision of additional fresh/frozen tumor samples is strongly encouraged. If ≥15 slides cannot be provided, the sponsor should be contacted for further guidance.
23. Concomitant medications will be collected from the time of main study informed consent through 90 days following the last dose of study drug.
24. AEs and SAEs (regardless of causality) will be collected from the time of informed consent through 90 days following the last dose of study drug.
25. Study Drug Discontinuation: Visit will occur within 7 days after the last dose or decision by the investigator to discontinue subject from treatment.
26. Zolbetuximab 30-day Safety Follow-up Visit should occur 30 days (+ 7) after last dose of zolbetuximab.
27. mFOLFOX6 30-day Safety Follow-up phone call should occur 30 days (+ 7) after last dose of mFOLFOX6 (all components).
28. Zolbetuximab 90-day Follow-up Visit should occur 90 days (+ 7) after last dose of zolbetuximab.
29. mFOLFOX6 90-day Safety Follow-up phone call should occur 90 days (+ 7) after the last dose of mFOLFOX6 (all components).
30. Post-treatment Follow-up: if a subject discontinues study drug prior to radiographic or clinical disease progression as confirmed by the independent central reader, the subject should enter the post-treatment follow-up period and continue to undergo imaging assessments until progression is documented per the investigator, or the subject starts another cancer treatment, whichever occurs earlier.

Table 4 Schedule of Assessments for Cohort 2 Cycles ≥ 2

VISIT	Treatment Period (cycle = 42 days)								Zolbetuximab Study Drug Discontinuation Visit[20]	mFOLFOX6 Study Drug Discontinuation Visit[20]	Zolbetuximab 30-Day Safety Follow-up Visit[21]	mFOLFOX6 30-Day Safety Follow-up Phone Call[22]	Zolbetuximab 90-Day Follow-up Visit[23]	mFOLFOX6 90-Day Safety Follow-up Phone Call[24]	Post-treatment Follow-up[25]
	Cycles 2 to 4<br><br>Combination Treatment Period<br>mFOLFOX6 + Zolbetuximab				Cycles ≥ 5<br><br>Combination Treatment Period<br>Zolbetuximab<br>+<br>Leucovorin or folinic acid + 5-FU										
**Day**	**1**	**15**	**22**	**29**	**1**	**15**	**22**	**29**							
Visit Window (business days)	0	+ 2	+ 2	+ 2	0	+ 2	+ 2	+ 2	+ 7	+ 7	± 7	± 7	+ 7	+ 7	± 14
**Study Drug Administration**															
Antiemetic Pretreatment[1]	X	X	X	X	X	X	X	X							
Zolbetuximab [2]	X		X		X		X								
Post-Infusion Observation Period[3]	X		X		X		X								
mFOLFOX6 [4]	X	X		X	X	X		X							
**Physical Examination/Assessments**															
Physical Examination[5]	X		X		X		X		X	X	X				
Weight [5]	X		X		X		X		X	X	X				
ECOG Performance Status[5]	X		X		X		X		X	X	X				
Vital Signs [6]	X		X		X		X		X	X	X				
**Laboratory Assessments**															
Biochemistry[7]	X	X	X	X	X	X	X	X	X	X	X				
Hematology[7]	X	X	X	X	X	X	X	X	X	X	X				
Urinalysis[8]	If clinically indicated								X	X	X				
Cytokine/Chemokine and/or Tryptase[9]	If clinically indicated														
TSH and T4	If clinically indicated														
PT, PTT and INR[10]	If clinically indicated														
Urine Pregnancy Test[11]	X		X		X		X		X	X	X				

Table continued on next page

VISIT	Treatment Period (cycle = 42 days)								Zolbetuximab Study Drug Discontinuation Visit[20]	mFOLFOX6 Study Drug Discontinuation Visit[20]	Zolbetuximab 30-Day Safety Follow-up Visit[21]	mFOLFOX6 30-Day Safety Follow-up Phone Call[22]	Zolbetuximab 90-Day Follow-up Visit[23]	mFOLFOX6 90-Day Safety Follow-up Phone Call[24]	Post-treatment Follow-up[25]
	Cycles 2 to 4<br>Combination Treatment Period mFOLFOX6 + Zolbetuximab				Cycles ≥ 5<br>Combination Treatment Period Zolbetuximab + Leucovorin or folinic acid + 5-FU										
**Day**	**1**	**15**	**22**	**29**	**1**	**15**	**22**	**29**							
Visit Window (business days)	0	+ 2	+ 2	+ 2	0	+ 2	+ 2	+ 2	+ 7	+ 7	± 7	± 7	+ 7	+ 7	± 14
Pharmacokinetics of Zolbetuximab	See Table 5 below for pharmacokinetic collection schedule										X		X		
Pharmacokinetics of mFOLFOX6	See Table 6 below for pharmacokinetic collection schedule														
Anti-Drug Antibodies (immunogenicity)	See Table 5 below for sample collection schedule										X		X		
Exploratory Biomarkers (Serum)[12]	X		X						X						
Exploratory Biomarkers (Plasma)[12]	X		X						X						
Immune Cell Subsets (Whole Blood)[12]	X		X						X						
Cryopreserved PBMC[12]	X		X						X						
**Cardiac Safety**															
12-lead ECG[13]	X	X		X	If clinically indicated				X		X				
**Radiology**															
Image Assessment[14]	Every 9 (± 1) weeks from C1D1 for the first 54 weeks and then every 12 (± 2) weeks thereafter.														
**HRQoL**															
HRQoL[15]	X		X		X		X		X		X		X		
Table continued on next page															

VISIT	Treatment Period (cycle = 42 days)								Zolbetuximab Study Drug Discontinuation Visit[20]	mFOLFOX6 Study Drug Discontinuation Visit[20]	Zolbetuximab 30-Day Safety Follow-up Visit[21]	mFOLFOX6 30-Day Safety Follow-up Phone Call[22]	Zolbetuximab 90-Day Follow-up Visit[23]	mFOLFOX6 90-Day Safety Follow-up Phone Call[24]	Post-treatment Follow-up[25]
	Cycles 2 to 4<br>Combination Treatment Period mFOLFOX6 + Zolbetuximab				Cycles ≥ 5<br>Combination Treatment Period Zolbetuximab + Leucovorin or folinic acid + 5-FU										
**Day**	**1**	**15**	**22**	**29**	**1**	**15**	**22**	**29**							
Visit Window (business days)	0	+ 2	+ 2	+ 2	0	+ 2	+ 2	+ 2	+ 7	+ 7	± 7	± 7	+ 7	+ 7	± 14
**Tissue Samples**															
Post-progression Tumor Sample (optional)[16]									X						
On-Treatment Biopsy[17]	X														
**Safety Assessment**															
Concomitant Medication[18]	X	X	X	X	X	X	X	X	X	X	X	X	X	X	
AEs/SAEs[19]	X	X	X	X	X	X	X	X	X	X	X	X	X	X	

5-FU: fluorouracil; ADA: anti-drug antibody; AE: adverse event; BSA: body surface area; C: cycle; CLDN: claudin; CT: computerized tomography; D: day; eCRF: electronic case report form; ECG: electrocardiogram; ECOG: Eastern Cooperative Oncology Group; FFPE: formalin-fixed paraffin-embedded; GP: Global Pain; HER2: human epidermal growth factor receptor 2; HRQoL: health-related quality of life; HRU: Health Resource Utilization; ICF: informed consent form; INR: international normalized ratio; IRR: infusion related reaction; IV: intravenous; MRI: magnetic resonance imaging; ORR: objective response rate; PBMC: peripheral blood mononuclear cells; PET: positron emission tomography; PFS: progression-free survival; PGx: pharmacogenomics; PT: prothrombin time; PTT: partial thromboplastin time; SAE: serious adverse event; T4: thyroxine; TSH: thyroid-stimulating hormone.

Laboratory tests will be performed predose according to the Schedule of Assessments and sent to a central laboratory for analysis. In case of multiple laboratory data within this period, the most recent data should be used.

1. Prophylactic antiemetics and, if needed, other premedications should be given according to institutional standards and the respective product package insert(s). Antiemetic premedication should be given at least 30 minutes prior to each dose of zolbetuximab and mFOLFOX6. It is recommended that the prophylactic antiemetic regimen include the following agents: NK-1 receptor blockers and 5-HT3 receptor blockers. On days when subjects receive both zolbetuximab and mFOLFOX6, antiemetic premedication will be given prior to zolbetuximab administration.
2. Zolbetuximab will be administered as a minimum 2-hour IV infusion. Zolbetuximab IV infusion may be interrupted or slowed down to manage toxicity.
3. Post-Infusion Observation Period: If the subject does not develop any AEs, the subject should be observed for 1 hour post infusion for their subsequent zolbetuximab infusions. If AEs are observed, infusion time should be extended and subjects should continue to be observed for 2 hours post infusion.

Footnotes continued on next page

4. mFOLFOX6 will be administered every 2 weeks. If both zolbetuximab and mFOLFOX6 are to be administered during the same visit, zolbetuximab should be administered prior to mFOLFOX6.
5. Physical examination and other evaluations include height (at screening only), weight and ECOG performance status. The physical exam only needs to be repeated on C1D1 if clinically significant changes from screening (in the opinion of the investigator) are observed. Target (symptom-driven) physical exams should be conducted every 3 weeks on zolbetuximab visit days.
6. Vital signs (pulse, blood pressure, temperature) should be taken at the following time points:
   a. Predose on dosing days
   b. Subsequent zolbetuximab infusions: Every 30 (± 10) minutes if the subject did not develop any AEs during the Post-Infusion Observation Period of cycle 1.
   c. Post-treatment observation period: 30 and 60 (± 10) minutes post infusion.
7. See [Section 5.4.3] for list of laboratory assessments. Laboratory tests must be sent to the central laboratory for analysis. Central laboratory results must be used to confirm eligibility. The screening labs used to determine eligibility should be collected within 14 days prior to C1D1. In situations where central laboratory results are outside of the permitted range, the investigator may opt to retest the subject and subsequent within-range screening results may be used to confirm eligibility. In case of multiple laboratory data within the Screening period, the most recent central laboratory data should be used to confirm eligibility. Laboratory test results will be reviewed by the investigator prior to any study treatment. Local laboratory results may be used for treatment decisions; however, central laboratory samples must also be drawn per protocol and sent to the central laboratory. Central and local labs may be collected up to 48 hours prior to study treatment. Holidays and weekends should be taken into account when scheduling these blood draws. Additional assessments may be done centrally or locally to monitor AEs or as clinically indicated. Clinical significance of out-of-range laboratory findings is to be determined and documented by the investigator/subinvestigator who is a qualified physician. CT scans and MRI conducted as part of a subject's routine clinical management (i.e., standard of care) obtained before signing the ICF may be utilized for screening or baseline purposes, provided the procedures met the protocol-specified criteria and were performed within the Screening period.
8. Urinalysis: Urinalysis should be performed at screening and is to be repeated if clinically indicated after starting study treatment. Urinalysis should also be performed at the zolbetuximab and mFOLFOX6 study discontinuation visits and at the zolbetuximab 30-day follow-up visit. Urinalysis tests will be sent to a central laboratory for analysis.
9. Cytokines/chemokines and/or tryptase need to be collected centrally.
10. Ongoing evaluation should be continued for subjects who are receiving therapeutic anticoagulation according to local standard of care.
11. Urine Pregnancy Test: For female subjects of childbearing potential only. Urine pregnancy tests are to be completed prior to zolbetuximab administration. Urine pregnancy tests can be confirmed at a local laboratory.
12. Biomarker (serum, plasma, whole blood and cryopreserved PBMCs) samples should be taken within 48 hours prior to dosing.
    a. C2D1: predose
    b. C2D22: predose
    c. C3D22: predose
    d. C4D22: predose
    e. Zolbetuximab study drug discontinuation visit

Footnotes continued on next page

13. Single ECG will be performed at the following time points:
    - Screening
    - Up to 48 hours prior to every oxaliplatin infusion (before any antiemetic treatment)†
    - Up to 6 hours following completion of every oxaliplatin infusion†
    - Zolbetuximab study drug discontinuation visit
    - Zolbetuximab 30-day follow-up visit
    - If clinically indicated

    †Local read only – do not transmit to central ECG laboratory
14. Imaging assessments to be collected at screening and every 9 (± 1) weeks counting from C1D1 for the first 54 weeks, and then every 12 (± 2) weeks thereafter. Imaging assessments completed prior to consent, but within 28 days of first dose of study drug, may be used for screening. Imaging will include CT scans with contrast of the thorax, abdomen, and pelvis (if CT scan is medically not feasible with contrast, MRI may be used for imaging). Bone scans (or focal X-ray) or brain imaging should be performed if metastatic disease is suspected. Disease must be evident by radiology; measurable lesions only. Same mode of imaging should be utilized throughout the study unless medical necessity requires change. CT scan performed with PET scan can be used if it is of quality, which allows accurate tumor measurements. PFS and tumor responses will be evaluated by investigator per RECIST 1.1. All imaging will be sent to a central independent radiographic review within 7 days.
15. HRQoL questionnaires and HRU are to be administered on zolbetuximab visit days before any drug treatment (or up to 48 hours prior to treatment) or other scheduled assessments are conducted and before the disease status is discussed with the subject. HRQoL will be measured by Quality of Life Questionnaire – Core Questionnaire (QLQ-C30), Oesophago-Gastric Module (OG-25), Global Pain (GP) and the EuroQOL Five Dimensions (EQ-5D) questionnaires. The HRU questionnaire will not be administered at the screening visit. Questionnaires should only be administered on days when the subject receives zolbetuximab treatment.
16. For subjects who signed a separate ICF, an optional post-progression tumor sample for exploratory biomarker analysis should be collected following confirmation of disease progression and prior to commencement of subsequent anti-cancer therapy.
17. Subjects are required to provide an on-treatment tumor specimen collected at the C2D1 visit (± 15 days). A minimum of 1 FFPE tumor tissue block (preferred) OR a minimum of 15 FFPE unstained sections are required. If slides are submitted, the slides should be freshly cut from the FFPE block within the time frame described in the laboratory manual. If available, the provision of additional fresh/frozen tumor samples is strongly encouraged. If ≥ 15 slides cannot be provided, the sponsor should be contacted for further guidance.
18. Concomitant medications will be collected from the time of main study informed consent through 90 days following the last dose of study drug.
19. AEs and SAEs (regardless of causality) will be collected from the time of main informed consent through 90 days following the last dose of study drug.
20. Study Drug Discontinuation: Visit will occur within 7 days after the last dose or decision by the investigator to discontinue subject from treatment.
21. Zolbetuximab 30-day Safety Follow-up Visit should occur 30 days (± 7) after last dose of zolbetuximab.
22. mFOLFOX6 30-day Safety Follow-up Visit should occur 30 days (± 7) after last dose of mFOLFOX6 (all components).
23. Zolbetuximab 90-day Follow-up Visit should occur 90 days (+ 7) after last dose of zolbetuximab.
24. mFOLFOX6 90-day Safety Follow-up Visit should occur 90 days (+ 7) after the last dose of mFOLFOX6 (all components).
25. Post-treatment Follow-up: if a subject discontinues study drug prior to radiographic or clinical disease progression as confirmed by the independent central reader, the subject should enter the post-treatment follow-up period and continue to undergo imaging assessments until progression is documented per the investigator, or the subject starts another cancer treatment, whichever occurs earlier.

Table 5 Cohort 2 – Zolbetuximab Pharmacokinetic and Immunogenicity Schedule

Study Day (1 cycle = 42 Days)		Time (Relative to Dosing in each cycle)	Zolbetuximab Pharmacokinetics [1,2]	Zolbetuximab Immunogenicity [3]
Cycle 1	Day 3	Predose (0 hour)	X	X
		EOI	X	
		0.5 hour after EOI	X	
		3 hours after EOI	X	
		6 hours after EOI	X	
	Day 4	24 hours after EOI	X	
	Day 8	120 hours after EOI	X	
	Day 15	288 hours after EOI	X	
	Day 22	Predose (456 hours after C1D3 EOI)	X	X
Cycle 2	Day 1	Predose (0 hour)	X	X
		EOI	X	
		0.5 hour after EOI	X	
		3 hours after EOI	X	
		6 hours after EOI	X	
	Day 2	24 hours after EOI	X	
	Day 4	72 hours after EOI	X	
	Day 8	168 hours after EOI	X	
	Day 15	336 hours after EOI	X	
	Day 22	Predose (504 hours after C2D1 EOI)	X	
Cycles 3, 5, 7 & 9	Day 1	Predose (0 hour)	X	X
Zolbetuximab 30-day Safety Follow-up Visit			X	X
Zolbetuximab 90-day Follow-up Visit			X	X

C1D3: cycle 1 day 3; EOI: End of Infusion

1. Pharmacokinetic sampling windows are as below. The date and time of each sample collection will be recorded to the nearest minute.
   - Predose: within 60 minutes prior to dosing
   - End of infusion: within 10 minutes after the end of the infusion
   - Samples at 0.5 to 6 hours: ± 10 minutes
2. Unscheduled pharmacokinetic blood samples may be taken at any time during the study to evaluate drug exposure following a safety event.
3. Immunogenicity sampling window: Predose: within 60 minutes prior to dosing.

Table 6 Cohort 2 – mFOLFOX6 Pharmacokinetic Schedule

**Study Day (1 cycle = 42 Days)**		**Time (Relative to Dosing in each cycle)**	**5-FU Pharmacokinetics [1,2]**	**Oxaliplatin Pharmacokinetics [1,2]**
Cycle 1	Day 1	Predose (0 hour)	X	X
		EOI		X
		0.5 hour after start dosing	X	X
		1 hour after start dosing	X	X
		2 hours after start dosing	X	X
		5 hours after start dosing	X	X
	Day 2	24 hours after start dosing	X	X
	Day 3	48 hours after start dosing	X	
Cycle 2	Day 1	Predose (0 hour)	X	X
		EOI		X
		0.5 hour after start dosing	X	X
		1 hour after start dosing	X	X
		2 hours after start dosing	X	X
		5 hours after start dosing	X	X
	Day 2	24 hours after start dosing	X	X
	Day 3	48 hours after start dosing	X	

EOI: End of Infusion

1. "After start dosing" means:
    - 5-FU: after starting bolus dosing
    - Oxaliplatin: after start of infusion
2. Pharmacokinetic sampling windows are as below. The date and time of each sample collection will be recorded to the nearest minute.
    - Predose: within 60 minutes prior to dosing
    - End of infusion: within 10 minutes after the end of the infusion
    - Samples at 0.5 to 5 hours: ± 10 minutes

Table 7 Schedule of Assessments for Cohort 3A

VISIT	Pre-Screening[1]	Screening[2]	Combination Treatment Period Pembrolizumab + Zolbetuximab<br><br>cycle = 21 days				Zolbetuximab Study Drug Discontinuation Visit[29]	Pembrolizumab Study Drug Discontinuation Visit[29]	Zolbetuximab 30 Day Safety Follow-up Visit[30]	Pembrolizumab 30 Day Safety Follow-up Visit[31]	Zolbetuximab 90 Day Follow-up Visit[32]	Pembrolizumab 90 Day Follow-up Visit[33]
**Cycle[3]**			**1**	**2**	**3**	**≥ 4**						
**Day**			**1**	**22**	**43**	**64**						
Visit Window (business days)		-28 to -1	0	+ 2	+ 2	+ 2	+ 7	+ 7	± 7	± 7	+ 7	+ 7
Pre-Screening ICF	X											
Tumor Sample for Eligibility[1]	X											
Main Study ICF		X										
Tumor Biopsy[4]		X										
Medical and Disease History		X										
Confirmation of Eligibility		X	X									
Cohort Assignment[5]			X									
**Study Drug Administration**												
Antiemetic Pretreatment[6]			X	X	X	X						
Zolbetuximab[7]			X	X	X	X						
Post-infusion Observation Period[8]			X	X	X	X						
Pembrolizumab [9]			X	X	X	X						
**Physical Examination/Assessments**												
Physical Examination[10]		X	X	X	X	X	X	X	X			
Weight[10]		X	X	X	X	X	X	X	X			
ECOG Performance Status[10]		X	X	X	X	X	X	X	X			
Vital Signs [11]		X	X	X	X	X	X	X	X			
Table continued on next page												

VISIT	Pre-Screening[1]	Screening[2]	Combination Treatment Period Pembrolizumab + Zolbetuximab<br>cycle = 21 days				Zolbetuximab Study Drug Discontinuation Visit[29]	Pembrolizumab Study Drug Discontinuation Visit[29]	Zolbetuximab 30 Day Safety Follow-up Visit[30]	Pembrolizumab 30 Day Safety Follow-up Visit[31]	Zolbetuximab 90 Day Follow-up Visit[32]	Pembrolizumab 90 Day Follow-up Visit[33]
**Cycle[3]**			**1**	**2**	**3**	**≥ 4**						
**Day**			**1**	**22**	**43**	**64**						
Visit Window (business days)		-28 to -1	0	+ 2	+ 2	+ 2	+ 7	+ 7	± 7	± 7	+ 7	+ 7
**Laboratory Assessments**												
Biochemistry[12]		X	X	X	X	X	X	X	X		X	
Hematology[12]		X	X	X	X	X	X	X	X		X	
Urinalysis[13]		X	If clinically indicated				X	X	X			
Cytokine/Chemokine and/or Tryptase[14]			If clinically indicated									
TSH and T4[15]		X	X	X		X		X		X		
PT, PTT and INR[16]		X	If clinically indicated									
Serum Pregnancy Test[17]		X	If clinically indicated									
Urine Pregnancy Test[18]			X	X	X	X	X	X	X			
Pharmacokinetics of Zolbetuximab			See Table 8 below for pharmacokinetic collection schedule						X		X	
Pharmacokinetics of Pembrolizumab			See Table 8 below for pharmacokinetic collection schedule							X		X
Anti-Drug Antibodies (Immunogenicity)			See Table 8 below for sample collection schedule						X		X	
Tumor Markers[19a]			X	X	X	X	X					
Exploratory Biomarkers (Serum)[19b]			X	X	X	X	X					
Exploratory Biomarkers (Plasma)[19b]			X	X	X	X	X					
Immune Cell Subsets (Whole Blood)[19b]			X	X	X	X	X					
Cryopreserved PBMC[19b]			X	X	X	X	X					

Table continued on next page

VISIT	Pre-Screening[1]	Screening[2]	Combination Treatment Period Pembrolizumab + Zolbetuximab cycle = 21 days				Zolbetuximab Study Drug Discontinuation Visit[29]	Pembrolizumab Study Drug Discontinuation Visit[29]	Zolbetuximab 30 Day Safety Follow-up Visit[30]	Pembrolizumab 30 Day Safety Follow-up Visit[31]	Zolbetuximab 90 Day Follow-up Visit[32]	Pembrolizumab 90 Day Follow-up Visit[33]
**Cycle[3]**			**1**	**2**	**3**	**≥ 4**						
**Day**			**1**	**22**	**43**	**64**						
Visit Window (business days)		-28 to -1	0	+ 2	+ 2	+ 2	+ 7	+ 7	± 7	± 7	+ 7	+ 7
Genetic Immune Polymorphisms (Whole blood)[19c]			X									
Whole Blood Sample for PGx (optional)[20]			X									
**Cardiac Safety**												
12-lead ECG [21]		X	If clinically indicated				X		X			
**Radiology**												
Image Assessment[22]		X	Every 6 (± 1) weeks from C1D1 for the first 24 weeks and then every 12 (± 2) weeks thereafter.									
**HRQoL**												
HRQoL[23]			X	X	X	X	X		X		X	
**Tissue Sample**												
Baseline Tumor Sample[24] (optional)		X										
On-Treatment Biopsy[25] (optional)					X							
Post-progression Tumor Sample (optional)[26]							X					
**Safety Assessment**												
Concomitant Medication[27]		X	X	X	X	X	X	X	X	X	X	X
AEs/SAEs[28]		X	X	X	X	X	X	X	X	X	X	X

Footnotes appear on next page

ADA: anti-drug antibody; AE: adverse event; βHCG: beta human chorionic gonadotropin; BSA: body surface area; C1D1: cycle 1 day 1; CLDN: claudin; CT: computerized tomography; eCRF: electronic case report form; ECG: electrocardiogram; ECOG: Eastern Cooperative Oncology Group; FFPE: formalin-fixed paraffin-embedded; GP: Global Pain; HRQoL: health-related quality of life; HRU: Health Resource Utilization; ICF: informed consent form; INR: international normalized ratio; IRR: infusion related reaction; IV: intravenous; MRI: magnetic resonance imaging ; ORR: objective response rate; PBMC: peripheral blood mononuclear cells; PET: positron emission tomography; PGx: pharmacogenomics; PFS: progression-free survival; PGx: pharmacogenomics; PT: prothrombin time; PTT: partial thromboplastin time; SAE: serious adverse event; T4: thyroxine; TSH: thyroid-stimulating hormone

1. Pre-Screening: FFPE tumor specimens will be collected for central pre-screening tests to determine CLDN18.2 and PD-L1 status (if required). There should be sufficient tumor tissue in the specimen used to send to the central laboratory for testing. Archival tumor tissue is preferred. A minimum of 1 FFPE tumor tissue block (preferred) OR a minimum of 9 FFPE unstained sections are required. If slides are submitted, the slides should be freshly cut from the FFPE block within the time frame described in the laboratory manual. If archival tumor tissue is insufficient or unavailable for pre-screening, the subject will enter the screening period and a biopsy will be performed to obtain the tumor sample for eligibility. If ≥ 9 slides cannot be provided, the sponsor should be contacted for further guidance.
2. Screening period is 28 days. Subjects may be rescreened once during the screening period. Any procedures that fall outside of the screening period must be repeated. If more than 1 assessment is taken during the screening period, the assessment closest to enrollment date should be used for eligibility.

   <u>Laboratory testing:</u>

   Eligibility can be determined based on central and/or local testing; however:

   - The most recent laboratory data must be used to confirm the subject's eligibility.
   - Central labs must be collected and submitted to the central laboratory during the Screening period.
   - If retesting of lab values is necessary to confirm eligibility, local labs can be used without requiring additional sample collection for central laboratory submission.
   - The screening labs used to determine eligibility should be collected within 14 days prior to enrollment.
3. Assessments should be collected on day 1 of each corresponding cycle.
4. If archival tumor tissue is insufficient or unavailable for pre-screening, the subject will enter the screening period and a biopsy will be performed to obtain the tumor sample for eligibility.
5. All laboratory results should be available and reviewed by the investigator before starting any study treatment (zolbetuximab/pembrolizumab).
6. Prophylactic antiemetics and, if needed, other premedications should be given according to institutional standards and the respective product package insert(s). Antiemetic premedication should be given at least 30 minutes prior to each dose of zolbetuximab. It is recommended that the prophylactic antiemetic regimen include the following agents: NK-1 receptor blockers and 5-HT3 receptor blockers. On days when subjects receive both zolbetuximab and pembrolizumab, antiemetic premedication will be given prior to zolbetuximab administration.
7. Zolbetuximab will be administered as a minimum 2-hour IV infusion. Zolbetuximab IV infusion may be interrupted or slowed down to manage toxicity.
8. Post-Infusion Observation Period: Following the subject's first dose of zolbetuximab on C1D1, the subject must remain at the site facility for 2 hours post infusion. If AEs are observed during this time, infusion time should be extended and subjects should continue to be observed for 2 hours post infusion.
9. Pembrolizumab will be administered every 3 weeks as an infusion of 200 mg over 30 minutes. Zolbetuximab should be administered and completed 1 hour prior to pembrolizumab administration.

Footnotes continued on next page

10. Physical examination and other evaluations include height (at screening only), weight and ECOG performance status. The physical exam only needs to be repeated on C1D1 if clinically significant changes from screening (in the opinion of the investigator) are observed. Targeted (symptom-driven) physical exams should be conducted every 3 weeks on zolbetuximab visit days.
11. Vital signs for **zolbetuximab infusion** (pulse, blood pressure, temperature) should be taken at the following time points:
    - Predose at every visit
    - C1D1: Every 30 (± 10) minutes during zolbetuximab infusion.
    - Subsequent zolbetuximab infusions: every 60 (± 10) minutes during zolbetuximab infusions if the subject did not develop any ≥ grade 2 AEs during the C1D1 zolbetuximab infusion or the Post-Infusion Observation Period.
    - Every 60 (±10) minutes post zolbetuximab infusion during the Post-Infusion Observation Period.
    - Unscheduled if clinically indicated

    Vital signs for **pembrolizumab infusion** (pulse, blood pressure, temperature) should be taken at the following time points:
    - Predose at every visit
    - Every 15 minutes (-5 to +10 minute window) after the start of infusion
    - At the end of pembrolizumab infusion (-5 to +10 minute window)
    - At 30 (± 10) minutes after completion of pembrolizumab infusion
12. See [Section 5.4.3] for list of laboratory assessments. Laboratory tests must be sent to the central laboratory for analysis unless otherwise approved by the sponsor. For screening/eligibility laboratory assessments, see footnote 2.
    - Laboratory test results (central or local) will be reviewed by the investigator prior to any study treatment. Clinical significance of out-of-range laboratory findings is to be determined and documented by the investigator/subinvestigator who is a qualified physician.
    - Local laboratory results may be used for treatment decisions; however, central laboratory samples must also be drawn per protocol and sent to the central laboratory unless otherwise approved by the sponsor.
    - Central and local labs may be collected up to 48 hours prior to study treatment.
    - Holidays and weekends should be taken into account when scheduling these blood draws.
    - Additional assessments may be done centrally or locally to monitor AEs or as clinically indicated.
13. Urinalysis: Urinalysis should be performed at screening and is to be repeated if clinically indicated after starting study treatment. Urinalysis should also be performed for the zolbetuximab and pembrolizumab study drug discontinuation visits and at the zolbetuximab 30-day follow-up visit. Urinalysis tests will be sent to a central laboratory for analysis.

Footnotes continued on next page

14. Cytokines/chemokines and/or tryptase need to be collected centrally.
15. Assessments must be completed at the following time points:
    - Screening
    - Baseline
    - Predose cycle 2
    - Predose cycle 4 and every other cycle thereafter.
    - Pembrolizumab study treatment discontinuation
    - Pembrolizumab 30 day follow-up visit
16. Ongoing evaluation should be continued for subjects who are receiving therapeutic anticoagulation according to local standard of care.
17. Serum Pregnancy Test: Will be collected for female subjects of childbearing potential only. Serum pregnancy tests are to be completed at screening. Subjects with elevated serum βHCG during screening and a demonstrated non-pregnant status through additional testing are eligible. Local or central laboratory results must be used to confirm eligibility.
18. Urine Pregnancy Test: For female subjects of childbearing potential only. Urine pregnancy tests are to be completed at every visit prior to zolbetuximab administration. Urine pregnancy can be confirmed at a local laboratory.
19. Biomarker (serum, plasma, whole blood and cryopreserved PBMCs) samples should be taken within 48 hours prior to dosing:
    a. Samples will be collected predose at every cycle through zolbetuximab study drug discontinuation.
    b. Samples will be collected predose at C1D1, C2D1, C3D1, C4D1, C6D1, C8D1 and zolbetuximab study drug discontinuation.
    c. Samples will be collected predose on C1D1.
20. For subjects who signed a separate ICF, an optional whole blood sample for PGx for exploratory biomarker analysis should be collected within 48 hours prior to dosing.
21. A single ECG will be performed at the following time points:
    - Screening
    - Zolbetuximab study drug discontinuation
    - Zolbetuximab 30-day follow-up visit
    - If clinically indicated

    †Local read only – do not transmit to central ECG laboratory.
22. Imaging assessments to be collected at screening and every 6 (± 1) weeks counting from C1D1 for the first 24 weeks, and then every 12 (± 2) weeks thereafter. Imaging assessments completed prior to consent, but within 28 days of first dose of study drug, may be used for screening. Imaging will include CT scans with contrast of the thorax, abdomen, and pelvis (if CT scan is medically not feasibly with contrast, MRI may be used for imaging). Bone scans (or focal X-ray) or brain imaging should be performed if metastatic disease is suspected. Disease must be evident by radiology; measurable lesions only. Same mode of imaging should be utilized throughout the study unless medical necessity requires change. CT scan performed with PET scan can be used if it is of quality, which allows accurate tumor measurement. Progression and tumor responses will be evaluated by investigator per RECIST 1.1. All imaging will be sent to a central independent radiographic review within 7 days.

Footnotes continued on next page

23. HRQoL questionnaires and HRU are to be administered on zolbetuximab visit days before any drug treatment (or up to 48 hours prior to treatment) or other scheduled assessments are conducted and before the disease status is discussed with the subject. HRQoL will be measured by Quality of Life Questionnaire – Core Questionnaire (QLQ-C30), Oesophago-Gastric Module (OG-25), Global Pain (GP) and the EuroQOL Five Dimensions (EQ-5D) questionnaires. The HRU questionnaire will not be administered at the screening visit. Questionnaires should only be administered on days when the subject receives zolbetuximab treatment. Questionnaire completion will not be required if the subject is illiterate or the questionnaire is not available in the local language.
24. Optional for Cohort 3A: Subjects may provide a tumor specimen collected within 3 months prior to the first dose of study treatment. If the specimen is insufficient or unavailable, a biopsy may be performed to obtain tumor tissue. Ensure that subject meets all other study eligibility criteria prior to performing the biopsy (as applicable). Tissue slides or blocks submitted as the baseline tumor sample must be a separate submission than the tumor sample for eligibility. A minimum of 1 FFPE tumor tissue block (preferred) OR a minimum of 15 FFPE unstained sections are required. This is optional for Cohort 3A. If ≥ 15 slides cannot be provided, the sponsor should be contacted for further guidance.
25. Optional for Cohort 3A: Subjects may provide an on-treatment tumor specimen collected ± 15 days of the C3D1 visit. A minimum of 1 FFPE tumor tissue block (preferred) OR a minimum of 15 FFPE unstained sections are required. This is optional for Cohort 3A. If ≥ 15 slides cannot be provided, the sponsor should be contacted for further guidance.
26. For subjects who signed a separate ICF, an optional post-progression tumor sample for exploratory biomarker analysis should be collected following confirmation of disease progression and prior to commencement of subsequent anti-cancer therapy. A minimum of 1 FFPE tumor tissue block (preferred) OR a minimum of 15 FFPE unstained sections are required.
27. Concomitant medications will be collected from the time of main study informed consent through 90 days following the last dose of study drug.
28. AEs and SAEs (regardless of causality) will be collected from the time of main informed consent through 90 days following the last dose of study drug.
29. Study Drug Discontinuation: Visit will occur within 7 days after the last dose or decision by the investigator to discontinue subject from treatment.
30. Zolbetuximab 30-day Safety Follow-up Visit should occur 30 days (± 7) after last dose of zolbetuximab.
31. Pembrolizumab 30-day Safety Follow-up phone call should occur 30 days (± 7) after last dose of pembrolizumab (all components).
32. Zolbetuximab 90-day Follow-up Visit should occur 90 days (+ 7) after last dose of zolbetuximab.
33. Pembrolizumab 90-day Safety Follow-up phone call should occur 90 days (+ 7) after the last dose of pembrolizumab (all components).

Table 8 Cohort 3A– Zolbetuximab and Pembrolizumab Pharmacokinetic/Immunogenicity Schedule

Study Day (1 cycle = 3 Weeks)		Time (Relative to Dosing in each cycle)	Zolbetuximab Pharmacokinetics [1,2]	Zolbetuximab Immunogenicity [3]	Pembrolizumab Pharmacokinetics [4]
Cycle 1	Day 1	Predose (0 hour)	X	X	X
		EOI	X		X
		0.5 hour after EOI	X		
		3 hours after EOI	X		
		6 hours after EOI	X		
	Day 2	24 hours after EOI	X		
	Day 4	72 hours after EOI	X		
	Day 8	168 hours after EOI	X		
	Day 15	336 hours after EOI	X		
Cycle 2	Day 1	Predose (504 hours after C1D1 EOI)	X	X	
Cycles 3, 5 & 9	Day 1	Predose (0 hour)	X	X	X
		EOI	X		X
Cycles 13 & 17	Day 1	Predose (0 hour)	X	X	X
30-day Safety Follow-up Visit			X	X	X
90-day Follow-up Visit			X	X	X

C1D1: cycle 1 day 1; EOI: end of infusion

1. Zolbetuximab pharmacokinetic sampling windows are as below. The date and time of each sample collection will be recorded to the nearest minute.
   - Predose: within 60 minutes prior to **zolbetuximab** dosing
   - End of infusion (EOI): within 10 minutes after the end of the **zolbetuximab** infusion
   - Samples at 0.5 to 6 hours after EOI: ± 10 minutes
2. Unscheduled pharmacokinetic blood samples may be taken at any time during the study to evaluate drug exposure following a safety event.
3. Zolbetuximab immunogenicity sampling window: Predose: within 60 minutes prior to **zolbetuximab** dosing.
4. Pembrolizumab pharmacokinetics sampling windows are as below. The date and time of each sample collection will be recorded to the nearest minute.
   - Predose: within 60 minutes prior to **zolbetuximab** dosing
   - End of infusion (EOI): within 10 minutes after the end of the **pembrolizumab** infusion

Table 9 Schedule of Assessments for Cohort 4

VISIT	Pre-Screening[1]	Screening[2]	Treatment Period (1 cycle = 42 days)						Zolbetuximab Study Drug Discontinuation Visit[28]	mFOLFOX6 and/or nivolumab Study Drug Discontinuation Visit[28]	Zolbetuximab 30-Day Safety Follow-up Visit[29]	mFOLFOX6 and/or nivolumab 30-Day Safety Follow-up Visit[30]	Zolbetuximab 90-Day Follow-up Visit[31]	mFOLFOX6 and/or nivolumab 90-Day Safety Follow-up Visit[32]	Post-treatment Follow-up[33]	Survival Follow-up (Cohort 4B only)[34]
			Cycles 1 to 4<br>zolbetuximab + mFOLFOX6 + nivolumab			Cycles ≥ 5<br>zolbetuximab + leucovorin or folinic acid + 5-FU + nivolumab										
**Day**			**1**	**15**	**29**	**1**	**15**	**29**								
Visit Window (business days)		-28 to -1	+ 2[3]	+ 2	+ 2	+ 2	+ 2	+ 2	+ 7	+ 7	± 7	±7	± 7	± 7	± 14	
Pre-Screening ICF	X															
Tumor Sample for Eligibility[1]	X															
Main Study ICF		X														
Tumor Biopsy[4]		X														
Medical and Disease History		X														
Confirmation of Eligibility	X[36]	X	X													
Cohort Assignment[5]			X													
**Study Drug Administration**																
Antiemetic Pretreatment[6]			X	X	X	X	X	X								
Zolbetuximab[7]			X	X	X	X	X	X								

Table continued on next page

VISIT	Pre-Screening[1]	Screening[2]	**Treatment Period (1 cycle = 42 days)** Cycles 1 to 4: zolbetuximab + mFOLFOX6 + nivolumab			Cycles ≥ 5: zolbetuximab + leucovorin or folinic acid + 5-FU + nivolumab			Zolbetuximab Study Drug Discontinuation Visit[28]	mFOLFOX6 and/or nivolumab Study Drug Discontinuation Visit[28]	Zolbetuximab 30-Day Safety Follow-up Visit[29]	mFOLFOX6 and/or nivolumab 30-Day Safety Follow-up Visit[30]	Zolbetuximab 90-Day Follow-up Visit[31]	mFOLFOX6 and/or nivolumab 90-Day Safety Follow-up Visit[32]	Post-treatment Follow-up[33]	Survival Follow-up (Cohort 4B only)[34]
**Day**			**1**	**15**	**29**	**1**	**15**	**29**								
Visit Window (business days)		-28 to -1	+ 2[3]	+ 2	+ 2	+ 2	+ 2	+ 2	+ 7	+ 7	± 7	±7	± 7	± 7	± 14	
Post-Infusion Observation Period[8]			X	X	X	X	X	X								
Nivolumab[9]			X	X	X	X	X	X								
mFOLFOX6[10]			X	X	X	X	X	X								
**Physical Examination/Assessments**																
Physical Examination[11]		X	X	X	X	X	X	X	X	X	X					
Weight[11]		X	X	X	X	X	X	X	X	X	X					
ECOG Performance Status[11]		X	X	X	X	X	X	X	X	X	X					
Vital Signs[12]		X	X	X	X	X	X	X	X	X	X					
**Laboratory Assessments**																
Biochemistry[13]		X	X	X	X	X	X	X	X	X	X					
Hematology[13]		X	X	X	X	X	X	X	X	X	X					
Urinalysis[14]		X	If clinically indicated						X	X	X					
DPD testing per local requirements		X														

Table continued on next page

VISIT	Pre-Screening[1]	Screening[2]	Treatment Period (1 cycle = 42 days)						Zolbetuximab Study Drug Discontinuation Visit[28]	mFOLFOX6 and/or nivolumab Study Drug Discontinuation Visit[28]	Zolbetuximab 30-Day Safety Follow-up Visit[29]	mFOLFOX6 and/or nivolumab 30-Day Safety Follow-up Visit[30]	Zolbetuximab 90-Day Follow-up Visit[31]	mFOLFOX6 and/or nivolumab 90-Day Safety Follow-up Visit[32]	Post-treatment Follow-up[33]	Survival Follow-up (Cohort 4B only)[34]
			**Cycles 1 to 4**<br><br>**zolbetuximab + mFOLFOX6 + nivolumab**			**Cycles ≥ 5**<br><br>**zolbetuximab + leucovorin or folinic acid + 5-FU + nivolumab**										
**Day**			**1**	**15**	**29**	**1**	**15**	**29**								
Visit Window (business days)		-28 to -1	+ 2[3]	+ 2	+ 2	+ 2	+ 2	+ 2	+ 7	+ 7	± 7	±7	± 7	± 7	± 14	
Cytokine/Chemokine and/or Tryptase			If clinically indicated													
TSH and T4		X	X[35]			X[35]										
PT, PTT and INR[15]		X	If clinically indicated													
Serum Pregnancy Test[16]		X														
Urine Pregnancy Test[17]			X	X	X	X	X	X	X	X	X					
Pharmacokinetics of Zolbetuximab			See Table 10 below for pharmacokinetic collection schedule								X		X			
Pharmacokinetics of Nivolumab			See Table 10 below for pharmacokinetic collection schedule									X		X		
Anti-Drug Antibodies (immunogenicity)			See Table 10 below for sample collection schedule								X		X			
Tumor Markers[18a]			X	X	X	X			X							

Table continued on next page

VISIT	Pre-Screening[1]	Screening[2]	Treatment Period (1 cycle = 42 days)						Zolbetuximab Study Drug Discontinuation Visit[28]	mFOLFOX6 and/or nivolumab Study Drug Discontinuation Visit[28]	Zolbetuximab 30-Day Safety Follow-up Visit[29]	mFOLFOX6 and/or nivolumab 30-Day Safety Follow-up Visit[30]	Zolbetuximab 90-Day Follow-up Visit[31]	mFOLFOX6 and/or nivolumab 90-Day Safety Follow-up Visit[32]	Post-treatment Follow-up[33]	Survival Follow-up (Cohort 4B only)[34]
			**Cycles 1 to 4**<br>**zolbetuximab + mFOLFOX6 + nivolumab**			**Cycles ≥ 5**<br>**zolbetuximab + leucovorin or folinic acid + 5-FU + nivolumab**										
**Day**			**1**	**15**	**29**	**1**	**15**	**29**								
Visit Window (business days)		-28 to -1	+ 2[3]	+ 2	+ 2	+ 2	+ 2	+ 2	+ 7	+ 7	± 7	±7	± 7	± 7	± 14	
Exploratory Biomarkers (Serum)[18b]			X	X	X				X							
Exploratory Biomarkers (Plasma)[18b]			X	X	X				X							
Immune Cell Subsets (Whole Blood)[18b]			X	X	X				X							
Cryopreserved PBMC[18b]			X	X	X				X							
Genetic Immune Polymorphisms – (whole blood)[18c]			X													
Whole Blood Sample for PGx (optional)[19]			X													
**Cardiac Safety**																
12-lead ECG[20]		X	X	X	X	If clinically indicated			X		X					
Table continued on next page																

VISIT	Pre-Screening[1]	Screening[2]	Treatment Period (1 cycle = 42 days)						Zolbetuximab Study Drug Discontinuation Visit[28]	mFOLFOX6 and/or nivolumab Study Drug Discontinuation Visit[28]	Zolbetuximab 30-Day Safety Follow-up Visit[29]	mFOLFOX6 and/or nivolumab 30-Day Safety Follow-up Visit[30]	Zolbetuximab 90-Day Follow-up Visit[31]	mFOLFOX6 and/or nivolumab 90-Day Safety Follow-up Visit[32]	Post-treatment Follow-up[33]	Survival Follow-up (Cohort 4B only)[34]
			**Cycles 1 to 4**<br>**zolbetuximab + mFOLFOX6 + nivolumab**			**Cycles ≥ 5**<br>**zolbetuximab + leucovorin or folinic acid + 5-FU + nivolumab**										
**Day**			**1**	**15**	**29**	**1**	**15**	**29**								
Visit Window (business days)		-28 to -1	+ 2[3]	+ 2	+ 2	+ 2	+ 2	+ 2	+ 7	+ 7	± 7	±7	± 7	± 7	± 14	
**Radiology**																
Image Assessment[21]		X	Every 8 (± 1) weeks from C1D1 for the first 56 weeks and then every 12 (± 2) weeks thereafter.													
**HRQoL**																
HRQoL[22]		X	X	X	X	X	X	X	X		X		X			
**Tissue Samples**																
Baseline Tumor Sample[23] (Required for Cohort 4B, optional for Cohort 4A)		X														
On-Treatment Biopsy[24] (Required for Cohort 4B, optional for Cohort 4A)			X													
Post-progression Tumor Sample (optional) [25]									X							

Table continued on next page

VISIT	Pre-Screening[1]	Screening[2]	Treatment Period (1 cycle = 42 days): Cycles 1 to 4 zolbetuximab + mFOLFOX6 + nivolumab			Treatment Period (1 cycle = 42 days): Cycles ≥ 5 zolbetuximab + leucovorin or folinic acid + 5-FU + nivolumab			Zolbetuximab Study Drug Discontinuation Visit[28]	mFOLFOX6 and/or nivolumab Study Drug Discontinuation Visit[28]	Zolbetuximab 30-Day Safety Follow-up Visit[29]	mFOLFOX6 and/or nivolumab 30-Day Safety Follow-up Visit[30]	Zolbetuximab 90-Day Follow-up Visit[31]	mFOLFOX6 and/or nivolumab 90-Day Safety Follow-up Visit[32]	Post-treatment Follow-up[33]	Survival Follow-up (Cohort 4B only)[34]
**Day**			**1**	**15**	**29**	**1**	**15**	**29**								
Visit Window (business days)		-28 to -1	+ 2[3]	+ 2	+ 2	+ 2	+ 2	+ 2	+ 7	+ 7	± 7	±7	± 7	± 7	± 14	
**Safety Assessment**																
Concomitant Medication[26]			X	X	X	X	X	X	X	X	X	X	X	X		
AEs/SAEs[27]		X	X	X	X	X	X	X	X	X	X	X	X	X		
Survival Follow-up[33]																X

5-FU: fluorouracil; ADA: anti-drug antibody; AE: adverse event; BSA: body surface area; C: cycle; CLDN: claudin; CT: computerized tomography; D: day; eCRF: electronic case report form; ECG: electrocardiogram; ECOG: Eastern Cooperative Oncology Group; FFPE: formalin-fixed paraffin-embedded; GP: Global Pain; HER2: human epidermal growth factor receptor 2; HRQoL: health-related quality of life; HRU: Health Resource Utilization; ICF: informed consent form; INR: international normalized ratio; IRR: infusion related reaction; IV: intravenous; MRI: magnetic resonance imaging; ORR: objective response rate; PBMC: peripheral blood mononuclear cells; PET: positron emission tomography; PFS: progression-free survival; PGx: pharmacogenomics; PT: prothrombin time; PTT: partial thromboplastin time; SAE: serious adverse event; T4: thyroxine; TSH: thyroid-stimulating hormone.

Laboratory tests will be performed predose according to the Schedule of Assessments and sent to a central laboratory for analysis. In case of multiple laboratory data within this period, the most recent data should be used.

Footnotes continued on next page

1. Pre-Screening: FFPE tumor tissue will be collected for central testing to determine CLDN18.2 and HER2 status (if required). Archival tumor tissue from the primary tumor (gastric or GEJ) is preferred. If primary tumor tissue is not available, tumor tissue from a metastatic site (excluding bone metastasis) may be used. A minimum of 1 FFPE tumor tissue block (preferred) OR a minimum of 15 FFPE unstained slides are required, as allowed per local policy. If slides are submitted, the slides should be freshly cut from the FFPE block within the time frame described in the laboratory manual. If HER2 results are already available from local testing, a minimum of 12 FFPE unstained slides are required to be submitted to the central lab. If the specimen is insufficient or unavailable, the subject will enter the screening period and a biopsy will be performed to obtain primary tumor tissue (preferred) or tumor tissue from metastatic site (excluding bone metastasis). Sponsor pre-approval is required when the sole purpose of the biopsy procedure is to assess eligibility for this study.
2. Screening period is 28 days. Subjects may be rescreened once during the screening period. Any procedures that fall outside of the screening period must be repeated. If more than 1 assessment is taken during the screening period, the assessment closest to enrollment date should be used for eligibility.

   Laboratory testing:

   Eligibility can be determined based on central and/or local testing; however:

   - The most recent laboratory data must be used to confirm the subject's eligibility.
   - Central labs must be collected and submitted to the central laboratory during the Screening period.
   - If retesting of lab values is necessary to confirm eligibility, local labs can be used without requiring additional sample collection for central laboratory submission.
   - The screening labs used to determine eligibility should be collected within 14 days prior to enrollment.
3. For C1D1, there is a 0-business day visit window.
4. If archival tumor tissue is insufficient or unavailable for pre-screening, the subject will enter the screening period and a biopsy will be performed to obtain the tumor sample for eligibility.
5. All laboratory results should be available and reviewed by the investigator before starting any study treatment (zolbetuximab, mFOLFOX6, nivolumab).
6. Prophylactic antiemetics and, if needed, other premedications should be given according to institutional standards and the respective product package insert(s). Antiemetic premedication should be given at least 30 minutes prior to zolbetuximab administration. If one study drug is discontinued, antiemetic premedication should be given at least 30 minutes prior to each dose of zolbetuximab, mFOLFOX6 and nivolumab. It is recommended that the prophylactic antiemetic regimen include the following agents: NK-1 receptor blockers and 5-HT3 receptor blockers.
7. Zolbetuximab will be administered as a minimum 2-hour IV infusion. Zolbetuximab IV infusion may be interrupted or slowed down to manage toxicity.
8. Post-Infusion Observation Period: Following the subject's first dose of zolbetuximab, the subject must remain at the site facility for 2 hours post infusion. If AEs are observed during this time, infusion time should be extended and subjects should continue to be observed for 2 hours post infusion.
9. Nivolumab will be administered every 2 weeks at a dose of 240 mg. Zolbetuximab will be administered first, followed by nivolumab and then mFOLFOX6.
10. mFOLFOX6 will be administered every 2 weeks. mFOLFOX6 will be administered after zolbetuximab and nivolumab. Beginning at cycle 5, subjects may continue on 5-FU and leucovorin or folinic acid along with zolbetuximab and nivolumab for the remainder of the study per investigator's discretion.
11. Physical examination and other evaluations include height (at screening only), weight and ECOG performance status. The physical exam only needs to be repeated on C1D1 if clinically significant changes from screening (in the opinion of the investigator) are observed. Targeted (symptom-driven) physical exams should be conducted every 2 weeks on zolbetuximab visit days.
12. Vital signs (pulse, blood pressure, temperature) should be taken at the following time points:

Footnotes continued on next page

- Predose at every visit.
- C1D1: Every 30 (± 10) minutes during zolbetuximab infusion.
- Subsequent zolbetuximab infusions: every 60 (± 10) minutes during zolbetuximab infusions if the subject did not develop any ≥ grade 2 AEs during the C1D1 zolbetuximab infusion or the Post-Infusion Observation Period. If the subject has an ≥ grade 2 AE, then vital signs are to be taken be every 30 minutes, subsequently. If the next cycle is ≤ grade 1, then vital signs can be assessed every 60 minutes.
- Every 60 (±10) minutes post zolbetuximab infusion during the Post-Infusion Observation Period.
- Unscheduled if clinically indicated

13. See [Section 5.4.3] for list of laboratory assessments. Laboratory tests must be sent to the central laboratory for analysis unless otherwise approved by the sponsor. For screening/eligibility laboratory assessments, see footnote 2.
    - Laboratory test results (central or local) will be reviewed by the investigator prior to any study treatment. Clinical significance of out-of-range laboratory findings is to be determined and documented by the investigator/subinvestigator who is a qualified physician.
    - Local laboratory results may be used for treatment decisions; however, central laboratory samples must also be drawn per protocol and sent to the central laboratory unless otherwise approved by the sponsor.
    - Central and local labs may be collected up to 48 hours prior to study treatment.
    - Holidays and weekends should be taken into account when scheduling these blood draws.
    - Additional assessments may be done centrally or locally to monitor AEs or as clinically indicated.
14. Urinalysis: Urinalysis should be performed at screening and is to be repeated if clinically indicated after starting study treatment. Urinalysis should also be performed for the zolbetuximab, mFOLFOX6 and nivolumab study drug discontinuation visits and at the zolbetuximab 30-day follow-up visit. Urinalysis tests will be sent to a central laboratory for analysis.
15. Ongoing evaluation should be continued for subjects who are receiving therapeutic anticoagulation according to local standard of care.
16. Serum Pregnancy Test: Will be collected for female subjects of childbearing potential only. Serum pregnancy tests are to be completed at screening. Subjects with elevated serum βHCG during screening and a demonstrated non-pregnant status through additional testing are eligible. Central laboratory must be used to confirm eligibility.
17. Urine Pregnancy Test: For female subjects of childbearing potential only. Urine pregnancy tests are to be completed at every visit prior to zolbetuximab administration. Urine pregnancy can be confirmed at a local laboratory.
18. Biomarker (serum, plasma, whole blood and cryopreserved PBMCs) samples should be taken within 48 hours prior to dosing:
    a. Samples will be collected predose at C1D1, C1D15, C1D29, C2D1, C2D29, C3D15, C4D1, C4D29 and then predose on D1 of every additional cycle through zolbetuximab study drug discontinuation.
    b. Samples will be collected predose at C1D1, C1D15, C1D29, C2D1, C2D29, C3D15, C4D1, C4D29 and zolbetuximab study drug discontinuation.
    c. Samples will be collected predose on C1D1.
19. For subjects who signed a separate ICF, an optional whole blood sample for PGx for exploratory biomarker analysis should be collected within 48 hours prior to dosing.

Footnotes continued on next page

20. A single ECG will be performed at the following time points:
    - Screening
    - Up to 48 hours prior to every oxaliplatin infusion (before any antiemetic treatment)
    - Up to 6 hours following completion of every oxaliplatin infusion
    - Zolbetuximab study drug discontinuation
    - Zolbetuximab 30-day follow-up visit
    - If clinically indicated
21. Imaging assessments to be collected at screening and every 8 (± 1) weeks counting from C1D1 for the first 56 weeks, and then every 12 (± 2) weeks thereafter. Imaging assessments completed prior to consent, but within 28 days of first dose of study drug, may be used for screening. Imaging will include CT scans with contrast of the thorax, abdomen, and pelvis (if CT scan is medically not feasibly with contrast, MRI may be used for imaging). Bone scans (or focal X-ray) or brain imaging should be performed if metastatic disease is suspected. Disease must be evident by radiology; measurable lesions only. Same mode of imaging should be utilized throughout the study unless medical necessity requires change. CT scan performed with PET scan can be used if it is of quality, which allows accurate tumor measurement. Progression and tumor responses will be evaluated by investigator per RECIST 1.1. All imaging will be sent to a central independent radiographic review within 7 days.
22. HRQoL questionnaires and HRU are to be administered on zolbetuximab visit days before any drug treatment (or up to 48 hours prior to treatment) or other scheduled assessments are conducted and before the disease status is discussed with the subject. HRQoL will be measured by QLQ-C30, OG-25, GP and the EQ-5D questionnaires. The HRU questionnaire will not be administered at the screening visit. Questionnaires should only be administered on days when the subject receives zolbetuximab treatment. A combined visit can be completed if zolbetuximab is discontinued on the same day. Questionnaire completion will not be required if the subject is illiterate or the questionnaire is not available in the local language.
23. Cohort 4B Only: Subjects are required to provide a tumor specimen collected within 3 months prior to the first dose of study treatment. If the specimen is insufficient or unavailable, a biopsy may be performed to obtain tumor tissue. Ensure that subject meets all other study eligibility criteria prior to performing the biopsy (as applicable). Tissue slides or blocks submitted as the baseline tumor sample must be a separate submission than the tumor sample for eligibility. A minimum of 1 FFPE tumor tissue block (preferred) OR a minimum of 15 FFPE unstained sections are required. This is optional for Cohort 4A. If ≥ 15 slides cannot be provided, the sponsor should be contacted for further guidance.
24. Cohort 4B Only: Subjects are required to provide an on-treatment tumor specimen collected ± 15 days of the C2D1 visit. A minimum of 1 FFPE tumor tissue block (preferred) OR a minimum of 15 FFPE unstained sections are required. This is optional for Cohort 4A. If ≥ 15 slides cannot be provided, the sponsor should be contacted for further guidance.
25. For subjects who signed a separate ICF, an optional post-progression tumor sample for exploratory biomarker analysis should be collected following confirmation of radiographic disease progression and prior to commencement of subsequent anti-cancer therapy.
26. Concomitant medications will be collected from the time of main study informed consent through 90 days following the last dose of study drug.
27. AEs and SAEs (regardless of causality) will be collected from the time of main informed consent through 90 days following the last dose of study drug.
28. Study Drug Discontinuation: Visit will occur within 7 days after the last dose or decision by the investigator to discontinue subject from treatment.
29. Zolbetuximab 30-day Safety Follow-up Visit should occur 30 days (± 7) after last dose of zolbetuximab.

Footnotes continued on next page

30. mFOLFOX6/nivolumab 30-day Safety Follow-up Visit should occur 30 days (± 7) after last dose of mFOLFOX6 (all components) and/or nivolumab.
    - For subjects that have discontinued mFOLFOX6 and nivolumab on the same day, the safety follow-up visit must be conducted in-person.
    - For subjects that have discontinued on mFOLFOX6 and nivolumab on different days, the safety follow-up visit for nivolumab must be conducted in-person, while the safety follow-up visit for mFOLFOX6 can be conducted via phone call.
31. Zolbetuximab 90-day Follow-up Visit should occur 90 days (± 7) after last dose of zolbetuximab.
32. mFOLFOX6/nivolumab 90-day Safety Follow-up Visit should occur 90 days (± 7) after the last dose of mFOLFOX6 (all components) and/or nivolumab.
    - For subjects that have discontinued mFOLFOX6 and nivolumab on the same day, the safety follow-up visit must be conducted in-person.
    - For subjects that have discontinued on mFOLFOX6 and nivolumab on different days, the safety follow-up visit for nivolumab must be conducted in-person, while the safety follow-up visit for mFOLFOX6 can be conducted via phone call.
33. Post-treatment Follow-up: if a subject discontinues study drug prior to radiographic disease progression as confirmed by the independent central reader, the subject should enter the post-treatment follow-up period and continue to undergo imaging assessments until radiographic progression is documented per the investigator, or the subject starts another cancer treatment, whichever occurs earlier.
34. Cohort 4B only: Survival follow-up by telephone contact is to be performed every 12 weeks ± 2 weeks for all subjects following radiographic disease progression or the start of another anti-cancer therapy (whichever occurs first) or who left the study due to any other reason than death or withdrawal of consent. Subjects will be followed to collect survival status until subject death, withdrawal of consent, or study closure. Additional follow-up contacts may be required per sponsor request for analysis purposes.
35. Thyroid function to be assessed at day 1 of cycles 3, 5 and all odd cycles >5.
36. At pre-screening, data will be collected in the eCRF including date of the pre-screening ICF, tumor sample collection status, demographics, primary diagnosis, substance use-tobacco history and protocol version.

Table 10 Cohorts 4A and 4B – Zolbetuximab and Nivolumab Pharmacokinetic/Immunogenicity Schedule

Study Day (1 cycle = 6 Weeks)		Time (Relative to Dosing in each cycle)	Zolbetuximab Pharmacokinetics [1,2]		Zolbetuximab Immunogenicity [3]	Nivolumab Pharmacokinetics [4]
			Cohort 4A	Cohort 4B	Cohort 4 (A and B)	Cohort 4 (A and B)
Cycle 1	Day 1	Predose (0 hour)	X		X	X
		EOI	X	X		X
		0.5 hour after EOI	X			
		3 hours after EOI	X			
		6 hours after EOI	X			
	Day 2	24 hours after EOI	X			
	Day 4	72 hours after EOI	X			
	Day 8	168 hours after EOI	X			
	Day 15	Predose (336 hours after C1D1 EOI)	X	X	X	X
Cycles 2, 3 & 5	Day 1	Predose (0 hour)	X	X	X	X
		EOI	X	X		X
Cycles 7 & 9	Day 1	Predose (0 hour)	X	X	X	X
30-day Safety Follow-up Visit[5]			X	X	X	X
90-day Follow-up Visit[5]			X	X	X	X

C1D1: cycle 1 day 1; EOI: end of infusion

1. Zolbetuximab pharmacokinetic sampling windows are as below. The date and time of each sample collection will be recorded to the nearest minute.
   - Predose: within 60 minutes prior to **zolbetuximab** dosing
   - End of infusion (EOI): within 10 minutes after the end of the **zolbetuximab** infusion
   - Samples at 0.5 to 6 hours after EOI: ± 10 minutes
   - Samples at 24 to 168 hours after EOI: ± 120 minutes
2. Unscheduled pharmacokinetic blood samples may be taken at any time during the study to evaluate drug exposure following a safety event.
3. Zolbetuximab immunogenicity sampling window: Predose: within 60 minutes prior to **zolbetuximab** dosing.
4. Nivolumab pharmacokinetics sampling windows are as below. The date and time of each sample collection will be recorded to the nearest minute.
   - Predose: within 60 minutes prior to **zolbetuximab** dosing
   - End of infusion (EOI): within 10 minutes after the end of the **nivolumab** infusion
5. Zolbetuximab follow-up pharmacokinetics will be sampled at zolbetuximab follow-up visits, and nivolumab follow-up pharmacokinetics will be sampled at nivolumab follow-up visits.

Table 11 Schedule of Assessments for Cohort 5

VISIT	Pre-Screening[1]	Screening[2]	Preoperative cycles 1 to 4 zolbetuximab + FLOT		Preoperative Follow-up		Surgery[3]	Postoperative cycles 5 to 8 zolbetuximab + FLOT[23]		Postoperative Follow-up or Early Discontinuation			Post-Treatment Follow-up[25]	Survival Follow-up[26]
Day			1	2[4]	7-14 days after last preop dose	30 days after last preop dose	0	1	2[4]	EOT[24]	EOT +30	EOT +90		
Visit Window (business days)		-28 to -1	C1: 0<br>C2-4: +2	+ 2		± 7		+ 2	+ 2	+ 7	± 7	± 7		
Pre-Screening ICF	X													
Tumor Sample for Eligibility[1]	X	X												
Main Study ICF		X												
Medical and Disease History		X												
Confirmation of Eligibility	X	X												
Cohort Assignment[5]			X											
**Study Drug Administration**														
Antiemetic Pretreatment[6]			X	X[4]				X	X[4]					
Zolbetuximab[7]			X					X						
FLOT			X[4]	X[4]				X[4]	X[4]					
**Physical Examination/Assessments**														
Physical Examination[8]		X	X		X	X		X		X	X			
Weight[8]		X	X		X	X		X		X	X			
ECOG Performance Status[8]		X	X		X	X		X		X	X			
Vital Signs[9]		X	X		X	X		X		X	X			
HRQoL[10]		X	X		X	X		X		X	X	X	X	

Table continued on next page

VISIT	Pre-Screening[1]	Screening[2]	Preoperative cycles 1 to 4 zolbetuximab + FLOT		Preoperative Follow-up		Surgery[3]	Postoperative cycles 5 to 8 zolbetuximab + FLOT[23]		Postoperative Follow-up or Early Discontinuation			Post-Treatment Follow-up[25]	Survival Follow-up[26]
**Day**			**1**	**2[4]**	**7-14 days after last preop dose**	**30 days after last preop dose**	**0**	**1**	**2[4]**	**EOT[24]**	**EOT +30**	**EOT +90**		
Visit Window (business days)		-28 to -1	C1: 0<br>C2-4: +2	+ 2		± 7		+ 2	+ 2	+ 7	± 7	± 7		
**Laboratory Assessments**														
Biochemistry[11]		X	X		X	X		X		X	X			
Hematology[11]		X	X		X	X		X		X	X			
Urinalysis[12]		X	If Clinically Indicated							X	X			
DPD testing per local requirements		X												
Cytokine/Chemokine and/or Tryptase	If Clinically Indicated													
TSH and T4		X	X	If Clinically Indicated										
PT, PTT and INR[13]		X	If Clinically Indicated											
Serum Pregnancy Test[14]		X												
Urine Pregnancy Test[15]			X		X	X		X		X	X			
Pharmacokinetics of Zolbetuximab	See Table 12													
Anti-Drug Antibodies (immunogenicity)	See Table 12													
Exploratory Biomarkers (blood/plasma)[16]	See Table 13													
Table continued on next page														

VISIT	Pre-Screening[1]	Screening[2]	Preoperative cycles 1 to 4 zolbetuximab + FLOT		Preoperative Follow-up		Surgery[3]	Postoperative cycles 5 to 8 zolbetuximab + FLOT[23]		Postoperative Follow-up or Early Discontinuation			Post-Treatment Follow-up[25]	Survival Follow-up[26]
**Day**			**1**	**2[4]**	**7-14 days after last preop dose**	**30 days after last preop dose**	**0**	**1**	**2[4]**	**EOT[24]**	**EOT +30**	**EOT +90**		
Visit Window (business days)		-28 to -1	C1: 0<br>C2-4: +2	+ 2		± 7		+ 2	+ 2	+ 7	± 7	± 7		
Whole Blood Sample for PGx (optional)[17]			X											
**Tissue Samples**														
Postoperative (Surgical) Sample[18]							X							
Post-progression Tumor Sample (optional)[19]										X				
Diagnostic Laparoscopy (optional depending on institutional guidelines/standard-of-care)		X				X								
**Radiology**														
Image Assessment		X	Imaging at restaging (after preoperative chemotherapy prior to surgery) and after a subject discontinues or completes all study treatment (every 3 [± 1] months during the first year, then every 4 [± 2] months during the second year in post-treatment follow-up period.											
**Safety Assessments**														
12-lead ECG[20]		X	X	X	X	X		X	X	X	X			
Concomitant Medication[21]			X	X	X	X	X	X	X	X	X	X		
AEs/SAEs[22]		X	X	X	X	X	X	X	X	X	X	X		
Survival Assessment														X

AE: adverse event; βHCG: beta human chorionic gonadotropin; C: cycle; CLDN: claudin; D: day; DPD: dihydropyrimidine dehydrogenase; ECG: electrocardiogram; ECOG: Eastern Cooperative Oncology Group; EOT: end of treatment; EQ-5D: EuroQOL five dimensions questionnaire; FFPE: formalin-fixed paraffin-embedded; FLOT: fluorouracil, leucovorin or folinic acid, oxaliplatin and docetaxel; GEJ: gastroesophageal junction; GP: Global Pain; HRQoL: health-related quality of life; HRU: Health Resource Utilization; ICF: informed consent form; IV: intravenous; OG-25: Oesophago-Gastric Module (EORTC QLQ-OG-25); PGx: pharmacogenomics; preop: preoperative; PT: prothrombin time; PTT: partial thromboplastin time; QLQ-C30: Quality of Life Questionnaire - Core Questionnaire; SAE: serious adverse event; TSH: thyroid-stimulating hormone

Laboratory tests will be performed predose according to the Schedule of Assessments and sent to a central laboratory for analysis. In case of multiple laboratory data within this period, the most recent data should be used.

1. Pre-Screening: FFPE tumor tissue will be collected for central testing to determine CLDN18.2 status. Archival tumor tissue from the primary tumor (gastric or GEJ) is preferred. If primary tumor tissue is not available, tumor tissue from a metastatic site (excluding bone metastasis) may be used. A minimum of 1 FFPE tumor tissue block (preferred) OR a minimum of 10 FFPE unstained slides are required, as allowed per local policy. If slides are submitted, the slides should be freshly cut from the FFPE block within the time frame described in the laboratory manual. If archival tumor tissue is insufficient or unavailable for pre-screening, the subject will enter the screening period and a biopsy will be performed to obtain the tumor sample for eligibility.
2. Screening period is 28 days. Subjects may be rescreened once during the screening period. Any procedures that fall outside of the screening period must be repeated. If more than 1 assessment is taken during the screening period, the assessment closest to enrollment date should be used for eligibility.

   Laboratory screening testing:

   Eligibility can be determined based on central and/or local testing; however:

   - The most recent laboratory data must be used to confirm the subject’s eligibility
   - Central labs must be collected and submitted to the central laboratory during the screening period.
   - If retesting of lab values is necessary to confirm eligibility, local labs can be used without requiring additional sample collection for central laboratory submission.
   - The screening labs used to determine eligibility should be collected within 14 days prior to enrollment.
3. Surgery to be performed 4-8 weeks after completion of preoperative chemotherapy.
4. For cycles 1 and 5, zolbetuximab will be administered on day 1 and FLOT will be administered on day 2. For cycles 2-4 and 6-8, FLOT may be administered following zolbetuximab on day 1 or can be administered on day 2 as per investigator’s clinical judgment.
5. All laboratory results should be available and reviewed by the investigator before starting any study treatment.
6. Prophylactic antiemetics and, if needed, other premedications should be given according to institutional standards and the respective product package insert(s) (FLOT). Antiemetic premedication should be given at least 30 minutes prior to zolbetuximab administration. If one study drug is discontinued or administered on different days, antiemetic premedication should be given at least 30 minutes prior to each dose of zolbetuximab and FLOT. It is recommended that the prophylactic antiemetic regimen include the following agents: NK-1 receptor blockers and 5-HT3 receptor blockers.
7. Zolbetuximab will be administered with a preoperative loading dose at cycle 1 and a postoperative loading dose at cycle 5. Zolbetuximab IV infusion may be interrupted or slowed down to manage toxicity. Refer to [Section 5.1.1.1] for zolbetuximab dose regimen and infusion rate details.
8. Physical examination and other evaluations include height (at screening only), weight and ECOG performance status. The physical exam only needs to be repeated on C1D1 if clinically significant changes from screening (in the opinion of the investigator) are observed. Targeted (symptom-driven) physical exams should be conducted at every 2-week visit.

Footnotes continued on next page

9. Vital signs (pulse, blood pressure, temperature) should be taken at the following time points:
   - Predose at every visit.
   - C1D1: Every 30 (± 10) minutes during zolbetuximab infusion.
   - Subsequent zolbetuximab infusions: every 60 (± 10) minutes during zolbetuximab infusions if the subject did not develop any ≥ grade 2 AEs during the C1D1 zolbetuximab infusion or the Post-Infusion Observation Period. If the subject has a ≥ grade 2 AE, then vital signs are to be taken be every 30 minutes, subsequently. If the next cycle is ≤ grade 1, then vital signs can be assessed every 60 minutes.
   - Every 60 (±10) minutes post zolbetuximab infusion during the Post-Infusion Observation Period.
   - Unscheduled if clinically indicated.
10. HRQoL questionnaires and HRU are to be administered on zolbetuximab visit days before any drug treatment (or up to 48 hours prior to treatment) or other scheduled assessments are conducted and before the disease status is discussed with the subject. HRQoL will be measured by QLQ-C30, OG-25, GP and the EQ-5D questionnaires. The HRU questionnaire will not be administered at the screening visit. Questionnaires should only be administered on days when the subject receives zolbetuximab treatment. A combined visit can be completed if zolbetuximab is discontinued on the same day. Questionnaire completion will not be required if the subject is illiterate or the questionnaire is not available in the local language.
11. See [Section 5.4.3] for list of laboratory assessments. Laboratory tests must be sent to the central laboratory for analysis. For screening/eligibility laboratory assessments, see footnote 2.
   - Laboratory test results (central or local) will be reviewed by the investigator prior to any study treatment. Clinical significance of out-of-range laboratory findings is to be determined and documented by the investigator/subinvestigator who is a qualified physician.
   - Local laboratory results may be used for treatment decisions; however, central laboratory samples must also be drawn per protocol and sent to the central laboratory.
   - Central and local labs may be collected up to 48 hours prior to study treatment.
   - Holidays and weekends should be taken into account when scheduling these blood draws.
   - Additional assessments may be done centrally or locally to monitor AEs or as clinically indicated.
12. Urinalysis should be performed at screening and is to be repeated if clinically indicated after starting study treatment. Urinalysis should also be performed at the postoperative follow-up or early discontinuation visits (EOT and EOT +30). Urinalysis tests will be sent to a central laboratory for analysis.
13. Ongoing evaluation should be continued for subjects who are receiving therapeutic anticoagulation according to local standard of care.
14. Serum Pregnancy Test: Will be collected for female subjects of childbearing potential only. Serum pregnancy tests are to be completed at screening. Subjects with elevated serum βHCG during screening and a demonstrated non-pregnant status through additional testing are eligible. Central laboratory must be used to confirm eligibility.
15. Urine Pregnancy Test: For female subjects of childbearing potential only. Urine pregnancy tests are to be completed at every visit prior to zolbetuximab administration. Urine pregnancy can be confirmed at a local laboratory.
16. Biomarkers (blood, plasma) will be collected through the post-treatment follow-up period as indicated in [Table 13].
17. For subjects who signed a separate ICF, an optional whole blood sample for PGx for exploratory biomarker analysis should be collected within 48 hours prior to dosing.

Footnotes continued on next page

18. Subjects are required to provide the postoperative (surgical) sample. A minimum of 1 FFPE tumor tissue block (preferred) OR a minimum of 15 FFPE unstained sections are required.
19. For subjects who signed a separate ICF, an optional post-progression tumor sample for exploratory biomarker analysis should be collected following confirmation of radiographic disease progression and prior to commencement of subsequent anti-cancer therapy. A minimum 1 FFPE tumor tissue block (preferred) OR a minimum of 15 FFPE unstained sections are required.
20. A single ECG will be performed at the time points shown below.
    - Screening
    - Up to 48 hours prior to every oxaliplatin infusion (before any antiemetic treatment)
    - Up to 6 hours following completion of every oxaliplatin infusion
    - Preoperative Follow-up visits (7-14 days and 30 days after last preoperative dose)
    - Postoperative Follow-up or Early Discontinuation visits (EOT and 30-day follow-up)
    - If clinically indicated

ECG will be done on day 2 for cycles 1 and 5. Cycles 2-4 and 6-8 ECG to be performed on FLOT administration days.

21. Concomitant medications will be collected from the time of main study informed consent through 90 days following the last dose of study drug.
22. AEs and SAEs (regardless of causality) will be collected from the time of main informed consent through 90 days following the last dose of study drug, except for inpatient hospitalization for planned procedures as allowed per study (e.g., surgery for subjects in Cohort 5).
23. Postoperative treatments will begin 6-12 weeks after completion of surgery.
24. End of treatment visit will occur within 7 days after the last dose or decision by the investigator to discontinue subject from treatment. If zolbetuximab is discontinued prior to FLOT, an EOT visit and 30- and 90-day follow-up visits should be conducted relative to both zolbetuximab discontinuation and FLOT discontinuation.
25. Subjects who discontinue or complete all study treatment will enter the post-treatment follow-up period for up to 2 years.
26. Following completion of the post-treatment follow-up period, disease recurrence (or radiographic disease progression) or the start of another anticancer therapy (whichever occurs first), subjects in Cohort 5 will enter the survival follow-up period for the remainder of the 3-year period following last dose of study treatment.

Table 12 Cohort 5 – Zolbetuximab Pharmacokinetic/Immunogenicity Schedule

**Study Day (1 cycle = 2 Weeks)**		**Time (Relative to Dosing in each cycle)**	**Zolbetuximab Pharmacokinetics [1,2]**	**Zolbetuximab Immunogenicity [3]**
Cycles 1-8	Day 1	Predose (0 hour)	X	X
		EOI	X	
Preoperative 30-day Follow-up Visit			X	X
Postoperative (or EOT) 30-day Follow-up [4]			X	X
Postoperative (or EOT) 90-day Follow-up [4]			X	X

EOI: end of infusion; EOT: end of treatment

1. Zolbetuximab pharmacokinetic sampling windows are as below. The date and time of each sample collection will be recorded to the nearest minute.
   - Predose: within 60 minutes prior to zolbetuximab dosing.
   - End of infusion (EOI): within 10 minutes after the end of the zolbetuximab infusion.
2. Unscheduled pharmacokinetic blood samples may be taken at any time during the study to evaluate drug exposure following a safety event.
3. Zolbetuximab immunogenicity predose sampling window: within 60 minutes prior to zolbetuximab dosing.
4. Zolbetuximab follow-up pharmacokinetics will be sampled at zolbetuximab follow-up visits.

Table 13 Cohort 5 – Exploratory Biomarkers Collection Schedule

Study Day (1 cycle = 2 Weeks)		Time (Relative to Dosing in each cycle)	Biomarkers	
			Exploratory Biomarkers (Blood)	Exploratory Biomarkers (Plasma)
Cycle 1	Day 1	Predose	X	X
14-30 days after last preoperative dose				X
Cycle 5	Day 1	Predose		X
Cycle 8	Day 1	Predose		X
Postoperative (or EOT) 30-day Follow-up				X
Postoperative (or EOT) 90-day Follow-up				X
6 months postoperative [1]				X
9 months postoperative				X
12 months postoperative				X
16 months postoperative				X
20 months postoperative				X
24 months postoperative				X

1. Postoperative biomarker collection at months 6, 9, 12, 16, 20 and 24 can be conducted at the same time as the follow-up imaging (every 3 [± 1] months during the first year, then every 4 [± 2] months during the second year in post-treatment follow-up period).

1 INTRODUCTION

Gastric and gastroesophageal junction (GEJ) cancers are among the malignancies with the highest unmet medical need. Gastric cancer-related mortality is the fourth leading cause of cancer death worldwide, even if its incidence has decreased over past decades in different regions of the world [World Health Organization, 2018; Amiri et al, 2011]. On the other hand, the incidence of patients with GEJ adenocarcinoma has increased in recent decades, coinciding with a shift in histological type and primary tumor location [Waddell et al, 2013; Sahin et al, 2008].

In 2017, an estimated 723100 people will die worldwide from gastric cancer [Lederman 2017]. The overall 5-year survival rate for gastroesophageal cancer is about 20% in the US and Europe, despite aggressive standard treatments, which are also associated with substantial side effects [Pennathur et al, 2013; Sahin et al, 2008]. The lack of a major benefit from the various newer generation combination chemotherapy regimens for these cancers has stimulated research into the use of targeted agents such as monoclonal antibodies. Zolbetuximab has been developed with the goal of addressing this medical need.

In the US and Europe, the current standard of care consists of fluoropyrimidine and platinum-based combination chemotherapy regimens with or without a third agent such as docetaxel or epirubicin [National Comprehensive Cancer Network (NCCN), 2017; Waddell et al, 2013; Pasini et al, 2011]. The lack of a major benefit from the various newer generation combination chemotherapy regimens for these cancers has stimulated research into the use of targeted agents such as monoclonal antibodies. Two monoclonal antibodies have received approval in gastric cancer: trastuzumab selectively binds the extracellular domain of human epidermal growth factor receptor 2 (HER2), which is overexpressed in approximately 20% to 30% of gastric tumors [Bang et al, 2009], and ramucirumab specifically binds vascular endothelial growth factor (VEGF) receptor 2 and blocks binding of VEGF receptor ligands VEGF-A, VEGF-C and VEGF-D. Trastuzumab is approved for treatment of HER2 overexpressing metastatic gastric or GEJ adenocarcinoma, while ramucirumab is approved as a single agent or in combination with paclitaxel, for treatment of advanced gastric or GEJ adenocarcinoma, with disease progression on or after prior fluoropyrimidine- or platinum-containing chemotherapy [HERCEPTIN Prescribing Information, 2016; CYRAMZA Prescribing Information, 2017]. These agents prolonged median overall survival (OS) by 4 months or fewer when given alone or in combination with chemotherapy, compared with standard of care cytotoxic chemotherapy [Fuchs et al, 2014; Wilke et al, 2014; Ohtsu et al, 2011; Bang et al, 2010]. Approximately 70% to 80% of patients with metastatic or advanced unresectable gastric and GEJ adenocarcinoma in the first line setting have tumors that are HER2 negative. These patients have an expected median survival of approximately 1 year. Therefore, a significant unmet medical need exists for the first-line treatment of patients with non-HER2 expression metastatic or locally advanced unresectable gastric and GEJ cancers.

In the locoregional advanced, resectable setting, recurrence is common and 5-year survival rates remain low. Multimodal treatment strategies incorporating perioperative chemotherapy

can improve overall survival, and standard of care includes surgery with perioperative fluorouracil, leucovorin or folinic acid, oxaliplatin and docetaxel (FLOT) chemotherapy. However, additional treatment strategies are needed to improve the overall survival rate, which remains low at approximately 31% across all stages [Bang et al, 2019].

1.1 Background

Zolbetuximab (IMAB362) is a genetically engineered, highly purified chimeric (mouse/human IgG1) antibody directed against the tight junction molecule Claudin 18.2 (CLDN18.2). The target is a member of the Claudin family of more than 20 structurally related proteins that are involved in the formation of tight junctions in epithelia and endothelia [Niimi et al, 2001]. Tight junctions, together with adherens junctions and desmosomes, form the apical junctional complex in epithelial and endothelial cellular sheets. Adherens junctions and desmosomes are responsible for the mechanical adhesion between adjacent cells, whereas tight junctions are essential for the tight sealing of the cellular sheets forming a luminary barrier and controlling the paracellular ion flux.

CLDN18.2 is a 27.7 kDa protein with 4 membrane-spanning domains and 2 small extracellular loops [Gunzel & Yu, 2013; Sahin et al, 2008]. Zolbetuximab recognizes the first extracellular domain of CLDN18.2 with high affinity and specificity. Zolbetuximab does not bind to any other claudin family member including the closely related splice variant 1 of CLDN18.1.

CLDN18.2 is a highly cell type specific differentiation antigen that is expressed by differentiated gastric mucosa cells in the pit and base regions of gastric glands. Moreover, CLDN18.2 is not detectable in any other normal cell type of the human body either at transcript level or as protein. Due to this highly selective tissue distribution pattern results in CLDN18.2 expression being strictly confined to a subpopulation of gastric epithelial cells in normal tissue [Sahin et al, 2008].

CLDN18.2 is expressed in a diversity of human cancers and is the dominant isoform in gastroesophageal and pancreatic cancer [Lee et al, 2011]. The expression of CLDN18.2 is retained upon malignant transformation of gastric epithelia and is present in 81% of primary gastric adenocarcinomas. CLDN18.2 expression is frequently detected in diffuse and in intestinal gastric cancers. The CLDN18.2 protein is also localized in lymph node metastases of gastric cancer adenocarcinomas and in distant metastases into the bile duct, lung and especially into the ovary (so-called Krukenberg tumors). Furthermore, over 42% of esophageal adenocarcinomas and 50% to 70% of pancreatic cancers display significant expression of CLDN18.2 as determined by the sponsor and others [Woll et al, 2014; Lee et al, 2011; Sanada et al, 2010; Karanjawala et al, 2008; Sahin et al, 2008].

Zolbetuximab is being developed for the first-line treatment of adult subjects with metastatic or locally advanced CLDN18.2-positive, HER2-negative adenocarcinoma of the stomach and GEJ in combination with platinum- and fluoropyrimidine-based chemotherapy. Zolbetuximab monotherapy is also being evaluated in patients with later-line metastatic gastric and GEJ cancer as monotherapy and in combination with pembrolizumab. The tolerability of zolbetuximab in combination with platinum- and fluoropyrimidine-based

chemotherapy and nivolumab is also being evaluated in adult subjects with metastatic or locally advanced CLDN18.2-positive, HER2-negative adenocarcinoma of the stomach and GEJ in combination.

1.2 Nonclinical and Clinical Data

1.2.1 Nonclinical Data

In vitro studies with CLDN18.2-positive and negative cancer cell lines showed that zolbetuximab binds to the extracellular domain 1 of CLDN18.2 on human gastric cancer cell lines with high relative affinity and selectivity. In vitro assays demonstrated that zolbetuximab mediated an efficient lysis of CLDN18.2-positive cells through antibody-dependent cellular cytotoxicity (ADCC) and complement-dependent cytotoxicity (CDC).

All zolbetuximab-mediated effects are strictly CLDN18.2 antigen-specific.

In biodistribution studies in nude mice with human tumor xenografts, intravenously administered zolbetuximab was retained as well as specifically and strongly enriched in CLDN18.2-positive human xenografts. No or little in vivo binding of zolbetuximab to any other mouse tissues including stomach tissue was observed.

Administration of repeated doses of zolbetuximab to mice bearing CLDN18.2-positive tumors resulted in retardation of tumor growth kinetics in tumor models.

A series of experiments were conducted with CLDN18.2-expressing cell lines derived from NUGC-4 and KATO-III to investigate the effects of combining these chemotherapy agents with zolbetuximab. Combinations of chemotherapy agents used in the treatment of gastric and esophageal cancers, including 5-fluorouracil (5-FU), oxaliplatin and epirubicin (e.g., 5-FU/oxaliplatin, 5-FU/oxaliplatin/epirubicin) and docetaxel augmented zolbetuximab activity. In vitro presensitization of human gastric cancer cells with chemotherapy resulted in an increase in the amount of cell surface of CLDN18.2 and improved zolbetuximab-mediated ADCC and CDC. The combination treatment of zolbetuximab with an anti-mPD-1 antibody significantly increase the antitumor efficacy of either zolbetuximab or the anti-PD-1 antibody alone in an mCLDN18.2-expressing model.

In immunocompetent mice, zolbetuximab in combination with chemotherapy resulted in a pronounced T cell infiltration into the tumors and significant long-term survival benefit over zolbetuximab alone. Most likely this effect was mediated by induction of adaptive T cell immunity, which may have led to a prolonged antitumor effect and immune surveillance.

CLDN18.2 is highly conserved across species and the epitope of zolbetuximab is identical between humans, mice and cynomolgus monkeys. In addition, the binding affinity of zolbetuximab to CLDN18.2 orthologs from mice, humans and cynomolgus monkeys was shown to be comparable, providing sufficient evidence that testing in mice and monkeys covers the potential on-target effects and toxicities of zolbetuximab.

The nonclinical pharmacology studies conducted with zolbetuximab provide sufficient experimental evidence that zolbetuximab depletes CLDN18.2-positive cells via ADCC and

CDC. Cytotoxic drugs were shown to increase CLDN18.2 expression on human cancer cells and to improve the activity of the major mechanism of action (ADCC and CDC). An anti-PD-1 antibody in combination with zolbetuximab showed greater anti-tumor activity than either alone. Hence, the combination of zolbetuximab with first-line chemotherapy, in third line with pembrolizumab, in first line with chemotherapy and nivolumab, and perioperatively with FLOT is being investigated in the clinic.

Safety pharmacology and toxicity of zolbetuximab were assessed in mice and cynomolgus monkeys. In mice, the maximum exposure tested was 300 mg/kg weekly over 13 weeks, and in cynomolgus monkeys, the maximum exposure tested was 100 mg/kg weekly over 4 weeks.

In both species, no target organs of toxicity were identified; however, in monkeys, emesis was observed in a non-dose-related manner. The emesis that was observed in monkeys was not severe and spontaneously resolved despite continued dosing. The emetic potential of zolbetuximab was confirmed in an investigational study in ferrets. This effect is considered to be related to the binding of zolbetuximab to junctional protein, CLDN18.2, in the gastric epithelium. A human tissue cross-reactivity study of zolbetuximab showed that the gastric mucosa was the only tissue with strong membrane staining. However, histological assessment of the gastric tissue in monkeys failed to identify any histopathological lesions.

Besides the findings listed previously, no other zolbetuximab-related adverse effects were observed in any organ, neither clinically, nor macroscopically or histologically upon postmortem analysis.

In summary, the nonclinical data outlined above indicated that zolbetuximab may be evaluated alone as well as in combination with standard chemotherapy and in combination with PD-1 inhibitors for the treatment of CLDN18.2-positive gastroesophageal adenocarcinomas.

1.2.2 Clinical Data

Zolbetuximab has been evaluated in clinical studies as a single agent and in combination with epirubicin, oxaliplatin and capecitabine (EOX) chemotherapy or in combination with immunomodulation therapy (zoledronic acid [ZA] with or without interleukin-2 [IL-2]) for the treatment of adult subjects with CLDN18.2-positive advanced adenocarcinoma of the stomach, esophagus or GEJ.

CLDN18.2 expression was immunohistochemically determined for all subjects enrolled in the clinical studies. Several studies had an enrichment type of design meaning that only patients above a certain threshold of CLDN18.2 positivity in their tumors were eligible for treatment.

To date, completed phase 1 studies include GM-IMAB-001, referred to as first-in-human (FIM), GM-IMAB-001-04 (PILOT), 8951-CL-0104 and 8951-CL-0105. Completed phase 2/2a studies include GM-IMAB-001-02 (MONO) and GM-IMAB-001-03 (FAST).

The phase 3 SPOTLIGHT and GLOW studies are ongoing and have enrolled participants whose tumors are CLDN18.2-positive (≥ 75% of tumor cells demonstrating moderate to

strong membranous CLDN18 staining), HER2-negative tumors for first-line treatment with a combination of zolbetuximab and oxaliplatin, leucovorin or folinic acid and 5-FU (mFOLFOX6) or CAPOX. Both studies met the primary endpoint of PFS and the key secondary endpoint of overall survival based on primary analysis.

Zolbetuximab has been granted orphan drug designation for the treatment of stomach cancer by the EMA and FDA.

1.3 Summary of Key Safety Information for Study Drugs

Current phase 1/2 study status and enrollment are available in the zolbetuximab Investigator's Brochure (IB) (see end-of-text Table 4.1 in the IB). Nausea and vomiting have been confirmed as important identified risks as has hypersensitivity reactions (HSRs), including infusion-related reactions (IRRs). Anemia and neutropenia are considered important potential risks. These important identified risks along with important potential risks, based on observations from the clinical studies, are described in Section 5.2 of the Investigator's Brochure (IB). Expected adverse drug reactions, including Reference Safety Information (RSI) used for expedited health authority reporting are described in Appendix 1 of the IB.

One patient from an ongoing phase 2 study (monotherapy arm) experienced grade 4 acute coronary syndrome, grade 4 cardiac arrest, grade 4 posterior reversible encephalopathy syndrome (PRES) and grade 3 pulmonary embolism, 22 days after the first infusion of zolbetuximab monotherapy. The patient had a medical history significant for uncontrolled hypertension, asthenia, and anemia. The patient recovered from these events that were deemed serious and a possible causal relationship to zolbetuximab could not be excluded. Refer to the IB for additional details.

In clinical studies, adverse reactions with nausea and/or vomiting and HSRs up to National Cancer Institute-Common Terminology Criteria for Adverse Events (NCI-CTCAE) grade 3 were reported. Subjects receiving zolbetuximab should receive prophylactic antiemetic medications, but do not need to be premedicated for prevention of HSRs and IRRs; however, subjects should be closely monitored for IRRs to facilitate early identification and management. In the case of zolbetuximab-induced nausea, vomiting or hypersensitivity, the infusion rate of zolbetuximab may be reduced or the infusion may be paused or discontinued based on investigator's clinical judgment about severity of toxicity and local standard of care.

Detailed information on the toxicities associated with mFOLFOX6 can be found within Section 4.8 of the summary of product characteristics (SPC) for each component. Potential overlapping toxicities during treatment with zolbetuximab in combination with mFOLFOX6 include nausea and vomiting.

Detailed information on the toxicities associated with FLOT can be found within Section 4.8 of the SPC for each component. Potential overlapping toxicities during treatment with zolbetuximab in combination with FLOT include nausea, vomiting and hypersensitivity reactions.

1.4 Efficacy

1.4.1 Efficacy Results from Study GM-IMAB-001-02 (MONO)

In the MONO study, efficacy analyses included subjects who received zolbetuximab at least once (Full Analysis Set [FAS]) 300 mg/m^2 (3 subjects) and 600 mg/m^2 (40 subjects). The best overall confirmed response for FAS subjects in the zolbetuximab 600 mg/m^2 dose group was partial response (PR) in 4 subjects (9.3%) that ranged in duration from 43 through 1037 days (GM-IMAB-001-02, Listing 13.2.6.4). The best overall confirmed response was stable disease in 6 subjects (14.0%) and progressive disease (PD) in 31 subjects (72.1%) (GM-IMAB-001-02, Table 12.3.7.1). No subject achieved complete response (CR). Median progression-free survival (PFS) in the FAS was 10 weeks (95% confidence interval [CI]: 9.1, 10.1 weeks) (GM-IMAB-001-02, Table 12.3.3.1).

1.4.2 Efficacy Results from Study GM-IMAB-001-03 (FAST)

Treatment groups in the FAST study are referred to as EOX, EOX + zolbetuximab (EOX plus zolbetuximab 600 mg/m^2 once every 3 weeks, with a loading dose of 800 mg/m^2 in cycle 1) and EOX + zolbetuximab (EOX plus zolbetuximab 1000 mg/m^2 once every 3 weeks).

Primary Analysis

In the FAST study, efficacy analyses of all randomized subjects were included the intent-to-treat (ITT) set and included subjects randomized to EOX only (85 subjects), EOX + Changed to zolbetuximab (79 subjects) and EOX + Changed to zolbetuximab (88 subjects). Additional analyses were completed for the FAS, defined as randomized subjects who received at least 1 dose of any study medication (epirubicin, oxaliplatin, capecitabine or zolbetuximab). The FAS differed from the ITT set (all randomized subjects) by 6 subjects all of whom discontinued the study early without death or post baseline tumor assessment. The reasons for early discontinuation of these subjects included protocol violation (1 subject), physician decision (1 subject), withdrawal by subject (2 subjects) and adverse event (AE; 1 subject was anemic and another had a deep vein thrombosis) (GM-IMAB-001-03 Clinical Study Report, Listing 13.2.1).

Progression-free Survival

<u>PFS Based on Central Independent Review (Kaplan-Meier Model, Intent-to-Treat)</u>

A PFS event was reported for 72.9% of the EOX subjects vs 53.2% of subjects in the EOX + zolbetuximab group and 55.7% in the EOX + zolbetuximab group (ITT set). The addition of zolbetuximab to EOX led to a statistically significant prolongation of PFS, both for the lower zolbetuximab dose (hazard ratio [HR] 0.45, P < 0.0005) and the higher zolbetuximab dose (HR 0.57, P = 0.0114). Median PFS was 7.5 months in the EOX + zolbetuximab arm and 7.1 months in the EOX + zolbetuximab arm vs 5.3 months in the EOX arm, representing a median PFS prolongation by 2.2 and 1.8 weeks, respectively.

Using the FAS, the Kaplan-Meier curves of PFS for EOX vs EOX + zolbetuximab and EOX vs EOX + zolbetuximab separated early and remained separated over the full observation

period, with a noticeably more pronounced treatment benefit for the EOX + zolbetuximab regimen.

<u>Overall Survival (Kaplan-Meier, ITT)</u>

The OS event rate was highest in the EOX group (88.2%) vs 78.4% in the EOX + zolbetuximab group and 68.4% in the EOX + zolbetuximab group. The addition of zolbetuximab 600 mg/m^2 led to a statistically significant prolongation in OS (HR 0.52, P < 0.0005). Median OS was 13.0 months in the EOX + zolbetuximab arm vs 8.3 months in the EOX arm, representing an increase in median OS by approximately 4.7 months. In the EOX + zolbetuximab arm, median OS was 9.6 months and hence, numerically longer than in the EOX arm. However, the difference between the groups did not reach statistical significance. The difference in OS between the 2 zolbetuximab doses was statistically significant (P = 0.0406) (GM-IMAB-001-03 Clinical Study Report, Table 12.3.1.1.5.3).

No major imbalances were seen between the treatment groups in the subsequent use of any anticancer therapy (EOX: 38.1%; EOX + zolbetuximab: 40.3%; EOX + zolbetuximab: 34.1%) and any chemotherapy (EOX: 34.5%; EOX + zolbetuximab: 39.0%; EOX + zolbetuximab: 29.4%) (GM-IMAB-001-03 Clinical Study Report, Tables 12.2.1.1.5.1 and 12.2.1.5 [Safety-evaluable set]).

<u>OS (FAS)</u>

Results for OS based on the FAS were virtually identical to the ITT analyses, due to the fact that the FAS and ITT sets differ by a total of 6 subjects who discontinued the study early.

<u>Best Overall Tumor Response by Central Independent Review</u>

Based on confirmed responses, the objective response rate (ORR) was 38.0% in the EOX + zolbetuximab arm, 29.5% in the EOX + zolbetuximab arm and 24.7% in the EOX arm. This included 10.1% of subjects with a CR in the EOX + zolbetuximab arm, 4.5% in the EOX + zolbetuximab arm and 3.5% in the EOX arm.

1.4.3 Efficacy of mFOLFOX6

Subjects in this study will receive mFOLFOX6 (a combination 5-FU, folinic acid and oxaliplatin), which is an accepted standard-of-care treatment for subjects with locally advanced unresectable metastatic gastric or gastroesophageal cancer [NCCN 2017].

1.4.4 Efficacy of mFOLFOX6 and Nivolumab

Subjects in this study will receive mFOLFOX6 (a combination 5-FU, folinic acid and oxaliplatin) in combination with Nivolumab. This is based on data from the Checkmate 649 comparing mFOLFOX6/XELOX to mFOLFOX6/XELOX with nivolumab. The addition of nivolumab to standard of care increased mPFS (7.7 vs. 6.1 mos, HR = 0.68, P < 0.0001) and mOS (14.4 vs. 11.1 mos, HR = 0.71, P < 0.0001) for subjects with a combined positive score (CPS) > 5. In addition, a benefit was seen with the addition of nivolumab for CPS > 1 with a mPFS (7.3 vs. 6.9 mos, HR = 0.74) and mOS (14.0 vs. 11.3 mos, HR = 0.77, P < 0.0001) [Moehler et al, 2020].

1.4.5 Efficacy of FLOT

Subjects in Cohort 5 will receive FLOT (a combination of docetaxel, oxaliplatin, leucovorin and 5-FU), which is an accepted standard of care treatment for subjects with locoregional gastric or gastroesophageal cancer. In the FLOT4 study, perioperative FLOT improved overall survival compared with ECF/ECX (epirubicin and cisplatin plus fluorouracil or capecitabine) in locally advanced, resectable gastric or GEJ adenocarcinoma [Al-Batran et al, 2019].

1.5 Risk Benefit Assessment

Zolbetuximab is an investigational agent in the treatment of CLDN18.2-positive, metastatic or locally advanced unresectable gastric or GEJ adenocarcinoma and locoregional gastric or GEJ adenocarcinoma both as single agent and in combination with standard of care chemotherapy and/or immunotherapy as first-line treatment or in later line setting.

Based on clinical efficacy and safety data from the completed and ongoing zolbetuximab clinical studies and supportive preclinical pharmacological studies, there is potential of achieving clinically relevant benefit in the later line setting as a single agent or in combination with oxaliplatin and fluoropyrimidine-based chemotherapy in a first-line setting. The identified risks are nausea, vomiting and hypersensitivity reactions (HSRs; including IRRs). Potential risks include neutropenia and anemia. Based on currently available clinical data, zolbetuximab alone and in combination was tolerated from a safety perspective and most observed AEs have been considered manageable. Non-clinical toxicity data were supportive of the clinical findings. Overall the potential benefits of zolbetuximab, zolbetuximab in combination with mFOLFOX6 (with or without nivolumab), zolbetuximab in combination with pembrolizumab and zolbetuximab in combination with FLOT outweigh the identified and potential risks; allowing for further clinical development in subjects with CLDN18.2-positive, metastatic or locally advanced unresectable gastric or GEJ adenocarcinoma or locoregional resectable gastric or GEJ adenocarcinoma who meet protocol eligibility criteria.

2 STUDY OBJECTIVE(S), DESIGN AND ENDPOINTS

2.1 Study Objectives

2.1.1 Primary Objective

The primary objective is to determine the ORR of zolbetuximab as a single agent as assessed by an independent central reader.

2.1.2 Secondary Objectives

- To evaluate the pharmacokinetics of zolbetuximab as a single agent, in combination with mFOLFOX6, in combination with pembrolizumab, in combination with mFOLFOX6 and nivolumab, and in combination with FLOT
- To evaluate pharmacokinetics of oxaliplatin and 5-FU in combination with zolbetuximab

- To assess the safety and tolerability of zolbetuximab as a single agent, in combination with mFOLFOX6 (with or without nivolumab), in combination with pembrolizumab, and in combination with FLOT
- To assess the immunogenicity of zolbetuximab as a single agent, in combination with mFOLFOX6 (with or without nivolumab), in combination with pembrolizumab, and in combination with FLOT
- To evaluate health-related quality of life (HRQoL)
- To assess ORR of zolbetuximab in combination with pembrolizumab and in combination with mFOLFOX6 as assessed by an independent central reader
- To assess ORR of zolbetuximab as a single agent, in combination with mFOLFOX6 (with or without nivolumab) based on investigator assessment
- To evaluate disease control rate (DCR), duration of response (DOR) and progression-free survival (PFS) of zolbetuximab as a single agent and in combination with mFOLFOX6 based on independent central reader assessment
- To evaluate DCR, DOR and PFS of zolbetuximab as a single agent and in combination with mFOLFOX6 (with or without nivolumab) based on investigator assessment
- To assess OS of zolbetuximab as a single agent, in combination with mFOLFOX6 and nivolumab, and in combination with FLOT
- Cohort 5: To evaluate antitumor activity of zolbetuximab and FLOT as measured by radiological response (restaging) and pathological response (ypTNM)
- Cohort 5: To assess disease-free survival (DFS) of zolbetuximab in combination with FLOT
- Cohort 5: To assess minimal residual disease and disease recurrence

2.1.3 Exploratory Objective

- To evaluate potential genomic and/or other biomarkers that may correlate with treatment outcome of zolbetuximab as a single agent, in combination with mFOLFOX6 (with or without nivolumab), and in combination with FLOT
- To assess the effects of zolbetuximab as a single agent and in combination with mFOLFOX6 (with or without nivolumab) on CLDN18.2 expression
- To assess the immunomodulatory effects of zolbetuximab as a single agent, in combination with mFOLFOX6 (with or without nivolumab), and in combination with FLOT
- To evaluate the pharmacokinetics of pembrolizumab in combination with zolbetuximab
- To evaluate the pharmacokinetics of nivolumab in combination with zolbetuximab and mFOLFOX6

2.2 Study Design and Dose Rationale

2.2.1 Study Design

This is a phase 2, open-label, multi-arm, non-randomized, multicenter study to assess the antitumor activity of zolbetuximab, an IgG1 chimeric monoclonal antibody directed against CLDN18.2, in subjects with metastatic or locally advanced unresectable gastric or GEJ adenocarcinoma and locoregional gastric or GEJ adenocarcinoma whose tumors are

CLDN18.2-positive. For each cohort, the study consists of the following periods: pre-screening, screening, treatment and follow-up for radiographic disease progression (or post-treatment follow-up for disease recurrence, which will be conducted for Cohort 5). In addition, there will be a survival follow-up period for Cohorts 1A, 4B and 5 subjects only. Up to 25 centers located in North America, Europe and Asia Pacific will participate in this study for Cohorts 1-4. At least 50% of the subjects enrolled in Cohorts 1A and 2 must be from the US or Europe to ensure the study is regionally balanced. Up to 5 centers in North America and Japan will participate in this study for Cohort 5.

Pre-Screening:

After the pre-screening informed consent has been obtained, an archival tumor sample will be submitted for each subject to determine CLDN18.2 status (in addition to HER2 status for Cohorts 2 and 4 if required). If available, the most recent tumor sample is preferred. If archival tumor tissue is insufficient or unavailable for pre-screening, the subject will forego the pre-screening period and directly enter the screening period and biopsy will be performed to obtain the tumor sample for eligibility needed to determine CLDN18.2 status and HER2 status as required. While not required for eligibility, programmed death ligand 1 (PD-L1) status will be determined based on the tumor sample submitted.

Screening and Enrollment:

Enrollment for Cohorts 1A, 2, 3A and 4A has been completed. Enrollment for Cohorts 4B and 5 is ongoing. After the screening main informed consent has been obtained, screening will take place up to 28 days prior to subject enrollment on cycle 1 day 1.

The following screening assessments may be repeated within the 28-day screening period:

- ECG, laboratory assessments and vital signs

Subjects do not need to be “screen failed” in Interactive Response Technology (IRT) and re-entered in screening with a new subject identification as long as the subject is enrolled within the 28-day window from signing of the informed consent.

If more than 28 days elapses from the date of signing the informed consent, the subject must be screen failed in IRT. A new consent must be signed and the subject entered into screening with a new subject ID. Subjects may only be rescreened once.

Treatment Period:

Cohort 1A:

Subjects in Cohort 1A will be treated with zolbetuximab on a 21-day cycle in which zolbetuximab will be administered as a single agent every 3 weeks until disease progression, toxicity requiring cessation, start of another anti-cancer treatment or other treatment discontinuation criteria are met. Cohort 1A will enroll approximately 20 subjects with measurable disease at baseline. Imaging in Cohort 1A will be performed every 6 weeks from cycle 1 day 1 (C1D1) for the first 24 weeks and then every 12 weeks thereafter.

Cohort 2:

Subjects in Cohort 2 will be treated with zolbetuximab and mFOLFOX6 on a 42-day cycle in which zolbetuximab is administered on days 1 and 22, and mFOLFOX6 is administered on days 1, 15 and 29; however, for the first cycle, zolbetuximab will be administered on day 3 (instead of day 1) to allow for pharmacokinetic collection. Subjects will receive up to 12 mFOLFOX6 treatments (4 cycles).

Beginning at cycle 5, subjects may continue on 5-FU and leucovorin or folinic acid along with zolbetuximab for the remainder of the study per investigator's discretion. Imaging in Cohort 2 will be performed every 9 weeks from C1D1 for the first 54 weeks, and then every 12 weeks thereafter.

Cohort 3A:

Subjects in Cohort 3A will be treated with zolbetuximab and pembrolizumab on a 21-day cycle in which zolbetuximab and pembrolizumab are administered on day 1 of each cycle. This cohort is to determine safety and tolerability. Imaging in Cohort 3A will be performed every 6 weeks for the first 24 weeks and then every 12 weeks thereafter.

The tolerability and safety of zolbetuximab in combination with pembrolizumab will be evaluated during the 3-week dose-limiting toxicity (DLT) assessment period. Initially, 3 subjects will be enrolled into Cohort 3A and receive the zolbetuximab loading dose of 800 mg/m^2 in combination with pembrolizumab 200 mg on C1D1, followed by zolbetuximab 600 mg/m^2 in combination with pembrolizumab 200 mg on day 1 of each subsequent cycle. Any of the zolbetuximab and/or pembrolizumab related AEs specified as the DLTs will be assessed.

Cohort 4:

In Cohort 4A, the tolerability and safety of zolbetuximab in combination with mFOLFOX6 and nivolumab will be evaluated during the 2-week DLT assessment period. Initially, 3 subjects will be enrolled into Cohort 4A and receive the zolbetuximab loading dose of 800 mg/m^2 in combination with nivolumab 240 mg and mFOLFOX6 on cycle 1 day 1, followed by zolbetuximab 400 mg/m^2 in combination with nivolumab 240 mg and mFOLFOX6 every 2 weeks [days 15 and 29] (1 cycle = 6 weeks). Zolbetuximab will be administered first, followed by the nivolumab and then mFOLFOX6. Any of the zolbetuximab, mFOLFOX6 and/or nivolumab-related AEs specified as the DLTs will be assessed. Subjects will receive up to 12 mFOLFOX6 treatments (4 cycles).

In Cohort 4B, subjects will be treated with the combination of zolbetuximab, mFOLFOX6 and nivolumab at the dose deemed tolerable in Cohort 4A. Subjects will receive up to 12 mFOLFOX6 treatments (4 cycles).

For Cohorts 4A and 4B, beginning at cycle 5, subjects may continue on 5-FU and leucovorin or folinic acid along with zolbetuximab and nivolumab for the remainder of the study per investigator's discretion. Imaging in Cohort 4A and 4B will be performed every 8 weeks for the first 56 weeks, and then every 12 weeks thereafter.

Cohort 5:

Subjects in Cohort 5 will be treated with zolbetuximab in combination with FLOT for a total of eight 2-week cycles. Four cycles will be administered preoperatively and 4 cycles will be administered postoperatively. The tolerability and safety of treatment with zolbetuximab in combination with FLOT will be evaluated for DLTs during the 2-week DLT assessment period following the first preoperative treatment with zolbetuximab. Any of the zolbetuximab and/or FLOT related AEs considered DLTs will be assessed.

Preoperative Treatment

Subjects will be enrolled into Cohort 5 and receive the zolbetuximab loading dose of 800 mg/m^2 on cycle 1 day 1, followed by FLOT on cycle 1 day 2. For cycles 2-4, subjects may receive zolbetuximab 400 mg/m^2 in combination with FLOT, dosed on day 1 of each cycle. When dosing both treatments on the same day, zolbetuximab should be administered first, followed by FLOT. However, dosing may continue to be split over 2 days with zolbetuximab administration on day 1 and FLOT administration on day 2, based on investigator judgment. Subjects should undergo surgery within 4-8 weeks after completion of 4 cycles of preoperative zolbetuximab + FLOT.

If the zolbetuximab 800 mg/m^2 loading dose is assessed as not tolerable based on the DLT assessment, then 3 to 6 subjects will be treated with zolbetuximab 600 mg/m^2 loading dose on cycle 1 day 1 in combination with FLOT on cycle 1 day 2, followed by zolbetuximab 400 mg/m^2 in combination with FLOT on day 1 (or zolbetuximab on day 1 with FLOT on day 2) of cycles 2-4 (2-week cycles).

Postoperative Treatment

Subjects who have undergone complete surgical resection (R0) should receive 4 cycles of postoperative treatment with zolbetuximab + FLOT, starting 6-12 weeks after surgery.

Subjects will receive the zolbetuximab loading dose of 800 mg/m^2 on cycle 5 day 1, followed by FLOT on cycle 5 day 2. For subjects who experience a DLT during preoperative treatment on the loading dose of 800 mg/m^2, the postoperative loading dose may be reduced to 600 mg/m^2 based on the judgment of the investigator. However, for subjects who experience a DLT during preoperative treatment on a loading dose of 600 mg/m^2, the postoperative loading dose may not be omitted, but dose reduction/modification can be considered for FLOT based on investigator judgment.

For cycles 6-8, subjects may receive zolbetuximab 400 mg/m^2 in combination with FLOT dosed on day 1 of each cycle. When dosing both treatments on the same day, zolbetuximab will be administered first, followed by FLOT. However, dosing may continue to be split over 2 days with zolbetuximab administration on day 1 and FLOT administration on day 2, based on investigator judgment.

Tolerability evaluation meeting (TEM): Cohorts 3A, 4A and 5

The tolerability evaluation meeting (TEM) will be held after the end of the 3-week (Cohort 3A) or 2-week (Cohorts 4A and 5) DLT assessment period for the first 3 or 6 total

DLT-evaluable subjects enrolled in a cohort. The sponsor will comprehensively assess the data (including AEs reported for subjects who are unevaluable for DLTs) generated from the cohort to be assessed, and discuss the tolerability of the current dose with the investigators at the TEM. Based on the results of the discussion, the sponsor will decide on the tolerability of the current dose level.

For Cohort 3A, it has been determined that zolbetuximab 800/600 mg/m^2 in combination with pembrolizumab 200 mg is tolerable.

For Cohort 4A, it has been determined that zolbetuximab 800/400 mg/m^2 in combination with mFOLFOX6 and nivolumab is tolerable.

For Cohort 5, the starting dose of the preoperative combination therapy is zolbetuximab 800 mg/m^2 loading dose on cycle 1 day 1 in combination with FLOT on cycle 1 day 2, followed by zolbetuximab 400 mg/m^2 in combination with FLOT on day 1 (or zolbetuximab on day 1 with FLOT on day 2) of cycles 2-4 (2-week cycles).

- If zolbetuximab 800 mg/m^2 loading dose is assessed as tolerable based on the DLT assessment, then Cohort 5 may proceed to complete enrollment.
- If zolbetuximab 800 mg/m^2 loading dose is assessed as <u>not</u> tolerable based on the DLT assessment, then 3 to 6 subjects will be treated with zolbetuximab 600 mg/m^2 loading dose on cycle 1 day 1 in combination with FLOT on cycle 1 day 2, followed by zolbetuximab 400 mg/m^2 in combination with FLOT on day 1 (or zolbetuximab on day 1 with FLOT on day 2) of cycles 2-4 (2-week cycles).
- If zolbetuximab 600 mg/m^2 loading dose, based on the DLT assessment, is assessed as:

  A) Tolerable, then:
    - Cohort 5 may proceed to complete enrollment, OR
    - Re-escalation to 800 mg/m^2 loading dose may occur based on the Bayesian Optimal Interval Design [Liu & Yuan, 2015] criteria shown in the Dose Limiting Toxicities section below.

  B) Not tolerable, then:
    - Cohort 5 will cease enrollment.

In following the recommendations by the Bayesian Optimal Interval Design, the sponsor may choose a more conservative pathway based on the safety evaluation conducted regularly, as well as the totality of safety data available. Based on these, the sponsor may decide to further assess safety on a lower loading dose level and enroll additional subjects.

<u>Dose Limiting Toxicities:</u>

Quantitative assessment of DLTs will be performed according to the criteria shown in Table 14 referring to the concept of Bayesian Optimal Interval Design [Liu & Yuan, 2015] with the target DLT rate of 33% with the maximum number of subjects limited to 6 in each dose level.

- 0 of 3 initial evaluable subjects experience DLTs: If this is not the highest available dose level, 3 subjects may be enrolled at the next available higher dose level. If this is the

highest available dose level, this dose may be considered tolerable based on the totality of the data.

- 1 of 3 initial evaluable subjects experience DLTs: Additional 3 evaluable subjects will be enrolled at the current dose level and the assessment is continued.
  - Less than 2 of the 6 evaluable subjects experience DLTs: If this is not the highest available dose level, 3 subjects may be enrolled at next available higher dose level. If this is the highest available dose level, this dose may be considered tolerable based on the totality of the data.
  - 2 of 6 evaluable subjects experience DLTs: The tolerability of this dose level will be determined based on a comprehensive assessment of the incidence of DLTs and other safety data at the TEM (the totality of the data).
  - 3 of 6 evaluable subjects experience DLTs: If this is not the lowest available dose level, 3 subjects will be enrolled at next available lower dose level. If this is the lowest available dose level, then this dose will not be considered tolerable.
  - 4 or more of 6 evaluable subjects experience DLTs: If this is not the lowest available dose level, 3 subjects will be enrolled at the next available lower dose level, and the current dose level will be eliminated from the available dose levels. If this is the lowest available dose level, this dose will not be considered tolerable.
- 2 of 3 initial evaluable subjects experience DLTs: If this is not the lowest available dose level, 3 subjects will be enrolled at the next available lower dose level and this dose level may be considered available for re-escalation. If this is the lowest available dose level, this dose will not be considered tolerable.
- 3 of 3 initial evaluable subjects experience DLTs: If this is not the lowest available dose level, 3 subjects will be enrolled at the next lower dose level and the current dose level will be eliminated from the available dose levels. If this is the lowest available dose level, this dose will not be considered tolerable.

Table 14 Recommended Action Based on the Number of Subjects for DLT Assessment

	**Number of Subjects for DLT Assessment**			
Recommended Action by Bayesian Criteria				
Number of Subjects for DLT Assessment	3	4*	5*	6
If the number of subjects with DLTs is equal to the number in the cell to the right or less:<br>1) If this is not the highest available dose level, 3 subjects may be enrolled at next available higher dose level.<br>2) If this is the highest available dose level, this dose level is considered tolerable.	0	1	1	1
If the number of subjects with DLTs is equal to the number in the cell to the right and:<br>1) If the current dose level has reached 3 subjects, enroll additional 3 subjects at this dose level.<br>2) If the current dose level has reached 6 subjects, the tolerability of this dose level will be determined based on the totality of the data.	1	-	-	2
Table continued on next page				

	Number of Subjects for DLT Assessment			
If the number of subjects with DLTs is equal to the number in the cell to the right and:<br>1) If this is not the lowest available dose level, enroll 3 subjects at the next available lower dose level. This dose level may be considered available for re-escalation.<br>2) If this is the lowest available dose level, this dose level will not be considered tolerable.	2	2	2 or 3	3
If the number of subjects with DLTs is the number in the cell to the right or more:<br>1) If this is not the lowest available dose level, enroll 3 subjects at the next available lower dose level, and this dose will be eliminated from the available dose levels for re-escalation.<br>2) If this is the lowest available dose level, this dose level will not be considered tolerable.	3	3	4	4

* In case the number of subjects for dose DLT assessment is 4 or 5, it is considered exceptional, including, for example, if informed consent is obtained from more than 3 subjects in the process of enrolling 3 subjects in the 1st dose level. In such a case, these subjects will be handled as subjects for DLT assessment in the 1st cohort by deciding that the planned cohort size has been modified from 3 to 4 or 5 subjects.

A DLT is defined as any of the following AEs (graded using NCI-CTCAE version 4.03) or laboratory findings that the investigator (or sponsor) cannot clearly attribute to a cause other than study drugs (zolbetuximab, mFOLFOX6, pembrolizumab, nivolumab and FLOT) occurring during the 3-week (Cohort 3A) or 2-week (Cohorts 4A and 5) DLT assessment period:

- Grade 4 neutropenia (afebrile)
- Grade ≥ 3 febrile neutropenia
- Grade 4 thrombocytopenia
- Grade 3 thrombocytopenia accompanied by bleeding that requires any transfusion (platelets or red blood cells)
- Grade 4 anemia
- Grade 3 anemia requiring red blood cell transfusion
- Grade ≥ 3 non-hematologic AE
  (NOTE: Grade 3 nausea, vomiting, diarrhea, fatigue, or abdominal pain that improves to ≤ grade 2 with appropriate treatment within 7 days will not be assessed as a DLT)
- Grade ≥ 2 pneumonitis
- Grade ≥ 2 encephalopathy or meningitis
- Guillain-Barré syndrome or myasthenic syndrome/myasthenia gravis
- Aspartate aminotransferase (AST) or alanine aminotransferase (ALT) > 5 × upper limit of normal (ULN; grade ≥ 3) in subjects without liver metastases
- AST or ALT > 8 × ULN in subjects with liver metastases
- AST or ALT > 3 × ULN and total bilirubin > 2 × ULN (in subject with Gilbert syndrome: AST or ALT > 3 × ULN and direct bilirubin > 1.5 × ULN) in subjects without liver metastases
- Total bilirubin > 3 × ULN (grade ≥ 3)
- Amylase or lipase > 2 × ULN (grade ≥ 3)

- Grade ≥ 3 infusion-related reaction (other than nausea, vomiting, or abdominal pain) including hypersensitivity reactions and reactions with features of anaphylaxis
- Grade 5 toxicity

Replacement of Subjects:

In Cohorts 3A, 4A and 5, subjects who meet any of the following criteria during the 3-week or 2-week DLT assessment period, respectively, will be considered unevaluable for DLT and will be replaced by another subject:

- For Cohorts 3A and 4A, a subject without a DLT who receives less than the planned dose on cycle 1 day 1, or does not complete cycle 1 evaluation for a reason other than DLT (e.g., consent withdrawal)
- For Cohort 5, a subject without a DLT who receives less than the planned dose of zolbetuximab on cycle 1 day 1 and/or of FLOT by cycle 1 day 2, or does not complete cycle 1 evaluation for a reason other than DLT (e.g., consent withdrawal)

Tolerability Evaluation for Japanese Subjects:

Cohort 3: Tolerability of zolbetuximab in combination with pembrolizumab in Japanese subject(s) will be evaluated in the Cohort 3A DLT assessment. Quantitative assessment of any tolerability issues observed in the 3-week assessment period will be performed according to the criteria referring to the Bayesian Optimal Interval Design with the target rate of 0.33.

Cohort 4: Tolerability of zolbetuximab in combination with mFOLFOX6 and nivolumab in Japanese subject(s) will be evaluated in Cohort 4B, if Japanese subjects are not enrolled in the Cohort 4A DLT assessment. Tolerability and safety will be evaluated during a 2-week assessment period for one Japanese subject enrolled in Cohort 4B (if no DLT is observed in Cohort 4A) or 3 Japanese subjects (if 1 or more DLTs are observed in Cohort 4A). The Japanese subject(s) will be assessed for tolerability in Cohort 4B, using the same DLT criteria as those used for the Cohort 4A DLT assessment, but the subject(s) will not be part of the formal DLT assessment already completed in Cohort 4A. Quantitative assessment of any tolerability issues observed in the 2-week assessment period will be performed according to the criteria referring to the Bayesian Optimal Interval Design with the target rate of 0.33. Additional Japanese subjects may be enrolled in Cohort 4B, by up to 6 evaluable Japanese subjects, to continue the tolerability assessment. The details of tolerability evaluation procedure in Cohort 4B will be documented in the Japan Tolerability Guidelines.

Cohort 5: If Japanese subjects are enrolled and assigned to Cohort 5, a separate Japanese tolerability evaluation at the same dose level will not occur because the tolerability of Japanese subjects will have been evaluated, as well as non-Japanese subjects.

Cohorts 1-4 Discontinuation:

Following discontinuation from zolbetuximab, subjects will have a study drug discontinuation visit and safety follow-up visits 30 days and 90 days after their last dose of zolbetuximab. For subjects in Cohort 2, following mFOLFOX6 (all components) discontinuation, Cohort 3 following pembrolizumab discontinuation or Cohort 4 following

discontinuation of mFOLFOX6 (all components) and/or nivolumab, subjects will have a study drug discontinuation visit and safety follow-up visits 30 and 90 days after their last dose of mFOLFOX6, pembrolizumab or nivolumab. If mFOLFOX6, pembrolizumab, or nivolumab are discontinued at the same time as zolbetuximab, 1 discontinuation visit for both zolbetuximab and mFOLFOX6 or zolbetuximab and pembrolizumab or zolbetuximab and nivolumab may be performed.

If a subject discontinues mFOLFOX6 (or components of mFOLFOX6), pembrolizumab or nivolumab due to any reason other than radiographic progression as confirmed by the independent central reader (Cohort 4 only: by the investigator), they may continue on zolbetuximab at the discretion of the investigator provided that all of the following have been met:

- The subject completed at least 1 cycle (42 days [3 treatments]) of mFOLFOX6 (with or without nivolumab) or 2 cycles of pembrolizumab (42 days) treatment;
- The subject will not receive or has not received other chemotherapy;
- And in the investigator’s opinion the subject continues to derive clinical benefit with acceptable toxicity.

Subjects should continue to follow the Study Treatment Period schedule of assessments.

Cohort 5 Discontinuation

Zolbetuximab and FLOT will be discontinued after completion of the 4 planned postoperative cycles, with an end of treatment visit taking place up to 7 days after treatment discontinuation. Additionally, subjects will have safety follow-up visits at 30 and 90 days after completion of postoperative treatment or after early discontinuation, if applicable. If zolbetuximab and FLOT are discontinued separately, the safety follow-up visits should take place relative to each treatment discontinuation.

Follow-up Period for Radiographic Disease Progression (Cohorts 1A, 2 and 4 only)

If a subject discontinues all study treatment prior to radiographic disease progression, the subject will enter the post-treatment follow-up period and continue to undergo imaging assessments until radiographic disease progression or the subject starts another anti-cancer treatment, whichever occurs first.

Post-Treatment Follow-up Period for Disease Recurrence (Cohort 5 only)

If a subject discontinues or completes all study treatment, the subject will enter the post-treatment follow-up period for up to 2 years. Subjects will undergo imaging assessments every 3 months (± 1 month) during the first year, and then every 4 months (± 2 months) or according to local guidelines/standard clinical practice during the second year until disease recurrence (or radiographic disease progression) or until the subject starts another anticancer treatment, whichever happens first. Subjects will also have exploratory biomarker samples collected at cycle 1 day 1, 14-30 days after last preoperative dose, cycle 5 day 1 and cycle 8

day 1, postoperative 30-day and 90-day follow-up, and at 6, 9, 12, 16, 20 and 24 months from the date of surgery.

Survival Follow-up Period (Cohorts 1A and 4B only)

Following radiographic disease progression or the start of another anti-cancer therapy (whichever occurs first), subjects in Cohorts 1A and 4B will enter the survival follow-up period and be followed every 12 weeks. Survival follow-up will be performed via telephone.

Survival Follow-up Period (Cohort 5 only)

Following completion of the post-treatment follow-up period, disease recurrence (or radiographic disease progression) or the start of another anticancer therapy (whichever occurs first), subjects in Cohort 5 will enter the survival follow-up period for the remainder of the 3-year period following last dose of study treatment. Survival follow-up will be performed via telephone every 3 months.

2.2.2 Dose Rationale

The dose of zolbetuximab in this study is an 800 mg/m^2 loading dose followed by 600 mg/m^2 every 3 weeks as a single agent for Cohort 1A, in combination with mFOLFOX6 for Cohort 2, and in combination with pembrolizumab for Cohort 3A. This dose and schedule of zolbetuximab (800/600 mg/m^2 every 3 weeks) were chosen based on the observed data from the GM-IMAB-001-03 (FAST) study. In the GM-IMAB-001-03 (FAST) study, addition of zolbetuximab 800/600 mg/m^2 every 3 weeks to EOX (Arm 2) demonstrated a statistically significant and clinically meaningful improvement on PFS and OS in subjects with CLDN18.2-positive advanced gastric/GEJ cancer compared to EOX (Arm 1). The mean serum trough concentration of zolbetuximab was maintained above the targeted value of 50 μg/mL (based on half maximal effective concentration [EC_{50}] of in vitro ADCC and CDC activities) following 800/600 mg/m^2 every 3 weeks administration. A higher zolbetuximab dose (1000 mg/m^2 every 3 weeks) in combination with EOX was added 18 months later (with the allocation ratio of 1:1:7) as Arm 3 in the FAST study to evaluate safety and efficacy at the higher dose. However, this arm had less treatment benefit compared to Arm 2. Although there were numerical differences observed in some demographics and baseline characteristics, the sample size was not sufficient to evaluate and confirm the impact of such differences; therefore, the reason for underperformance in Arm 3 remains inconclusive.

In Cohort 4, zolbetuximab will be dosed at 800 mg/m^2 in combination with nivolumab and mFOLFOX6 on C1D1, followed by subsequent doses of 400 mg/m^2 every 2 weeks in combination with nivolumab and mFOLFOX6. Based on the preliminary pharmacokinetics analysis, 800/400 mg/m^2 every 2 weeks will provide similar zolbetuximab C_{trough} as what was observed in the Arm 2 (800/600 mg/m^2 every 3 weeks zolbetuximab + EOX) of the FAST study. A 2-week regimen is used for zolbetuximab in Cohort 4 to align with the treatment schedule of nivolumab and mFOLFOX6. The loading dose of 800 mg/m^2 may be de-escalated to 600 mg/m^2 if DLTs are observed. The dosing and schedule of nivolumab and mFOLFOX6 in Cohort 4 are based on the CheckMate 649 clinical trial, in which statistically and clinically significant survival benefit were reported for the combination treatment.

There is no single standard, globally accepted first-line reference chemotherapeutic regimen for advanced gastric cancer. A fluoropyrimidine (5-FU or capecitabine) in combination with platinum agent (cisplatin or oxaliplatin) is an accepted standard of care in both Western and Asian countries (Kim et al. 1993; Ajani 2000; Ohtsu et al. 2003; Ajani et al. 2010; NCCN 2017). Both classes of agents are considered to be interchangeable according to NCCN and ESMO treatment guidelines. Cohort 2 in this study will receive the combination of 5-FU, folinic acid, and oxaliplatin (mFOLFOX6), which is a globally accepted standard-of-care treatment for subjects with metastatic gastroesophageal cancer. Cohort 4 in this study will receive the combination of mFOLFOX6 and nivolumab. The safety of these combination regimens is well documented.

There is no globally accepted checkpoint inhibitor in third line or greater advanced gastric cancer, but pembrolizumab is approved in the US (200 mg every 3 weeks [Fuchs et al, 2018]) and nivolumab in Japan, Taiwan and Korea. Other third line agents include trifluridine and tipiracil or irinotecan.

Similar to Cohort 4, a 2-week regimen is used for zolbetuximab in Cohort 5 to align with the treatment schedule of FLOT. Zolbetuximab will be dosed at 800 mg/m² in combination with FLOT in cycles 1 and 5, followed by subsequent doses of 400 mg/m² every 2 weeks in cycles 2-4 (preoperative) and cycles 6-8 (postoperative) in combination with FLOT. The loading dose of 800 mg/m² may be de-escalated to 600 mg/m² if DLTs are observed. However, for subjects who experience a DLT during preoperative treatment on a loading dose of 600 mg/m^2, the postoperative loading dose may not be omitted, but dose reduction/modification can be considered for FLOT based on investigator judgment. The dosing and schedule of FLOT in Cohort 5 is based on the FLOT4 clinical trial, in which perioperative FLOT demonstrated improved overall survival compared with perioperative ECF/ECX [Al-Batran et al, 2019].

2.3 Endpoints

2.3.1 Primary Endpoints

The primary endpoint is:

- ORR of zolbetuximab as a single agent as assessed by an independent central reader.

2.3.2 Secondary Endpoints

- Pharmacokinetics of zolbetuximab (Cohorts 1A, 2, 3A, 4 and 5): AUC_{inf}, AUC_{inf} (%extrap), AUC_{last}, AUC_{tau}, C_{max}, C_{trough}, t_{max}, $t_{1/2}$, t_{last}, CL, V_z, as appropriate
- Pharmacokinetics of oxaliplatin (Cohort 2) and 5-FU (Cohort 2): AUC_{inf}, AUC_{inf} [%extrap], AUC_{last}, C_{max}, t_{max}, $t_{1/2}$, $t_{last,}$ CL, V_z, as appropriate
- Safety and tolerability of single agent zolbetuximab, in combination with mFOLFOX6 (with or without nivolumab), in combination with pembrolizumab, and in combination with FLOT evaluated by AEs, electrocardiogram (ECG), vital signs, ECOG performance status and laboratory assessments (NCI-CTCAE version 4.03)
- Safety and tolerability of zolbetuximab + FLOT (Cohort 5) include the following additional assessments:

    - Surgical complications
    - Surgical mortality as defined by death within 30 days of surgery
    - Percentage of subjects able to complete preoperative chemotherapy
    - Perioperative mortality and morbidity at 30 days and 90 days post last dose
    - Percentage of subjects able to start postoperative chemotherapy
    - Percentage of subjects able to complete postoperative chemotherapy
- Immunogenicity of zolbetuximab as a single agent, in combination with mFOLFOX6 (with or without nivolumab), in combination with pembrolizumab, and in combination with FLOT as measured by the frequency of anti-drug antibody (ADA) positive subjects
- HRQoL measured by the Quality of Life Questionnaire – Core Questionnaire (QLQ-C30), Oesophago-Gastric Module (OG-25), Global Pain (GP), EuroQOL Five Dimensions Questionnaire (EQ-5D) and the Health Resource Utilization (HRU) questionnaires
- ORR of zolbetuximab in combination with pembrolizumab and in combination with mFOLFOX6 as assessed by an independent central reader
- ORR of zolbetuximab as a single agent, in combination with mFOLFOX6 (with or without nivolumab) by investigator assessment
- DCR, DOR and PFS of zolbetuximab as a single agent and in combination with mFOLFOX6, as assessed by an independent central reader
- DCR, DOR and PFS of zolbetuximab as a single agent and in combination with mFOLFOX6 (with or without nivolumab), as assessed by the investigator
- OS of zolbetuximab as a single agent, in combination with mFOLFOX6 and nivolumab, and in combination with FLOT
- Cohort 5 Only: Antitumor activity of zolbetuximab and FLOT as measured by radiological response (restaging) and pathological response ypTNM (pCR)
- Cohort 5 Only: DFS
- Cohort 5 Only: Minimal residual disease and disease recurrence as measured by circulating tumor DNA (ctDNA)

2.3.3 Exploratory Endpoints

- Potential genomic and/or other biomarkers that may be related to treatment outcome of zolbetuximab as a single agent, in combination with mFOLFOX6 (with or without nivolumab), and in combination with FLOT
- Changes in tumor expression of CLDN18.2
- Changes in immune-related biomarkers in tumor tissue and blood samples
- Pharmacokinetics of pembrolizumab (predose and end-of-infusion concentrations) in combination with zolbetuximab
- Pharmacokinetics of nivolumab (predose and end-of-infusion concentrations) in combination with zolbetuximab and mFOLFOX6

3 STUDY POPULATION

3.1 Selection of Study Population

Subjects with metastatic or locally advanced unresectable gastric or GEJ adenocarcinoma and locoregional gastric or GEJ adenocarcinoma whose tumors are CLDN18.2-positive by central immunohistochemistry (IHC) testing, defined as follows:

- High CLDN18.2 expression has ≥ 75% of tumor cells demonstrating moderate to strong membranous staining as determined by central IHC testing.
- Intermediate CLDN18.2 expression has ≥ 50%, but < 75% of tumor cells demonstrating moderate to strong membranous staining as determined by central IHC testing.

Subjects in Cohorts 2 and 4 must be human epidermal growth factor receptor 2 (HER2) negative based on local or central testing results.

Approximately 143 subjects will be enrolled into the study as outlined in the table below:

	Treatment	No. of Subjects	Line of Treatment	CLDN18.2 Expression	HER2 Status
**Cohort 1A****	Zolbetuximab	30 enrolled*	≥ 3rd Line	High	Not Applicable
**Cohort 2****	Zolbetuximab + mFOLFOX6	21 enrolled	1st Line	High	HER2 Negative
**Cohort 3A****	Zolbetuximab + Pembrolizumab	3 enrolled	≥ 3rd Line	High or Intermediate	Not Applicable
**Cohort 4A****	Zolbetuximab + mFOLFOX6 + Nivolumab	12 enrolled	1st Line	High or Intermediate	HER2 Negative
**Cohort 4B**	Zolbetuximab + mFOLFOX6 + Nivolumab	Approximately 65	1st Line	High or Intermediate	HER2 Negative
**Cohort 5**	Zolbetuximab + FLOT	Approximately 12	Perioperative	High	Not Applicable

CLDN18.2: claudin 18.2; HER2: human epidermal growth factor receptor 2

*Subjects with measurable disease at screening based on RECIST v 1.1 per independent central reader.

**Closed to enrollment.

At least 50% of the subjects enrolled in Cohorts 1A and 2 must be from the US or Europe to ensure the study is regionally balanced.

3.1.1 Cohort 1A

In Cohort 1A, ORR assessed by an independent central reader will be used to evaluate the efficacy of zolbetuximab in approximately 20 subjects. If subjects enrolled do not have measurable disease as assessed by independent central reader, additional subjects may be enrolled to ensure 20 efficacy evaluable subjects.

3.1.2 Cohort 2

Pharmacokinetic evaluable subjects should have sufficient concentration data to derive C_{max} and AUC of zolbetuximab (cycle 2 day 1) and oxaliplatin/5-FU (cycle 1 day 1 and cycle 2

day 1). If any of the 12 subjects enrolled are not pharmacokinetic evaluable, additional subjects may be enrolled to ensure 12 pharmacokinetic evaluable subjects.

3.1.3 Cohort 3A

In Cohort 3A, the tolerability and safety of zolbetuximab in combination with pembrolizumab will be evaluated during the 3-week DLT assessment period. The TEM will be held after the end of the 3-week DLT assessment period for the first 3 or 6 total DLT-evaluable subjects enrolled in this cohort.

3.1.4 Cohort 4

In Cohort 4A, the tolerability and safety of zolbetuximab in combination with mFOLFOX6 and nivolumab will be evaluated during the 2-week dose-limiting toxicity (DLT) assessment period. Initially, 3 subjects will be enrolled into Cohort 4A and receive the zolbetuximab loading dose of 800 mg/m^2 in combination with nivolumab 240 mg and mFOLFOX6 on cycle 1 day 1, followed by zolbetuximab 400 mg/m^2 in combination with nivolumab 240 mg and mFOLFOX6 every 2 weeks [days 15 and 29] (1 cycle = 6 weeks). Zolbetuximab will be administered first, followed by the nivolumab and then mFOLFOX6. Any of the zolbetuximab, mFOLFOX6 and/or nivolumab related adverse events (AEs) specified as the DLTs will be assessed. Subjects will receive up to 12 mFOLFOX6 treatments (4 cycles).

In Cohort 4B, subjects will be treated with the combination of zolbetuximab, mFOLFOX6 and nivolumab at the dose deemed tolerable in Cohort 4A. Subjects will receive up to 12 mFOLFOX6 treatments (4 cycles).

3.1.5 Cohort 5

In Cohort 5, the tolerability and safety of zolbetuximab in combination with FLOT will be evaluated in subjects with locoregional resectable gastric or GEJ adenocarcinoma.

3.2 Inclusion Criteria

Waivers to the inclusion criteria will NOT be allowed. Laboratory results obtained during screening should be used to determine eligibility criteria. In situations where laboratory results are outside of the permitted range, the investigator may opt to retest the subject and subsequent within-range screening results may be used to confirm eligibility. Computerized tomography (CT) scans and magnetic resonance imaging (MRI) conducted as part of a subject's routine clinical management (i.e., standard of care) obtained before signing the ICF may be utilized for screening or baseline purposes, provided the procedures met the protocol-specified criteria and were performed within the Screening period.

Subject is eligible for the study if all of the following apply:

1. Institutional Review Board (IRB)/Independent Ethics Committee (IEC) approved written informed consent and privacy language as per national regulations (e.g., Health Insurance Portability and Accountability Act [HIPAA] Authorization for US sites) must be obtained from the subject or legally authorized representative prior to any study-related procedures (including withdrawal of prohibited medication, if applicable).

2. Subject is considered an adult according to local regulation at the time of signing informed consent.
3. Female subject is eligible to participate if she is not pregnant [See Appendix 12.6 Contraception Requirements] and at least one of the following conditions applies:
   a. Not a woman of child-bearing potential (WOCBP) as defined in Appendix 12.6 Contraception Requirements
   OR
   b. WOCBP who agrees to follow the contraceptive guidance as defined in Appendix 12.6 Contraception Requirements throughout the treatment period and for at least 9 months after the final oxaliplatin administration and 6 months after the final administration of all other study drugs
4. Female subject must agree not to breastfeed starting at screening and throughout the study period, and for 6 months after the final study drug administration.
5. Female subject must agree not to donate ova starting at screening and throughout the study period, and for 9 months after the final oxaliplatin administration and 6 months after the final administration of all other study drugs.
6. A sexually active male subject with a female partner(s) who is of child-bearing potential must agree to use contraception as detailed in Appendix 12.6 Contraception Requirements during the treatment period and for at least 6 months after the final study drug administration.
7. Male subject must agree not to donate sperm starting at screening and throughout the study period, and for 6 months after the final study drug administration.
8. Male subject with a pregnant or breastfeeding partner(s) must agree to remain abstinent or use a condom for the duration of the pregnancy or time partner is breastfeeding throughout the study period and for 6 months after the final study drug administration.
9. Subject has histologically confirmed gastric or GEJ adenocarcinoma.
10. Cohorts 1-4: Subject has radiographically confirmed, locally advanced, unresectable or metastatic disease within 28 days prior to the first dose of study treatment.
11. Subject's tumor is positive for CLDN18.2 expression demonstrating moderate to strong membranous staining as determined by central IHC testing.
12. Subject agrees not to participate in another interventional study while on treatment.
13. Subject has ECOG performance status 0 to 1.
14. Subject has predicted life expectancy ≥ 12 weeks in the opinion of the investigator.
15. Subject must meet all of the following criteria based on the centrally or locally analyzed laboratory tests collected within 14 days prior to the first dose of study treatment. In case of multiple central laboratory data within this period, the most recent data should be used.
   - Hemoglobin (Hgb) ≥ 9 g/dL (transfusion is allowed, but post-transfusion Hgb [24 hours or later following transfusion] must be ≥ 9 g/dL)
   - Absolute neutrophil count (ANC) ≥ 1.5×10^9/L
   - Platelets ≥ 100×10^9/L

- Albumin ≥ 2.5 g/dL
- Total bilirubin ≤ 1.5 × upper limit of normal (ULN)
- Aspartate aminotransferase (AST) and alanine aminotransferase (ALT) ≤ 2.5 × ULN in subjects without liver metastases (≤ 5 × ULN if liver metastases are present)
- Cohorts 1-4: Estimated creatinine clearance ≥ 30 mL/min
- Cohort 5: Serum creatinine ≤ 1.5 × ULN, or estimated creatinine clearance ≥ 50 mL/min for subjects with serum creatinine levels > 1.5 × ULN
- Prothrombin time/international normalized ratio and partial thromboplastin time ≤ 1.5 × ULN (except for subjects receiving anticoagulation therapy)

Specific to Cohort 1A:

1. Subject has measurable disease according to RECIST 1.1 within 28 days prior to the first dose of study treatment per investigator assessment. For subjects with only 1 evaluable lesion and prior radiotherapy ≤ 3 months before enrollment, the lesion must either be outside the field of prior radiotherapy or must have documented progression following radiation therapy.
2. Subject has disease progression on or after at least 2 prior regimens for their advanced disease, including fluoropyrimidine and platinum-containing chemotherapy, and if appropriate, HER2/neu-targeted therapy and all associated side effects have resolved to grade 1 or less.
3. Subject must have an additional available tumor specimen collected within 3 months prior to the first dose of study treatment.
4. Subject must be an appropriate candidate for a tumor biopsy and is amenable to undergo a tumor biopsy during the screening period (if applicable) and treatment period as indicated in the Schedule of Assessments.

Specific to Cohort 2:

1. Subject has measurable disease according to RECIST 1.1 within 28 days prior to the first dose of study treatment per investigator assessment. For subjects with only 1 evaluable lesion and prior radiotherapy ≤ 3 months before enrollment, the lesion must either be outside the field of prior radiotherapy or must have documented progression following radiation therapy.
2. Subject has not received prior systemic anti-cancer therapy for their advanced disease (subject may have received neoadjuvant and/or fluorouracil-containing adjuvant chemotherapy as long as it has been completed ≥ 6 months before the first dose of study treatment).
3. Subject has a gastric or GEJ tumor that is HER2-negative as determined by local or central testing.
4. Subject must have an additional available tumor specimen collected within 3 months prior to the first dose of study treatment.

5. Subject must be an appropriate candidate for a tumor biopsy and is amenable to undergo a tumor biopsy during the screening period (if applicable) and treatment period as indicated in the Schedule of Assessments.

Specific to Cohort 3A:

1. Subject has radiologically evaluable disease (measurable and/or non-measurable) according to RECIST 1.1, per local assessment, ≤ 28 days prior to the first dose of study treatment. For subjects with only 1 evaluable lesion and prior radiotherapy ≤ 3 months before enrollment, the lesion must either be outside the field of prior radiotherapy or must have documented progression following radiation therapy.
2. Subject has disease progression on or after at least 2 prior regimens for their advanced disease, including fluoropyrimidine and platinum-containing chemotherapy, and if appropriate, HER2/neu-targeted therapy.
3. Subject has not received prior checkpoint inhibitor therapy.

Specific to Cohorts 4A and 4B:

1. Subject has radiologically evaluable disease (measurable and/or non-measurable) according to RECIST 1.1, per local assessment, ≤ 28 days prior to the first dose of study treatment. For subjects with only 1 evaluable lesion and prior radiotherapy ≤ 3 months before enrollment, the lesion must either be outside the field of prior radiotherapy or must have documented radiographic progression following radiation therapy.
2. Subject has not received prior systemic anti-cancer therapy for their advanced disease (subject may have received neoadjuvant and/or fluorouracil-containing adjuvant chemotherapy as long as it has been completed ≥ 6 months before the first dose of study treatment).
3. Subject has a gastric or GEJ tumor that is HER2-negative as determined by local or central testing.
4. Subject has not received prior checkpoint inhibitor therapy.

Specific to Cohort 4B Only:

1. Subject must have an additional available tumor specimen collected within 3 months prior to the first dose of study treatment.
2. Subject must be an appropriate candidate for a tumor biopsy and is amenable to undergo a tumor biopsy during the screening period (if applicable) and treatment period as indicated in the Schedule of Assessments.

Specific to Cohort 5 Only:

1. Subject has new histologically confirmed primary gastric or GEJ adenocarcinoma that is amenable to curative resection.
2. Subject has locoregional, resectable gastric or GEJ adenocarcinoma. GEJ may include type I-III Siewert classification. Clinical stage will be determined by endoscopic ultrasound (EUS) and/or CT or MRI. Diagnostic laparoscopy may be used as per institutional guidelines and clinical practices.

3. Subject meets one of the following criteria of locoregional disease by clinical TNM staging:
    a. GEJ: cT2,N0 (high risk-lesions: ≥ 3 cm, poorly differentiated), cT1b-cT2,N+ or cT3–cT4a,Any N.
    b. Gastric: T2 to T4a, and/or N1-3,M0.
4. Subject's tumor expresses CLDN18.2 in ≥ 75% of tumor cells demonstrating moderate to strong membranous staining as determined by central IHC testing

3.3 Exclusion Criteria

Waivers to the exclusion criteria will **NOT** be allowed.

Subject who meets any of the following exclusion criteria prior to enrollment is not eligible for enrollment:

1. Subject has had prior severe allergic reaction or intolerance to known ingredients of zolbetuximab or other monoclonal antibodies, including humanized or chimeric antibodies.
2. Subject has known immediate or delayed hypersensitivity or contraindication to any component of study treatment.
3. Subject has received other investigational agents or devices concurrently or within 28 days prior to first dose of study treatment.
4. Subject has received systemic immunosuppressive therapy, including systemic corticosteroids 14 days prior to first dose of study treatment. Subjects using a physiologic replacement dose of hydrocortisone or its equivalent (defined as up to 30 mg per day of hydrocortisone or up to 10 mg per day of prednisone), receiving a single dose of systemic corticosteroids or receiving systemic corticosteroids as pre-medication for radiologic imaging contrast use are allowed.
5. Subject has a complete gastric outlet syndrome or a partial gastric outlet syndrome with persistent recurrent vomiting.
6. Per investigator judgment, subject has significant gastric bleeding and/or untreated gastric ulcers that would preclude the subject from participation per investigator judgment.
7. Subject has history of central nervous system metastases and/or carcinomatous meningitis from gastric/GEJ cancer.
8. Subject has a known history of a positive test for human immunodeficiency virus (HIV) infection or known active hepatitis B (positive hepatitis B surface antigen [HBsAg]) or hepatitis C infection. NOTE: Screening for these infections should be conducted per local requirements.
    - For subjects who are negative for HBs Ag, but HBc Ab positive, an HB DNA test will be performed and if positive the subject will be excluded.
    - Subjects with positive hepatitis C virus (HCV) serology, but negative HCV RNA test results are eligible.
    - Subjects treated for HCV with undetectable viral load results are eligible.

9. Subject has had within 6 months prior to first dose of study treatment any of the following: unstable angina, myocardial infarction, ventricular arrhythmia requiring intervention or hospitalization for heart failure.
10. Subject has active infection requiring systemic therapy that has not completely resolved within 7 days prior to the start of study treatment.
11. Subject has active autoimmune disease that has required systemic treatment within the past 3 months prior to the start of study treatment.
12. Subject has a clinically significant disease or co-morbidity that in the opinion of the investigator may adversely affect the safe delivery of treatment within this study or make the subject unsuitable for study participation.
13. Subject has psychiatric illness or social situations that would preclude study compliance per investigator's judgment.
14. Subject has had a major surgical procedure ≤ 28 days before start of study treatment.
15. Subject is without complete recovery from a major surgical procedure ≤ 14 days before start of study treatment.
16. Subject has received radiotherapy for locally advanced unresectable or metastatic gastric or GEJ adenocarcinoma ≤ 14 days (Cohorts 1 and 3A) and ≤ 28 days (Cohorts 2 and 4A or 4B) prior to start of study treatment and has NOT recovered from any related toxicity.
17. Subject has another malignancy for which treatment is required, per investigator's clinical judgment.
18. Cohorts 2, 4 and 5 Only, subject has any of the following:
    - Prior severe allergic reaction or intolerance to any component of mFOLFOX6 or FLOT chemotherapeutics in this study.
    - Known dihydropyrimidine dehydrogenase deficiency (DPD) (screening for DPD deficiency should be conducted per local requirements).
    - Known peripheral neuropathy > grade 1 (absence of deep tendon reflexes as the sole neurological abnormality does not render the subject ineligible).
    - Sinusoidal obstruction syndrome, formerly known as veno-occlusive disease, if present, should be stable or improving per investigator's judgment.
    - History of clinically significant ventricular arrhythmias (i.e., sustained ventricular tachycardia, ventricular fibrillation, or Torsades de Pointes).
    - QTc interval > 450 msec for male subjects; QTc interval > 470 msec for female subjects.
    - History or family history of congenital long QT syndrome.
    - Cardiac arrhythmias requiring anti-arrhythmic medications (Subjects with rate controlled atrial fibrillation for > 1 month prior to first dose of study treatment are eligible).
19. Cohorts 3A, 4A and 4B Only, subject has any of the following:
    - Ongoing or previous autoimmune disease or interstitial lung disease, active diverticulitis or gastrointestinal ulcerative disease, or solid organ or stem cell

transplant (for Cohort 4), or other uncontrolled or clinically significant medical disorders.

- Type 1 diabetes mellitus, endocrinopathies stably maintained on appropriate replacement therapy or skin disorders (e.g., vitiligo, psoriasis, or alopecia) not requiring systemic treatment are allowed.
- Known history of serious hypersensitivity reaction to a known ingredient of pembrolizumab or nivolumab.
- Cohort 4B Only: Subject has microsatellite instability-high or mismatch repair deficient tumors.

20. Cohort 5 Only, subject has either of the following:
    - Subject cannot undergo curative resection per the investigator's judgment
    - Subject meets the following criterion of locoregional disease by clinical TNM staging: cT1N0.

4 TREATMENT

4.1 Identification of Investigational Medicinal Product

4.1.1 Study Drug

The investigational medicinal product (IMP), zolbetuximab, is a sterile lyophilized powder for solution for infusion. Chimeric (mouse/human IgG1) monoclonal antibody zolbetuximab is the active pharmaceutical ingredient. Each vial contains 105 mg of zolbetuximab and has to be reconstituted with 5.0 mL water for injection (WFI) per vial to a concentration of 20 mg/mL. Further dilution with sterile 0.9% sodium chloride to a final concentration of 2 mg/mL is required.

Vials are single-use only and are closed with a rubber stopper and an aluminum cap. All excipients are animal component free and of compendial grade (Pharm. Eur. current version). No preservatives are contained, since the vial is designed for single use.

4.1.2 Other Drugs

mFOLFOX6, pembrolizumab, nivolumab, and FLOT are authorized products and will be used in this study according to the marketing authorizations.

4.1.2.1 mFOLFOX6 (Cohorts 2 and 4 Only)

mFOLFOX6 components are auxiliary medicinal products (AxMP)/noninvestigational medicinal products (nIMP) and will be administered in combination with zolbetuximab. If folinic acid is not available, it may be replaced by levofolinic acid. mFOLFOX6 products should be given according to institutional standards, published guidelines and the respective product package insert(s), and dosed according to this protocol.

4.1.2.2 Pembrolizumab (Cohort 3 Only)

Pembrolizumab is an AxMP/nIMP and will be administered in combination with zolbetuximab. Pembrolizumab products should be given according to institutional standards,

published guidelines and the respective product prescribing information, and dosed according to this protocol.

4.1.2.3 Nivolumab (Cohort 4 Only)

Nivolumab is an AxMP/nIMP and will be administered in combination with zolbetuximab and mFOLFOX6. Nivolumab products should be given according to institutional standards, published guidelines and the respective product prescribing information, and dosed according to this protocol.

4.1.2.4 FLOT (Cohort 5 Only)

FLOT components are AxMP/nIMP and will be administered in combination with zolbetuximab**.** If folinic acid is not available, it may be replaced by levofolinic acid. FLOT component products should be given according to institutional standards, published guidelines and the respective product package insert(s), and dosed according to this protocol.

4.1.3 Comparative Drug(s)

Not applicable

4.2 Packaging and Labeling

The IMP used in this study will be prepared, packaged and labeled under the responsibility of qualified staff at Astellas Pharma Global Development Inc. (APGD), Astellas Pharma Inc. (API) or sponsor’s designee in accordance with APGD or sponsor’s designee Standard Operating Procedures (SOPs), Good Manufacturing Practice (GMP) guidelines, ICH Good Clinical Practice (GCP) guidelines and applicable local laws/regulations.

Each vial will bear a label conforming to regulatory guidelines, GMP and local laws and regulations that identifies the contents as investigational drug.

A qualified person from Astellas Pharma Europe B.V. or sponsor’s designee will perform the final release of the study drug according to the requirements of the EU Clinical Trial Regulation 536/2014, current GMP guidelines, ICH GCP guidelines and applicable local laws/regulations.

4.3 Study Drug Handling

Current ICH GCP Guidelines require the investigator to ensure that study drug and other drug deliveries from the sponsor are received by the investigator/or designee and that:

- Such deliveries are recorded,
- Study drug is handled and stored according to labeled storage conditions,
- Study drug with appropriate expiry/retest and is only dispensed to study subjects in accordance with the protocol and
- Any unused study drug is returned to the sponsor.

Study drug inventory and accountability records will be kept by the investigator or designee. Study drug accountability throughout the study must be documented and reconciled. The following guidelines are therefore pertinent:

- The investigator agrees not to supply study drugs to any persons except the eligible subjects in this study in accordance with the protocol.
- The investigator or designee will keep the study drugs in a pharmacy or other locked and secure storage facility under controlled storage conditions, accessible only to those authorized by the investigator to dispense these study drugs.
- A study drug inventory will be maintained by the investigator or designee. The inventory will include details of material received and a clear record of when they were dispensed and to which subject.
- At the conclusion or termination of this study, the investigator or designee agrees to conduct a final drug supply inventory and to record the results of this inventory on the Drug Accountability Record. It must be possible to reconcile delivery records with those of used and/or returned study drug. Any discrepancies must be accounted for and documented. Appropriate forms of deliveries and returns must be signed by the site staff delegated this responsibility.
- The site staff must return study drug to the sponsor or designee at the end of the study or upon expiration unless otherwise approved by the sponsor.

Unique to Japan region:

The head of the study site or the test product storage manager should take accountability of the test product as following issues:

- The drug storage manager should store and take accountability of the study drug in conforming to the procedures for handling the study drug written by the sponsor.
- The drug storage manager should prepare and retain records of the study drug's receipt, inventory at the study site, use by each participant, and return of unused study drug to the sponsor or alternative disposal. These records should include dates, quantities, batch/serial numbers, expiration dates (if applicable) and the unique code numbers assigned to the study drug and participants.
- The drug storage manager should prepare and retain records that document adequately that the participants were provided the doses specified in the protocol and reconcile all the study drug supplied from the sponsor.

4.3.1 Zolbetuximab

The IMP, zolbetuximab, is a sterile lyophilized powder with the chimeric (mouse/human IgG1) monoclonal antibody zolbetuximab as the active pharmaceutical ingredient.

The IMP is supplied by Astellas in single-use glass vials containing 105 mg of zolbetuximab. All excipients are animal component free and of compendial grade (Pharm. Eur. current version). No preservatives are contained, since the vial is designed for single use.

The IMP should be stored at refrigerated conditions (2°C to 8°C/36°F to 46°F). Temperature should be controlled and monitored. Details of IMP receipt, labeling, storage and preparation are provided in the Pharmacy Manual and Infusion Guidelines.

The zolbetuximab used in this study will be prepared, packaged, and labeled under the responsibility of qualified staff at Astellas Pharma Global Development, Inc. (APGD),

Astellas US Technologies, Inc. or sponsor's designee in accordance with APGD or sponsor's designee Standard Operating Procedures (SOPs), Good Manufacturing Practices (GMP) guidelines, International Council for Harmonisation (ICH) Good Clinical Practice (GCP) guidelines, and applicable local laws/regulations.

Each vial and carton will bear a label conforming to regulatory guidelines, GMP and local laws and regulations that identifies the contents as investigational drug.

As required, a qualified person of Astellas Pharma Europe B.V. (APEBV) or sponsor's designee will perform the final release of the medication according to the requirements of the EU Clinical Trial Regulation 536/2014.

4.3.1.1 Observation Period Following Zolbetuximab Infusion

Following the subject's first dose of zolbetuximab, the subject must remain at the site facility for 2 hours post infusion. If AEs are observed during this time, infusion time should be extended and subjects should continue to be observed for 2 hours post infusion. If the subject does not develop any AEs, the subject should be observed for 1 hour post infusion for their subsequent zolbetuximab infusions. The subject should be instructed to notify site personnel if they develop any AEs during this time period.

4.3.2 0.9% Sodium Chloride Injection

0.9% Sodium Chloride Injection will be used for infusion solution preparation in this study for zolbetuximab. 0.9% Sodium Chloride Injection will not be manufactured or provided to sites by the sponsor. Sites should use their own commercially obtained supply of 0.9% Sodium Chloride Injection. Details of preparation of infusion solution are provided in the Pharmacy Manual and Infusion Guidelines.

4.3.3 mFOLFOX6 (Cohort 2)

mFOLFOX6 treatment follows the zolbetuximab infusion, if both are scheduled on the same day.

Oxaliplatin 85 mg/m² IV infusion is given as a 2-hour IV infusion in 500 mL concurrent with leucovorin 400 mg/m² (or levo-leucovorin 200 mg/m²) IV infusion. Both solutions must be prepared separately and administered using a Y-type administration set.

This is followed by 5-FU 400 mg/m² IV bolus (given in 5 to 15 minutes), followed by a continuous 46 to 48-hour 5-FU infusion 2400 mg/m².

4.3.3.1 Oxaliplatin

Oxaliplatin vials containing 50 or 100 mg of oxaliplatin powder for injection must be reconstituted with 10 or 20 mL, respectively, WFI or 5% dextrose injection to provide a solution containing 5 mg/mL. Reconstituted solution or commercially available concentrate (5 mg/mL) must be further diluted in 500 mL of 5% dextrose injection. Follow the specific instructions for storage and preparation from the package insert.

4.3.3.2 Leucovorin

Leucovorin is available as powder for injection and solution for injection. The powder for injection is reconstituted with sterile WFI to provide a solution containing 10 or 20 mg/mL. Further dilution to the required dose should be made with 5% dextrose or 0.9% saline for infusion, and followed by immediate administration. If leucovorin is not available, it may be replaced by levofolinic acid. Follow the specific instructions for storage and preparation from the package insert.

4.3.3.3 Fluorouracil

5-FU is available as solution for IV use (e.g., 50 mg/mL). Further dilution to the required dose should be made with 5% dextrose or 0.9% saline for infusion. Follow the specific instructions for storage and preparation from the package insert.

4.3.4 Pembrolizumab (Cohort 3)

Pembrolizumab will be supplied by the responsible site pharmacy of each investigational site or by the APGD, if applicable.

Pembrolizumab is available in 2 dosage forms:

- For Injection: 50 mg white to off-white lyophilized powder in a single-dose vial for reconstitution.
- Injection: 100 mg/4 mL (25 mg/mL) clear to slightly opalescent, colorless to slightly yellow solution in a single-dose vial (supplied by APGD).

Pembrolizumab treatment should begin 1 hour after the zolbetuximab infusion ends. Pembrolizumab (200 mg) infusion solution will be administered intravenously over 30 minutes through an intravenous line containing a sterile, non-pyrogenic, low-protein binding 0.2 micron to 5 micron in-line or add-on filter. Do not co-administer other drugs through the same infusion line. Refer to the approved pembrolizumab package insert, SPC, monograph or local product information supplied by the manufacturer for additional instructions for drug preparation and dilution.

4.3.5 mFOLFOX6 and Nivolumab (Cohort 4)

Nivolumab is available in 3 forms for injection: 40 mg/4 mL (10 mg/mL), 100 mg/10 mL (10 mg/mL), and 240 mg/24 mL (10 mg/mL) as clear to opalescent, colorless to pale-yellow solution in a single-dose vial.

Nivolumab (240 mg) treatment should begin approximately 30 minutes after the zolbetuximab infusion ends. Nivolumab infusion solution will be administered intravenously over 30 minutes through an intravenous line containing a sterile, non-pyrogenic, low protein binding in-line filter (pore size of 0.2 µm to 1.2 µm). Do not co-administer other drugs through the same infusion line. Refer to the approved nivolumab package insert, SPC, monograph or local product information supplied by the manufacturer for additional instructions for drug preparation and dilution.

mFOLFOX6 treatment should follow the nivolumab infusion. Oxaliplatin 85 mg/m² IV infusion is given as a 2-hour IV infusion in 500 mL concurrent with leucovorin 400 mg/m² (or levo-leucovorin 200 mg/m²) IV infusion. Both solutions must be prepared separately and administered using a Y-type administration set. This is followed by 5-FU 400 mg/m² IV bolus (given in 5 to 15 minutes), followed by a continuous 46 to 48-hour 5-FU infusion 2400 mg/m².

4.3.6 FLOT (Cohort 5)

FLOT will be locally sourced by the responsible site pharmacy or by the sponsor, as applicable, and prepared according to the local product labels. For both preoperative and postoperative treatment, FLOT will be administered on day 2 of cycles 1 and 5. For cycles 2-4 and 6-8, FLOT may be administered after zolbetuximab on day 1, or may be split over 2 days with FLOT administered on day 2 of each cycle. Refer to [Section 5.1.1.6] for administration details.

4.4 Blinding

This section is not applicable as this is an open-label study.

4.5 Assignment and Allocation

Subject enrollment will be performed via IRT. All subjects who meet the eligibility criteria will be enrolled. The site personnel will dispense the treatment according to the IRT system's assignment. Specific procedures for enrollment through the IRT are contained in the study procedures manual.

5 TREATMENTS AND EVALUATION

5.1 Dosing and Administration of Study Drug(s) and Other Medication(s)

5.1.1 Dose/Dose Regimen and Administration Period

5.1.1.1 Zolbetuximab

Zolbetuximab will be administered every 3 weeks (every 2 weeks for Cohorts 4 and 5) as a minimum 2-hour IV infusion. IV infusion may be interrupted or slowed down to manage toxicity per Table 15 below. Please refer to the Pharmacy Manual and Infusion Guidelines for more detailed information.

For Cohorts 3A, 4A and 5, DLTs will be assessed and the sponsor will decide on the tolerability of the current dose level.

For Cohorts 1A, 2 and 3A, an 800 mg/m^2 loading dose will be administered at cycle 1, followed by subsequent doses of 600 mg/m^2 every 3 weeks.

Cohort 2 Only: Zolbetuximab will be administered on cycle 1 day 3 (Cohort 2) to allow for the mFOLFOX6 pharmacokinetic collection. For all subsequent cycles, zolbetuximab will be administered on day 1 every 3 weeks. For visits at which zolbetuximab and mFOLFOX6 are

to be administered on the same day, zolbetuximab should be administered PRIOR to mFOLFOX6.

Cohort 4A Only: An 800 mg/m^2 loading dose will be administered followed by subsequent doses of 400 mg/m^2 every 2 weeks (day 1, day 15 and day 29 of every 6-week cycle). May be de-escalated to 600 mg/m^2 followed by subsequent doses of 400 mg/m^2 every 2 weeks if DLTs are observed.

Cohort 4B Only: Subjects will be treated with the combination of zolbetuximab, mFOLFOX6 and nivolumab at the dose deemed tolerable in Cohort 4A.

Cohort 5 Only: An 800 mg/m^2 loading dose will be administered, followed by subsequent doses of 400 mg/m^2 every 2 weeks for 4 cycles preoperatively and 4 cycles postoperatively. If the zolbetuximab 800 mg/m^2 loading dose administered on cycle 1 day 1 is assessed as not tolerable based on the DLT assessment, then 3 to 6 subjects will be treated with a zolbetuximab 600 mg/m^2 loading dose on cycle 1 day 1, followed by zolbetuximab 400 mg/m^2 on day 1 of cycles 2-4. For subjects who experience a DLT during preoperative treatment on the loading dose of 800 mg/m^2, the postoperative loading dose may be reduced to 600 mg/m^2. However, for subjects who experience a DLT during preoperative treatment on a loading dose of 600 mg/m^2, the postoperative loading dose may not be omitted, but dose reduction/modification can be considered for FLOT based on investigator judgment.

Table 15 Infusion Rates Recommended for Each Zolbetuximab Infusion

Zolbetuximab Dose	Initial Infusion Rate (First 30-60 Minutes)*	Subsequent Infusion Rate†
800 mg/m^2	100 mg/m^2/hr**	200-400 mg/m^2/hr‡
600 mg/m^2	75 mg/m^2/hr**	150-300 mg/m^2/hr‡
400 mg/m^2	50 mg/m^2/hr**	100-200 mg/m^2/hr‡

* In the absence of adverse reactions after 30-60 minutes, the infusion rate can be increased to the subsequent infusion rate as tolerated.
** Infusion rate represents 12.5% of total infusion dose (volume)/hour (as detailed in the pharmacy manual).
† For subsequent infusion rate, recommend starting at the lower indicated infusion rate and increasing to the maximum infusion rate as tolerated.
‡ Subsequent infusion rate represents 25% to 50% of total infusion dose (volume)/hour (as detailed in the pharmacy manual).

5.1.1.2 Antiemetic Premedication

Antiemetic premedication (prophylactic antiemetics) should be administered prior to each study treatment. (NOTE: Subjects receiving zolbetuximab do not need to be premedicated for prevention of IRRs; however, subjects should be closely monitored for IRRs to facilitate early identification and management.)

- Antiemetic premedication
  - IV antiemetic premedication should be initiated prior to treatment, or
  - Oral antiemetic premedication should be initiated at a minimum of 30 minutes prior to treatment.

On days when subjects receive both zolbetuximab and mFOLFOX6 (Cohorts 2 and 4 only), pembrolizumab (Cohort 3 only), or FLOT (Cohort 5), antiemetic premedication will be given prior to zolbetuximab administration. It is recommended that the prophylactic antiemetic regimen include (but is not limited to) the following agents:

- NK-1 receptor blockers
- 5-HT3 receptor blockers*

* To minimize the risk of Torsades de Pointes, administer 5-HT3 receptor blockers with caution to subjects who have or may develop QTc prolongation.

The impact of corticosteroids on the potential efficacy of zolbetuximab, pembrolizumab or nivolumab is not known. Therefore, use corticosteroids with caution if necessary as clinically indicated.

Antiemetics are authorized products and will be used in this study according to the marketing authorizations.

5.1.1.3 mFOLFOX6 (Cohorts 2, 4A and 4B Only)

mFOLFOX6 will be administered every 2 weeks (day 1, day 15 and day 29 of every 6-week cycle) for up to 12 mFOLFOX6 treatments (4 cycles). Beginning at cycle 5, subjects may continue on 5-FU and leucovorin or folinic acid per investigator’s discretion.

- Oxaliplatin: 85 mg/m^2 IV infusion over 2 hours or per institutional guidelines every 2 weeks for a maximum of 4 cycles (3 treatments in each cycle). NOTE: ECG is required to be performed and assessed locally prior to every oxaliplatin infusion (before any antiemetic treatment) and following completion of every oxaliplatin infusion. ECG should be performed up to 48 hours *prior* to and up to 6 hours *following* every oxaliplatin infusion. Oxaliplatin administration and electrolyte levels should be managed according to the investigator’s judgment for subjects with grade 1 or 2 hypokalemia, hypomagnesemia and/or hypocalcemia.
- Leucovorin: 400 mg/m^2 IV infusion over 2 hours every 2 weeks (or levofolinic acid given at either the protocol-recommended doses or as deemed appropriate by the investigator in accordance with institutional standard of care).
  SPECIFIC TO JAPAN: Or, levofolinate 200 mg/m^2 IV infusion over 2 hours or per institutional standard of care every 2 weeks for 4 cycles. Levofolinate can be continued beyond 4 cycles based on investigator’s judgment.
- 5-FU Bolus: 400 mg/m^2 IV bolus over 5 to 15 minutes or infused per institutional guidelines every 2 weeks.
- 5-FU Infusion: 2400 mg/m^2 continuous IV infusion over 46 to 48 hours or per institutional guidelines every 2 weeks.
- Cohort 4 Only: Infusion should begin after the completion of nivolumab.

5.1.1.4 Pembrolizumab (Cohort 3A Only)

Pembrolizumab will be administered at a dose of 200 mg intravenously over 30 minutes on day 1 of every 21-day cycle and will be infused 1 hour after the zolbetuximab infusion is completed. Dose reductions are not permitted.

Pembrolizumab should be given intravenously according to institutional guidelines, published guidelines and the respective product prescribing information, and dosed according to this protocol.

5.1.1.5 Nivolumab (Cohorts 4A and 4B Only)

Nivolumab will be administered every 2 weeks (day 1, day 15 and day 29 of every 6-week cycle) at a dose of 240 mg intravenously over 30 minutes and will be infused 30 minutes after the zolbetuximab infusion is completed. mFOLFOX6 infusion will follow nivolumab. Dose reductions are not permitted.

Nivolumab should be given intravenously according to institutional guidelines, published guidelines and the respective product prescribing information, and dosed according to this protocol.

5.1.1.6 FLOT (Cohort 5 Only)

- Docetaxel: 50 mg/m^2 IV infusion over 1 hour on cycles 1 and 5 day 2 and cycles 2-4 and 6-8 days 1 or 2.
- Oxaliplatin: 85 mg/m^2 IV infusion over 2 hours or per institutional guidelines on cycles 1 and 5 day 2 and cycles 2-4 and 6-8 days 1 or 2.
    - NOTE: ECG is required to be performed and assessed locally prior to every oxaliplatin infusion (before any antiemetic treatment) and following completion of every oxaliplatin infusion. ECG should be performed 48 hours prior to and up to 6 hours following every oxaliplatin infusion. Oxaliplatin administration and electrolyte levels should be managed according to the investigator’s judgment for subjects with grade 1 or 2 hypokalemia, hypomagnesemia and/or hypocalcemia.
- Leucovorin: 200 mg/m^2 IV infusion over 2 hours on cycles 1 and 5 day 2 and cycles 2-4 and 6-8 days 1 or 2 (or levofolinic acid given as deemed appropriate by the investigator in accordance with institutional standard of care).
    - ***SPECIFIC TO JAPAN:*** Or, levofolinate 100 mg/m^2 IV infusion over 2 hours or per institutional standard of care on cycles 1 and 5 day 2 and cycles 2-4 and 6-8 days 1 or 2.
- 5-FU: 2600 mg/m^2 IV infusion over a 24-hour period on cycles 1 and 5 day 2 and cycles 2-4 and 6-8 days 1 or 2.

At the investigator’s discretion growth factors, supportive therapeutic and prophylactic actions may be used according to standard practice guidelines.

5.1.2 Treatment Delays or Discontinuation

5.1.2.1 Zolbetuximab Delay

Cohort 1: In case of zolbetuximab treatment delay beyond 2 days, it may be administered as soon as possible. Subsequent zolbetuximab treatment should be administered every 3 weeks from last date of administration, which would now be considered day 1 of the next cycle.

Cohort 2: Dose delays for zolbetuximab should not occur in the first 2 cycles without discussion with the medical monitor. If zolbetuximab treatment is delayed more than 2 days

then it should be administered with the next scheduled mFOLFOX6 treatment, which would now be considered day 1 of the next cycle. If mFOLFOX6 has been discontinued, zolbetuximab should continue to be administered every 3 weeks from the last date of administration.

Cohort 3: In cases where zolbetuximab and pembrolizumab are both delayed beyond 2 days, they both may be administered as soon as possible. Subsequent zolbetuximab and pembrolizumab treatment should be administered every 3 weeks from last date of administration, which would now be considered day 1 of the next cycle. If only zolbetuximab or pembrolizumab is delayed more than 5 days, the delayed drug should be given at the next scheduled combined dosing.

Cohort 4: Dose delays for zolbetuximab should not occur in the first 2 cycles without discussion with the medical monitor. If zolbetuximab treatment is delayed more than 2 days then it should be administered with the next scheduled mFOLFOX6 and nivolumab treatment, which would now be considered day 1 of the next cycle. If mFOLFOX6 has been discontinued, zolbetuximab (with or without nivolumab) should continue to be administered every 2 weeks from the last date of administration.

Cohort 5: Dose delays for zolbetuximab should not occur in cycles 1, 2, 5 or 6 without discussion with the medical monitor. If zolbetuximab treatment is delayed more than 2 days, then the next dose will be administered on day 1 of the following cycle.

A delay of zolbetuximab treatment for > 28 days from when the next zolbetuximab treatment was scheduled to be administered (> 42 days from when the last dose of zolbetuximab began) due to unresolved toxicity associated with zolbetuximab, will result in the subject discontinuing zolbetuximab. For Cohort 5, this does not apply to the duration between preoperative and postoperative zolbetuximab administration.

One case of PRES has been reported in subjects receiving zolbetuximab. **Discontinue zolbetuximab if PRES is suspected.** Confirm PRES diagnosis by brain imaging, preferably by MRI.

5.1.2.2 mFOLFOX6 Delay (Cohorts 2 and 4)

In case of mFOLFOX6 treatment delay beyond 2 days, mFOLFOX6 should be administered at the next scheduled zolbetuximab treatment visit, which would now be considered day 1 of the next cycle.

A delay of mFOLFOX6 treatment for > 28 days from when the next mFOLFOX6 treatment was scheduled to be administered (> 42 days from when the last dose of mFOLFOX6 began) due to unresolved toxicity associated with mFOLFOX6, will result in the subject discontinuing mFOLFOX6 (all components).

5.1.2.3 Pembrolizumab Delay (Cohort 3)

In case of pembrolizumab treatment delay beyond 7 days, pembrolizumab should be administered at the next scheduled zolbetuximab treatment visit, which would now be considered day 1 of the next cycle.

A delay of pembrolizumab treatment for > 28 days from when the next pembrolizumab treatment was scheduled to be administered (> 49 days from when the last dose of pembrolizumab began) due to unresolved toxicity associated with pembrolizumab, will result in the subject discontinuing pembrolizumab.

5.1.2.4 Nivolumab Delay (Cohort 4)

In case of nivolumab treatment delay beyond 2 days, nivolumab should be administered at the next scheduled treatment visit.

A delay of nivolumab treatment for > 28 days from when the next nivolumab treatment was scheduled to be administered (> 42 days from when the last dose of nivolumab began) due to unresolved toxicity associated with nivolumab, will result in the subject discontinuing nivolumab.

5.1.2.5 FLOT Delay (Cohort 5)

If FLOT treatment is delayed more than 2 days, then the next dose will be administered on day 1 of the following cycle.

A delay of FLOT treatment for > 28 days from when the next FLOT treatment was scheduled to be administered (> 42 days from when the last dose of FLOT began) due to unresolved toxicity associated with FLOT, will result in the subject discontinuing FLOT (all components).

5.1.2.6 Discontinuation of mFOLFOX6 Components

If a subject has 5-FU discontinued or withheld, oxaliplatin should be discontinued or withheld until 5-FU is resumed. For additional information, refer to the approved Package Insert, SPC or local product information supplied by the manufacturer for each agent (oxaliplatin, leucovorin or folinic acid, 5-FU).

5.1.2.7 Continuation of mFOLFOX6 (or Some Components, with or without Nivolumab) if Zolbetuximab is Discontinued First

If zolbetuximab is discontinued first for reasons other than radiographic progression, subjects may continue to receive mFOLFOX6 (or its components as described above, with or without nivolumab) until radiographic disease progression, toxicity requiring cessation, start of another anti-cancer treatment or other treatment discontinuation criteria is met. Subjects should continue to follow the Study Treatment Period schedule of assessments.

5.1.2.8 Continuation of Zolbetuximab if mFOLFOX6 (with or without Nivolumab) or Pembrolizumab is Discontinued First

If mFOLFOX6 (all or some components, with or without nivolumab) or pembrolizumab are discontinued first for reasons other than radiographic PD and no other anti-cancer treatment is started in the absence of radiographic progression, subjects may continue to receive zolbetuximab as a single agent until radiographic disease progression, toxicity requiring cessation, start of another anti-cancer treatment or other treatment discontinuation criteria are met, provided that all of the following have been met:

- The subject completed at least 1 cycle (42 days) of mFOLFOX6 or 2 cycles of pembrolizumab (42 days) treatment;
- The subject will not receive or has not received other chemotherapy;
- And in the investigator's opinion the subject continues to derive clinical benefit with acceptable toxicity.

5.1.2.9 Continuation of Pembrolizumab if Zolbetuximab is Discontinued First

If zolbetuximab is discontinued first for reasons other than PD and no other anti-cancer treatment is started in the absence of progression, subjects may continue to receive pembrolizumab as a single agent until disease progression, toxicity requiring cessation, start of another anti-cancer treatment or other treatment discontinuation criteria are met. Subjects without disease progression will receive pembrolizumab for up to 24 months.

5.1.2.10 Continuation of Nivolumab if Zolbetuximab is Discontinued First

If zolbetuximab is discontinued first for reasons other than radiographic PD and no other anti-cancer treatment is started in the absence of radiographic progression, subjects may continue to receive nivolumab until radiographic disease progression, toxicity requiring cessation, start of another anti-cancer treatment or other treatment discontinuation criteria is met. Subjects should continue to follow the Study Treatment Period schedule of assessments.

5.1.2.11 Discontinuation of Both Zolbetuximab and mFOLFOX6 (All Components, with or without Nivolumab) or Pembrolizumab

If both zolbetuximab and mFOLFOX6 (all components, with or without nivolumab) or pembrolizumab are discontinued for reasons other than radiographic progressive disease as confirmed by independent central reader, the subject should enter the post-treatment follow-up period and continue to undergo imaging assessments per the Schedule of Assessments until radiographic progression is documented per the independent central reader or investigator (Cohort 4 only) or the subject starts another anti-cancer treatment, whichever occurs earlier.

5.1.2.12 Continuation of FLOT (or Some Components) if Zolbetuximab is Discontinued First

If zolbetuximab is discontinued first for reasons other than radiographic PD, subjects may continue to receive FLOT (or its components as described above) until completion of therapy, radiographic disease progression, toxicity requiring cessation, start of another anti-cancer treatment or other treatment discontinuation criteria is met. Subjects should continue to follow the Study Treatment Period schedule of assessments. If zolbetuximab was discontinued early during preoperative treatment, subjects may receive FLOT with or without zolbetuximab for postoperative treatment.

5.1.3 Increase or Reduction in Dose of the Study Drug

Dose increase and dose reduction for zolbetuximab are not allowed. BSA should only be recalculated if there is a weight change of at least 10%.

5.1.3.1 Zolbetuximab

Dose interruption, slower infusion or discontinuation will be permitted for the management of toxicity. A delay of zolbetuximab treatment for > 28 days from when the next zolbetuximab treatment was scheduled to be administered (> 49 days from when the last dose of zolbetuximab began, > 42 days in Cohort 4) due to unresolved toxicity associated with zolbetuximab, will result in the subject discontinuing zolbetuximab.

Table 16 Infusion-related Reactions

**Refer to Table 17 for management of infusion-related reactions of nausea/vomiting/abdominal pain**	
**CTCAE v4.03 Grade**	**Management**
Grade 1 standard infusion reactions *other than nausea, vomiting or abdominal pain*[#]	Continue infusion and closely monitor the subject.
Grade 2 standard infusion reaction *other than nausea, vomiting or abdominal pain*[#]	Interrupt. Medical management as per type of reaction. Resume infusion once toxicity Grade ≤ 1 and reduce the infusion rate for the remaining infusion*.<br>For the next infusion:<br>• Increase infusion time.<br>• Premedicate as appropriate**.<br>• Closely monitor the subject for symptoms and signs of an infusion reaction.
Any infusion reaction with features of anaphylaxis<br>**OR**<br>Grade 3 or 4 standard infusion reactions or any reaction with features of anaphylaxis *other than nausea, vomiting or abdominal pain*[#]	Stop the infusion immediately. Institute appropriate medical management immediately based on the type of reaction. Once the subject has been stabilized, collect blood for cytokine/chemokine panel (ad hoc collection for shipment to central laboratory).<br>If the reaction is suggestive of anaphylaxis, collect blood (standard red top tube) for serum total tryptase level (levels typically peak within 3 hours after the onset of symptoms). Serum should be frozen if the assay cannot be performed promptly at the central laboratory.

CTCAE v4.03: Common Terminology Criteria for Adverse Events

*The infusion may be interrupted as long as the administration of the zolbetuximab infusion does not exceed the expiration period of the infusion solution.

** At the investigator's discretion, anti-histamines may be used as premedication for the next infusion. Systemic corticosteroids should be avoided or minimized while subject is on study treatment unless required for management of an emergent medical condition (e.g., severe nausea/vomiting or hypersensitivity reaction).

[#] For grade 3 or 4 infusion-related reactions of nausea, vomiting or abdominal pain, collect blood for cytokine/chemokine panel and serum total tryptase level.

Table 17 Guidelines for Zolbetuximab Treatment Modification Due to Non-hematologic Toxicity

Toxicity	Grade 1	Grade 2	Grade 3	Grade 4
**Infusion-related reaction (IRR)**<br>See Table 16 for further guidance	Continue Infusion	Interrupt infusion.<br>Infusion may be resumed at a reduced rate when toxicity has improved to Grade ≤ 1.	Stop the infusion immediately.<br>Institute appropriate medical management immediately based on the type of reaction.<br>Permanently discontinue zolbetuximab	
**Nausea**	Continue Infusion	Interrupt the infusion until Grade ≤ 1, then resume at a reduced infusion rate for the remaining infusion. For the next infusion, administer per the infusion rates in Table 15.	Interrupt infusion. Hold zolbetuximab treatment until toxicity has improved to Grade ≤ 1, then restart the infusion at a lower rate.<br>If the investigator determines that the toxicity is not related to zolbetuximab and the toxicity has improved to Grade ≤ 2, then infusion may be restarted at the investigator's discretion at a lower rate.	Permanently Discontinue zolbetuximab
**Vomiting**	Continue Infusion	Interrupt the infusion until Grade ≤ 1, then resume at a reduced infusion rate for the remaining infusion. For the next infusion, administer per the infusion rates in Table 15.	Interrupt infusion. Hold zolbetuximab treatment until toxicity has improved to Grade ≤ 1 and then restart infusion at a lower rate.	Permanently Discontinue zolbetuximab
**Other Non-Hematologic toxicity**	Continue Infusion		Interrupt infusion. Hold zolbetuximab treatment until toxicity has improved to Grade ≤ 1.<br>If the investigator determines that the toxicity is not related to zolbetuximab and the toxicity has improved to Grade ≤ 2, then infusion may be restarted at the investigator's discretion.*	Permanently Discontinue zolbetuximab
**PRES**	Discontinue zolbetuximab if PRES is suspected.			

PRES: posterior reversible encephalopathy syndrome.

Note: These guidelines should be followed regardless of investigator assessment of relationship to zolbetuximab.

* For subjects with a pulmonary embolism, treatment can continue without resolved to grade 2 or less.

Table 18 Zolbetuximab: Dose Modifications for Hematologic Toxicity

Toxicity	Grade 1	Grade 2	Grade 3	Grade 4
**Neutropenia**	ANC < LLN - 1.5 × 10^9/L	ANC 1.0-1.5 × 10^9/L	ANC 0.5-1.0 × 10^9/L	ANC < 0.5 × 10^9/L
➢ **Action**	Continue treatment		Hold treatment and recheck blood counts weekly until ANC resolves to ≥ 1.5 × 10^9/L before restarting treatment. Discontinue zolbetuximab if ANC remains < 1.5 × 10^9/L after a 28-day delay.	
➢ **Dose Modification**	Maintain dose level		Dose reduction for zolbetuximab will not be allowed.	
**Febrile Neutropenia†**			Grade 3 (ANC < 1.0 × 10^9/L and fever ≥ 38.5°C)	Grade 4 (life-threatening consequences including septic shock, hypotension, acidosis)
➢ **Action**			Follow standard treatment guidelines. Hold treatment and recheck blood counts weekly until fever has resolved and ANC recovers to ≥ 1.5 × 10^9/L. Discontinue zolbetuximab if ANC remains < 1.5 × 10^9/L after a 28 day delay.	
➢ **Dose Modification**			Dose reduction for zolbetuximab will not be allowed	
**Thrombocytopenia**	PLT < LLN - 75 × 10^9/L	PLT 50-75 × 10^9/L	PLT 25-50 × 10^9/L	PLT < 25 × 10^9/L
➢ **Action**	Continue treatment		Withhold treatment and recheck blood counts weekly until platelets recover to > 75× 10^9/L before restarting treatment. Discontinue zolbetuximab if PLT remains < 75 × 10^9/L after a 28 day delay.	
➢ **Dose Modification**	Maintain dose level		Dose reduction for zolbetuximab will not be allowed.	
**Anemia**	Hgb < LLN - 10.0 g/dL	Hgb < 10.0 - 8.0 g/dL	Hgb < 8.0 g/dL	Life-threatening consequences
➢ **Action**	Continue treatment		Withhold treatment, transfuse if indicated and recheck blood counts weekly until Hgb recovers to > 8.0 g/dL before restarting treatment. Discontinue zolbetuximab if Hgb remains < 8.0 g/dL after a 28 day delay.	Withhold treatment. Urgent intervention required following the standard treatment and transfuse blood. The subject may be discontinued after discussion with Medical Monitor.
➢ **Dose Modification**	Maintain dose level		Dose reduction for zolbetuximab will not be allowed.	

ANC: absolute neutrophil count; PLT: platelet count; Hgb: Hemoglobin
Note: These guidelines should be followed regardless of investigator assessment of relationship to zolbetuximab.
† At the investigator's discretion, growth factors may be used according to standard practice guidelines.

5.1.3.2 mFOLFOX6 (Cohorts 2 and 4)

The dose of mFOLFOX6 should not be modified until cycle 1 day 15. After the assessment of tolerability, dose adjustments should be performed based on the investigator's judgment utilizing institutional standard of care, approved package insert, SPC or local product information and/or the recommended criteria in [Table 19] based on maximum hematologic or non-hematologic toxicity data from the previous cycle as shown in Table 20 and Table 21, respectively. Dose reduction criteria for oxaliplatin-related neurotoxicity are presented in Table 22. Each drug may be dose reduced independently based on the specific types of toxicities observed. It is recommended that no more than 2 dose reductions per drug per subject occur (see Table 19). Dose re-escalation is not recommended after treatment-related AEs. If further dose reduction is required beyond the criteria in Table 19, that component of mFOLFOX6 should be discontinued.

In subjects experiencing toxicity requiring a delay in chemotherapy, subject may continue to receive zolbetuximab as clinically appropriate. If chemotherapy is withheld, subject should be evaluated weekly (at a minimum) until the toxicity has resolved to Grade ≤ 1 or has stabilized sufficiently. Upon resolution of the toxicity, subject should be retreated as described in Table 20, Table 21 or Table 22 (as applicable). A delay of mFOLFOX6 treatment for > 28 days from when the next mFOLFOX6 treatment was scheduled to be administered (> 42 days from when the last dose of mFOLFOX6 began) due to unresolved toxicity associated with mFOLFOX6, will result in the subject discontinuing mFOLFOX6 (all components).

Prior to each 5-FU dose, subjects should receive folinic acid (400 mg/m^2 [or such dose deemed appropriate by the investigator]), or if unavailable, levofolinic acid (200 mg/m^2 [or such dose deemed appropriate by the investigator]). If both folinic acid and levofolinic acid are unavailable, they may be omitted from the treatment regimen. Folinic or levofolinic acid should be delayed if 5-FU treatment is delayed.

Table 19 Recommended Dose Adjustment Levels for Oxaliplatin and 5-FU

Drug		Oxaliplatin	5-FU Bolus	5-FU Infusion
**Initial Dose**		85 mg/m^2	400 mg/m^2	2400 mg/m^2
**Dose Reduction**	**Level 1**	65 mg/m^2	320 mg/m^2	1900 mg/m^2
	**Level 2**	50 mg/m^2	260 mg/m^2	1500 mg/m^2

If a subject has 5-FU discontinued or withheld, oxaliplatin should be discontinued or withheld until 5-FU is resumed. Subjects with delay or discontinuation of chemotherapy agent(s) may continue to receive zolbetuximab if clinically appropriate.

Dose modifications will be made based on the following criteria.

5.1.3.2.1 mFOLFOX6: Dose Modifications for Hematologic Toxicity

The chemotherapy dose modifications for hematologic toxicity are presented in Table 20. Dose modifications should be maintained until recovery from hematologic toxicity. A delay of mFOLFOX6 treatment for > 28 days from when the next mFOLFOX6 treatment was

scheduled to be administered (> 42 days from when the last dose of mFOLFOX6 began) due to hematologic toxicity associated with mFOLFOX6, will result in the subject discontinuing mFOLFOX6 (all components).

Table 20 mFOLFOX6 Dose Modification Due to Hematologic Toxicity

**Toxicity**	**Grade 1**	**Grade 2**	**Grade 3**	**Grade 4**
**Neutropenia**	ANC < LLN - 1.5 × 10^9/L	ANC < 1.5 - 1.0 × 10^9/L	ANC < 1.0 - 0.5 × 10^9/L	ANC < 0.5 × 10^9/L
➢ **Action**	Continue treatment.		Hold treatment and recheck blood counts weekly until ANC resolves to ≥ 1.5 × 10^9/L before restarting treatment. Discontinue mFOLFOX6 if ANC remains < 1.5 × 10^9/L after a 28 day delay.	
➢ **Dose Modification**	Maintain dose level.		Reduce oxaliplatin by one dose level.† If oxaliplatin is held, omit 5-FU bolus.	<u>First event</u>: Omit 5-FU bolus and reduce oxaliplatin by one level.† <u>Second event</u>: Reduce 5-FU infusion and oxaliplatin one dose level.†
**Febrile Neutropenia†**			Grade 3 (ANC < 1.0 × 10^9/L and fever ≥ 38.5°C)	Grade 4 (life-threatening consequences including septic shock, hypotension, acidosis)
➢ **Action**			Follow standard treatment guidelines	Proceed to next treatment cycle when fever has resolved and ANC recovers to ≥ 1.5 × 10^9/L
➢ **Dose Modification**			<u>First event</u>: Omit 5-FU bolus and reduce oxaliplatin by one level.† <u>Second event</u>: Reduce 5-FU infusion and oxaliplatin one dose level.†	
**Thrombocytopenia**	PLT < LLN - 75 × 10^9/L	PLT < 50 - 75 × 10^9/L	PLT < 25 - 50 × 10^9/L	PLT < 25 × 10^9/L

Table continued on next page

Toxicity	Grade 1	Grade 2	Grade 3	Grade 4
➢ **Action**	Continue treatment.		Withhold treatment and recheck blood counts weekly until platelets recover to > 75× 10^9/L before restarting treatment. Discontinue mFOLFOX6 if PLT remains < 75 × 10^9/L after a 28 day delay.	
➢ **Dose Modification**	Maintain dose level.		Reduce oxaliplatin one dose level. Omit bolus 5-FU if the subject is not receiving oxaliplatin.	<u>First event</u>:<br>Omit 5-FU bolus and reduce oxaliplatin by one level.[†]<br><br><u>Second event</u>:<br>Reduce 5-FU infusion and oxaliplatin one dose level.[†]

5-FU: fluorouracil; ANC: absolute neutrophil count; PLT: platelet count

† At the investigator’s discretion growth factors, supportive therapeutic and prophylactic actions may be used according to standard practice guidelines, upon occurrence of complicated neutropenia (e.g., febrile neutropenia, documented infection with neutropenia, neutropenia lasting more than 7 days or grade 4 neutropenia).

5.1.3.2.2 mFOLFOX6: Dose Modification for Non-Hematologic Toxicity

Chemotherapy dose modifications for non-hematologic toxicity should be based on the most severe toxicity experienced during the last treatment (Table 21). Retreatment should be delayed until recovery of all non-hematologic toxicity to ≤ Grade 1 with the exception of increased bilirubin or ALT, which must recover to Grade 1 or baseline, whichever was higher. The maximum permitted treatment delay is 28 days from when the next study treatment was scheduled to be administered (42 days from when the last dose of mFOLFOX6 began) for recovery of non-hematologic toxicity. If after a 28-day delay, the subject has not recovered sufficiently to meet retreatment criteria, mFOLFOX6 should be discontinued.

ECG is required to be performed and assessed locally prior to every oxaliplatin infusion (before any antiemetic treatment) and following completion of every oxaliplatin infusion. ECG should be performed up to 48 hours *prior* to and up to 6 hours *following* every oxaliplatin infusion. Oxaliplatin administration and electrolyte levels should be managed according to the investigator’s judgment for subjects with grade 1 or 2 hypokalemia, hypomagnesemia and/or hypocalcemia.

During or following study treatment, additional ECG monitoring should be initiated for subjects who experience syncope, presyncope, palpitations and/or bradycardia per local standard of care.

- If the QTc interval is > 450 msec, medically manage per local standard of care, including correction of hypokalemia, hypomagnesemia, and/or hypocalcemia.
- If the QTc interval is > 500 msec, medically manage per local standard of care, withhold oxaliplatin and 5-FU treatment, ensure appropriate (continuous) ECG monitoring, and obtain cardiology consultation. If the QTc interval resolves to ≤ 450 msec, the subject may resume treatment at 1 reduced dose level.

Table 21 mFOLFOX6 Dose Modification Due to Non-hematologic Toxicity

Toxicity	Grade 1	Grade 2	Grade 3	Grade 4
**Diarrhea**				
➢ **Action**	Continue treatment.	Start medical management for diarrhea. Continue treatment.	Start medical management for diarrhea. Withhold all treatment. Restart treatment after diarrhea recovers to ≤ Grade 1	
➢ **Dose Modification**	None	Maintain dose level. If Grade 2 diarrhea persists despite medical management, reduce 5-FU bolus and infusion one dose level.	Reduce 5-FU bolus and 5-FU infusion one dose level.	Reduce 5-FU bolus, 5-FU infusion and oxaliplatin 1 dose level
**Other Non-Hematologic Toxicities Attributable to mFOLFOX6[†‡]**				
➢ **Action**			Withhold all treatment until toxicity improves to ≤ Grade 1.	
➢ **Dose Modification**			Reduce dose of drug(s) responsible for toxicity by one dose level. At the investigator's discretion, the 5-FU bolus may be omitted in lieu of or in conjunction with dose reduction(s) in the 5-FU infusion and/or oxaliplatin.	

5-FU: fluorouracil

† For mucositis/stomatitis, decrease only 5-FU, not oxaliplatin.

‡ Exceptions: alopecia, fatigue, anorexia, nausea/vomiting (if can be controlled by antiemetics) and constipation (if can be controlled with laxatives, stool softeners, etc.).

5.1.3.3 Oxaliplatin-Induced Neurotoxicity

Oxaliplatin is known to be associated with peripheral neuropathy, including paresthesia and dysesthesia of the hands, feet and perioral region. Subjects treated with oxaliplatin in this study should be advised to avoid cold drinks and exposure to cold water or air, especially within 3 to 5 days of receiving oxaliplatin. Dose modifications for oxaliplatin related to neurotoxicity are presented in Table 22. Oxaliplatin should be discontinued in the event of Grade 3 or 4 neurotoxicity according the criteria in Table 22. Cases of PRES have been reported in subjects receiving oxaliplatin combination chemotherapy. Discontinue oxaliplatin if PRES is suspected. Confirm PRES diagnosis by brain imaging, preferably MRI.

Table 22 Oxaliplatin Dose Modification for Associated Neurotoxicity

<table>
<tr><th>Toxicity/Duration</th><th>Grade 1</th><th>Grade 2</th><th>Grade 3</th><th>Grade 4</th></tr>
<tr><td>Paresthesia or dysesthesia</td><td>Paresthesia or dysesthesia[‡] that does not interfere with function</td><td>Paresthesia or dysesthesia[‡], interfering with function, but not ADL</td><td>Paresthesia or dysesthesia[‡] with pain or with functional impairment that also interferes with ADL</td><td>Persistent paresthesia or dysesthesia that is disabling or life-threatening</td></tr>
<tr><td>1 to 7 Days</td><td rowspan="3">No dose reduction</td><td rowspan="2">No dose reduction</td><td><u>First Event:</u>
Reduce oxaliplatin by one dose level.
<u>Second Event:</u>
Reduce oxaliplatin by a second dose level.</td><td rowspan="3">Discontinue oxaliplatin</td></tr>
<tr><td>➢ 7 Days</td><td>Discontinue oxaliplatin</td></tr>
<tr><td>Persistent between cycles†</td><td>Reduce oxaliplatin by one dose level</td><td>Discontinue oxaliplatin</td></tr>
<tr><td>Acute laryngopharyngeal dysesthesia[‡]
(during or after the 2-hour infusion)</td><td colspan="4">Discontinue current infusion
Increase duration of next infusion to 6 hours; may also pretreat with benzodiazepines</td></tr>
</table>

ADL: activities of daily living; NA: not applicable
† Not resolved by the beginning of the next cycle.
‡ May be cold induced.

5.1.3.3.1 Oxaliplatin-Induced Laryngopharyngeal Dysesthesia

Oxaliplatin has also been associated with laryngopharyngeal dysesthesia, which is an unusual loss of sensation of breathing (acute respiratory distress) without any objective evidence of respiratory distress (hypoxia, laryngospasm or bronchospasm). Laryngopharyngeal dysesthesia may be induced or exacerbated upon exposure to cold.

Subjects developing laryngopharyngeal dysesthesia should have their oxygen saturation evaluated via a pulse oximeter. If results are normal, reassurance should be provided, a benzodiazepine or other anxiolytic agent should be considered, and the subject should remain for observation in the clinic until the episode has resolved. After resolution, the oxaliplatin infusion may then be continued at one third the rate.

Because laryngopharyngeal dysesthesia may be associated with the rate of oxaliplatin infusion, subsequent infusions of oxaliplatin should be prolonged from a normal 2-hour infusion to a 6-hour infusion.

Subjects receiving oxaliplatin should avoid consuming cold drinks or ice chips on day 1 of each cycle, as this may exacerbate oral or throat dysesthesia, as well as laryngopharyngeal

dysesthesia. Administration of prophylactic medication such as Mg^{2+}/Ca^{2+} infusions or others is at the discretion of the investigator.

The symptoms and treatments of laryngopharyngeal dysesthesia and platinum HSRs are compared in Table 23.

Table 23 Comparison of the Symptoms and Treatment of Laryngopharyngeal and Platinum Hypersensitivity Reactions

Clinical Symptoms	Laryngopharyngeal Dysesthesia	Platinum Hypersensitivity
Dyspnea	Present	Present
Bronchospasm	Absent	Present
Laryngospasm	Absent	Present
Anxiety	Present	Present
O_2 saturation	Normal	Decreased
Difficulty swallowing	Present (loss of sensation)	Absent
Pruritus	Absent	Present
Urticaria/rash	Absent	Present
Cold-induced symptoms	Yes	No
Blood pressure	Normal or increased	Normal or decreased
Treatment	Reassurance, anxiolytics, observation in a controlled clinical setting until symptoms abate or at the physician's discretion	Oxygen, steroids, epinephrine, bronchodilators; fluids and vasopressors, if appropriate

5.1.3.3.2 Allergic Reaction to Oxaliplatin

Subjects developing Grade 1 or 2 allergic reaction to oxaliplatin should receive premedication according to institutional practice prior to further administration of oxaliplatin. Appropriate premedication should also be given if Grade 1 to 2 allergic reaction persists into the next cycle. Oxaliplatin should be discontinued in subjects developing Grade 3 to 4 allergic reactions.

Oxaliplatin should be interrupted pending further investigation in subjects experiencing respiratory symptoms indicative of pulmonary fibrosis such as nonproductive cough, dyspnea, crackles, rales, hypoxia, tachypnea or radiological pulmonary infiltrates. Oxaliplatin should be permanently discontinued in subjects with confirmed interstitial pulmonary fibrosis.

5.1.3.3.3 Extravasation of Oxaliplatin

Necrosis has been seen in conjunction with extravasation of oxaliplatin. Subjects with suspected extravasation should have their infusion stopped and the drug administered at another site. Extravasation should be treated according to institutional guidelines.

5.1.3.4 Dose Modifications for Pembrolizumab

No dose reductions of pembrolizumab are recommended. Withhold or discontinue pembrolizumab to manage adverse reactions as described in Table 24.

Table 24 Recommended Dose Modifications for Adverse Reactions – Pembrolizumab

Adverse Reaction	Severity*	Dose Modification for Pembrolizumab
Immune-mediated pneumonitis	Grade 2	Withhold†
	Grade 3 or 4 or recurrent Grade 2	Permanently discontinue
Immune-mediated colitis	Grade 2 or 3	Withhold†
	Grade 4	Permanently discontinue
Immune-mediated hepatitis	AST or ALT > 3 but ≤ 5 × ULN or total bilirubin > 1.5, but no more than 3 × ULN	Withhold
Immune-mediated endocrinopathies	Grade 3 or 4	Withhold until clinically stable
Immune-mediated nephritis	Grade 2	Withhold
	Grade 3 or 4	Permanently discontinue
Immune-mediated skin adverse reactions	Grade 3 or suspected SJS or TEN	Withhold
	Grade 4 or confirmed SJS or TEN	Permanently discontinue
Other immune-mediated adverse reactions	Grade 2 or 3 based on the severity and type of reaction	Withhold†
	Grade 3 based on the severity and type of reaction or Grade 4	Permanently discontinue
Recurrent immune-mediated adverse reactions	Recurrent Grade 2 pneumonitis<br>Recurrent Grades 3 or 4	Permanently discontinue
Inability to taper corticosteroid	Requirement for 10 mg per day or greater prednisone or equivalent for more than 12 weeks after last dose of pembrolizumab	Permanently discontinue
Persistent Grade 2 or 3 adverse reaction (excluding endocrinopathy)	Grades 2 or 3 adverse reactions lasting 12 weeks or longer after last dose of pembrolizumab	Permanently discontinue
Infusion-related reactions	Grade 1 or 2	Interrupt or slow the rate of infusion
	Grade 3 or 4	Permanently discontinue

ALT: alanine aminotransferase; AST: aspartate aminotransferase; SJS: Stevens-Johnson Syndrome; TEN toxic epidermal necrolysis: ULN: upper limit of normal.

* Toxicity was graded per National Cancer Institute Common Terminology Criteria for Adverse Events, version 4.0 (NCI-CTCAE v4).

† Resume in subjects with complete or partial resolution (Grade 0 or 1) after corticosteroid taper.

Source: [KEYTRUDA Prescribing Information]

5.1.3.5 Dose Modifications for Nivolumab

Modify nivolumab to manage adverse reactions as described in [Table 25].

Table 25 Recommended Dose Modifications – Nivolumab

Adverse Reaction	Severity*	Dose Modification for Nivolumab
Colitis	Grade 2 diarrhea or colitis	Withhold dose†
	Grade 3 diarrhea or colitis	Withhold dose†
	Grade 4 diarrhea or colitis	Permanently discontinue
Pneumonitis	Grade 2 pneumonitis	Withhold dose†
	Grade 3 or 4 pneumonitis	Permanently discontinue
Hepatitis	AST or ALT 3 to 5 x ULN or total bilirubin 1.5 to 3 x ULN	Withhold dose†
	AST or ALT > 5 x ULN or total bilirubin > 3 x ULN	Permanently discontinue
Hypophysitis	Grade 2 or 3 hypophysitis	Withhold dose†
	Grade 4 hypophysitis	Permanently discontinue
Adrenal Insufficiency	Grade 2 adrenal insufficiency	Withhold dose†
	Grade 3 or 4 adrenal insufficiency	Permanently discontinue
Type I Diabetes Mellitus	Grade 3 hyperglycemia	Withhold dose†
	Grade 4 hyperglycemia	Permanently discontinue
Nephritis and Renal Dysfunction	Serum creatinine > 1.5 to 6 x ULN	Withhold dose†
	Serum creatinine > 6 x ULN	Permanently discontinue
Skin	Grade 3 rash or suspected SJS or TEN	Withhold dose†
	Grade 4 rash or confirmed SJS or TEN	Permanently discontinue
Encephalitis	New-onset moderate or severe neurologic signs or symptoms	Withhold dose†
	Immune-mediated encephalitis	Permanently discontinue
Other	Other Grade 2 adverse reaction	Withhold dose until events are reduced to a Grade 1 or Grade 0. Subjects may continue to receive nivolumab with close monitoring and use of premedication according to local treatment guidelines for prophylaxis of infusion reactions
	Other Grade 3 adverse reaction<br>First occurrence<br>Recurrence of same Grade 3 adverse reaction	Withhold dose†<br>Permanently discontinue
	Severe or Life-threatening (Grade 3 or Grade 4) adverse reaction	Permanently discontinue
	Grade 2 myocarditis<br><br>Grade 3 myocarditis	Withold dose until symptoms resolve and management with corticosteroids is complete.<br><br>Permanently discontinue
	Requirement for ≥ 10 mg/day prednisone or equivalent for more than 12 weeks	Permanently discontinue
	Persistent Grade 2 or 3 adverse reactions lasting 12 weeks or longer	Permanently discontinue

ALT: alanine aminotransferase; AST: aspartate aminotransferase; SJS: Stevens-Johnson Syndrome; TEN: toxic epidermal necrolysis; ULN: upper limit of normal

* Toxicity was graded per National Cancer Institute Common Terminology Criteria for Adverse Events, version 4.0 (NCI-CTCAE v4).

† Resume treatment when adverse reaction improves to Grade 0 or 1.

‡ Resume treatment when AST/ALT returns to baseline.

Source: [OPDIVO Prescribing Information]

5.1.3.6 Dose Modifications for FLOT

Table 26 Recommended Dose Modifications – FLOT

Drug		Oxaliplatin	Docetaxel	5-FU Infusion
**Initial Dose**		85 mg/m^2	50 mg/m^2	2600 mg/m^2
**Dose Reduction**	**Level 1**	64 mg/m^2	37.5 mg/m^2	1950 mg/m^2
	**Level 2**	42.5 mg/m^2	25 mg/m^2	1300 mg/m^2

5-FU: fluorouracil

Level 1: this is 25% reduction of initial dose.

Dose Reduction Level 2: this is 50% reduction of initial dose.

5.1.3.7 Dose Modifications for FLOT Due to Hematologic Toxicity

Table 27 Recommended Dose Modifications for FLOT Due to Hematologic Toxicity

Toxicity	Grade 1	Grade 2	Grade 3	Grade 4
**Neutropenia**	ANC < LLN- 1.5 × 10^9/L	ANC < 1.5- 1.0 × 10^9/L	ANC < 1.0- 0.5 × 10^9/L	ANC < 0.5 × 10^9/L
➢ **Action**	Continue treatment.		Hold treatment and recheck blood counts weekly until ANC resolves to ≥ 1.5 × 10^9/L before restarting treatment. Discontinue FLOT if ANC remains < 1.5 × 10^9/L after a 28-day delay.	
➢ **Dose Modification**	Maintain dose level.		Reduce oxaliplatin and docetaxel by one dose level.	First event: Reduce oxaliplatin, docetaxel and 5-FU infusion by one dose level of the initial dose. Second event: Reduce oxaliplatin, docetaxel and 5-FU by two dose levels of the initial dose.
**Febrile Neutropenia†**			Grade 3 (ANC < 1.0 × 10^9/L and fever ≥ 38.5°C/101°F).	Grade 4 (life-threatening consequences including septic shock, hypotension, acidosis).
➢ **Action**			Follow standard treatment guidelines.	Hold treatment until fever has resolved and ANC recovers to ≥ 1.5 × 10^9/L, before restarting treatment.
➢ **Dose Modification**			First event: Reduce oxaliplatin, docetaxel and 5-FU infusion by one dose level of the initial dose. Second event: Reduce oxaliplatin, docetaxel and 5-FU by two dose levels of the initial dose.	

Table continued on next page

Toxicity	Grade 1	Grade 2	Grade 3	Grade 4
**Thrombocytopenia**	PLT < LLN- 75 $\times 10^9$/L	PLT < 50-75 $\times 10^9$/L	PLT < 25-50 $\times 10^9$/L	PLT < 25 $\times 10^9$/L
➢ **Action**	Continue treatment.		Withhold treatment and recheck blood counts weekly until platelets recover to > 75$\times 10^9$/L before restarting treatment. Discontinue FLOT if PLT remains < 75 $\times 10^9$/L after a 28 day delay.	
➢ **Dose Modification**	Maintain dose level.		Reduce oxaliplatin and docetaxel by one dose level.	<u>First event</u>:<br>Reduce oxaliplatin, docetaxel and 5-FU infusion by one dose level of the initial dose.<br><u>Second event</u>:<br>Reduce oxaliplatin, docetaxel and 5-FU by two dose levels of the initial dose.

5-FU: fluorouracil; ANC: absolute neutrophil count; FLOT: fluorouracil, leucovorin or folinic acid, oxaliplatin and docetaxel; PLT: platelet count.

† At the investigator’s discretion, growth factors may be used according to standard practice guidelines.

5.1.3.8 Dose Modifications for FLOT Due to Non-hematologic Toxicity

Table 28 Recommended Dose Modifications for FLOT Due to Non-hematologic Toxicity

Toxicity	Grade 1	Grade 2	Grade 3	Grade 4
**Diarrhea**				
➢ **Action**	Continue treatment.	Start medical management for diarrhea. Continue treatment.	Start medical management for diarrhea. Withhold all treatment. Restart treatment after diarrhea recovers to ≤ Grade 1	
➢ **Dose Modification**	None	Maintain dose level. If Grade 2 diarrhea persists despite medical management, reduce 5-FU infusion one dose level of the initial dose.	First event: Reduce 5-FU infusion one dose level of the initial dose. Second event: Reduce docetaxel one dose level of the initial dose	First event: Reduce docetaxel and 5-FU infusion and oxaliplatin one dose level of the initial dose. Second event: Discontinue FLOT treatment.
**Stomatitis/Mucositis**				
➢ **Action**	Continue treatment.	Start medical management for stomatitis/mucositis. Continue treatment.	Start medical management for stomatitis/mucositis. Withhold all treatment. Restart treatment after stomatitis/mucositis recovers to ≤ Grade 1	
➢ **Dose Modification**	None	Maintain dose level. If Grade 2 stomatitis/mucositis persists despite medical management, reduce 5-FU infusion one dose level of the initial dose.	First event: Reduce 5-FU infusion one dose level of the initial dose. Second event: Discontinue 5-FU only, at all subsequent cycles. Third event: Reduce docetaxel one dose level of the initial dose.	First event: Discontinue 5-FU only, at all subsequent cycles. Second event: Reduce docetaxel one dose level of the initial dose.
**Other Non-Hematologic Toxicities Attributable to FLOT**				
➢ **Action**			Withhold all treatment until toxicity improves to ≤ Grade 1.	
➢ **Dose Modification**			Reduce dose of drug(s) responsible for toxicity by one dose level of the initial dose. At the investigator's discretion, the 5-FU infusion may be reduced in conjunction with dose reduction(s) in docetaxel and/or oxaliplatin.	

5-FU: fluorouracil; FLOT: fluorouracil, leucovorin or folinic acid, oxaliplatin and docetaxel

If creatinine clearance is reduced to < 40 but > 30 mL/min, hyperhydration for 48 hours will be done and assessment of creatinine clearance repeated. If reduction of renal function persists (30-40 mL/min), oxaliplatin will be reduced to a lower dose level. If a creatinine clearance of <30 mL/min is measured, oxaliplatin treatment will be stopped. 5-FU and docetaxel administration can be continued under consideration of the recommendations given in the respective SPCs.

5.1.3.9 Dose Adjustment for FLOT in Case of Oxaliplatin-related Neurotoxicity

Table 29 Recommended Dose Adjustment for FLOT in Case of Oxaliplatin-related Neurotoxicity

	**Duration of Toxicity**		
**Neurotoxicity**	**≤ 7 days**	**> 7 and < 14**	**Present between cycles**
Cold-induced dysaesthesia	No change	No change	No change
Paraesthesia	No change	No change	Reduction to 75%
Paraesthesia with pain	No change	Reduction to 75%	Stop oxaliplatin*<br>Continue docetaxel/5-FU/leucovorin
Paraesthesia with functional impairment	No change	Reduction to 50%	Stop oxaliplatin*<br>Continue docetaxel/5-FU/leucovorin

5-FU: fluorouracil

*Usually, discontinuation will be permanent; however, administration of oxaliplatin can be resumed (e.g., after complete recovery from the related symptoms) if the investigator decides this is in the best interest of the patient and if the toxicity is not expected to reoccur.

5.1.3.10 Dose Adjustments for FLOT in Case of Other Toxicities

If non-hematologic toxicities ≥ grade 3 appear, the dose of the chemotherapeutic agent most likely responsible for the observed toxicity should be reduced to 75% of the initial dose (for all further cycles). This procedure may apply to grade 2 toxicities per the investigator’s judgment.

In case of repeated toxicity, a further dose reduction to 50% of the initial dose should be performed. If the toxicity reoccurs at the 50% dose level, the investigator should remove the relevant drug(s) or stop the whole treatment.

5.1.4 Previous and Concomitant Treatment (Medication and Non-medication Therapy)

All medications and concomitant treatments administered from the time of informed consent through the 90-day safety follow-up visit must be recorded in the electronic case report form (eCRF). Documentation will include the medication name, indication, route and dates of administration.

Prohibited Concomitant Treatment

The following concomitant medications are strictly prohibited:

- Sorivudine or analogs (during 5-FU treatment)
- Systemic immunosuppressive agents.
  - Concurrent systemic immunosuppressive therapy (including systemic corticosteroids) should be stopped 2 weeks prior to first dose of study drug.
  - Subjects are allowed to use a physiologic replacement dose of hydrocortisone or its equivalent (defined as up to 30 mg per day of hydrocortisone or up to 10 mg per day of prednisone) or a single dose of systemic corticosteroids.
- Other systemic chemotherapy, immunotherapy or other medications intended for antitumor activity.
- Investigational products or therapy other than zolbetuximab.
- Live vaccines should be avoided during the treatment period in which the subject is receiving oxaliplatin or 5-FU and up to 6 months after the final oxaliplatin or 5-FU dose. In cases where a live vaccine is needed for COVID-19 prevention and allowed per local regulations, please contact the Medical Monitor for discussion.

Cautionary Concomitant Treatment

The following should be avoided or used with caution during 5-FU treatment and appropriate monitoring should be conducted:

- CYP2C9 substrates
- Metronidazole and cimetidine
- Anti-epileptic medications (e.g., phenobarbital, phenytoin and primidone)

The following should be avoided or used with caution during oxaliplatin treatment and appropriate monitoring should be conducted:

- Medications known to prolong the QT or QTc interval (refer to https://www.crediblemeds.org for a list of these medications)

The following should be avoided or used with caution during docetaxel treatment and appropriate monitoring should be conducted:

- Strong inhibitors or inducers of CYP3A4

Consideration should be given to avoid or minimize the use of the following concomitant medications, if possible, during zolbetuximab administration:

- Systemic corticosteroids because their impact on the potential efficacy of zolbetuximab, pembrolizumab and nivolumab is not known.
- To minimize risk of Torsades de Pointes, administer 5-HT3 receptor blockers with caution to subjects who have or may develop QTc prolongation.
- Nonsteroidal anti-inflammatory drugs (NSAIDs) because of the potential to cause gastric ulcers.

- In such cases where NSAID use is necessary, the use of NSAIDs with lower gastric ulcerogenic potency is preferred and efficient gastric protection with proton pump inhibitors is warranted.
- Special attention should be paid to Hgb levels to identify covert bleeding.

5.1.5 Treatment Compliance

The dose and schedule of zolbetuximab administered to each subject will be recorded on the appropriate form at every cycle. Reasons for dose delay or omission will also be recorded. This information will be used to assess compliance with the treatment.

The dose and schedule of mFOLFOX6, pembrolizumab and nivolumab administered to each subject will also be recorded on the appropriate form. Reasons for dose delay, reduction or omission will also be recorded.

5.1.6 Criteria for Continuation of Treatment

Zolbetuximab may be made available after conclusion of the study to subjects who are still receiving and benefitting from study treatment until a study-defined treatment discontinuation criterion is met.

5.2 Demographics and Baseline Characteristics

5.2.1 Demographics

Demographic information will be collected for all subjects and will include date of birth (where permitted), age (if date of birth is not allowed) sex, race and ethnicity.

5.2.2 Medical History

Medical history includes all significant medical conditions that have resolved prior to informed consent or are ongoing at the time of main consent. Details that will be collected include the onset date and recovery date and CTCAE grade, if applicable for ongoing conditions.

5.2.3 Diagnosis of the Target Disease, Severity and Duration of Disease

A complete medical history of the target disease will be recorded at screening. This will include the subject’s medical condition, date of initial diagnosis, tumor location, first treatment, gastric progression after first treatment, and other disease specific information as designated in the eCRF.

5.3 Efficacy Assessments

Response and progression will be evaluated in this study using RECIST V1.1. For Cohorts 1A and 3A, imaging will be evaluated at screening and every 6 (± 1) weeks counting from C1D1 for the first 24 weeks, and then every 12 (± 2) weeks thereafter. For Cohort 2, imaging will be evaluated at screening and every 9 (± 1) weeks counting from C1D1 for the first 54 weeks, and then every 12 (± 2) weeks thereafter. For Cohort 4, imaging will be evaluated every 8 (±1) weeks from C1D1 for the first 56 weeks and then every 12 (± 2) weeks thereafter. For Cohort 5, imaging will be evaluated at screening, restaging (after preoperative

chemotherapy prior to surgery) and after a subject discontinues or completes all study treatment (every 3 [± 1] months during the first year, then every 4 [± 2] months during the second year in post-treatment follow-up period. Tumor assessments should be performed on this schedule regardless of whether study treatment has been administered or held.

All radiologically evaluable disease (measurable and/or non-measurable) must be documented at screening and re-assessed at each subsequent tumor evaluation. Imaging of the chest, abdomen, and pelvis, or other areas as clinically indicated (brain, bone, etc.), will include CT scans with contrast unless clinically contraindicated. If CT scan is medically not feasible with contrast, MRI may be used. Bone scans (or focal X-ray) or brain imaging should be performed if metastatic disease is suspected. Disease must be evident by radiology; measurable lesions only. If the sole lesion lies within the field of prior radiotherapy, there must be evidence of disease progression prior to inclusion in the study. The same mode of imaging should be utilized throughout the study unless medical necessity requires change. CT scan performed with PET scan can be used if of quality which allows accurate tumor measurement.

Imaging for Cohorts 1A, 2 and 3A will be sent to an independent central reader within 7 days for the assessment of PFS, ORR, CR and PR based on RECIST 1.1. Central radiographic review assessment will be conveyed to the investigator if the central reader determines that subject has met RECIST 1.1 defined radiographic progression. Imaging for Cohorts 4 and 5 should also be sent to the independent central reader within 7 days. The central radiographic review for these cohorts will occur at the end of study or at a time point to be specified by the sponsor.

Subjects who investigators determined to have clinical progression per investigator assessment (e. g., resistant ascites or effusion) of underlying cancer in absence of meeting RECIST 1.1 criteria based on radiographic progression will also be assessed as disease progression. Investigators should obtain new radiographic imaging for disease assessment if medically feasible for subjects discontinuing study treatments due to clinical progression and send to the independent central reader.

Earliest date when radiographic disease progression or death event was reported will be considered as the date of the PFS event.

Following a PFS event, subjects will enter the survival follow-up period and be followed every 12 weeks for survival status, including date of death, last date known to be alive, etc.

Imaging for Restaging

For subjects in Cohort 5, restaging will be conducted after completion of 4 cycles of preoperative treatment and prior to surgery. Radiological restaging may include imaging, endoscopy and/or laparoscopy.

5.4 Safety Assessment

5.4.1 Vital Signs

Vital signs, including systolic and diastolic blood pressures (mmHg) and radial pulse rate (beats/minute) and temperature will be obtained and recorded at the times specified in the Schedules of Assessments [Table 1, Table 3, Table 4, Table 7, Table 9 and Table 11]. All vital sign measurements will be obtained in a consistent manner (sitting or supine) throughout their study participation. Height and weight will be measured using normal institutional standards.

If clinically significant vital sign changes from baseline (pretreatment) are noted, the changes will be documented as AEs on the AE page of the eCRF. Clinical significance will be defined as a variation in vital signs, which has medical relevance as deemed by the investigator that could result in an alteration in medical care. The investigator will continue to monitor the subject until the parameter returns to Grade ≤ 1, or to the baseline (pretreatment) value, or until the investigator determines that follow-up is no longer medically necessary.

5.4.2 Adverse Events

AEs and SAEs, regardless of causality, will be collected from the time of informed consent through 90 days following the last dose of any study drug, except for inpatient hospitalization for planned procedures as allowed per study (e.g., surgery for subjects in Cohort 5). AEs will be documented at each clinic visit, but can be collected at any time. Any AE that meets the definition of an SAE will also be reported on a separate form to the sponsor.

See [Section 5.5 Adverse Events and Other Safety Aspects] for information regarding AE collection and data handling.

5.4.2.1 Adverse Events of Possible Hepatic Origin

See [Appendix 12.2 Liver Safety Monitoring and Assessment**]** for detailed information on liver abnormalities, monitoring and assessment, if the AE for a subject enrolled in a study and receiving study drug is accompanied by increases in liver function test (LFT) values [e.g., AST, ALT, bilirubin, etc.] or is suspected to be due to hepatic dysfunction.

Subjects with AEs of hepatic origin accompanied by LFT abnormalities should be carefully monitored.

5.4.3 Laboratory Assessments

Below is [Table 30] of the laboratory tests that will be performed during the conduct of the study. See Schedule of Assessments [Table 1, Table 3, Table 4, Table 7, Table 9 and Table 11] for study visit collection dates.

Table 30 Clinical Laboratory Tests

Panel/Assessment	Parameters to be Analyzed
Hematology	Hematocrit (Hct)<br>Hemoglobin (Hgb)<br>Red blood cell count (RBC)<br>White blood cell count (WBC)<br>WBC differential (absolute)<br>Platelets<br>Mean corpuscular volume (MCV)<br>Mean corpuscular hemoglobin (MCH)<br>Mean corpuscular hemoglobin concentration (MCHC)
Biochemistry (fasting not required)	Sodium (Na)<br>Magnesium (Mg)<br>Creatine phosphokinase (CK)<br>Potassium (K)<br>Calcium (Ca)<br>Chloride (Cl)<br>Phosphate (P)<br>Creatinine (Cr)<br>Glucose (Gl)<br>Blood urea nitrogen (BUN)<br>Alkaline phosphatase (ALP)<br>Aspartate aminotransferase (AST)<br>Alanine aminotransferase (ALT)<br>Lactate dehydrogenase (LDH)<br>Bilirubin total (TBL) (total and direct)<br>Total protein (TP)<br>Albumin (Alb)<br>Bicarbonate (HCO3)<br>Thyroid stimulating hormone (TSH)<br>Free thyroxine (T4)<br>Creatinine Clearance
Urinalysis	Color<br>Clarity/turbidity<br>Specific Gravity<br>Nitrites<br>Leukocyte esterase<br>RBCs<br>WBCs<br>Protein<br>Glucose<br>pH<br>Occult blood<br>Bilirubin<br>Urobilinogen
Urine Pregnancy Test	Human chorionic gonadotropin (HCG)
Serum Pregnancy Test	Human chorionic gonadotropin (HCG)
Coagulation Profile	Partial Thromboplastin Time (PTT)<br>Prothrombin time (PT)<br>International normalized ratio (INR)
Grade 3 or 4 Infusion-related Reaction (IRR)	Cytokine/Chemokine†
Any reaction with features of anaphylaxis	Serum total tryptase†
Dihydropyrimidine dehydrogenase (DPD) deficiency screening†	DPD deficiency alleles

†As applicable

Laboratory tests will be performed predose according to the Schedule of Assessments and sent to a central laboratory for analysis. Central or local laboratory results must be used to confirm eligibility. The screening labs used to determine eligibility should be collected within 14 days prior to cycle 1 day 1. In case of multiple laboratory data within this period, the most recent central laboratory data should be used. Additional assessments may be done centrally or locally to monitor AEs or as required by dose modification requirements.

Laboratory results obtained during screening should be used to determine eligibility criteria. In situations where laboratory results are outside of the permitted range, the investigator may opt to retest the subject and subsequent within-range screening results may be used to confirm eligibility. If retesting of lab values is necessary to confirm eligibility, local labs can be used without requiring additional sample collection for central laboratory submission. CT scans and MRI conducted as part of a subject's routine clinical management (i.e., standard of care) obtained before signing the ICF may be utilized for screening or baseline purposes, provided the procedures met the protocol-specified criteria and were performed within the Screening period.

Laboratory test results will be reviewed by the investigator prior to any study treatment. Local laboratory results may be used for treatment decisions; however, central laboratory samples must also be drawn per the protocol and sent to the central laboratory, unless otherwise approved by the sponsor. Central and local labs may be collected up to 48 hours prior to study treatment. Additional laboratory tests should be performed according to institutional standard of care. Clinical significance of out-of-range laboratory findings is to be determined and documented by the investigator/subinvestigator who is a qualified physician.

5.4.4 Physical Examination

Physical examinations will be conducted at visits as outlined in the Schedules of Assessments [Table 1, Table 3, Table 4, Table 7, Table 9 and Table 11]. Each physical examination will include the observation and review of all body systems and weight; height is only required at screening. A full physical exam is required at screening. The physical exam only needs to be repeated on C1D1 if clinically significant changes from screening are observed. Subsequently, targeted (symptom-driven) physical exams should be conducted every 2 or 3 weeks on zolbetuximab visit days. If clinically significant worsening of findings from baseline is noted at any study visit, the changes will be documented as AEs on the AE eCRF. Clinical significance is defined as any variation in physical findings, which has medical relevance that could result in an alteration in medical care. The investigator will continue to monitor the subject until the parameter returns to Grade ≤ 1, or to the baseline condition, or until the investigator determines that follow-up is no longer medically necessary.

5.4.5 Electrocardiogram

A single 12-lead ECG will be performed at screening, at the zolbetuximab study drug discontinuation visit, zolbetuximab 30-day follow-up visit (Cohort 5 only: and preoperative follow-up visits [7-14 days and 30 days after last preoperative dose] and postoperative follow-up visits [EOT and 30-day follow-up]) and if clinically indicated. A single 12-lead

ECG will also be performed and assessed locally prior to every oxaliplatin infusion (before any antiemetic treatment) and following completion of every oxaliplatin infusion. Triplicate ECG will be collected in Cohort 1A at the time points outlined in the Schedules of Assessments [Table 2] to evaluate the potential of zolbetuximab to induce QT/corrected QT (QTc) interval prolongation. Prior to performing triplicate ECG, subjects should rest in supine position for 10 minutes and their ECGs should be recorded in triplicate with 2 minutes apart per time point. When collected on the same day, ECG should be collected prior to pharmacokinetic samples. Additional ECG may be performed based on medical history and investigator medical judgment. All ECGs for Cohorts 1A and 2 will be read centrally except for ECGs performed prior to and following completion of every oxaliplatin infusion, in which case a local read will be acceptable. All ECGs for Cohorts 3A, 4 and 5 will require a local read.

5.5 Adverse Events and Other Safety Aspects

5.5.1 Definition of Adverse Events

An AE is any untoward medical occurrence in a subject, temporally associated with the use of a medicinal product, whether or not considered related to the medicinal product. An AE can therefore be any unfavorable and unintended sign (including an abnormal laboratory finding), symptom or disease (new or exacerbated) temporally associated with the use of a medicinal product.

In order to identify any events that may be associated with study procedures and could lead to a change in the conduct of the study, Astellas collects AEs even if the subject has not received study drug treatment. AE collection begins after the signing of the main informed consent and will be collected until 90 days after the last dose of study drug.

Some countries may have additional local requirements for events that are required to be reported as AEs or in an expedited manner similar to an SAE. In these cases, it is the investigator's responsibility to ensure that these AEs or other reporting requirements are followed and the information is appropriately recorded in the source data accordingly.

5.5.1.1 Abnormal Laboratory Findings

Any abnormal laboratory test result (e.g., hematology, clinical chemistry or urinalysis) or other safety assessment (e.g., ECGs, radiographic scans, vital signs measurements, physical examination), including those that worsen from baseline, which is considered to be clinically significant in the medical and scientific judgment of the investigator and not related to underlying disease, is to be reported as an (S)AE.

Any clinically significant abnormal laboratory finding or other abnormal safety assessment, which is associated with the underlying disease does not require reporting as an (S)AE, unless judged by the investigator to be more severe than expected for the subject's condition.

Repeating an abnormal laboratory test or other safety assessment, in the absence of any of the above criteria, does not constitute an AE. Any abnormal test result that is determined to be an error does not require reporting as an AE.

5.5.1.2 Potential Cases of Drug-induced Liver Injury

Refer to [Appendix 12.2 Liver Safety Monitoring and Assessment] for detailed instructions on Drug Induced Liver Injury (DILI). Abnormal values in AST and/or ALT concurrent or with abnormal elevations in total bilirubin that meet the criteria outlined in [Appendix 12.2 Liver Safety Monitoring and Assessment], in the absence of other causes of liver injury, are considered potential cases of DILI (potential Hy's Law cases) and are always to be considered important medical events and reported per [Section 5.5.5 Reporting of Serious Adverse Events].

5.5.1.3 Disease Progression and Study Endpoints

Under this protocol, the following event(s) will not be considered as an(S)AE:

- Disease Progression: events including defined study endpoints that are clearly consistent with the expected pattern of progression of the underlying disease are not to be recorded as AEs. These data will be captured as efficacy assessment data as outlined in [Section 5.3 Efficacy Assessments]. If there is any uncertainty as to whether an event is due to anticipated disease progression and/or if there is evidence suggesting a causal relationship between the study drug and the event, it should be reported as an (S)AE. All deaths up to 90 days after the last dose of study drug must be reported as an SAE, even if attributed to disease progression.
- Pre-planned and elective hospitalizations or procedures for diagnostic, therapeutic or surgical procedures for a pre-existing condition that did not worsen during the course of the clinical trial. These procedures are collected per the eCRFs Completion Guidelines.

Disease progression can be considered as the worsening of a subject's condition attributable to gastric cancer. It may be an increase in the severity of the disease under study and/or increases in the symptoms of the disease. The development of new, or progression of existing metastases to the primary cancer under study should be considered as disease progression not an AE. Events which are unequivocally due to disease progression should not be reported as an AE during the study.

5.5.2 Definition of Serious Adverse Events

An AE is considered "serious" if, in the view of either the investigator or sponsor, it results in any of the following outcomes:

- Results in death
- Is life-threatening (an AE is considered "life-threatening" if, in the view of either the investigator or sponsor, its occurrence places the subject at immediate risk of death. It does not include an AE that, had it occurred in a more severe form, might have caused death)
- Results in persistent or significant disability/incapacity or substantial disruption of the ability to conduct normal life functions
- Results in congenital anomaly, or birth defect

- Requires inpatient hospitalization (except for planned procedures as allowed per study, e.g., surgery for subjects in Cohort 5) or leads to prolongation of hospitalization (except if prolongation of planned hospitalization is not caused by an AE). Hospitalization for treatment/observation/examination caused by AE is to be considered as serious.)
- Other medically important events (defined in paragraph below)

Medical and scientific judgment should be exercised in deciding whether expedited reporting is appropriate in other situations, such as important medical events that may not be immediately life-threatening or result in death or hospitalization, but may jeopardize the subject or may require intervention to prevent 1 of the other outcomes listed in the definition above. These events, including those that may result in disability/incapacity, usually are considered serious. Examples of such events are intensive treatment in an emergency room or at home for allergic bronchospasm; blood dyscrasias or convulsions that do not result in hospitalization; or development of drug dependency or drug abuse.

If an AE occurs that the sponsor determines to be an Important Medical Event, additional information on the event (e.g., investigator confirmation of seriousness, causality) will be requested.

Progression of gastric cancer, including signs and symptoms of progression, should not be reported as an SAE unless it results in death within 90 days of the last dose of study drug. For progression-related death reported as an SAE, there should be available immediate cause of death reported as the event term. “Death due to disease progression” should be recorded as the AE term only when the cause of death cannot be otherwise determined.

5.5.3 Criteria for Causal Relationship to Study Drug

The investigator is obligated to assess the relationship between the study drug and each occurrence of each (S)AE. The medically qualified investigator will use medical judgment as well as the RSI [Section 1.3] to determine the relationship. The causality assessment is one of the criteria used when determining regulatory reporting requirements.

The medically qualified investigator is requested to provide an explanation for the causality assessment for each SAE and must document in the medical notes that he/she has reviewed the (S)AE and has provided an assessment of causality. The causality assessment is one of the criteria used when determining regulatory reporting requirements. The investigator may revise his/her assessment of causality in light of new information regarding the SAE and shall send an SAE follow-up report and update the eCRF with the new information and updated causality assessment.

Following a review of the relevant data, the causal relationship between the study drug and each (S)AE will be assessed by answering ‘**yes**’ or ‘**no**’ to the question “**Do you consider that there is a reasonable possibility that the event may have been caused by the study drug**”.

When making an assessment of causality, the following factors are to be considered when deciding if there is evidence and/or arguments to suggest there is a ‘reasonable possibility’

that an (S)AE may have been caused by the study drug (rather than a relationship cannot be ruled out) or if there is evidence to reasonably deny a causal relationship:

- Plausible temporal relationship between exposure to the study drug and (S)AE onset and/or resolution. Has the subject actually received the study drug? Did the (S)AE occur in a reasonable temporal relationship to the administration of the study drug?
- Plausibility; i.e., could the event have been caused by the study drug? Consider biologic and/or pharmacologic mechanism, half-life, literature evidence, drug class, preclinical and clinical study data, etc.
- Dechallenge/Dose reduction/Rechallenge:
  - Did the (S)AE resolve or improve after stopping or reducing the dose of the suspect drug? Also consider the impact of treatment for the event when evaluating a dechallenge experience.
  - Did the (S)AE reoccur if the suspected drug was reintroduced after having been stopped?
- Laboratory or other test results; a specific laboratory investigation supports the assessment of the relationship between the (S)AE and the study drug (e.g., based on values pre-, during and post-treatment)
- Available alternative explanations independent of study drug exposure; such as other concomitant drugs, past medical history, concurrent or underlying disease, risk factors including medical and family history, season, location, etc. and strength of the alternative explanation

There may be situations in which an SAE has occurred and the investigator has minimal information to include in the initial report to the sponsor. However, it is very important that the investigator always make an assessment of causality for every event before the initial transmission of the SAE data to the sponsor. With limited or insufficient information about the event to make an informed judgment and in absence of any indication or evidence to establish a causal relationship, a causality assessment of ‘no’ is to be considered. In such instance, the investigator is expected to obtain additional information regarding the event as soon as possible and to re-evaluate the causality upon receipt of additional information.

5.5.4 Criteria for Defining the Severity of an Adverse Event

AEs, including abnormal clinical laboratory values, will be graded using the NCI-CTCAE guidelines (Version 4.03). The items that are not stipulated in the NCI-CTCAE Version 4.03 will be assessed according to the criteria below and entered into the eCRF.

Grade	Assessment Standard
1-Mild	Asymptomatic or mild symptoms, clinical or diagnostic observations noted; intervention not indicated.
2-Moderate	Local or noninvasive intervention indicated.
3-Severe	Medically significant but not immediately life threatening, hospitalization or prolonged hospitalization.
4-Life Threatening	Life threatening consequences, urgent intervention indicated
5-Death	Death related to AE

AE: adverse event

5.5.5 Reporting of Serious Adverse Events

The collection of AEs and the expedited reporting of SAEs will start following receipt of the main informed consent and will continue to until 90 days after the last dose of study drug.

In the case of a SAE, the investigator must contact the sponsor by fax or email immediately (within 24 hours of awareness).

The investigator must complete and submit a SAE worksheet containing all information that is required by local and/or regional regulations to the sponsor by email or fax immediately (within 24 hours of awareness).

Specific to Japan sites: In the case of a SAE, the investigator or subinvestigator must report to the head of the study site and must contact the sponsor by fax or email immediately (within 24 hours of awareness). The investigator should complete and submit JUTOKUNA YUUGAIJISHOU HOUKOKUSHO containing all information that is required by the appropriate regulatory authorities to the sponsor by fax or email immediately (within 24 hours of awareness) and to the head of the hospital.

The SAE worksheet must be signed by a medically qualified investigator (as identified on the Delegation of Authority Log). Signature confirms accuracy and completeness of the SAE data as well as the investigator causality assessment including the explanation for the causality assessment.

For contact details, see [Section II Contact Details of Key Sponsor's Personnel]. Fax or email the SAE/Special Situations Worksheet to:

Astellas Pharma Global Development – United States
Pharmacovigilance
North American Fax: 888-396-3750
(North America Alternate Fax: 847-317-1241)
International Fax: +44-800-471-5263
Email: safety-us@astellas.com

Specific to Japan sites: For contact details, see [Contact Details of Sponsor's Key Personnel]. Fax or email the JUTOKUNA YUUGAIJISHOU HOUKOKUSHO special situations worksheet to:

Astellas Pharma Inc. – Japan
Pharmacovigilance
Fax number +81-(0)3-3243-5747
Email: rk-safety-jp@astellas.com

If there are any questions or if clarification is needed regarding the SAE, please contact the sponsor's Medical Monitor/Study Physician or his/her designee [Section II Contact Details of Key Sponsor’s Personnel].

Follow-up information for the event should be sent promptly (within 7 days of the initial notification. ***Specific to Japan sites***: within 2 days for the initial notification).

Full details of the SAE should be recorded on the medical records, SAE/Special Situation Worksheet and on the (e)CRF.

The following minimum information is required:

- International Study Number (ISN)/study number,
- Subject number, sex and age,
- The date of report,
- A description of the SAE (event, seriousness criteria),
- Causal relationship to the study drug (including reason) and
- The drug provided (if any)

The sponsor or sponsor's designee will medically evaluate the SAE and determine if the report meets the requirements for expedited reporting based on seriousness, causality, and expectedness of the events (e.g., Suspected Unexpected Serious Adverse Reaction (SUSAR) reporting) according to current local/regional regulatory requirements in participating countries. The sponsor or sponsor’s designee will submit expedited safety reports (e.g., IND Safety Reports, Council for International Organizations of Medical Sciences Form 1 [CIOMS-I]) to Competent Authorities and concerned Ethics Committee per current local regulations, and will inform the investigators of such regulatory reports as required. Investigators must submit safety reports as required by their IRB/local IEC within timelines set by regional regulations (e.g., EU, electronic Common Technical Document, FDA) where required. Documentation of the submission to and receipt by the IRB/ local IEC of expedited safety reports should be retained by the site.

The sponsor will notify all investigators responsible for ongoing clinical studies with the study drug of all SUSARs which require submission per local requirements to the IRB, local IEC or head of the study site.

The investigators should provide written documentation of IRB/IEC notification for each report to the sponsor.

The investigator may contact the sponsor's Medical Monitor/Study Physician for any other problem related to the safety, welfare or rights of the subject.

5.5.6 Follow-up of Adverse Events

All AEs occurring during or after the subject has discontinued the study are to be followed up until resolved or judged to be no longer clinically significant, or until they become chronic to the extent that they can be fully characterized by the investigator.

If after the protocol defined AE collection period [see Section 5.5.1 Definition of Adverse Event], an AE progresses to a SAE or the investigator learns of any (S)AE including death, where he/she considers there is reasonable possibility it is related to the study drug treatment or study participation, the investigator must promptly notify the sponsor.

5.5.7 Monitoring of Common Serious Adverse Events

Common SAEs are SAEs commonly anticipated to occur in the study population independent of drug exposure. SAEs classified as “common” are provided in [Appendix 12.3 Common Serious Adverse Events] for reference. The list does NOT change the investigator’s reporting obligations, nor his obligations to perform a causality assessment, or prevent the need to report an AE meeting the definition of an SAE as detailed above. The purpose of this list is to alert the investigator that some events reported as SAEs may not require expedited reporting to the regulatory authorities based on the classification of “common SAEs” as specified in [Appendix 12.3 Common Serious Adverse Events]. The sponsor will monitor these events throughout the course of the study for any change in frequency. Any changes to this list will be communicated to the participating investigational sites. Investigators must report individual occurrences of these events as stated in [Section 5.5.5 Reporting of Serious Adverse Events].

5.5.8 Adverse Events of Special Interest

In case of zolbetuximab induced nausea, vomiting or hypersensitivity, infusion rate of zolbetuximab may be reduced or infusion paused or discontinued based on investigator’s clinical judgment about severity of toxicity and local standard of care (see [Section 5.1.3.1 Zolbetuximab]).

If the AEs of interest are classified as serious, they are to be collected via the SAE/Special Situation worksheet and reported within 24 hours as described in [Section 5.5.5 Reporting of Serious Adverse Events].

5.5.8.1 Guidelines for Infusion-related Reactions

Subjects should be closely monitored for infusion-related reactions to facilitate early identification and management, as per the guidelines in Table 16. The management of such toxicities should be based on institutional standard of care and published guidelines, as appropriate based on investigator judgment and on the protocol instructions regarding interruption or discontinuation of study treatment.

A subject with an infusion reaction should be evaluated specifically for the symptoms and signs that are highly suggestive of anaphylaxis (urticaria, repetitive cough, wheeze and throat tightness/change in voice). A careful examination of the skin is advised in order to detect urticaria, which often appears first in the neck, trunk, abdomen and axillae.

Not all anaphylactic reactions manifest as anaphylactic shock. Because anaphylaxis can recur and worsen with re-exposure, it is recommended that any subject having a reaction with features (even if mild) that are highly suggestive of anaphylaxis not be re-exposed to the causative agent until an allergy specialist has evaluated the subject.

5.5.9 Special Situations

Certain Special Situations observed in association with the study drug(s), such as incorrect administration (e.g., wrong dose of study drug, comparator or background therapy) are collected in the eCRF, as Protocol Deviation per [Section 8.1.6 Major Protocol Deviations] or may require special reporting, as described below. These Special Situations are not considered AEs, but do require to be communicated to Astellas as per the timelines defined below.

If a Special Situation is associated with, or results in, an AE, the AE is to be assessed separately from the Special Situation and captured as an AE in the eCRF or electronic data source. If the AE meets the definition of a SAE, the SAE is to be reported as described in [Section 5.5.5 Reporting of Serious Adverse Events] and the details of the associated Special Situation are to be included in the clinical description on the SAE worksheet.

The Special Situations are:

- Pregnancy
- Medication error, overdose and use outside protocol
- Misuse/abuse
- Occupational exposure
- (Suspicion of) Transmission of infectious agent
- Suspected drug-drug interaction

5.5.9.1 Pregnancy

If a female subject becomes pregnant during the study dosing period, within 9 months from the discontinuation of oxaliplatin or within 6 months of discontinuation of other study drugs, the investigator is to report the information to the sponsor. If a partner of a male subject becomes pregnant within the study period or within 6 months from the discontinuation of dosing, the investigator is to report the information to the sponsor. For reporting, the investigator is to report the information to the sponsor according to the timelines in [Section 5.5.5 Reporting of Serious Adverse Events] using the Pregnancy Reporting Form and in the eCRF.

The expected date of delivery or expected date of the end of the pregnancy, last menstruation, estimated conception date, pregnancy result and neonatal data etc., should be included in this information.

While pregnancy itself is not considered to be an AE or SAE, any pregnancy complication or termination (including elective termination) of a pregnancy is to be reported for a female study subject as an AE in the eCRF or SAE per [Section 5.5.5 Reporting of Serious Adverse Events]. For (S)AEs experienced by a female partner of a male subject, (S)AEs are to be reported via the Pregnancy Reporting Form.

Additional information regarding the outcome of a pregnancy when also categorized as an SAE is mentioned below:

- "Spontaneous abortion" includes miscarriage, abortion and missed abortion.
- Death of a newborn or infant within 1 month after birth is to be reported as an SAE regardless of its relationship with the study drug.
- If an infant dies more than 1 month after the birth, is to be reported if a relationship between the death and intrauterine exposure to the study drug is judged as "possible" by the investigator.
- Congenital anomaly (including anomaly in miscarried fetus)

Unless a congenital anomaly is identified prior to spontaneous abortion or miscarriage, the embryo or fetus should be assessed for congenital defects by visual examination. (S)AEs experienced by the newborn/infant should be reported via the Pregnancy Reporting Form. Generally, follow-up will be no longer than 6 to 8 weeks following the estimated delivery date.

5.5.9.2 Medication Error, Overdose and “Off-label Use”

If a Medication Error, Overdose or “Off- label Use” (i.e., use outside of what is stated in the protocol) is suspected, refer to [Section 8.1.6 Major Protocol Deviations]. Any associated (S)AEs are to be reported in the eCRF. If the AE meets the definition of a SAE, the SAE is also to be reported as described in [Section 5.5.5 Reporting of Serious Adverse Events] together with the details of the medication error, overdose or “Off-Label Use”.

There is no antidote for overdose of the study drug. In the event of suspected zolbetuximab overdose, the subject should receive supportive care and monitoring. The Medical Monitor should be contacted as applicable.

In the event of mFOLFOX6, pembrolizumab, nivolumab or FLOT overdose, refer to the approved Package Insert, SPC or local prescribing information supplied by the manufacturer for each agent.

5.5.9.3 Misuse/Abuse

If misuse or abuse of the study drug(s) is suspected, the investigator must forward the Special Situation worksheet to the sponsor by fax or email immediately (within 24 hours of awareness). Any associated (S)AEs are to be reported in the eCRF. If the AE meets the definition of a SAE, the SAE is also to be reported as described in [Section 5.5.5 Reporting of Serious Adverse Events] together with details of the misuse or abuse of the study drug(s).

5.5.9.4 Occupational Exposure

If occupational exposure (e.g., inadvertent exposure to the study drug(s) of site staff whilst preparing it for administration to the subject) to the study drug(s) occurs, the investigator must forward the Special Situation worksheet to the sponsor by fax or email immediately (within 24 hours of awareness). Any associated (S)AEs occurring to the individual associated with or resulting from the Special Situation are to be reported on the Special Situations worksheet.

5.5.9.5 (Suspicion of) Transmission of Infectious Agent

If transmission of an infectious agent associated with the study drug(s) is suspected, the investigator must forward the Special Situation worksheet to (the sponsor/delegated contract research organization [CRO]) by fax or email immediately (within 24 hours of awareness) and any associated (S)AEs are to be reported in the eCRF. If the AE meets the definition of a SAE, the SAE is also to be reported as described in [Section 5.5.5 Reporting of Serious Adverse Events (SAEs)] together with the details of the suspected transmission of infectious agent.

5.5.9.6 Suspected Drug-Drug Interaction

If a suspected drug-drug interaction associated with the study drug(s) is suspected, the investigator must forward the Special Situation worksheet to the sponsor by fax or email immediately (within 24 hours of awareness). Any associated (S)AEs are to be reported in the eCRF. If the AE meets the definition of a SAE, the SAE is also to be reported as described in [Section 5.5.5 Reporting of Serious Adverse Events] together with details of the suspected drug-drug interaction.

5.5.10 Supply of New Information Affecting the Conduct of the Study

When new information becomes available necessary for conducting the clinical study properly, the sponsor will inform all investigators involved in the clinical study as well as the regulatory authorities. Investigators should inform the IRB/IEC of such information when needed.

The investigator will also inform the subjects, who will be required to sign an updated informed consent form (ICF) in order to continue in the clinical study.

Specific to Japan sites:

1. When information is obtained regarding serious and unexpected adverse drug reactions (or other) that are specified in Article 273 of the Act on Securing Quality, Efficacy and Safety of Pharmaceuticals, Medical Devices, Regenerative and Cellular Therapy Products, Gene Therapy Products, and Cosmetics, in compliance with Article 80-2 Paragraph 6 of the Pharmaceutical Affairs Law, the sponsor should inform all investigators involved in the study, head of the study site and appropriate regulatory authorities of such information. The head of the study site who receives such information will decide whether the study should be continued after hearing the opinions of the IRB. The investigator will supply the new information to the

participants, in compliance with [Section 8.2.3.2 Supply of New and Important Information Influencing the Subject's Consent and Revision of the Written Information].

2. In addition, when the head of the study site receives the revisions of the IB, protocol, written information, information on the matters covering the quality of the test product, efficacy and safety, information necessary for conducting the study properly or documents to be examined by the IRB, these documents should be sent to the IRB.

5.5.11 Urgent Safety Measures

An Urgent Safety Measure (USM) is an intervention, which is not defined by the protocol and can be put in place with immediate effect without needing to gain prior approval by the sponsor, relevant Competent Authorities, IRB/IEC, where applicable, in order to protect study participants from any immediate hazard to their health and/or safety. Either the investigator or the sponsor can initiate an USM. The cause of an USM can be safety, product or procedure related.

5.5.12 Reporting Urgent Safety Measures

In the event of a potential USM, the investigator must contact the Astellas Study Physician (***Specific to Japan sites:*** an Astellas team member) (within 24 hrs of awareness). Full details of the potential USM are to be recorded in the subject's medical records. The sponsor may request additional information related to the event to support their evaluation.

If the event is confirmed to be an USM, the sponsor will take appropriate action to ensure the safety and welfare of the subjects. These actions may include but are not limited to a change in study procedures or study treatment, halting further enrollment in the trial, or stopping the study in its entirety. The sponsor or sponsor's designee will notify Competent Authority and central Ethics Committee within the timelines required per current local regulations, and will inform the investigators as required. When required, investigators must notify their IRB/IEC within timelines set by regional regulations.

5.6 Test Drug Concentration

Serum concentrations of zolbetuximab, pembrolizumab and nivolumab, and plasma concentrations of oxaliplatin (measured as total and free platinum) and 5-FU, will be measured. Samples will be collected as outlined in the Schedule of Assessments. Blood sampling, processing, storage and shipment instructions will be provided in the laboratory manual. Samples will be shipped to and analyzed by a sponsor-designated analytical laboratory using validated analytical methods. Samples remaining after pharmacokinetic assessments may be used for additional biomarker analysis as described in [Section 5.7 Other Measurements, Assessments or Methods]. Please refer to the laboratory manual for more detailed information.

5.7 Other Measurements, Assessments or Methods

5.7.1 Biomarkers

Tumor tissue and blood/serum/plasma samples [described in Sections 5.7.2 Blood/Serum/Plasma Samples/PBMCs for Exploratory Biomarkers and 5.7.3 Tumor Tissue Samples] may be collected and used for research purposes as allowed per local policy to identify genomic and/or other biomarkers that may be associated with clinical outcome or dynamic changes associated with any study drug treatment (in terms of dose, safety, tolerability and efficacy). Since the identification of exploratory biomarkers that correlate with the efficacy or safety of zolbetuximab treatment may continue to evolve as new findings becomes available, additional analyses related to zolbetuximab activity on tumor signaling pathways or clinical outcomes may be conducted. Tumor tissue and blood/serum samples remaining after the specified biomarker assessments (e.g., aliquots of tumor cell RNA or DNA, peripheral blood mononuclear cells [PBMCs]) may be used for re-testing, additional analyses as defined above or developing, and validating assays related to prediction of response or dynamic changes associated with zolbetuximab treatment. The tumor tissue and blood/serum/plasma samples (e.g., aliquots of tumor cell RNA or DNA, PBMCs) will be stored at the study sponsors' facility or a contract laboratory facility for up to 15 years after database closure, at which time the samples will be destroyed. The procedures for the collection, handling and shipping of laboratory samples being submitted to the central laboratory will be specified in a laboratory manual.

5.7.2 Blood/Serum/Plasma Samples/PBMCs for Exploratory Biomarkers

Blood, serum, plasma and PBMC samples will be collected according to the Schedule of Assessments for exploratory biomarker measurements. Serum and plasma samples may be analyzed for biomarkers including but not limited to chemokines, cytokines, CDC activation, circulating DNA and soluble factors. Blood samples may be analyzed for biomarkers including but not limited to lymphocyte subsets, genetic markers and ADCC activity in PBMCs. If the study is terminated for reasons other than safety, and subjects who continue to receive benefit are allowed to continue receiving treatment, additional biomarker samples may not be collected.

5.7.3 Tumor Tissue Samples

Tissue samples may be analyzed for biomarkers including but not limited to CLDN18.2, HER2, PD-L1, and immune cell infiltration. In addition, tissue samples may be tested for other biomarkers including genetic biomarkers, proteins, other large molecules and small molecules, to further investigate how the study treatment works. Tumor sample collection is summarized below.

Tissue Requirements for All Cohorts

The investigator, in consultation with other specialists, as needed (e.g., radiology staff) will assess the risk associated with obtaining a tumor tissue sample and determine if the subject is an appropriate candidate for the procedure. Biopsies should be obtained in accordance with institutional policies/guidelines to minimize risk. Procedures requiring general anesthesia

should not be performed to obtain a tumor tissue sample; however, if a surgical procedure under general anesthesia is performed for a clinical indication, excess tumor tissue may be used for research purposes with the consent of the subject.

Table 31 Tumor Tissue Requirements Cohort 1

Sample	Cohort	Visit	Tumor Tissue Requirement
Tumor Sample for Eligibility	1A (required)	Prescreening or Screening (if biopsy required)	Tumor Sample for CLDN18.2: Archival tumor tissue is preferred but if the specimen is insufficient or unavailable, the subject must enter the screening period and undergo a biopsy to obtain the tumor sample for eligibility. A minimum of 1 FFPE tumor tissue block (preferred) OR a minimum of 9 FFPE unstained sections are required.
Baseline Tumor Sample	1A (required)	Screening	Subjects are required to provide a tumor specimen collected within 3 months prior to the first dose of study treatment. If the specimen is insufficient or unavailable, a biopsy may be performed to obtain tumor tissue. Ensure that subject meets all other study eligibility criteria prior to performing the biopsy (as applicable). Tissue slides or blocks submitted as the baseline tumor sample must be a separate submission than the tumor sample for eligibility. A minimum of 1 FFPE tumor tissue block (preferred) OR a minimum of 15 FFPE unstained sections are required
On-Treatment Biopsy	1A (required)	cycle 3 day 1	Subjects are required to provide an on-treatment tumor specimen collected ± 15 days of the cycle 3 day 1 visit. A minimum of 1 FFPE tumor tissue block (preferred) OR a minimum of 15 FFPE unstained sections are required.
Post-Progression Biopsy	1A (optional)	Post-Progression	Optional post-progression tumor tissue sample for exploratory biomarker analysis may be collected following disease progression for subjects who sign a separate ICF. A minimum 1 FFPE tumor tissue block (preferred) OR a minimum of 15 FFPE unstained sections are required

FFPE: formalin-fixed paraffin-embedded

Table 32 Tumor Tissue Requirements Cohort 2

Sample	Cohort	Visit	Tumor Tissue Requirement
Tumor Sample for Eligibility	2 (required)	Prescreening or Screening (if biopsy required)	Tumor Sample for CLDN18.2 and HER2 (if required): Archival tumor tissue is preferred, but if the specimen is insufficient or unavailable, the subject must enter the screening period and undergo a biopsy to obtain the tumor sample for eligibility. A minimum of 1 FFPE tumor tissue block (preferred) OR a minimum of 15 FFPE unstained sections are required.<br>*If local HER2 results are available, a minimum of 9 slides are required along with the pathology report. If local HER2 results are unavailable, follow guidance above.
Baseline Tumor Sample	2 (required)	Screening	Subjects are required to provide a tumor specimen collected within 3 months prior to the first dose of study treatment. If the specimen is insufficient or unavailable, a biopsy may be performed to obtain tumor tissue. Ensure that subject meets all other study eligibility criteria prior to performing the biopsy (as applicable). Tissue slides or blocks submitted as the baseline tumor sample must be a separate submission than the tumor sample for eligibility. A minimum of 1 FFPE tumor tissue block (preferred) OR a minimum of 15 FFPE unstained sections are required.
On-Treatment Biopsy	2 (required)	cycle 2 day 1	Subjects are required to provide an on-treatment tumor specimen collected at the cycle 2 day 1 visit (± 15 days). A minimum of 1 FFPE tumor tissue block (preferred) OR a minimum of 15 FFPE unstained sections are required.
Post-Progression Biopsy	2 (optional)	Post-Progression	Optional post-progression tumor tissue sample for exploratory biomarker analysis may be collected following disease progression for subjects who sign a separate ICF. A minimum 1 FFPE tumor tissue block (preferred) OR a minimum of 15 FFPE unstained sections are required.

FFPE: formalin-fixed paraffin-embedded; HER2: human epidermal growth factor receptor 2; ICF: informed consent form

Table 33 Tumor Tissue Requirements Cohort 3

Sample	Cohort	Visit	Tumor Tissue Requirement
Tumor Sample for Eligibility	3A (required)	Prescreening or Screening (if biopsy required)	Tumor sample for CLDN18.2: Archival tumor tissue is preferred. A minimum of 1 FFPE tumor tissue block (preferred) OR a minimum of 9 FFPE unstained sections are required. If archival tumor tissue is insufficient or unavailable for pre-screening, the subject will enter the screening period and a biopsy will be performed to obtain the tumor sample for eligibility.
Baseline Tumor Sample	3A (optional)	Screening	Subjects are required to provide a tumor specimen collected within 3 months prior to the first dose of study treatment. If the specimen is insufficient or unavailable, a biopsy may be performed to obtain tumor tissue. Ensure that subject meets all other study eligibility criteria prior to performing the biopsy (as applicable). Tissue slides or blocks submitted as the baseline tumor sample must be a separate submission than the tumor sample for eligibility. A minimum of 1 FFPE tumor tissue block (preferred) OR a minimum of 15 FFPE unstained sections are required.
On-Treatment Biopsy	3A (optional)	cycle 3 day 1	Subjects are required to provide an on-treatment tumor specimen collected ± 15 days of the C3D1 visit. A minimum of 1 FFPE tumor tissue block (preferred) OR a minimum of 15 FFPE unstained sections are required.
Post-Progression Biopsy	3A (optional)	Post-Progression	Optional post-progression tumor tissue sample for exploratory biomarker analysis may be collected following disease progression for subjects who sign a separate ICF. A minimum 1 FFPE tumor tissue block (preferred) OR a minimum of 15 FFPE unstained sections are required.

FFPE: formalin-fixed paraffin-embedded; HER2: human epidermal growth factor receptor 2; ICF: informed consent form

Table 34 Tumor Tissue Requirements Cohort 4

Sample	Cohort	Visit	Tumor Tissue Requirement
Tumor Sample for Eligibility	4A and 4B (required)	Prescreening or Screening (if biopsy required)	FFPE tumor tissue will be collected for central testing to determine CLDN18.2 and HER2 status (if required). Archival tumor tissue from the primary tumor (gastric or GEJ) is preferred. If primary tumor tissue is not available, tumor tissue from a metastatic site (excluding bone metastasis) may be used. A minimum of 1 FFPE tumor tissue block (preferred) OR a minimum of 15 FFPE unstained slides are required, as allowed per local policy. If slides are submitted, the slides should be freshly cut from the FFPE block within the time frame described in the laboratory manual. If HER2 results are already available from local testing, a minimum of 12 FFPE unstained slides are required to be submitted to the central lab. If the specimen is insufficient or unavailable, the subject will enter the screening period and a biopsy will be performed to obtain primary tumor tissue (preferred) or tumor tissue from metastatic site (excluding bone metastasis). Sponsor pre-approval is required when the sole purpose of the biopsy procedure is to assess eligibility for this study.
Baseline Tumor Sample	4A (optional) and 4B (required)	Screening	Subjects are required to provide a tumor specimen collected within 3 months prior to the first dose of study treatment. If the specimen is insufficient or unavailable, a biopsy may be performed to obtain tumor tissue. Ensure that subject meets all other study eligibility criteria prior to performing the biopsy (as applicable). Tissue slides or blocks submitted as the baseline tumor sample must be a separate submission than the tumor sample for eligibility. A minimum of 1 FFPE tumor tissue block (preferred) OR a minimum of 15 FFPE unstained sections are required. This is optional for Cohort 4A.
On-Treatment Biopsy	4A (optional) 4B (required)	cycle 2 day 1	Subjects are required to provide an on-treatment tumor specimen collected ± 15 days of the C2D1 visit. A minimum of 1 FFPE tumor tissue block (preferred) OR a minimum of 15 FFPE unstained sections are required. This is optional for Cohort 4A. If ≥ 15 slides cannot be provided, the sponsor should be contacted for further guidance.
Post-Progression Biopsy	4A and 4B (optional)	Zolbetuximab discontinuation visit	For subjects who signed a separate ICF, an optional post-progression tumor sample for exploratory biomarker analysis should be collected following confirmation of radiographic disease progression and prior to commencement of subsequent anti-cancer therapy. A minimum 1 FFPE tumor tissue block (preferred) OR a minimum of 15 FFPE unstained sections are required.

C2D1: cycle 2 day 1; FFPE: formalin-fixed paraffin-embedded; HER2: human epidermal growth factor receptor 2; ICF: informed consent form

Table 35 Tumor Tissue Requirements Cohort 5

Sample	Cohort	Visit	Tumor Tissue Requirement
Tumor Sample for Eligibility	5 (required)	Prescreening	FFPE tumor tissue will be collected for central testing to determine CLDN18.2 status. Archival tumor tissue sample is preferred. A minimum of 1 FFPE tumor tissue block (preferred) OR a minimum of 10 FFPE unstained slides are required, as allowed per local policy. If slides are submitted, the slides should be freshly cut from the FFPE block within the time frame described in the laboratory manual.
Post-operative (Surgical) Sample	5 (required)	Operative (surgical) sample	Subjects are required to provide the postoperative (surgical) sample. A minimum of 1 FFPE tumor tissue block (preferred) OR a minimum of 15 FFPE unstained sections are required.
Post-progression Biopsy	5 (optional)	Zolbetuximab discontinuation visit	For subjects who signed a separate ICF, an optional post-progression tumor sample for exploratory biomarker analysis should be collected following confirmation of radiographic disease progression and prior to commencement of subsequent anti-cancer therapy. A minimum of 1 FFPE tumor tissue block (preferred) OR a minimum of 15 FFPE unstained sections are required.

FFPE: formalin-fixed paraffin-embedded; ICF: informed consent form

FFPE eligibility tumor tissue samples will be obtained for all subjects and sent for central IHC testing during the pre-screening assessment in order to evaluate for CLDN18.2, and HER2 status if a previously documented HER2 test result is not available (Cohorts 2 and 4 only).

If a tumor biopsy is to be obtained during screening from a lesion that will be classified as 1 of the target lesions, the biopsy should be performed prior to obtaining the baseline scan, if possible. Otherwise, a new baseline scan should be obtained subsequent to the biopsy of the target lesion.

5.7.4 Immunogenicity Assessment

The formation of ADAs against zolbetuximab will be assessed. Serum samples will be collected as outlined in the Schedule of Assessments. Blood sampling, processing, storage and shipment instructions will be provided in the laboratory manual. Samples will be shipped to and analyzed by a sponsor designated analytical laboratory using validated analytical methods. Samples remaining after zolbetuximab immunogenicity assessments may be used for pembrolizumab or nivolumab immunogenicity analysis if deemed appropriate, or for additional biomarker analysis as described in [Section 5.7 Other Measurements, Assessments or Methods]. Please refer to the laboratory manual for more detailed information.

5.7.5 Optional Samples for Future Pharmacogenomics Analysis

For subjects who signed a separate ICF, an optional whole blood sample for pharmacogenomics (PGx) will be collected within 48 hours prior to dosing at cycle 1 day 1. PGx research may be conducted in the future to analyze or determine genes of relevance to clinical response, pharmacokinetics and toxicity/safety issues. A sample of whole blood for possible retrospective PGx analysis will be collected and processed. Blood sampling, processing, storage and shipment instructions will be provided in the laboratory manual. Samples will be shipped to and stored/analyzed by a sponsor-designated analytical storage laboratory. Please refer to the laboratory manual for more detailed information.

See [Appendix 12.5 Retrospective PGx Sub-Study (Optional)] for further details on the banking procedures.

5.7.6 Quality of Life and Patient Reported Outcomes

Subjects will be asked to complete patient reported outcome questionnaires as specified in the Schedule of Assessments. The questionnaires should be administered prior to the start of any assessments and before the disease status is discussed with the subject.

5.7.6.1 Quality of Life Questionnaire

The European Organisation for Research and Treatment of Cancer (EORTC) QLQ-C30 is a cancer-specific instrument consisting of 5 functional domain scales: physical, role, emotional, social and cognitive.

5.7.6.2 Oesophago-gastric Module

The EORTC-QLQ-OG-25 instrument evaluates gastric or GEJ cancer-specific symptoms such as stomach discomfort, difficulties eating and swallowing and indigestion.

5.7.6.3 Global Pain

The GP instrument is a single assessment of overall pain.

5.7.6.4 EuroQOL Five Dimensions Questionnaire

The EQ-5D-5L is a standardized instrument for use as a measure of health outcome consisting of 6 items that cover 5 main domains (mobility, self-care, usual activities, pain/discomfort and anxiety/depression) and a general visual analog scale for health status.

5.7.6.5 Health Resource Utilization

HRU questionnaire to assess the number of office visits, hospital stays and other healthcare resource utilization that occur outside of the clinical trial.

5.8 Total Amount of Blood

The total amount of blood collected for study assessments for each subject will vary depending on how long the subject stays on treatment.

At any time during the study, if any laboratory abnormalities are found for a subject or for disease assessment, additional blood may be drawn for monitoring.

Additional blood beyond standard of care monitoring that will be drawn for this study will include draws for eligibility assessment, hematology, chemistry, coagulation, pharmacokinetics, immunogenicity and biomarkers at specific study defined time points.

The maximum amount of blood collected in cycle 1 of Cohort 1A is approximately 96 mL. In Cohort 2, the maximum amount of blood collected in cycle 1 is approximately 174 mL, and less in later cycles. In Cohort 3, the maximum amount of blood collected in cycle 1 is approximately 77 mL and less in later cycles.

The maximum amount of blood collected in cycle 1 of Cohort 4A is approximately 156 mL. In Cohort 4B, the maximum amount of blood collected in cycle 1 is approximately 136 mL, and less in later cycles.

The maximum amount of blood collected in cycle 1 of Cohort 5 is approximately 45.7 mL.

6 DISCONTINUATION

6.1 Discontinuation of Individual Subject(s)

Discontinuation from treatment is defined as a subject who enrolled in the study and for whom all study treatment (zolbetuximab, mFOLFOX6 [all components], nivolumab, pembrolizumab and/or FLOT) is permanently discontinued for any reason.

The subject is free to withdraw from the study treatment and/or study for any reason and at any time without giving reason for doing so and without penalty or prejudice. The investigator is also free to discontinue the subject from study treatment or to terminate a subject's involvement in the study at any time if the subject's clinical condition warrants it.

If a subject is discontinued from the study with an ongoing AE or an unresolved laboratory result that is significantly outside of the reference range, the investigator will attempt to provide follow-up until the condition stabilizes or no longer is clinically significant.

The subject will be discontinued from the treatment period if any of the following occur:

- Investigator determines it is in the subject’s best interest to discontinue study treatment.
- Subject develops radiographic disease progression per RECIST 1.1 criteria based on assessment by an independent central reader (Cohort 4 only: by investigator assessment).
    - If there is radiographic evidence of PD; however, the investigator believes the subject is continuing to derive clinical benefit (asymptomatic and/or without worsening of performance status or overall health) from study drug and an increase in tumor burden is not likely to affect vital organ function, the subject may remain on study drug until the next scheduled radiographic assessment.
        - If the next radiographic assessment indicated PD per RECIST 1.1, which is confirmed by the independent central reader, then the subject must be discontinued from study drug.
        - In the rare event where PD suspected on initial assessment, but is not confirmed on subsequent scan by the investigator or central reviewer, the subject may continue in the study.
    - The investigator should make every effort to immediately submit radiographic assessments for central review when PD is either suspected or confirmed or uncertainty exists.
- Subject develops clinical progression per investigator assessment and radiographic assessment is not medically feasible due to the subject’s condition (this is anticipated to be infrequent).
- Subject starts another systemic chemotherapy, immunotherapy, radiotherapy or other treatment intended for antitumor activity.
- Subject starts another investigational agent or device.

- Subject develops unacceptable toxicity that results in discontinuation of zolbetuximab (Subjects in Cohort 2, Cohort 3A, Cohort 4 or Cohort 5 may continue on mFOLFOX6/5-FU and leucovorin or folinic acid, pembrolizumab, nivolumab or FLOT, as applicable, per investigator discretion.). See [Section 5.1.1 Dose/Dose Regimen and Administration Period] for information regarding dose modifications.
- Subject has a delay of zolbetuximab, mFOLFOX6, nivolumab, pembrolizumab or FLOT treatment for > 28 days from when the next zolbetuximab, mFOLFOX6, pembrolizumab, nivolumab or FLOT treatment was scheduled to be administered (> 49 days from when the last dose began if a 3-week cycle [Cohorts 1A, 2 and 3A] and > 42 days from when the last dose began if a 2-week cycle [Cohorts 4 and 5]).
- Inter-current illness that the investigator determines may jeopardize the subject's safety if the subject continues to receive study treatment.
- Female subject becomes pregnant.
- Significant deviation from the protocol or eligibility criteria as determined by the sponsor.
- Subject declines further treatment.
- Subject declines further study participation.
- Subject is lost to follow-up despite reasonable efforts by the investigator to locate the subject.
- Death
- Sponsor discontinues the study

NOTE: If a subject discontinues mFOLFOX6 (with or without nivolumab), pembrolizumab, zolbetuximab or FLOT prior to radiographic disease progression (i.e., PFS), the subject should enter the post-treatment follow-up period and continue to undergo imaging assessments until radiographic progression is documented per the independent central reader or investigator (Cohort 4 only) or the subject starts another anti-cancer treatment, whichever occurs earlier.

Following discontinuation from all study treatments (zolbetuximab and/or mFOLFOX6 [all components]), nivolumab, pembrolizumab and/or FLOT, subjects will have follow-up visits or phone calls 30 (± 7) and 90 (± 7) days after their last dose of study drug.

Cohort 2 and 4 Only: If zolbetuximab is discontinued for radiographic disease progression, all components of mFOLFOX6 (with or without nivolumab) must also be discontinued.

6.2 Discontinuation of the Site

If an investigator intends to discontinue participation in the study, the investigator must immediately inform the sponsor. For Japan sites, the head of the study site must also be informed immediately.

6.3 Discontinuation of the Study

If the study is prematurely terminated or suspended the sponsor or designee shall promptly inform the investigators, the IECs/IRBs, the regulatory authorities, and any contract research organization(s) used in the study of the reason for termination or suspension, as specified by the applicable regulatory requirements.

In case of premature study termination, zolbetuximab may be made available to subjects who are still receiving and benefitting from study treatment, until a treatment discontinuation criterion is met.

7 STATISTICAL METHODOLOGY

A Statistical Analysis Plan (SAP) will be written to provide details of the analysis, along with specifications for tables, listings and figures to be produced. The SAP will be finalized before the database soft lock at the latest. Any changes from the analyses planned in SAP will be justified in the Clinical Study Report.

In general, continuous data will be summarized with descriptive statistics (number of subjects, mean, SD, minimum, median and maximum) and frequency and percentage for categorical data. Data summary will be provided separately for each cohort (Cohort 1, 2, 3A and 4), unless stated otherwise.

7.1 Sample Size

The sample size of 20 for Cohort 1A is not based on a strict statistical power calculation, but is expected to provide adequate early efficacy and safety information.

The sample size of 12 for Cohort 2 is to provide sufficient pharmacokinetic information of zolbetuximab, oxaliplatin and 5-FU.

The sample size of 12 for Cohort 3A is not based on a statistical power calculation but is expected to provide safety information to determine the tolerability of the dose level of interest.

The sample size of 12 for Cohort 4A is not based on a statistical power calculation but is expected to provide safety information to determine the tolerability of the dose level of interest.

The sample size of approximately 65 for Cohort 4B is not based on a strict statistical consideration. The sample size of approximately 65 is expected to yield 50 subjects with high CLDN18.2 expression. For these subjects with high CLDN18.2 expression, assuming an accrual period of 12 months and a follow-up period of 3 to 6 months and 20 to 25 PFS events, the sample size of 50 will provide 70.37% to 76.13% power to detect the difference in PFS with the assumption of a 12-month median PFS vs 8.5-month median PFS (zolbetuximab in combination of nivolumab and mFOLFOX6 vs zolbetuximab and mFOLFOX6) using a 1-sided 15% Type I error. It is assumed that the survival time distributions of both groups are approximated reasonably well by the Weibull distribution with a shape parameter of 1.

The sample size of 12 for Cohort 5 is not based on a statistical power calculation but is expected to provide safety information to determine the tolerability of the dose level of interest.

7.2 Analysis Sets

7.2.1 Full Analysis Set

The FAS consists of all subjects who were enrolled and received at least 1 dose of zolbetuximab and who have at least 1 post-treatment disease assessment. At least 1 post-treatment disease assessment is defined as, for at least one time point, for either local or central assessment, the tumor imaging overall response does not equal to NE (not evaluable).

The FAS will be used for summaries of all efficacy data except PFS and OS, as well as selected demographic and baseline characteristics.

7.2.2 Safety Analysis Set

The Safety Analysis Set (SAF) consists of all subjects who received at least 1 dose of zolbetuximab. The SAF will be used for summaries of demographic and baseline characteristics, PFS, OS, and all safety and tolerability related variables.

7.2.3 Pharmacokinetic Analysis Set

The Pharmacokinetic Analysis Set (PKAS) consists of the subset of the SAF for which at least 1 concentration data is available for any analyte. Additional subjects may be excluded from the PKAS at the discretion of the pharmacokineticist. The PKAS is used for all tables and graphical summaries of the pharmacokinetic data.

7.2.4 Biomarker Analysis Set

The Biomarker Analysis Set (BMAS) consists of the subset of the SAF for which at least 1 pre-treatment or 1 on-treatment biomarker measurement is available. Additional subjects may be excluded from the BMAS at the discretion of the sponsor. The BMAS is used for all tables and graphical summaries of the biomarker data.

7.2.5 DLT Evaluation Analysis Set

The DLT Evaluation Analysis Set (DEAS) is defined as all subjects in the SAF excluding subjects without a DLT who receive less than the prescribed dose in cycle 1, or do not complete cycle 1 evaluations for a reason other than a DLT (e.g., consent withdrawal).

7.3 Demographics and Baseline Characteristics

Demographics and baseline characteristics (age, sex, race, ethnicity, weight, height and body mass index) will be summarized for each cohort.

7.3.1 Subject Disposition

The number and percentage of subjects who completed and discontinued treatment and reasons for treatment discontinuation will be presented for all registered subjects and for subjects in the SAF by treatment group and overall. Similar tables for screening disposition, investigational period disposition and follow-up disposition will also be presented for all registered subjects by treatment group and overall. All disposition details and dates of first and last evaluations for each subject will be listed.

7.3.2 Previous and Concomitant Medications

All previous and concomitant medications will be presented in a listing. The frequency of concomitant medications (prescription, over-the-counter and nutritional supplements) will be summarized.

7.3.3 Medical History

Medical history for each subject will be presented in a listing.

7.4 Analysis of Efficacy

7.4.1 Analysis of Primary Endpoint

The primary analysis will occur once all subjects have progressed, died, discontinued form the study or provided at least 1 post-baseline disease assessment, whichever occurs first. The ORR is defined as the proportion of subjects with complete or partial objective response based on RECIST V1.1. ORR will be summarized using exact 95% confidence interval. Best overall response is determined once all tumor response data for the subject is available. Subject will be classified by best response on study as outlined in RECIST V1.1 criteria.

7.4.2 Analysis of Secondary Endpoints

7.4.2.1 Disease Control Rate

The DCR is defined as the proportion of subjects with complete or partial objective response, or stable disease based on RECIST V1.1. DCR will be summarized using the same method used for ORR.

7.4.2.2 Duration of Response

DOR is defined as the time from the date of the first response CR/PR (whichever is first recorded) to the date of radiographic progression/death or date of censoring. DOR will be derived for subjects with confirmed response as CR or PR. The distribution of DOR will be estimated using Kaplan-Meier methodology. Median DOR and 95% confidence interval will be provided.

7.4.2.3 Clinical Response and Pathological Response

For zolbetuximab and FLOT (Cohort 5), the clinical response to preoperative chemotherapy (timepoints include baseline tumor assessment [prior to study treatment] to before surgery and after completion/discontinuation of preoperative chemotherapy) will be summarized using the following:

1. Percentage of subjects with radiological response at restaging will be summarized using exact 95% confidence intervals.
2. Percentage of subjects with pathological response (ypTNM) will be summarized using exact 95% confidence intervals.

Radiological response will include CR and PR.

Pathological response (ypTNM) will include ypCR, ypPR and major pathologic response.

7.4.2.4 Progression-free Survival

For each subject, PFS is defined as the time from date of treatment start until the date of radiographic disease progression, or until death due to any cause, whichever is earliest. The survival curve and the median PFS will be estimated using Kaplan-Meier method and will be reported along with corresponding 95% confidence interval.

In addition, PFS rate and its 95% confidence interval will be estimated at 3 months, 6 months, 9 months, and 12 months using Kaplan-Meier method.

7.4.2.5 Disease-free Survival

DFS is defined as the time from date of treatment start until the date of radiological disease recurrence or until death due to any cause, whichever is earliest. DFS will be analyzed using the same method as PFS (i.e., Kaplan-Meier method).

7.4.2.6 Overall Survival

OS is defined as the time from the date of treatment start until the documented date of death from any cause. All events of death will be included, regardless of whether the event occurred while the subject is still taking study drug or after the subject discontinue study drug. Subjects who are still alive at the time of analysis will be censored at the last day known to be alive. OS will be analyzed using the same method as PFS (i.e., Kaplan-Meier method).

7.4.2.7 HRQoL

HRQoL will be summarized by descriptive statistics by the QLQ-C30, OG-25, GP, EQ-5D and HRU questionnaires for each cohort. The change from baseline to final visit or early termination will also be summarized.

7.4.2.8 Minimal Residual Disease

Minimal residual disease and disease recurrence as measured by ctDNA will be summarized. Descriptive statistics will be used to summarize results and change from baseline by time point.

7.4.3 Analysis of Exploratory Endpoints

7.4.3.1 Biomarkers

Tumor expression of CLDN18.2 measured by IHC in baseline and on treatment biopsy samples, immune-related biomarkers assessed in tumor tissue and blood samples will be summarized graphically or descriptively, and summary statistics may be tabulated. Other biomarkers such as ctDNA may be summarized graphically or descriptively, and summary statistics may be tabulated. Associations between clinical (e.g., efficacy, safety, pharmacodynamics or pharmacokinetics) measures and biomarkers, including changes from baseline, may be performed on subjects who have sufficient baseline and on-study measurements to provide interpretable results for specific parameters. Additional post-hoc statistical analyses may be outlined in the SAP.

7.4.4 Imaging

A list of tumor imaging data will be provided.

7.5 Analysis of Safety

7.5.1 Adverse Events

AEs will be coded using MedDRA and graded using NCI-CTCAE. The number and percentage of AEs, SAEs, AEs leading to interruption/discontinuation, AEs leading to death and AEs related to study drug will be summarized by system organ class and preferred term for each cohort. The number and percentage of AEs by toxicity grade will also be summarized for each cohort. All AEs will be listed. Summary statistics will also be provided for laboratory parameters, vital signs, drug exposure and other safety parameters. All DLT incidences will be summarized for Cohorts 3A, 4A and 5.

A treatment-emergent AE (TEAE) is defined as an AE observed after starting the administration of the study drug and within 30 days after the last dose of study drug.

A study drug-related TEAE is defined as any TEAE with a causal relationship of YES by the investigator.

AEs of special interest described in [Section 5.5.8 Adverse Events of Special Interest] will be summarized.

7.5.2 Laboratory Assessments

For quantitative laboratory measurements descriptive statistics will be used to summarize results and change from baseline by time point for each cohort. Shifts from baseline to the worst grade based on NCI-CTCAE during treatment period in laboratory tests will also be tabulated.

7.5.3 Vital Signs

Descriptive statistics will be used to summarize vital sign results and changes from baseline for subjects in the SAF by time point.

7.5.4 Physical Examination

Physical examination will be listed by treatment group.

7.5.5 Routine 12-lead Electrocardiograms

For all analyses, replicates at each time point will be averaged for each continuous ECG parameter. Baseline will be defined as cycle 1 day 1 predose measurements.

Descriptive statistics will be used to summarize the continuous ECG parameter results and changes from baseline for subjects in the SAF by time point for each cohort. A shift analysis table showing shifts from baseline in overall ECG (normal and abnormal) will be provided.

The QT corrected by Fridericia's Correction formula (QTcF) interval will be summarized using frequency tables for values of clinical importance using the range criteria below.

	Corrected QT (QTcF) Interval Criteria Value (msec)
Normal	≤ 450
Borderline	> 450
Prolonged	> 480
Clinically significant	> 500

QTcF: Fridericia-corrected QT interval

The QT corrected (QTc) interval will also be summarized by the frequencies of subjects with a change from baseline of clinical importance using the criteria identified below.

Variable	Change from Baseline
Corrected QT (QTcF) Interval (msec)	< 0<br>≥ 0<br>> 30<br>> 60

QTcF: Fridericia-corrected QT interval

All ECG data (individual replicates and the averages) will be displayed in listings.

Effects of serum concentrations of zolbetuximab on ΔQTcF (defined as the mean change from baseline in QTcF) will be assessed.

7.5.6 Other Safety Aspects

The following safety measures will be summarized for zolbetuximab and FLOT (Cohort 5):

a. Percentage of subjects with surgical complications
b. Percentage of subjects with surgical mortality as defined by death within 30 days of surgery
c. Percentage of subjects able to complete preoperative chemotherapy
d. Percentage of subjects with perioperative mortality and morbidity at 30 days and 90 days post last dose
e. Percentage of subjects able to start postoperative chemotherapy
f. Percentage of subjects able to complete postoperative chemotherapy

7.6 Analysis of Pharmacokinetics

Descriptive statistics will include the number of subjects (n), mean, SD, coefficient of variation (CV), geometric mean, geometric CV, median, minimum, maximum. For the pharmacokinetic parameters t_{max} and t_{last} only n, median, minimum and maximum will be calculated.

7.6.1 Concentrations

Serum concentrations of zolbetuximab, pembrolizumab and nivolumab, and plasma concentrations of oxaliplatin (measured as total and free platinum) and 5-FU, will be listed and summarized using descriptive statistics by scheduled time point. Standard graphics

include mean serum or plasma concentration-time profiles, overlay (spaghetti) plots and individual subject serum or plasma concentration-time profiles will be produced.

7.6.2 Estimation of Pharmacokinetic Parameters

Noncompartmental analysis will be used for the calculation of plasma and serum pharmacokinetic parameters using Phoenix version 6.3 or higher (Certara L.P., 100 Overlook Center Suite 101, Princeton, NJ 08540, US).

Serum pharmacokinetic parameters of zolbetuximab and plasma parameters of oxaliplatin (measured as total and free platinum) and 5-FU will be listed and summarized using descriptive statistics.

7.6.3 Statistical Analysis of Pharmacokinetic Parameters

To evaluate potential effect of zolbetuximab on the pharmacokinetics of oxaliplatin and 5-FU in Cohort 2, the geometric least-squares mean ratio (cycle 2 day 1 vs cycle 1 day 1) and associated 90% confidence intervals will be provided for C_{max} and AUCs of total platinum, free platinum and 5-FU for Cohort 2.

7.6.4 Immunogenicity

Immunogenicity of zolbetuximab will be summarized using the frequency of ADA positive subjects for each cohort.

The potential relationship between zolbetuximab immunogenicity and zolbetuximab pharmacokinetics, efficacy and safety profiles in subjects may be assessed.

7.7 Major Protocol Deviations

Major protocol deviations as defined in [Section 8.1.6 Major Protocol Deviations] will be summarized for all enrolled subjects by cohort and total as well as by site. A data listing will be provided by site and subject.

The major protocol deviation criteria will be uniquely identified in the summary table and listing.

7.8 Interim Analysis (and Early Discontinuation of the Clinical Study)

No interim analysis will be performed.

7.9 Handling of Missing Data, Outliers, Visit Windows and Other Information

As a general principle, no imputation of missing data will be done. Exceptions are the start and stop dates of AEs and concomitant medication. The imputed dates will be used to determine whether or not an AE is treatment emergent and the medication is concomitant. Listings of the AEs and concomitant medications will present the actual partial dates; imputed dates will not be shown.

See the SAP for details of the definitions for windows to be used for analyses by visit.

8 OPERATIONAL AND ADMINISTRATIVE CONSIDERATIONS

8.1 Procedure for Clinical Study Quality Control

8.1.1 Data Collection

The investigator or site designee will enter data collected using an electronic data capture system. In the interest of collecting data in the most efficient manner, the investigator or site designee should record data (including laboratory values, if applicable) in the eCRF within 5 days after the subject visit.

The investigator or site designee is responsible to ensure that all data in the eCRFs and queries are accurate and complete and that all entries are verifiable with source documents. These documents should be appropriately maintained by the site.

Local laboratory results may be used for treatment decisions; however, central laboratory samples must also be drawn per protocol and sent to the central laboratory, unless otherwise approved by sponsor. Central laboratory data will be transferred electronically to the sponsor or designee at predefined intervals during the study. The central laboratory will provide the sponsor or designee with a complete and clean copy of the data.

For Cohorts 1A and 2, ECG results are performed at a central ECG laboratory. Central ECG read data will be transferred electronically to the sponsor or designee at predefined intervals during the study. The central ECG laboratory will provide the sponsor or designee with a complete and clean copy of the data.

Local ECGs will be performed at defined time points for Cohorts 3A, 4 and 5 and will also be assessed prior to every oxaliplatin infusion and following completion of every oxaliplatin infusion.

For screen failures the demographic data, reason for failing, informed consents, inclusion and exclusion criteria, ECOG performance status, primary diagnosis, substance use-tobacco history and AEs will be collected in the eCRF.

Pre-screening data will be collected for subjects in Cohorts 4A, 4B and 5, which include date of the pre-screening ICF and tumor sample collection status, demographics, inclusion and exclusion criteria (if applicable), primary diagnosis, substance use-tobacco history and protocol version.

8.1.1.1 Collection of Data Via Electronic Source and SAS Transfer

All procedures conducted under the protocol must be documented. For screen failures, the minimum demographic data (sex, birth date, race and informed consent date), outcome of eligibility assessment (inclusion and exclusion criteria), reason for screen failure and AEs details must be documented.

The investigator or designee will be responsible for case report form or source data completion and that all data and queries are accurate, complete and are verifiable with the source. The source should be appropriately maintained by the clinical unit.

Electronic data sources and any supporting documents should be available for review/retrieval by the sponsor/designee at any given time.

8.1.1.2 Electronic Patient Reported Outcome

Subject diaries and questionnaires will be completed by the subject on an electronic device. The information completed by the subject on the electronic device will be automatically uploaded into a central website. The investigator or site designee should review the diaries and questionnaire data on the website or electronic device for correct completion while the subject is at the site. The diary and questionnaire data will be transferred electronically to sponsor or designee at predefined intervals during the study. The vendor will provide sponsor or designee with a complete and clean copy of the data.

8.1.1.3 Data Quality Assurance

All participant data relating to the study will be recorded on the eCRF unless transmitted to the sponsor or designee electronically in an external data file (e.g., central laboratory data). The investigator is responsible for verifying that data entries on the eCRF are accurate and correct by physically or electronically signing the eCRF.

Guidance on completion of CRFs will be provided in a separate Electronic Case Report Form (eCRF) Completion Guideline.

The investigator must permit study-related monitoring, audits, IRB/IEC review, and regulatory agency inspections and provide direct access to source data documents. Monitoring details describing strategy, including definition of study critical data items and processes (e.g., risk-based initiatives in operations and quality such as risk management and mitigation strategies and analytical risk-based monitoring), methods, responsibilities and requirements, including handling of noncompliance issues and monitoring techniques (central, remote, or on-site monitoring) are provided in the [Monitoring Plan] [contracts].

The sponsor or designee is responsible for the data management of this study including quality checking of the data.

The sponsor assumes accountability for actions delegated to other individuals (e.g., CROs).

Records and documents, including signed ICFs, pertaining to the conduct of this study must be retained by the investigator according to ICH or applicable local regulatory requirements, whichever is longer, after study completion. No records may be destroyed during the retention period without the written approval of the sponsor. No records may be transferred to another location or party without written notification to the sponsor.

8.1.2 Specification of Source Documents

Source data must be available at the site to document the existence of the study subjects and to substantiate the integrity of study data collected. Source data must include the original documents relating to the study, as well as the medical treatment and medical history of the subject.

The investigator is responsible for ensuring the source data are attributable, legible, contemporaneous, original, accurate and complete whether the data are hand-written on paper or entered electronically. If source data are created (first entered), modified, maintained, archived, retrieved or transmitted electronically via computerized systems (and/or other kind of electric devices) as part of regulated clinical trial activities, such systems must be compliant with all applicable laws and regulations governing use of electronic records and/or electronic signatures. Such systems may include, but are not limited to, electronic medical/health records, protocol related assessments, AE tracking, and/or drug accountability.

Paper records from electronic systems used in place of electronic format must be certified copies. A certified copy must be an exact copy and must have all the same attributes and information as the original. Certified copies must include signature and date of the individual completing the certification. Certified copies must be a complete and chronological set of study records (including notes, attachments, and audit trail information (if applicable). All printed records must be kept in the subject file and available for archive.

Study monitors will perform ongoing source data review to confirm that the safety and rights of participants are being protected; and that the study is being conducted in accordance with the currently approved protocol and any other study agreements, ICH GCP, and all applicable regulatory requirements.

Specific to Japan sites:

The following are the major documents to be retained at the study site.

1. Source documents (clinical data, documents and records for preparing the CRF), hospital records, medical records, test records, memoranda, checklists for evaluation, administration records, data recorded by automatic measuring instruments, reproductions or transcripts verified as precise copies, microfiche, negative films, microfilms/magnetic media, X-ray films, participant files and study-related records kept at either a pharmacy, a laboratory, or medical technical office, as well as participant registration forms, laboratory test slips including central measurement, worksheets specified by the sponsor, records of clinical coordinators, and records related to the study selected from those verified in other departments or hospitals.
2. Study contracts, written ICFs, written information and other documents or their copies prepared by the study personnel. A letter of request for study (including a request for continuation/amendment), letter of request for review, notice of study contract, study contract, notification of discontinuation or completion of clinical study, written information for informed consent (including revisions), signed and dated written informed consent (including revisions), curriculum vitae of investigators, list of subinvestigators, list of signatures and print of seals (copy) and CRF (copy), etc.
3. The protocol, documents obtained from the IRB related to the adequacy of conducting the study by the head of the study sites (Article 32-1, MHW Ordinance No. 28), documents obtained from the IRB related to the adequacy of conducting a study whose period exceeds one year or the adequacy of continuously conducting the study from

which information on adverse drug reactions is obtained, and other documents obtained. A finalized protocol (including revisions), finalized IB (including revisions), operational procedures for the investigator, materials and information supplied by the sponsor (e.g., AE report), matters reported by the investigator (revisions of the protocol, AE reports, etc.), operational procedures for the IRB, the list of names of the IRB members, materials for IRB review (including continuous deliberation), IRB review records (including continuous deliberation) and the review result report of the IRB (including continuous deliberation), etc.

4. Records of control for IP and other duties related to the study. Procedure for controlling the IP, drug inventory and accountability record, vouchers for the receipt and return of the IP, and the prescriptions for concomitant medications

8.1.3 Clinical Study Monitoring

The sponsor or delegated CRO is responsible for monitoring the clinical study to ensure that subject's human rights, safety and well-being are protected, that the study is properly conducted in adherence to the current protocol, GCP, and EU CTR regulations (for EU sites), and study data reported by the investigator/subinvestigator are accurate and complete and that they are verifiable with study-related records such as source documents. The sponsor is responsible for assigning study monitor(s) to this study for proper monitoring. They will monitor the study in accordance with planned monitoring procedures.

8.1.4 Direct Access to Source Data/Documents

The investigator and the study site must accept monitoring and auditing by the sponsor or delegated CRO as well as inspections from the IRB/IEC and relevant regulatory authorities. In these instances, they must provide all study-related records, such as source documents [refer to Section 8.1.2 Specification of Source Documents] when they are requested by the sponsor monitors and auditors, the IRB/IEC or regulatory authorities. The confidentiality of the subject's identities shall be well protected consistent with local and national regulations when the source documents are subject to direct access.

8.1.5 Data Management

Data Management will be coordinated by the Data Science of the sponsor in accordance with the SOPs for data management. All study-specific processes and definitions will be documented by Data Management. eCRF completion will be described in the eCRF instructions. Coding of medical terms and medications will be performed using MedDRA and WHO Drug Dictionary, respectively.

8.1.6 Major Protocol Deviations

A major protocol deviation is generally an unplanned excursion from the protocol that is not implemented or intended as a systematic change. All deviations from the protocol are to be recorded. A protocol waiver is a documented prospective approval of a request from an investigator to deviate from the protocol. Protocol waivers are strictly prohibited.

The investigator is responsible for ensuring the study is conducted in accordance with the procedures and evaluations described in this protocol and must protect the rights, safety and welfare of subjects. The investigator should not implement any deviation from, or changes of, the protocol, unless it is necessary to eliminate an immediate hazard to study subjects.

When a major deviation from the protocol is identified for an individual subject, the investigator or designee must ensure the sponsor is notified. The sponsor will follow-up with the investigator, as applicable, to assess the deviation and the possible impact to the safety, efficacy or pharmacokinetic parameters of the subject to determine subject continuation in the study.

If a major deviation impacts the safety of a subject, the investigator must contact the sponsor immediately.

Major protocol deviation criteria will be summarized at the end of the study.

The investigator will also assure that deviations meeting IRB/IEC and applicable regulatory authorities' criteria are documented and communicated appropriately. All documentation and communications to the IRB/IEC and applicable regulatory authorities will be provided to the sponsor and maintained within the trial master file.

NOTE: Other deviations outside of the categories defined above that are required to be reported by the IRB/IEC in accordance with local requirements will be reported as applicable.

8.1.7 Start and End of Trial in All Participating Countries

First act of recruitment

The study start is defined as the date on which the clinical study will be open for recruitment of participants.

The first act of recruitment is the date the first participant signs the ICF and will be the study start date.

End of study

The end of the study is defined as the last visit or scheduled procedure shown in the Schedule of Assessments for the last study participant in the study.

A participant is considered to have completed the study if the participant has completed all periods of the study including the last assessment shown in the schedule of assessments.

Study completion is defined as the conclusion of data collection for the defined study endpoints. The study may be closed within a participating country per local regulations once the study has completed and if all subjects enrolled in the country are no longer receiving study treatment. In addition, the sponsor may prematurely terminate the study for reasonable cause at any time.

8.2 Ethics and Protection of Subject Confidentiality

8.2.1 Institutional Review Board/Independent Ethics Committee/Competent Authorities

GCP requires that the clinical protocol, any protocol amendments, the IB, the informed consent and all other forms of subject information related to the study (e.g., advertisements used to recruit subjects) and any other necessary documents be reviewed by an IEC/IRB. The IEC/IRB will review the ethical, scientific and medical appropriateness of the study before it is conducted. IEC/IRB approval of the protocol, informed consent and subject information and/or advertising, as relevant, will be obtained prior to the authorization of drug shipment to a study site.

Any substantial amendments to the protocol will require IEC/IRB approval prior to implementation of the changes made to the study design at the site. The investigator will be required to submit, maintain and archive study essential documents according to ICH GCP.

Any SAEs that meet reporting criteria, as dictated by local regulations, will be reported to both responsible ethics committees and regulatory agencies, as required. During the conduct of the study, the investigator should promptly provide written reports (e.g., ICH Expedited Reports, and any additional reports required by local regulations) to the IEC/IRB of any changes that affect the conduct of the study and/or increase the risk to subjects. Written documentation of the submission to the IEC/IRB should also be provided to sponsor.

If required by local regulations, the investigator shall make accurate and adequate written progress reports to the IEC/IRB at appropriate intervals, not exceeding 1 year. The investigator shall make an accurate and adequate final report to the IRB/IEC within 90 days after the close-out visit for APGD-sponsored studies, or for Astellas Pharma Europe BV/ Astellas Pharma Europe Ltd.-sponsored studies within 1 year after last subject out or termination of the study.

8.2.2 Ethical Conduct of the Study

The study will be conducted in accordance with the protocol, ICH guidelines, applicable regulations and guidelines governing clinical study conduct and the ethical principles that have their origin in the Declaration of Helsinki.

8.2.3 Informed Consent of Subjects

8.2.3.1 Subject Information and Consent

The investigator or his/her representative will explain the nature of the study to the subject or his/her guardian or legal representative, and answer all questions regarding this study. Prior to any study-related screening procedures being performed on the subject, the informed consent statement will be reviewed and signed and dated by the subject or his/her guardian or legal representative, the person who administered the informed consent and any other signatories according to local requirements. A copy of the signed ICF will be given to the subject and the original will be placed in the subject’s medical record. An entry must also be

made in the subject’s dated source documents to confirm that informed consent was obtained prior to any study-related procedures and that the subject received a signed copy.

Subject or his/her guardian or legal representative will be required to sign a statement of informed consent that meets the requirements of 21 CFR 50, EU CTR 536/2014 (including Article 29), local regulations, ICH GCP guidelines, the Declaration of Helsinki, HIPAA requirements, where applicable, and the IRB/IEC or study center.

The signed consent forms will be retained by the investigator and made available (for review only) to the study monitor and auditor regulatory authorities and other applicable individuals upon request.

8.2.3.2 Supply of New and Important Information Influencing the Subject's Consent and Revision of the Written Information

1. The investigator or his/her representative will immediately inform the subject orally whenever new information becomes available that may be relevant to the subject's consent or may influence the subject's willingness to continue to participate in the study (e.g., report of serious drug adverse drug reaction). The communication must be documented in the subject's medical records and whether the subject is willing to remain in the study or not must be confirmed and documented.
2. The investigator must update their ICF and submit it for approval to the IRB/IEC. The investigator or his/her representative must obtain written informed consent from the subject on all updated ICFs throughout their participation in the study. The investigator or his/her designee must reconsent subjects with the updated ICF even if relevant information was provided orally. The investigator or his/her representative who obtained the written informed consent and the subject should sign and date the ICF. A copy of the signed ICF will be given to the subject and the original will be placed in the subject's medical record. An entry must be made in the subject's records documenting the re-consent process.

8.2.4 Data Protection

The sponsor will use the personal data collected from participants in order to run the study and to use and publish the results of the study. The personal data of participants will be used throughout the development program of the investigational study intervention; e.g., to develop a product, obtain permission to market the product, monitor its safety and obtain coverage by health insurance and reimbursement schemes.

Participants will participate in the study only if they consent to it. Their personal information will be collected and processed for the above purposes on the basis of:

- Astellas's obligation to comply with legal obligations to which Astellas is subject, related in particular to pharmacovigilance, i.e., safety reporting of AEs and for compliance with other local laws and regulations. In these cases, processing of the participants' personal information is necessary for reasons of public interest in the area of public health, such as ensuring high standards of quality and safety of the investigational study intervention;
- Astellas's legitimate interest to evaluate the efficacy of the investigational study intervention. In that case, processing the participants' personal information is necessary for scientific research purposes.

The investigator and/or the site personnel will record information from the medical file of study participants in the eCRF and in an external (electronic) data file (e.g., central laboratory data). These records will identify the participants with a code instead of their name or other personal data. Only the investigator and the site personnel can match the code with the name of the study participant which will be retained in the medical file at the site only. Non-medical personnel acting on behalf of Astellas and being bound by a duty of confidentiality, as well as health authorities and/or IRB/IECs, may also be given access to this data at the site only to verify that the study is carried out in compliance with legal and quality requirements.

The sponsor collects information from the participants during the study that may be used for:

- submission to government regulatory authorities and IRB/IEC
- use in reports or public scientific presentations, and
- use in research, now or in the future.

However, the identity of participants will not be revealed if study participants' personal data is shared for these purposes.

The personal data of participants may be shared with:

- Astellas and its present or future affiliates,
- research, collaboration and licensing partners and/or service providers (such as laboratories conducting tests on behalf of Astellas) and authorized representatives of Astellas,
- study monitors appointed by Astellas or Astellas' service providers, and
- auditors/inspectors appointed by Astellas or Astellas' service providers or by health and regulatory authorities for GCP inspections.

The sponsor will inform the participants about their privacy rights and how to exercise them under the Informed Consent Form which participants will sign in order to participate in the study. The sponsor will provide the investigator and study staff with a privacy notice explaining how their personal data will be used and how to exercise their privacy rights.

The sponsor will comply and process personal data in accordance with all applicable privacy laws and regulations.

The Sponsor has performed a privacy impact assessment to describe the data processing activities, assess their necessity and proportionality and manage the risks to the rights and freedoms of data subjects resulting from the processing of personal information by assessing them and determining the measures or mitigation actions to address them. The Sponsor has assessed, among others, how the data is collected and the purposes they are used, the impact on data subject rights, the security mechanisms including the safeguards of potential transfer of data outside the European Union and the access rights to data. The Sponsor has taken actions to mitigate the risks to the rights and freedoms of data subjects resulting from the processing of their data as part of their participation in the study.

The contract between sponsor and study sites specifies responsibilities of the parties related to data protection, including handling of data security breaches and respective communication and cooperation of the parties.

Information technology systems used to collect, process, and store study-related data are secured by technical and organizational security measures designed to protect such data against accidental or unlawful loss, alteration, or unauthorized disclosure or access.

8.3 Administrative Matters

8.3.1 Arrangement for Use of Information and Publication of the Clinical Study

Information concerning the study drug, patent applications, processes, unpublished scientific data, the IB and other pertinent information is confidential and remains the property of the sponsor. Details should be disclosed only to the persons involved in the approval or conduct of the study. The investigator may use this information for the purpose of the study only. It is understood by the investigator that the sponsor will use the information obtained during the clinical study in connection with the development of the drug and therefore may disclose it as required to other clinical investigators or to regulatory agencies. In order to allow for the use of the information derived from this clinical study, the investigator understands that he/she has an obligation to provide the sponsor with all data obtained during the study.

Publication of the study results is discussed in the clinical study agreement.

8.3.2 Documents and Records Related to the Clinical Study

The sponsor will provide the investigator and/or institution with the following:

- Study protocol (and amendments, where applicable)
- IB (and amendments, where applicable)
- eCRFs
- Study drug with all necessary documentation
- Study contract

In order to start the study, the investigator and/or study site is required to provide the following documentation to the sponsor:

- Financial disclosure in compliance with federal regulation 21CFR Part 54
- Signed and dated FDA form 1572
- Signed Investigator's Statement in this protocol and eCRF
- Current Curricula Vitae of all investigators
- List of subinvestigators and collaborators
- IRB approval of the protocol, protocol amendments (if applicable) including a membership list with names and qualification (COPY)
- Study contract
- Laboratory normal reference ranges (if applicable, signed and dated by the responsible laboratory employee)

Records and documents, including signed ICFs, pertaining to the conduct of this study must be retained by the investigator according to ICH or applicable local regulatory requirements, whichever is longer, after study completion. No records may be destroyed during the retention period without the written approval of the sponsor. No records may be transferred to another location or party without written notification to the sponsor.

All data will be entered on the CRFs supplied for each subject.

Unique to Japan region:

The following are the major documents to be retained at the study site.

1. Source documents (clinical data, documents and records for preparing the CRF), hospital records, medical records, test records, memoranda, or check lists for evaluation, administration records, data recorded by automatic measuring instruments, reproductions or transcripts verified as precise copies, microfiche, negative films, microfilms/magnetic media, X-ray films, subject files and study-related records kept at either a pharmacy, a laboratory, or medical technical office, as well as subject registration forms, laboratory test slips including central measurement, worksheets specified by the sponsor, records of clinical coordinators, and records related to the clinical study selected from those verified in other departments or hospitals.
2. Contracts, written ICFs, written information, and other documents or their copies prepared by the study personnel. A letter of request for clinical study (including a request for continuation/amendment), letter of request for review, notice of clinical study contract, clinical study contract, notification of discontinuation or completion of clinical study, written information for informed consent (including revisions), signed and dated written informed consent (including revisions), curriculum vitaes of investigators, list of subinvestigators, list of signatures and print of seals (copy), and CRFs (copy), etc.
3. The protocol, documents obtained from the IRB related to the adequacy of conducting the clinical study by the head of the study sites (Article 32-1, MHW Ordinance No. 28), documents obtained from the IRB related to the adequacy of conducting a clinical study whose period exceeds 1 year or the adequacy of continuously conducting the clinical study from which information on adverse drug reactions is obtained, and other documents obtained. An agreed-upon protocol (including revisions), IB (including revisions), operational procedures for the investigator, materials and information supplied by the sponsor (e.g., AE report), matters reported by the investigator (revisions of the protocol, AE reports, etc.), operational procedures for the IRB, the list of names of the IRB members, materials for IRB review (including continuous deliberation), IRB review records (including continuous deliberation), and the review result report of the IRB (including continuous deliberation), etc.
4. Records of control for study drugs and other duties related to the clinical study. Procedure for controlling the study drugs, drug inventory and accountability record, vouchers for the receipt and return of the study drugs, and the prescriptions for concomitant medications

If applicable, the documents of the Efficacy and Safety Evaluation Committee (minutes and SOPs and others) and the judgment committee outside the study sites (minutes and SOPs and others) shall be retained by the sponsor.

8.3.3 Protocol Amendment and/or Revision

Any changes to the study that arise after approval of the protocol must be documented as protocol amendments: substantial amendments and/or nonsubstantial amendments.

Depending on the nature of the amendment, either IRB/IEC, Competent Authority approval or notification may be required. The changes will become effective only after the approval of the sponsor, the investigator, the regulatory authority and the IRB/IEC (if applicable).

Amendments to this protocol must be signed by the sponsor and the investigator. Written verification of IRB/IEC approval will be obtained before any amendment is implemented which affects subject safety or the evaluation of safety and/or efficacy or pharmacokinetics. Modifications to the protocol that are administrative in nature do not require IRB/IEC approval, but will be submitted to the IRB/IEC for their information, if required by local regulations.

If there are changes to the informed consent, written verification of IRB/IEC approval must be forwarded to the sponsor. An approved copy of the new informed consent must also be forwarded to the sponsor.

8.3.4 Insurance of Subjects and Others (ex-US sites only)

The sponsor has covered this study by means of an insurance of the study according to national requirements. The name and address of the relevant insurance company, the certificate of insurance, the policy number and the sum insured are provided in the investigator's file.

***Specific to Japan sites*:**

If a participant suffers any study-related injury, the sponsor will compensate the participant appropriately according to the severity and duration of the damage. However, if the injury was caused intentionally or was due to gross negligence by the study site, the sponsor will consult with the study site about handling the injury, based on the agreed study contract. Compensation for the study-related injury is provided by the following procedures:

1. If a participant incurs an injury as a result of participation in the study, the study site should provide medical treatment and other necessary measures. The sponsor should be notified of the injury.
2. When the participant claims compensation from the study site for the above study-related injury, or such compensation may be claimed, the study site should immediately communicate the fact to the sponsor. Both parties should work together towards a compensation settlement.
3. The sponsor shall pay compensation or indemnification and bear expenses necessary for the settlement as provided in the study contract.
4. The sponsor shall make an arrangement for insurance and take measures necessary to ensure the compensation or indemnification mentioned above.

Specific to interventional studies enrolling participants in EU:

The sponsor has covered this study by means of an insurance of the study according to national requirements. The name and address of the relevant insurance company, the certificate of insurance, the policy number and the sum insured are provided in the investigator’s file.

8.3.5 Dissemination of Clinical Study Data

ICH E3 guidelines recommend and EU Clinical Trial Regulation 536/2014 requires that a final CSR that forms part of a marketing authorization application, be signed by the representative for the coordinating investigator(s) or the principal investigator(s). The representative for the coordinating investigator(s) or the principal investigator(s) will have the responsibility to review the final study results to confirm to the best of their knowledge it accurately describes the conduct and results of the study. The representative for the coordinating investigator(s) or the principal investigator(s) will be selected from the participating investigators by the sponsor prior to database lock.

Unique to Japan Region

The medical advisor and/or the representative for the coordinating investigator(s) or the principal investigator(s) will have the responsibility to review the final study results to confirm to the best of their knowledge that it accurately describes the conduct and results of the study. The signatory will be the medical advisor and/or the representative for the coordinating investigator(s) or the principal investigator(s).

8.3.5.1 Disclosure of Study Information and Results

Astellas complies with relevant laws, regulatory requirements and industry guidance for registration of clinical trial information and disclosure of clinical trial results.

Astellas commits to registering all interventional clinical trials with a medicinal product sponsored by Astellas that seek to evaluate the safety and/or efficacy profile of an Astellas owned or in-licensed product. Clinical trials sponsored by Astellas that are covered under Astellas policy are registered on a publicly accessible clinical trial registry (e.g., www.clinicaltrials.gov). In addition, other Astellas sponsored studies (e.g., noninterventional studies, medical device, early access) are registered on national registries, if required by local/regional laws or regulations.

Astellas commits to disclosing summary results for all phase 1 to 4 interventional clinical trials with a medicinal product sponsored by Astellas, conducted in the target patient population, with Astellas products that have health authority approval. Summary results are also disclosed for clinical trials conducted with medicinal products that are terminated during development. These summary results are posted on the clinical trial results websites (www.clinicaltrials.astellas.com and/or www.trialsummaries.com/home/landingpage). In addition, summary results for other Astellas-sponsored studies (e.g., noninterventional studies, medical device, early access) are disclosed on national registries, if required by local/regional laws or regulations.

Summary of end of study results will be available only after all data have been obtained from all participating countries/sites. Statistical analyses of end of study results are conducted when all relevant data are available.

8.3.5.2 Access to Study Data

Subject to compliance with the applicable laws and regulations relevant to protection of personal data, Astellas provides a platform (www.clinicalstudydatarequest.com) where researchers may request access to participant level data, trial level data and protocols from Astellas sponsored clinical studies with a medicinal product conducted in patients that are completed after January 1, 2010.

Access to this data is granted for medicinal products and indications approved in any country after the request has been reviewed and approved by an independent panel of experts (“Scientific Review Board”) based on scientific merit and the qualifications of the researcher. Access is given by Astellas after review and approval by the Scientific Review Board and execution of a data sharing agreement.

Before participant-level data are shared, it is anonymized to respect the rights of the clinical trial participants to privacy and to protection of their personal health information in accordance with the applicable laws and regulations.

9 QUALITY ASSURANCE

The sponsor is implementing and maintaining quality assurance and quality control systems with written SOPs to ensure that studies are conducted and data are generated, documented, recorded, and reported in compliance with the protocol, GCP and applicable regulatory requirement(s). Where applicable, the quality assurance and quality control systems and written SOPs of the CRO will be applied.

The sponsor or sponsor's designee may arrange to audit the clinical study at any or all investigational sites and facilities. The audit may include on-site review of regulatory documents, CRFs and source documents. Direct access to these documents will be required by the auditors.

10 STUDY ORGANIZATION

10.1 Independent Data-Monitoring Committee/Data and Safety Monitoring Board

No independent data and safety monitoring will be done for this study.

10.2 Other Study Organization

Specific to Japan sites: The Japan site contact list is kept as a separate attachment to the protocol.

11 REFERENCES

Ajani JA. Standard chemotherapy for gastric carcinoma: is it a myth? J Clin Oncol. 2000;18:4001-3. [Comment in J Clin Oncol. 2001;19:2765-7].

Ajani JA, Barthel JS, Bekaii-Saab T, et al. Gastric cancer. J Natl Compr Canc Netw. 2010 Apr;8(4):378-409.

Al-Batran S-E, Homann N, Pauligk C, Goetze TO, Meiler J, Kasper S, et al. Perioperative chemotherapy with fluorouracil plus leucovorin, oxaliplatin, and docetaxel versus fluorouracil or capecitabine plus cisplatin and epirubicin for locally advanced, resectable gastric or gastro-oesophageal junction adenocarcinoma (FLOT4): a randomized, phase 2/3 trial. Lancet. 2019;393:1948-57.

Amiri M, Janssen F, Kunst AE. The decline in stomach cancer mortality: exploration of future trends in seven European countries. Eur J Epidermiol. 2011;26:23-28.

Bang Y, Chung H, Xu J, Lordick F, Sawaki A, Al-Sakaff N, et al. Pathological features of advanced gastric cancer (GC): Relationship to human epidermal growth factor receptor 2 (HER2) positivity in the global screening programme of the ToGA trial. J Clin Oncol. 2009;27(155):4556.

Bang YJ, Van Cutsem E, Feyereislova A, Chung HC, Shen L, Sawaki A. Trastuzumab in combination with chemotherapy versus chemotherapy alone for treatment of HER2-positive advanced gastric or gastro-oesophageal junction cancer (ToGA): a phase 3, open-label, randomised controlled trial. Lancet. 2010;376:687-97.

Bang YJ, Van Cutsem E, Fuchs CS, Ohtsu A, Tabernero J, Ilson DH, et al. KEYNOTE-585: Phase III study of perioperative chemotherapy with or without pembrolizumab for gastric cancer. Future Oncol. 2019;15:943-52.

CYRAMZA Prescribing Information. Indianapolis, IN: Eli Lilly and Company; 2017.

Fuchs CS, Doi T, Jang RW, Muro K, Satoh T, Machado M, et al. Safety and efficacy of pembrolizumab monotherapy in patients with previously treated advanced gastric and gastroesophageal junction cancer phase 2 clinical KEYNOTE-059 trial. JAMA Oncol. 2018;4(5):e180013. doi:10.1001/jamaoncol.2018.0013.

Fuchs CS, Tomasek J, Yong CJ, Dumitru F, Passalacqua R, Goswami C, et al. Ramucirumab monotherapy for previously treated advanced gastric or gastro-oesophageal junction adenocarcinoma (REGARD): an international, randomised, multicentre, placebo-controlled, phase 3 trial. Lancet. 2014;383:31-39.

Gunzel D, Yu SL. Claudins of the modulation of tight junction permeability. Physiol. Rev. 2013 Apr;93(2):525-569.

HERCEPTIN (trastuzumab) [package insert]. South San Francisco, CA: Genentech, Inc.; 2016.

Karanjawala ZE, Illei PB, Ashfaq R, Infante JR, Murphy K, Pandey A, et al. New markers of pancreatic cancer identified through differential gene expression analyses: claudin 18 and annexin A8. Am J Surg Pathol. 2008;32:188-196.

KEYTRUDA® (pembrolizumab) injection, for intravenous use (Prescribing Information). Whitehouse Station, New Jersey: Merck & Co., Inc.; January 2020.

Kim NK, Park YS, Heo DS, et al. A phase III randomized study of 5-fluorouracil and cisplatin versus 5-fluorouracil, doxorubicin, and mitomycin C versus 5-fluorouracil alone in the treatment of advanced gastric cancer. Cancer. 1993;71:3813-8.

Lederman L. Gastric Cancer: local and global burden. Supplement: the current and future management of gastric cancer. Am J Manag Care. June 29, 2017:2-5.

Lee JH, Kim KS, Kim TJ, Hong SP, Song SY, Chung JB, et al. Immunohistochemical analysis of claudin expression in pancreatic cystic tumors. Oncol Rep. 2011;25:971-978.

Liu S, Yuan Y. Bayesian optimal interval designs for phase I clinical trials. J R Stat Soc Ser C (Appl Stat). 2015;64:507-23.

Moehler M, Shitara K, Garrido M, Salman P, Shen L, Wyrwicz L, et al. LBA6_PR Nivolumab (nivo) plus chemotherapy (chemo) versus chemo as first-line (1L) treatment for advanced gastric cancer/gastroesophageal junction cancer (GC/GEJC)/esophageal adenocarcinoma (EAC): First results of the CheckMate 649 study. Ann Oncol. 2020; 31.

National Comprehensive Cancer Network (NCCN) Clinical Practice Guidelines in Oncology (NCCN Guidelines): Gastric cancer. National Comprehensive Cancer Network (NCCN). 2017; version 1.

Niimi T, Nagashima K, Ward JM, Minoo P, Zimonjic DB, Popescu NC, et al. claudin-18, a novel downstream target gene for the T/EBP/NKX2.1 homeodomain transcription factor, encodes lung- and stomach-specific isoforms through alternative splicing. Mol Cell Biol. 2001;21(21):7380-7390.

Ohtsu A, Shimada Y, Shirao K, et al. Randomized phase III trial of fluorouracil alone versus fluorouracil plus cisplatin versus uracil and tegafur plus mitomycin in patients with unresectable, advanced gastric cancer: The Japan Clinical Oncology Group Study (JCOG9205). J Clin Oncol. 2003;21:54-9.

Ohtsu A, Shah MA, Van Cutsem E, Rha SY, Sawaki A, Park SR, et al. Bevacizumab in combination with chemotherapy as first-line therapy in advanced gastric cancer: a randomized, double-blind, placebo-controlled phase III study. J Clin Oncol. 2011;29:3968-3976.

OPDIVO (nivolumab) injection, for intravenous use (Prescribing Information). Princeton, New Jersey: Bristol-Myers Squibb Company; June 2020.

Pasini F, Fraccon AP, De Manzoni G. The role of chemotherapy in metastatic gastric cancer. Cancer Res. 2011;31:3543-3554.

Pennathur A, Gibson MK, Jobe BA, Luketich JD. Oesophageal carcinoma. Lance.t 2013;381:400-12.

Sahin U, Koslowski M, Dhaene K, Usener D, Brandenburg G, Seitz G, et al. Claudin-18 splice variant 2 is a pan-cancer target suitable for therapeutic antibody development. Clin Cancer Res. 2008;14(23):7624-34.

Sanada Y, Hirose Y, Osada S, Tanaka Y, Takahashi T, Yamaguchi K, et al. Immunohistochemical study of claudin 18 involvement in intestinal differentiation during the progression of intraductal papillary mucinous neoplasm. Anticancer Res. 2010;30:2995-3003.

Waddell T, Chau I, Cunningham D, Gonzalez D, Okines AF, Wotherspoon A, et al. Epirubicin, oxaliplatin, and capecitabine with or without panitumumab for patients with previously untreated advanced oesophagogastric cancer (REAL3): a randomized, open-label phase 3 trial. Lancet Oncol. 2013;14(6)481-9.

Wilke H, Muro K, Van Cutsem E, Oh SC, Bodoky G, Shimada Y, et al. Ramucirumab plus paclitaxel versus placebo plus paclitaxel in patients with previously treated advanced gastric or gastro-oesophageal junction adenocarcinoma (RAINBOW): a double-blind, randomised phase 3 trial. Lancet Oncol. 2014;15:1224-35.

Woll S, Schlitter AM, Dhaene K, Roller M, Esposito I, Sahin U, et al. Claudin 18.2 is a target for IMAB362 antibody in pancreatic neoplasms. Int. J. Cancer. 2014;134:731-739.

World Health Organization. Cancer Fact Sheet. 12 Sep 2018 [Internet]. Available from: http://www.who.int/mediacentre/factsheets/fs297/en/ [Accessed 10 Mar 2020].

12 APPENDICES

12.1 List of Excluded Concomitant Medications

Concomitant Medication Restrictions or Requirements:

<u>Prohibited</u> Concomitant Medications - The following are strictly prohibited:

- Sorivudine or analogs (during 5-FU treatment period)
- Systemic immunosuppressive agents.
    - Concurrent systemic immunosuppressive therapy, (including systemic corticosteroids) should be stopped 2 weeks prior to first dose of study drug.
    - Subjects are allowed to use a physiologic replacement dose of hydrocortisone or its equivalent (defined as up to 30 mg per day of hydrocortisone or up to 10 mg per day of prednisone) or a single dose of systemic corticosteroids.
- Other systemic chemotherapy, immunotherapy or other medications intended for antitumor activity.
- Investigational products or therapy other than zolbetuximab.
- Live vaccines should be avoided during the treatment period in which the subject is receiving oxaliplatin or 5-FU and up to 6 months after the final oxaliplatin or 5-FU dose. In cases where a live vaccine is needed for COVID-19 prevention and allowed per local regulations, please contact the Medical Monitor for discussion.

<u>Cautionary</u> Concomitant Medications:

Considerations should be given to avoid or minimize the use of the following concomitant medications, if possible, during zolbetuximab administration:

- Systemic corticosteroids, because of their impact on the potential efficacy of zolbetuximab, pembrolizumab, and nivolumab is not known.
- To minimize the risk of Torsades de Pointes, administer 5-HT3 receptor blockers with caution to subjects who have or may develop corrected QT (QTc) prolongation.
- Nonsteroidal anti-inflammatory drugs (NSAIDs), because of the potential to cause gastric ulcers.
    - In such cases where NSAID use is necessary, the use of NSAIDs with lower gastric ulcerogenic potency is preferred and efficient gastric protection with proton pump inhibitors is warranted.
    - Special attention should be paid to hemoglobin levels to identify covert bleeding.

The following should be avoided or used with caution during <u>5-FU</u> treatment and appropriate monitoring should be conducted:

- CYP2C9 substrates
- Metronidazole and cimetidine
- Anti-epileptic medications (e.g., phenobarbital, phenytoin and primidone)

The following should be avoided or used with caution during <u>oxaliplatin</u> treatment and appropriate monitoring should be conducted:

- Medications known to prolong the QT or QTc interval (refer to https://www.crediblemeds.org for a list of these medications)

The following should be avoided or used with caution during <u>docetaxel</u> treatment and appropriate monitoring should be conducted:

- Strong inhibitors or inducers of CYP3A4

12.2 Liver Safety Monitoring and Assessment

Any subject enrolled in a clinical study with active drug therapy and reveals an increase of serum aminotransferases to > 3 × ULN or bilirubin > 2 × ULN should undergo detailed testing for liver enzymes (including at least ALT, AST, alkaline phosphatase [ALP] and TBL). Testing should be repeated within 72 hours of notification of the test results. For studies for which a central laboratory is used, alerts will be generated by the central laboratory regarding moderate and severe liver abnormality to inform the investigator, study monitor and study team. Subjects should be asked if they have any symptoms suggestive of hepatobiliary dysfunction.

Definition of Liver Abnormalities

Confirmed abnormalities will be characterized as moderate and severe where ULN:

	**ALT or AST**		**Total Bilirubin**
**Moderate**	> 3 × ULN	or	> 2 × ULN
**Severe**	> 3 × ULN	and	> 2 × ULN

In addition, the subject should be considered to have severe hepatic abnormalities for any of the following:

- ALT or AST > 8 × ULN.
- ALT or AST > 5 × ULN for more than 2 weeks.
- ALT or AST > 3 × ULN and INR > 1.5 (If INR testing is applicable/evaluated).
- ALT or AST > 3 × ULN with the appearance of fatigue, nausea, vomiting, right upper quadrant pain or tenderness, fever, rash and/or eosinophilia (> 5%).

The investigator may determine that abnormal liver function results, other than as described above, may qualify as moderate or severe abnormalities and require additional monitoring and follow-up.

Follow-up Procedures

Confirmed moderate and severe abnormalities in hepatic functions should be thoroughly characterized by obtaining appropriate expert consultations, detailed pertinent history, physical examination and laboratory tests. The site should complete the liver abnormality case report form (LA-CRF) that has been developed globally and can be activated for any study or an appropriate document. Subjects with confirmed abnormal liver function testing should be followed as described below.

Confirmed moderately abnormal LFTs should be repeated 2 to 3 times weekly then weekly or less if abnormalities stabilize or the study drug has been discontinued and the subject is asymptomatic.

Severe hepatic liver function abnormalities as defined above, in the absence of another etiology, may be considered an important medical event and may be reported as a SAE. The

sponsor should be contacted and informed of all subjects for whom severe hepatic liver function abnormalities possibly attributable to study drug are observed.

To further assess abnormal hepatic laboratory findings, the investigator is expected to:

- Obtain a more detailed history of symptoms and prior or concurrent diseases. Symptoms and new-onset diseases is to be recorded as “AEs” within the (e)CRF. Illnesses and conditions such as hypotensive events, and decompensated cardiac disease that may lead to secondary liver abnormalities should be noted. Nonalcoholic steatohepatitis is seen in obese hyperlipoproteinemic and/or diabetic subjects, and may be associated with fluctuating AT levels. The investigator should ensure that the medical history form captures any illness that predates study enrollment that may be relevant in assessing hepatic function.
- Obtain a history of concomitant drug use (including nonprescription medication, complementary and alternative medications), alcohol use, recreational drug use and special diets. Medications, is to be entered in the (e)CRF. Information on alcohol, other substance use and diet should be entered on the LA-CRF or an appropriate document.
- Obtain a history of exposure to environmental chemical agents.
- Based on the subject’s history, other testing may be appropriate including:
  - Acute viral hepatitis (A, B, C, D, E or other infectious agents),
  - Ultrasound or other imaging to assess biliary tract disease,
  - Other laboratory tests including INR, direct bilirubin.
- Consider gastroenterology or hepatology consultations.
- Submit results for any additional testing and possible etiology on the LA-CRF or an appropriate document.

Study Discontinuation

In the absence of an explanation for increased LFT’s, such as viral hepatitis, preexisting or acute liver disease or exposure to other agents associated with liver injury, the subject may be discontinued from the study. The investigator may determine that it is not in the subject’s best interest to continue study enrollment. Discontinuation of treatment should be considered if:

- ALT or AST > 8 × ULN.
- ALT or AST > 5 × ULN for more than 2 weeks.
- ALT or AST > 3 × ULN and TBL > 2 × ULN or INR > 1.5) (If INR testing is applicable/evaluated).
- ALT or AST > 3 × ULN with the appearance of fatigue, nausea, vomiting, right upper quadrant pain or tenderness, fever, rash and/or eosinophilia (> 5%).

In addition, if close monitoring for a subject with moderate or severe hepatic laboratory tests is not possible, drug should be discontinued.

*Hy’s Law Definition: Drug-induced jaundice caused by hepatocellular injury, without a significant obstructive component, has a high rate of bad outcomes, from 10% to 50% mortality (or transplant). The 2 “requirements” for Hy’s Law are: 1) Evidence that a drug can

cause hepatocellular-type injury, generally shown by an increase in transaminase elevations higher 3 × ULN (2 × ULN elevations are too common in treated and untreated subjects to be discriminating). 2) Cases of increased bilirubin (at least 2 × ULN) with concurrent transaminase elevations at least 3 × ULN and no evidence of intra- or extra-hepatic bilirubin obstruction (elevated ALP) or Gilbert's syndrome [Temple, 2006].

References

Temple R. Hy's law: Predicting Serious Hepatotoxicity. Pharmacoepidemiol Drug Saf. 2006 April;15(Suppl 4):241-3.

Guidance for Industry titled "Drug-Induced Liver Injury: Premarketing Clinical Evaluation" issued by FDA on July 2009.

12.3 Common Serious Adverse Events

The following is a list of SAEs that the sponsor considers to be associated with the disease state being studied. The list does NOT change your reporting obligations or prevent the need to report an AE meeting the definition of an SAE as detailed in [Section 5.5.2 Definition of Serious Adverse Events]. The purpose of this list is to alert the investigator that some events reported as SAEs may not require expedited reporting to the regulatory authorities based on the classification of "common SAEs". The investigator is required to follow the requirements detailed in [5.5.5 Reporting of Serious Adverse Events].

For IND safety reporting, single occurrences of the following events may be excluded from expedited reporting to the FDA. If aggregate analysis of these events indicates they occur more frequently with study drug, an expedited IND safety report may be submitted to the FDA.

<u>AEs most likely related to gastric or GE adenocarcinoma:</u>

- Gastric Reflux
- Abdominal pain
- Abdominal fullness or ascites
- Dysphagia or difficulty swallowing
- Loss of appetite

12.4 Eastern Cooperative Oncology Group (ECOG) Performance Status

Grade	ECOG
0	Fully active, able to carry on all pre-disease performance without restriction
1	Restricted in physically strenuous activity but ambulatory and able to carry out work of a light or sedentary nature, e.g., light house work, office work
2	Ambulatory and capable of all self-care but unable to carry out any work activities. Up and about more than 50% of waking hours
3	Capable of only limited self-care, confined to bed or chair more than 50% of waking hours
4	Completely disabled. Cannot carry on any self-care. Totally confined to bed or chair
5	Dead

Oken MM, Creech RH, Tormey DC, Horton J, Davis TE, McFadden ET, Carbone PP. Toxicity and Response Criteria of the Eastern Cooperative Oncology Group. Am J Clin Oncol 5:649-655, 1982.

Credit to Eastern Cooperative Oncology Group, Robert Comis MD, Group Chair.

12.5 Retrospective PGx Sub-Study (Optional)

INTRODUCTION

PGx research aims to provide information regarding how naturally occurring changes in a subject’s gene and/or expression based on genetic variation may impact what treatment options are best suited for the subject. Through investigation of PGx by technologies such as genotyping, gene sequencing, statistical genetics and Genome-Wide Association Studies, the relationship between gene profiles and a drug’s kinetics, efficacy or toxicity may be better understood. As many diseases may be influenced by 1 or more genetic variations, PGx research may identify which genes are involved in determining the way a subject may or may not respond to a drug.

OBJECTIVES

The PGx research that may be conducted in the future with acquired blood samples is exploratory. The objective of this research will be to analyze or determine genes of relevance to clinical response, pharmacokinetics and toxicity/safety issues.

By analyzing genetic variations, it may be possible to predict an individual subject’s response to treatment in terms of efficacy and/or toxicity.

SUBJECT PARTICIPATION

Subjects who have consented to participate in this study may participate in this PGx sub-study. As part of this sub-study, subjects must provide written consent prior to providing any blood samples that may be used at a later time for genetic analysis.

SAMPLE COLLECTION AND STORAGE

Subjects who consent to participate in this sub-study will provide 1 approximately 5-mL tube of whole blood per Astellas’ instructions. Each sample will be identified by the unique subject number (first code). Samples will be shipped frozen to a designated banking CRO either directly from facility site or via a central laboratory as directed by Astellas.

PGx ANALYSIS

Details on the potential PGx analysis cannot be established yet. Astellas may initiate the PGx analysis in case evidence suggests that genetic variants may be influencing the drug’s kinetics, efficacy and/or safety.

DISPOSAL OF PGx SAMPLES / DATA

All PGx samples collected will be stored for a period of up to 15 years following study database hardlock. If there is no requirement for analysis, the whole blood sample will be destroyed after the planned storage period. The subject has the right to withdraw consent at any time. When a subject’s withdraw notification is received, the PGx sample will be destroyed. The results of any PGx analysis conducted on a sample prior to its withdrawal will be retained at Astellas indefinitely.

INFORMATION DISCLOSURE TO THE SUBJECTS

Exploratory PGx analysis may be conducted following the conclusion of the clinical study, if applicable. The results of the genetic analysis will not be provided to any investigators or subjects, nor can the results be requested at a later date. Any information that is obtained from the PGx analysis will be the property of Astellas.

12.6 Contraception Requirements

WOCBP participants who choose complete abstinence must continue to have pregnancy tests, as specified in Schedule of Assessments.

WOMEN OF CHILDBEARING POTENTIAL DEFINITIONS AND METHODS OF CONTRACEPTION DEFINITIONS (WOCBP)

A woman is considered fertile following menarche and until becoming post-menopausal unless permanently sterile.

Women in the following categories are not considered WOCBP

- Premenarchal
- Premenopausal female with 1 of the following:
  - Documented hysterectomy
  - Documented bilateral salpingectomy
  - Documented bilateral oophorectomy
- Post-menopausal

Documentation of any of these categories can come from the site personnel’s review of the female subject’s medical records, medical examination, or medical history interview.

A postmenopausal state is defined as at least 12 months after last regular menstrual bleeding without an alternative medical cause.

- In case the last regular menstrual bleeding cannot be clearly determined, confirmation with repeated FSH measurements of at least > 40 IU/L (or higher per local institutional guidelines), is required.

Females on HRT and whose menopausal status is in doubt will be required to use one of the non-estrogen hormonal highly effective contraception methods if they wish to continue their HRT during the study. Otherwise, they must discontinue HRT to allow confirmation of postmenopausal status by repeated FSH measurements before study enrollment.

CONTRACEPTION GUIDANCE FOR FEMALE PARTICIPANTS OF CHILDBEARING POTENTIAL

One of the highly effective methods of contraception listed below is required at the time of informed consent and until the end of relevant systemic exposure, defined as 9 months after the final oxaliplatin administration and 6 months after the final administration of all other study drugs.†

Combined (estrogen- and progestogen-containing) hormonal contraception associated with inhibition of ovulation‡

- oral
- intravaginal
- transdermal

Progestogen-only hormonal contraception associated with inhibition of ovulation

- oral
- injectable
- implantable

Hormonal methods of contraception containing a combination of estrogen and progesterone, vaginal ring, injectables, implants and intrauterine hormone-releasing system (IUS)

- intrauterine device (IUD)
- bilateral tubal occlusion

Vasectomized partner *(A vasectomized partner is a highly effective contraception method provided that the partner is the sole male sexual partner of the WOCBP and the absence of sperm has been confirmed. If not, an additional highly effective method of contraception should be used.)*

Sexual abstinence *Sexual abstinence is considered a highly effective method only if defined as refraining from heterosexual intercourse during the entire period of risk associated with the study drug. The reliability of sexual abstinence needs to be evaluated in relation to the duration of the study and the preferred and usual lifestyle of the participant. It is not necessary to use any other method of contraception when complete abstinence is elected.*

† Local laws and regulations may require use of alternative and/or additional contraception methods.

‡ Typical use failure rates may differ from those when used consistently and correctly. Use should be consistent with local regulations regarding the use of contraceptive methods for participants participating in clinical studies.

CONTRACEPTION GUIDANCE FOR MALE PARTICIPANTS WITH PARTNER(S) OF CHILDBEARING POTENTIAL.

Male participants with female partners of childbearing potential are eligible to participate if they agree to the following during treatment and until the end of relevant systemic exposure defined as 6 months after final drug administration.†

- Inform any and all partner(s) of their participation in a clinical drug study and the need to comply with contraception instructions as directed by the investigator.
- Male participants are required to use a condom during treatment and until end of relevant systemic exposure defined as 6 months after final drug administration.
- Female partners of male participants who have not undergone a vasectomy with the absence of sperm confirmed or a bilateral orchiectomy should consider use of effective methods of contraception until the end of relevant systemic exposure, defined as 6 months after final drug administration.

† Local laws and regulations may require use of alternative and/or additional contraception methods.

12.7 Clinical Study Continuity

INTRODUCTION

The purpose of this appendix is to provide acceptable alternate methods to assess safety and efficacy parameters, as appropriate, in the event the clinical study is interrupted at the country, state, site or participant level during any crisis (e.g., natural disaster, pandemic).

BENEFIT-RISK RATIONALE

Maintaining the safety of clinical study participants and delivering continuity of care in the clinical study setting is paramount during any crisis. The site is expected to follow the protocol and associated Schedule of Assessments [Table 1], [Table 3], [Table 4], [Table 7], [Table 9] or [Table 11] for Cohorts 1, 2 (cycle 1), 2 (cycles ≥ 2), 3A, 4 or 5, respectively, unless the site PI discusses the need with the Astellas Medical Monitor to implement the alternate measures.

The approach outlined within this appendix defines which assessments are required to maintain a favorable benefit/risk to the participant, to maintain overall study integrity and to provide acceptable alternate methods to complete the study required assessments and procedures if study activities are unable to be performed as described in [Section VI] due to a crisis.

INFORMED CONSENT

Participants who need to follow any or all of the alternate measures outlined in this Appendix will be required to provide informed consent which explicitly informs them of the nature of, and rationale for these changes, and gain their agreement to continue participation in the study prior to the implementation of any of these changes. In the event the urgency of implementing the alternate measures does not allow for the participant to provide written consent prior to implementation, the PI or designee will obtain oral agreement from the subject followed by written documentation as soon as is feasible. A separate addendum to the study informed consent will be provided to document the participant’s consent of the changes.

PARTICIPANT PROCEDURES ASSESSMENT

Sites with participants who are currently enrolled into this clinical study may consider implementing the alternate methods outlined below if 1 or more of the following conditions are met due to the crisis:

- Regional or local travel has been restricted, inclusive of mandatory shelter in place measures, which makes participant travel to/from the study site nearly impossible
- Site facilities have been closed for clinical study conduct
- Site has been restricted to treating patients with conditions outside of the scope of the study
- Site personnel have temporarily relocated the conduct of the study to a location that place a burden on the participant with respect to time and travel

- Participant(s) have temporarily relocated from the current study site to an alternate study site to avoid placing a burden on the participant with respect to travel
- Participant(s) have temporarily relocated from their home location and the new distances from the site would cause undue burden with respect to time and travel
- Participant has risk factors for which traveling to the site poses an additional risk to the participant's health and safety

Adherence to the original protocol as reflected in the Schedule of Assessments [Table 4], [Table 9] and [Table 11] is expected, where plausible, in the case of a crisis. The alternate measures as noted in [Table 36], [Table 37] and [Table 38] below are only permissible in the event of a crisis, and after discussing the need with the Astellas Medical Monitor to implement the alternate measures. This is to allow for continuity of receiving IMP and maintaining critical safety and efficacy assessments for patients participating in the study at a time of crisis.

If 1 or more of the alternate measures noted below is implemented for a participant, the site should document in the participant's source document the justification for implementing the alternate measure and the actual alternate measures that were implemented, along with the corresponding time point(s).

Note: At the time this appendix was added to the protocol, all subjects in Cohorts 1A and 3A had discontinued from the study. Alternate approaches are described for Cohorts 2, 4 and 5 only.

Table 36 Alternative Schedule of Assessments in Response to a Crisis, Cohort 2 Cycle ≥ 2

Critical Assessment	Alternate Approach(es)	Critical Timepoint: Treatment Period (cycle = 42 days): Cycles 2 to 4 Combination Treatment Period mFOLFOX6 + Zolbetuximab				Cycles ≥ 5 Combination Treatment Period Zolbetuximab + Leucovorin + 5-FU				Follow-up: Zolbetuximab Study Drug Discontinuation Visit[20]	mFOLFOX6 Study Drug Discontinuation Visit[20]	Zolbetuximab 30-Day Safety Follow-up Visit[21]	mFOLFOX6 30-Day Safety Follow-up Phone Call[22]	Zolbetuximab 90-Day Follow-up Visit[23]	mFOLFOX6 90-Day Safety Follow-up Phone Call[24]	Post-treatment Follow-up[25]
**Day**		**1**	**15**	**22**	**29**	**1**	**15**	**22**	**29**							
**Study Drug Administration**																
Antiemetic Pretreatment[1]	Oral antiemetics can be administered at home as per SoC	X	X	X	X	X	X	X	X							
Zolbetuximab [2]	Window of -2 days acceptable	X		X		X		X								

Table continued on next page

Critical Assessment	Alternate Approach(es)	Critical Timepoint								Follow-up						
		Treatment Period (cycle = 42 days)								Zolbetuximab Study Drug Discontinuation Visit[20]	mFOLFOX6 Study Drug Discontinuation Visit[20]	Zolbetuximab 30-Day Safety Follow-up Visit[21]	mFOLFOX6 30-Day Safety Follow-up Phone Call[22]	Zolbetuximab 90-Day Follow-up Visit[23]	mFOLFOX6 90-Day Safety Follow-up Phone Call[24]	Post-treatment Follow-up[25]
		Cycles 2 to 4<br><br>Combination Treatment Period mFOLFOX6 + Zolbetuximab				Cycles ≥ 5<br><br>Combination Treatment Period Zolbetuximab + Leucovorin + 5-FU										
**Day**		**1**	**15**	**22**	**29**	**1**	**15**	**22**	**29**							
Post-Infusion Observation Period[3]	Decrease of initial observation period to 1 hour and subsequent observation period to 30 minutes is acceptable if there are no AEs of > grade 2	X		X		X		X								
mFOLFOX6 [4]	Window of -2 days acceptable<br>Patient may receive SoC (mFOLFOX6) treatment at a local site	X	X		X	X	X		X							
**Physical Examination/Assessments**																
Physical Examination[5]	Protocol allows for targeted exam after cycle 1, day 1.<br>If Physical exam is not completed at Study Treatment Discontinuation Visit and 30-Day Safety Follow-up Visit this is acceptable.	X		X		X		X								

Table continued on next page

Critical Assessment	Alternate Approach(es)	Critical Timepoint								Follow-up						
		Treatment Period (cycle = 42 days)														
		Cycles 2 to 4<br><br>Combination Treatment Period mFOLFOX6 + Zolbetuximab				Cycles ≥ 5<br><br>Combination Treatment Period Zolbetuximab + Leucovorin + 5-FU				Zolbetuximab Study Drug Discontinuation Visit[20]	mFOLFOX6 Study Drug Discontinuation Visit[20]	Zolbetuximab 30-Day Safety Follow-up Visit[21]	mFOLFOX6 30-Day Safety Follow-up Phone Call[22]	Zolbetuximab 90-Day Follow-up Visit[23]	mFOLFOX6 90-Day Safety Follow-up Phone Call[24]	Post-treatment Follow-up[25]
**Day**		**1**	**15**	**22**	**29**	**1**	**15**	**22**	**29**							
Weight [5]	If there are no associated active AEs, acceptable if weight is not done at Study Treatment Discontinuation Visit and 30-Day Safety Follow-up Visit.	X		X		X		X								
ECOG Performance Status[5]	Not required at Study Treatment Discontinuation visit or 30 Day Follow up Visit;<br>ECOG Performance Status may be assessed and captured via phone contact	X		X		X		X								

Table continued on next page

Critical Assessment	Alternate Approach(es)	Critical Timepoint								Follow-up						
		Treatment Period (cycle = 42 days)								Zolbetuximab Study Drug Discontinuation Visit[20]	mFOLFOX6 Study Drug Discontinuation Visit[20]	Zolbetuximab 30-Day Safety Follow-up Visit[21]	mFOLFOX6 30-Day Safety Follow-up Phone Call[22]	Zolbetuximab 90-Day Follow-up Visit[23]	mFOLFOX6 90-Day Safety Follow-up Phone Call[24]	Post-treatment Follow-up[25]
		Cycles 2 to 4<br><br>Combination Treatment Period mFOLFOX6 + Zolbetuximab				Cycles ≥ 5<br><br>Combination Treatment Period Zolbetuximab + Leucovorin + 5-FU										
**Day**		**1**	**15**	**22**	**29**	**1**	**15**	**22**	**29**							
Vital Signs [6]	If Day 15 and Day 29 mFOLOFX6 dosing is administered at local facility, SoC can be applied<br>If there are no associated active AEs, missed assessments at Study Treatment Discontinuation Visit and 30-Day Safety Follow-up Visit are acceptable; Vital Sign collection frequency during post observation period can be decreased	X		X		X		X								
**Laboratory Assessments**																
Biochemistry[7]	Sample may be collected up to 4 days prior to treatment visit after cycle 1, day 1;<br>Collection of samples during study treatment at local facility acceptable if results can be made available to investigative site.	X	X	X	X	X	X	X	X							

Table continued on next page

**Critical Assessment**	**Alternate Approach(es)**	**Critical Timepoint**								**Follow-up**						
		**Treatment Period (cycle = 42 days)**								**Zolbetuximab Study Drug Discontinuation Visit[20]**	**mFOLFOX6 Study Drug Discontinuation Visit[20]**	**Zolbetuximab 30-Day Safety Follow-up Visit[21]**	**mFOLFOX6 30-Day Safety Follow-up Phone Call[22]**	**Zolbetuximab 90-Day Follow-up Visit[23]**	**mFOLFOX6 90-Day Safety Follow-up Phone Call[24]**	**Post-treatment Follow-up[25]**
		**Cycles 2 to 4**<br><br>**Combination Treatment Period mFOLFOX6 + Zolbetuximab**				**Cycles ≥ 5**<br><br>**Combination Treatment Period Zolbetuximab + Leucovorin + 5-FU**										
**Day**		**1**	**15**	**22**	**29**	**1**	**15**	**22**	**29**							
Hematology[7]	Sample may be collected up to 4 days prior to treatment visit after cycle 1, day 1;<br>Collection of samples at local facility acceptable if results can be made available to investigative site	X	X	X	X	X	X	X	X	X	X	X				
Urinalysis[8]	Sample may be collected up to 4 days prior to treatment visit; collection at local facility also allowed.	If clinically indicated								X	X	X				
Cytokine/Chemokine and/or Tryptase[9]	Window of -2 days acceptable.	X		X		X		X								
TSH and T4	If this testing is unable to be performed, this is acceptable.	If clinically indicated														
PT, PTT and INR[10]	None as protocol allows standard of care if clinically indicated. If the subject is not on a concomitant medication that affects these parameters, acceptable if these are not done.	If clinically indicated														

Table continued on next page

**Critical Assessment**	**Alternate Approach(es)**	**Critical Timepoint**								**Follow-up**						
		**Treatment Period (cycle = 42 days)**								**Zolbetuximab Study Drug Discontinuation Visit[20]**	**mFOLFOX6 Study Drug Discontinuation Visit[20]**	**Zolbetuximab 30-Day Safety Follow-up Visit[21]**	**mFOLFOX6 30-Day Safety Follow-up Phone Call[22]**	**Zolbetuximab 90-Day Follow-up Visit[23]**	**mFOLFOX6 90-Day Safety Follow-up Phone Call[24]**	**Post-treatment Follow-up[25]**
		**Cycles 2 to 4**<br><br>**Combination Treatment Period mFOLFOX6 + Zolbetuximab**				**Cycles ≥ 5**<br><br>**Combination Treatment Period Zolbetuximab + Leucovorin + 5-FU**										
**Day**		**1**	**15**	**22**	**29**	**1**	**15**	**22**	**29**							
Urine Pregnancy Test[11]	Collection at local facility also allowed if results can be made available to investigative site. Sample may be collected up to 4 days prior to treatment visit C1D1.	X		X		X		X		X	X	X				
Pharmacokinetics of Zolbetuximab	If subject is dosed or visits clinic, PK samples should be collected. Samples cannot be collected at local facility. If central labs cannot receive sample shipments, samples can be stored at sites in -70°C degree freezer until shipping is acceptable again.	See Table 5 for pharmacokinetic collection schedule										X		X		

Table continued on next page

**Critical Assessment**	**Alternate Approach(es)**	**Critical Timepoint**								**Follow-up**						
		**Treatment Period (cycle = 42 days)**								**Zolbetuximab Study Drug Discontinuation Visit**[20]	**mFOLFOX6 Study Drug Discontinuation Visit**[20]	**Zolbetuximab 30-Day Safety Follow-up Visit**[21]	**mFOLFOX6 30-Day Safety Follow-up Phone Call**[22]	**Zolbetuximab 90-Day Follow-up Visit**[23]	**mFOLFOX6 90-Day Safety Follow-up Phone Call**[24]	**Post-treatment Follow-up**[25]
		**Cycles 2 to 4**<br><br>**Combination Treatment Period mFOLFOX6 + Zolbetuximab**				**Cycles ≥ 5**<br><br>**Combination Treatment Period Zolbetuximab + Leucovorin + 5-FU**										
**Day**		**1**	**15**	**22**	**29**	**1**	**15**	**22**	**29**							
Pharmacokinetics of mFOLFOX6	If subject is dosed or visits clinic, PK samples should be collected. Samples cannot be collected at local facility.<br>If central labs cannot receive sample shipments, samples can be stored at sites in -70°C until shipping is accepted again.	See Table 6 for pharmacokinetic collection schedule														
Anti-Drug Antibodies (immunogenicity)	If subject is dosed or visits clinic, ADA samples should be collected. Samples cannot be collected at local facility.<br>Sample collection prioritized if clinically indicated.<br>If central labs cannot receive sample shipments, samples can be stored at sites in -70°C degree freezer until shipping is acceptable again.	See Table 5 for sample collection schedule										X		X		

Table continued on next page

**Critical Assessment**	**Alternate Approach(es)**	**Critical Timepoint**								**Follow-up**						
		**Treatment Period (cycle = 42 days)**								**Zolbetuximab Study Drug Discontinuation Visit[20]**	**mFOLFOX6 Study Drug Discontinuation Visit[20]**	**Zolbetuximab 30-Day Safety Follow-up Visit[21]**	**mFOLFOX6 30-Day Safety Follow-up Phone Call[22]**	**Zolbetuximab 90-Day Follow-up Visit[23]**	**mFOLFOX6 90-Day Safety Follow-up Phone Call[24]**	**Post-treatment Follow-up[25]**
		**Cycles 2 to 4**<br><br>**Combination Treatment Period mFOLFOX6 + Zolbetuximab**				**Cycles ≥ 5**<br><br>**Combination Treatment Period Zolbetuximab + Leucovorin + 5-FU**										
**Day**		**1**	**15**	**22**	**29**	**1**	**15**	**22**	**29**							
Exploratory Biomarkers (Serum)[12]	Biomarker samples are collected on zolbetuximab treatment days and do not require a unique visit to the study site. If samples can be collected but central labs cannot receive samples, samples can be stored at sites until shipping is accepted again.	X		X						X						
Exploratory Biomarkers (Plasma)[12]	Biomarker samples are collected on zolbetuximab treatment days and do not require a unique visit to the study site. If samples can be collected but central labs cannot receive samples, samples can be stored at sites until shipping is accepted again.	X		X						X						

Table continued on next page

Critical Assessment	Alternate Approach(es)	Critical Timepoint								Follow-up						
		Treatment Period (cycle = 42 days)								Zolbetuximab Study Drug Discontinuation Visit[20]	mFOLFOX6 Study Drug Discontinuation Visit[20]	Zolbetuximab 30-Day Safety Follow-up Visit[21]	mFOLFOX6 30-Day Safety Follow-up Phone Call[22]	Zolbetuximab 90-Day Follow-up Visit[23]	mFOLFOX6 90-Day Safety Follow-up Phone Call[24]	Post-treatment Follow-up[25]
		Cycles 2 to 4<br><br>Combination Treatment Period mFOLFOX6 + Zolbetuximab				Cycles ≥ 5<br><br>Combination Treatment Period Zolbetuximab + Leucovorin + 5-FU										
**Day**		**1**	**15**	**22**	**29**	**1**	**15**	**22**	**29**							
Immune Cell Subsets (Whole Blood)[12]	Biomarker samples are collected on zolbetuximab treatment days and do not require a unique visit to the study site. If samples can be collected but central labs cannot receive samples, samples can be stored at sites until shipping is accepted again.	X		X						X						
Cryopreserved PBMC[11]	Samples are collected on zolbetuximab treatment days and do not require a unique visit to the study site. If samples can be collected but central labs cannot receive samples, samples can be stored at sites until shipping is accepted again.	X		X						X						

Table continued on next page

Critical Assessment	Alternate Approach(es)	Critical Timepoint: Treatment Period (cycle = 42 days): Cycles 2 to 4 Combination Treatment Period mFOLFOX6 + Zolbetuximab				Cycles ≥ 5 Combination Treatment Period Zolbetuximab + Leucovorin + 5-FU				Follow-up: Zolbetuximab Study Drug Discontinuation Visit[20]	mFOLFOX6 Study Drug Discontinuation Visit[20]	Zolbetuximab 30-Day Safety Follow-up Visit[21]	mFOLFOX6 30-Day Safety Follow-up Phone Call[22]	Zolbetuximab 90-Day Follow-up Visit[23]	mFOLFOX6 90-Day Safety Follow-up Phone Call[24]	Post-treatment Follow-up[25]
**Day**		**1**	**15**	**22**	**29**	**1**	**15**	**22**	**29**							
**Cardiac Safety**																
12-lead ECG[13]	ECGs allowed up to 4 days prior to treatment visits after cycle 1, day 1 and can be done locally but must be reviewed prior to dosing. If oxaliplatin dosing visits on day 15 or day 29 are conducted at local facility, standard of care ECG monitoring would be acceptable; For zolbetuximab Treatment Discontinuation Visit and 30-Day Safety Follow Up Visit required only if clinically indicated.	X	X		X	If clinically indicated				X		X				

Table continued on next page

Critical Assessment	Alternate Approach(es)	Critical Timepoint								Follow-up							
		Treatment Period (cycle = 42 days)								Zolbetuximab Study Drug Discontinuation Visit[20]	mFOLFOX6 Study Drug Discontinuation Visit[20]	Zolbetuximab 30-Day Safety Follow-up Visit[21]	mFOLFOX6 30-Day Safety Follow-up Phone Call[22]	Zolbetuximab 90-Day Follow-up Visit[23]	mFOLFOX6 90-Day Safety Follow-up Phone Call[24]	Post-treatment Follow-up[25]	
		Cycles 2 to 4<br>Combination Treatment Period mFOLFOX6 + Zolbetuximab				Cycles ≥ 5<br>Combination Treatment Period Zolbetuximab + Leucovorin + 5-FU											
**Day**		1	15	22	29	1	15	22	29								
**Radiology**																	
Image Assessment[14]	At select visits, efficacy assessment using radiological examinations are required. Imaging assessment can be done locally and if available, should be submitted to central imaging vendor. Independent central reading of locally obtained scans can be facilitated by sharing of Image Acquisition Guidelines from study site to local site if applicable. Investigational site will be requested to re-read the scan performed at local site. If investigational site read is not an option, the investigator should discuss the case with the local institution radiologist.	Every 9 (± 1) weeks from C1D1 for the first 54 weeks and then every 12 (± 2) weeks thereafter.															

Table continued on next page

Critical Assessment	Alternate Approach(es)	Critical Timepoint								Follow-up						
		Treatment Period (cycle = 42 days)								Zolbetuximab Study Drug Discontinuation Visit[20]	mFOLFOX6 Study Drug Discontinuation Visit[20]	Zolbetuximab 30-Day Safety Follow-up Visit[21]	mFOLFOX6 30-Day Safety Follow-up Phone Call[22]	Zolbetuximab 90-Day Follow-up Visit[23]	mFOLFOX6 90-Day Safety Follow-up Phone Call[24]	Post-treatment Follow-up[25]
		Cycles 2 to 4<br><br>Combination Treatment Period mFOLFOX6 + Zolbetuximab				Cycles ≥ 5<br><br>Combination Treatment Period Zolbetuximab + Leucovorin + 5-FU										
**Day**		**1**	**15**	**22**	**29**	**1**	**15**	**22**	**29**							
**HRQoL**																
HRQoL[15]	Not required at Study Treatment Discontinuation visit; may be assessed and captured via phone contact; may be captured a day or 2 ahead of visit	X	X	X	X	X	X	X	X	X		X		X		
**Tissue Samples**																
Post-progression Tumor Sample (optional)[16]	Biomarker samples are collected on zolbetuximab treatment days and do not require a unique visit to the study site. If samples can be collected but central labs cannot receive samples, samples can be stored at sites until shipping is accepted again.									X						
On-Treatment Biopsy[17]	Biomarker samples are collected on zolbetuximab treatment days and do not require a unique visit to the study site. If samples can be collected but central labs cannot receive samples, samples can be stored at sites until shipping is accepted again.	X														

Table continued on next page

Critical Assessment	Alternate Approach(es)	Critical Timepoint								Follow-up						
		Treatment Period (cycle = 42 days)								Zolbetuximab Study Drug Discontinuation Visit[20]	mFOLFOX6 Study Drug Discontinuation Visit[20]	Zolbetuximab 30-Day Safety Follow-up Visit[21]	mFOLFOX6 30-Day Safety Follow-up Phone Call[22]	Zolbetuximab 90-Day Follow-up Visit[23]	mFOLFOX6 90-Day Safety Follow-up Phone Call[24]	Post-treatment Follow-up[25]
		Cycles 2 to 4<br>Combination Treatment Period mFOLFOX6 + Zolbetuximab				Cycles ≥ 5<br>Combination Treatment Period Zolbetuximab + Leucovorin + 5-FU										
**Day**		1	15	22	29	1	15	22	29							
**Safety Assessment**																
Concomitant Medication[18]	Remote/Virtual/Telemedicine Visits allowed for non-dosing visits. Please refer to protocol schedule of assessments.	X	X	X	X	X	X	X	X	X	X	X	X	X	X	
AEs/SAEs[19]	Remote/Virtual/Telemedicine Visits allowed for non-dosing visits. Please refer to protocol schedule of assessments.	X	X	X	X	X	X	X	X	X	X	X	X	X	X	

5-FU: fluorouracil; ADA: anti-drug antibody; AE: adverse event; BSA: body surface area; C: cycle; CLDN: claudin; CT: computerized tomography; D: day; eCRF: electronic case report form; ECG: electrocardiogram; ECOG: Eastern Cooperative Oncology Group; FFPE: formalin-fixed paraffin-embedded; GP: Global Pain; HER2: human epidermal growth factor receptor 2; HRQoL: health-related quality of life; HRU: Health Resource Utilization; ICF: informed consent form; INR: international normalized ratio; IRR: infusion related reaction; IV: intravenous; MRI: magnetic resonance imaging; ORR: objective response rate; PBMC: peripheral blood mononuclear cells; PET: positron emission tomography; PFS: progression-free survival; PGx: pharmacogenomics; PT: prothrombin time; PTT: partial thromboplastin time; SAE: serious adverse event; SoC: standard of care; T4: thyroxine; TSH: thyroid-stimulating hormone.

Laboratory tests will be performed predose according to the Schedule of Assessments and sent to a central laboratory for analysis. In case of multiple laboratory data within this period, the most recent data should be used.

1. Prophylactic antiemetics and, if needed, other premedications should be given according to institutional standards and the respective product package insert(s). Antiemetic premedication should be given at least 30 minutes prior to each dose of zolbetuximab and mFOLFOX6. It is recommended that the prophylactic antiemetic regimen include the following agents: NK-1 receptor blockers and 5-HT3 receptor blockers. On days when subjects receive both zolbetuximab and mFOLFOX6, antiemetic premedication will be given prior to zolbetuximab administration.
2. Zolbetuximab will be administered as a minimum 2-hour IV infusion. Zolbetuximab IV infusion may be interrupted or slowed down to manage toxicity.

Footnotes continued on next page

3. Post-Infusion Observation Period: If the subject does not develop any AEs, the subject should be observed for 1 hour post infusion for their subsequent zolbetuximab infusions. If AEs are observed, infusion time should be extended and subjects should continue to be observed for 2 hours post infusion.
4. mFOLFOX6 will be administered every 2 weeks. If both zolbetuximab and mFOLFOX6 are to be administered during the same visit, zolbetuximab should be administered prior to mFOLFOX6.
5. Physical examination and other evaluations include height (at screening only), weight and ECOG performance status. The physical exam only needs to be repeated on C1D1 if clinically significant changes from screening (in the opinion of the investigator) are observed. Target (symptom-driven) physical exams should be conducted every 3 weeks on zolbetuximab visit days.
6. Vital signs (pulse, blood pressure, temperature) should be taken at the following time points:
    a. Predose on dosing days
    b. Subsequent zolbetuximab infusions: Every 30 (± 10) minutes if the subject did not develop any AEs during the Post-Infusion Observation Period of cycle 1.
    c. Post-treatment observation period: 30 and 60 (± 10) minutes post infusion.
7. See [Section 5.4.3] for list of laboratory assessments. Laboratory tests must be sent to the central laboratory for analysis. Central laboratory results must be used to confirm eligibility. The screening labs used to determine eligibility should be collected within 14 days prior to C1D1. In situations where central laboratory results are outside of the permitted range, the investigator may opt to retest the subject and subsequent within-range screening results may be used to confirm eligibility. In case of multiple laboratory data within the Screening period, the most recent central laboratory data should be used to confirm eligibility. Laboratory test results will be reviewed by the investigator prior to any study treatment. Local laboratory results may be used for treatment decisions; however, central laboratory samples must also be drawn per protocol and sent to the central laboratory. Central and local labs may be collected up to 48 hours prior to study treatment. Holidays and weekends should be taken into account when scheduling these blood draws. Additional assessments may be done centrally or locally to monitor AEs or as clinically indicated. Clinical significance of out-of-range laboratory findings is to be determined and documented by the investigator/subinvestigator who is a qualified physician. CT scans and MRI conducted as part of a subject's routine clinical management (i.e., standard of care) obtained before signing the ICF may be utilized for screening or baseline purposes, provided the procedures met the protocol-specified criteria and were performed within the Screening period.
8. Urinalysis: Urinalysis should be performed at screening and is to be repeated if clinically indicated after starting study treatment. Urinalysis should also be performed at the zolbetuximab and mFOLFOX6 study discontinuation visits and at the zolbetuximab 30-day follow-up visit. Urinalysis tests will be sent to a central laboratory for analysis.
9. Cytokines/chemokines and/or tryptase need to be collected centrally.
10. Ongoing evaluation should be continued for subjects who are receiving therapeutic anticoagulation according to local standard of care.
11. Urine Pregnancy Test: For female subjects of childbearing potential only. Urine pregnancy tests are to be completed prior to zolbetuximab administration. Urine pregnancy tests can be confirmed at a local laboratory.
12. Biomarker (serum, plasma, whole blood and cryopreserved PBMCs) samples should be taken within 48 hours prior to dosing.
    a. C2D1: predose
    b. C2D22: predose
    c. C3D22: predose
    d. C4D22: predose
    e. Zolbetuximab study drug discontinuation visit

Footnotes continued on next page

13. Single ECG will be performed at the following time points:
    - Screening
    - Up to 48 hours prior to every oxaliplatin infusion (before any antiemetic treatment)†
    - Up to 6 hours following completion of every oxaliplatin infusion†
    - Zolbetuximab study drug discontinuation visit
    - Zolbetuximab 30-day follow-up visit
    - If clinically indicated

    †Local read only – do not transmit to central ECG laboratory
14. Imaging assessments to be collected at screening and every 9 (± 1) weeks counting from C1D1 for the first 54 weeks, and then every 12 (± 2) weeks thereafter. Imaging assessments completed prior to consent, but within 28 days of first dose of study drug, may be used for screening. Imaging will include CT scans with contrast of the thorax, abdomen, and pelvis (if CT scan is medically not feasible with contrast, MRI may be used for imaging). Bone scans (or focal X-ray) or brain imaging should be performed if metastatic disease is suspected. Disease must be evident by radiology; measurable lesions only. Same mode of imaging should be utilized throughout the study unless medical necessity requires change. CT scan performed with PET scan can be used if it is of quality, which allows accurate tumor measurements. PFS and tumor responses will be evaluated by investigator per RECIST 1.1. All imaging will be sent to a central independent radiographic review within 7 days.
15. HRQoL questionnaires and HRU are to be administered on zolbetuximab visit days before any drug treatment (or up to 48 hours prior to treatment) or other scheduled assessments are conducted and before the disease status is discussed with the subject. HRQoL will be measured by Quality of Life Questionnaire – Core Questionnaire (QLQ-C30), Oesophago-Gastric Module (OG-25), Global Pain (GP) and the EuroQOL Five Dimensions (EQ-5D) questionnaires. The HRU questionnaire will not be administered at the screening visit. Questionnaires should only be administered on days when the subject receives zolbetuximab treatment. Questionnaire completion will not be required if the subject is illiterate or the questionnaire is not available in the local language.
16. For subjects who signed a separate ICF, an optional post-progression tumor sample for exploratory biomarker analysis should be collected following confirmation of disease progression and prior to commencement of subsequent anti-cancer therapy.
17. Subjects are required to provide an on-treatment tumor specimen collected at the C2D1 visit (± 15 days). A minimum of 1 FFPE tumor tissue block (preferred) OR a minimum of 15 FFPE unstained sections are required. If slides are submitted, the slides should be freshly cut from the FFPE block within the time frame described in the laboratory manual. If available, the provision of additional fresh/frozen tumor samples is strongly encouraged. If ≥ 15 slides cannot be provided, the sponsor should be contacted for further guidance.
18. Concomitant medications will be collected from the time of main study full main informed consent through 90 days following the last dose of study drug.
19. AEs and SAEs (regardless of causality) will be collected from the time of full main informed consent through 90 days following the last dose of study drug.
20. Study Drug Discontinuation: Visit will occur within 7 days after the last dose or decision by the investigator to discontinue subject from treatment.
21. Zolbetuximab 30-day Safety Follow-up Visit should occur 30 days (± 7) after last dose of zolbetuximab.
22. mFOLFOX6 30-day Safety Follow-up phone call should occur 30 days (± 7) after last dose of mFOLFOX6 (all components).
23. Zolbetuximab 90-day Follow-up Visit should occur 90 days (+ 7) after last dose of zolbetuximab.
24. mFOLFOX6 90-day Safety Follow-up phone call should occur 90 days (+ 7) after the last dose of mFOLFOX6 (all components).
25. Post-treatment Follow-up: if a subject discontinues study drug prior to radiographic or clinical disease progression as confirmed by the independent central reader, the subject should enter the post-treatment follow-up period and continue to undergo imaging assessments until progression is documented per the investigator, or the subject starts another cancer treatment, whichever occurs earlier.

Table 37 Alternative Schedule of Assessments in Response to a Crisis, Cohort 4

Critical Assessment	Alternate Approach(es)†	Critical Timepoint						Follow-up							
		Treatment Period (1 cycle = 42 days)						Zolbetuximab Study Drug Discontinuation Visit[28]	mFOLFOX6 and/or nivolumab Study Drug Discontinuation Visit[28]	Zolbetuximab 30-Day Safety Follow-up Visit[29]	mFOLFOX6 and/or nivolumab 30-Day Safety Follow-up Visit[30]	Zolbetuximab 90-Day Follow-up Visit[31]	mFOLFOX6 and/or nivolumab 90-Day Safety Follow-up Visit[32]	Post-treatment Follow-up[33]	Survival Follow-up (Cohort 4B only)[34]
		Cycles 1 to 4<br>zolbetuximab + mFOLFOX6 + nivolumab			Cycles ≥ 5<br>zolbetuximab + leucovorin or folinic acid + 5-FU + nivolumab										
**Day**		1	15	29	1	15	29								
**Study Drug Administration**															
Antiemetic Pretreatment[6]	Can be administered at home per standard of care	X	X	X	X	X	X								
Zolbetuximab[7]	Day 1 and Day 29 administration of zolbetuximab must be dosed at the study site. Day 15 dosing can be skipped. Window of -2 days acceptable.	X	X	X	X	X	X								
Post-Infusion Observation Period[8]	Decrease of initial observation period to 1 hour and subsequent observation period to 30 min is acceptable if there are no AEs of ≥ grade 2	X	X	X	X	X	X								
Nivolumab[9]	Day 1 and Day 29 administration of nivolumab must be dosed at the study site. Day 15 dosing can be skipped. Window of -2 days acceptable	X	X	X	X	X	X								

Table continued on next page

Critical Assessment	Alternate Approach(es)†	Critical Timepoint						Follow-up							
		Treatment Period (1 cycle = 42 days)						Zolbetuximab Study Drug Discontinuation Visit[28]	mFOLFOX6 and/or nivolumab Study Drug Discontinuation Visit[28]	Zolbetuximab 30-Day Safety Follow-up Visit[29]	mFOLFOX6 and/or nivolumab 30-Day Safety Follow-up Visit[30]	Zolbetuximab 90-Day Follow-up Visit[31]	mFOLFOX6 and/or nivolumab 90-Day Safety Follow-up Visit[32]	Post-treatment Follow-up[33]	Survival Follow-up (Cohort 4B only)[34]
		Cycles 1 to 4<br>zolbetuximab + mFOLFOX6 + nivolumab			Cycles ≥ 5<br>zolbetuximab + leucovorin or folinic acid + 5-FU + nivolumab										
**Day**		**1**	**15**	**29**	**1**	**15**	**29**								
mFOLFOX6[10]	No change in Day 1 administration; Day 15 and Day 29 visits can be conducted locally per standard of care by oncology qualified personnel if zolbetuximab dosing visits continue at the investigative site as planned on at least Day 1 of each cycle and dosing records can be obtained from the treating facility	X	X	X	X	X	X								
**Physical Examination/Assessments**															
Physical Examination[11]	Protocol allows for targeted exam after C1D1. If physical exam is not able to be completed after C1D1 that is acceptable.	X	X	X	X	X	X								
Weight[11]	If there are no associated active AEs, acceptable if weight is not done at Study Treatment Discontinuation Visit and 30-day Safety Follow-up Visit.	X	X	X	X	X	X								
ECOG Performance Status[11]	Not required at Study Treatment Discontinuation visit; may be assessed and captured via phone contact.	X	X	X	X	X	X								

Table continued on next page

**Critical Assessment**	**Alternate Approach(es)†**	**Critical Timepoint**						**Follow-up**							
		**Treatment Period (1 cycle = 42 days)**						**Zolbetuximab Study Drug Discontinuation Visit[28]**	**mFOLFOX6 and/or nivolumab Study Drug Discontinuation Visit[28]**	**Zolbetuximab 30-Day Safety Follow-up Visit[29]**	**mFOLFOX6 and/or nivolumab 30-Day Safety Follow-up Visit[30]**	**Zolbetuximab 90-Day Follow-up Visit[31]**	**mFOLFOX6 and/or nivolumab 90-Day Safety Follow-up Visit[32]**	**Post-treatment Follow-up[33]**	**Survival Follow-up (Cohort 4B only)[34]**
		**Cycles 1 to 4**<br><br>**zolbetuximab + mFOLFOX6 + nivolumab**			**Cycles ≥ 5**<br><br>**zolbetuximab + leucovorin or folinic acid + 5-FU + nivolumab**										
**Day**		**1**	**15**	**29**	**1**	**15**	**29**								
Vital Signs[12]	If Day 15 and Day 29 dosing is administered at local facility, standard of care can be applied; if there are no associated active AEs missed assessments at Study Treatment Discontinuation Visit and 30-day Safety Follow-up Visit acceptable; Vital Sign frequency during post observation period can be decreased.	X	X	X	X	X	X								
**Laboratory Assessments**															
Biochemistry[13]	Sample may be collected up to 4 days prior to treatment visit; collection of samples at local facility acceptable if results can be made available to investigative site.<br>Subjects in Cohort 4A prior to tolerability assessment, the visits must occur as scheduled.<br>Subjects in Cohort 4A after tolerability assessment and all subjects in Cohort 4B, collection of samples at local facilities is acceptable	X	X	X	X	X	X	X	X	X					
Hematology[13]		X	X	X	X	X	X	X	X	X					

Table continued on next page

Critical Assessment	Alternate Approach(es)†	Critical Timepoint: Treatment Period (1 cycle = 42 days): Cycles 1 to 4 zolbetuximab + mFOLFOX6 + nivolumab			Critical Timepoint: Treatment Period (1 cycle = 42 days): Cycles ≥ 5 zolbetuximab + leucovorin or folinic acid + 5-FU + nivolumab			Follow-up: Zolbetuximab Study Drug Discontinuation Visit[28]	Follow-up: mFOLFOX6 and/or nivolumab Study Drug Discontinuation Visit[28]	Follow-up: Zolbetuximab 30-Day Safety Follow-up Visit[29]	Follow-up: mFOLFOX6 and/or nivolumab 30-Day Safety Follow-up Visit[30]	Follow-up: Zolbetuximab 90-Day Follow-up Visit[31]	Follow-up: mFOLFOX6 and/or nivolumab 90-Day Safety Follow-up Visit[32]	Follow-up: Post-treatment Follow-up[33]	Follow-up: Survival Follow-up (Cohort 4B only)[34]
**Day**		**1**	**15**	**29**	**1**	**15**	**29**								
Urinalysis[14]	Sample may be collected up to 4 days prior to treatment visit; collection at local facility also allowed.	If clinically indicated						X	X	X					
DPD testing per local requirements	Sample testing can occur locally, if indicated, as part of screening.														
Cytokine/ Chemokine and/or Tryptase	Samples will only be collected on zolbetuximab treatment days at the study site.	If clinically indicated													
TSH and T4	If this testing is unable to be performed, this is acceptable.	X[36]			X[36]										
PT, PTT and INR[15]	None as protocol allows standard of care if clinically indicated. If the subject is not on a concomitant medication that affects these parameters, acceptable if these are not done	If clinically indicated													
Serum Pregnancy Test[16]	Collection at local facility also allowed.	If clinically indicated and/or per local requirements													

Table continued on next page

Critical Assessment	Alternate Approach(es)†	Critical Timepoint						Follow-up							
		Treatment Period (1 cycle = 42 days)						Zolbetuximab Study Drug Discontinuation Visit[28]	mFOLFOX6 and/or nivolumab Study Drug Discontinuation Visit[28]	Zolbetuximab 30-Day Safety Follow-up Visit[29]	mFOLFOX6 and/or nivolumab 30-Day Safety Follow-up Visit[30]	Zolbetuximab 90-Day Follow-up Visit[31]	mFOLFOX6 and/or nivolumab 90-Day Safety Follow-up Visit[32]	Post-treatment Follow-up[33]	Survival Follow-up (Cohort 4B only)[34]
		Cycles 1 to 4<br>zolbetuximab + mFOLFOX6 + nivolumab			Cycles ≥ 5<br>zolbetuximab + leucovorin or folinic acid + 5-FU + nivolumab										
**Day**		1	15	29	1	15	29								
Urine Pregnancy Test[17]	Collection at local facility also allowed if results can be made available to investigative site. Sample may be collected up to 4 days prior to treatment visit.	X	X	X	X	X	X	X	X	X					
Pharmacokinetics of Zolbetuximab	If subject is dosed or visits clinic, PK samples should be collected. Samples cannot be collected at local facility.<br>If central labs cannot receive sample shipments, samples can be stored at sites in - 70°C degree freezer until shipping is accepted again. .	See Table 10 for pharmacokinetic collection schedule								X		X			
Pharmacokinetics of nivolumab	If subject is dosed or visits clinic, PK samples should be collected. Samples cannot be collected at local facility.<br>If central labs cannot receive sample shipments, samples can be stored at sites in - 70°C degree freezer until shipping is accepted again.	See Table 10 for pharmacokinetic collection schedule									X		X		

Table continued on next page

**Critical Assessment**	**Alternate Approach(es)†**	**Critical Timepoint**						**Follow-up**							
		**Treatment Period (1 cycle = 42 days)**						**Zolbetuximab Study Drug Discontinuation Visit[28]**	**mFOLFOX6 and/or nivolumab Study Drug Discontinuation Visit[28]**	**Zolbetuximab 30-Day Safety Follow-up Visit[29]**	**mFOLFOX6 and/or nivolumab 30-Day Safety Follow-up Visit[30]**	**Zolbetuximab 90-Day Follow-up Visit[31]**	**mFOLFOX6 and/or nivolumab 90-Day Safety Follow-up Visit[32]**	**Post-treatment Follow-up[33]**	**Survival Follow-up (Cohort 4B only)[34]**
		**Cycles 1 to 4**<br>**zolbetuximab + mFOLFOX6 + nivolumab**			**Cycles ≥ 5**<br>**zolbetuximab + leucovorin or folinic acid + 5-FU + nivolumab**										
**Day**		**1**	**15**	**29**	**1**	**15**	**29**								
Anti-Drug Antibodies (immunogenicity)	If subject is dosed or visits investigative site, ADA samples should be collected. Samples cannot be collected at local facility. Sample collection prioritized if clinically indicated. If central lab cannot receive samples, samples can be stored at sites in -70°C freezer until shipping is accepted again.	See Table 10 for sample collection schedule								X		X			
Tumor Markers[18a]	Biomarker samples are collected on zolbetuximab treatment days and do not require a unique visit to the study site. If samples can be collected but central labs cannot receive samples, samples can be stored at sites until shipping is accepted again.	X	X	X	X			X							

Table continued on next page

Critical Assessment	Alternate Approach(es)†	Critical Timepoint						Follow-up							
		Treatment Period (1 cycle = 42 days)						Zolbetuximab Study Drug Discontinuation Visit[28]	mFOLFOX6 and/or nivolumab Study Drug Discontinuation Visit[28]	Zolbetuximab 30-Day Safety Follow-up Visit[29]	mFOLFOX6 and/or nivolumab 30-Day Safety Follow-up Visit[30]	Zolbetuximab 90-Day Follow-up Visit[31]	mFOLFOX6 and/or nivolumab 90-Day Safety Follow-up Visit[32]	Post-treatment Follow-up[33]	Survival Follow-up (Cohort 4B only)[34]
		Cycles 1 to 4<br><br>zolbetuximab + mFOLFOX6 + nivolumab			Cycles ≥ 5<br><br>zolbetuximab + leucovorin or folinic acid + 5-FU + nivolumab										
Day		1	15	29	1	15	29								
Exploratory Biomarkers (Serum)[18b]	Biomarker samples are collected on zolbetuximab treatment days and do not require a unique visit to the study site. If samples can be collected but central labs cannot receive samples, samples can be stored at sites until shipping is accepted again.	X	X	X				X							
Exploratory Biomarkers (Plasma)[18b]	Biomarker samples are collected on zolbetuximab treatment days and do not require a unique visit to the study site. If samples can be collected but central labs cannot receive samples, samples can be stored at sites until shipping is accepted again.	X	X	X				X							

Table continued on next page

Critical Assessment	Alternate Approach(es)†	Critical Timepoint						Follow-up							
		Treatment Period (1 cycle = 42 days)						Zolbetuximab Study Drug Discontinuation Visit[28]	mFOLFOX6 and/or nivolumab Study Drug Discontinuation Visit[28]	Zolbetuximab 30-Day Safety Follow-up Visit[29]	mFOLFOX6 and/or nivolumab 30-Day Safety Follow-up Visit[30]	Zolbetuximab 90-Day Follow-up Visit[31]	mFOLFOX6 and/or nivolumab 90-Day Safety Follow-up Visit[32]	Post-treatment Follow-up[33]	Survival Follow-up (Cohort 4B only)[34]
		Cycles 1 to 4<br>zolbetuximab + mFOLFOX6 + nivolumab			Cycles ≥ 5<br>zolbetuximab + leucovorin or folinic acid + 5-FU + nivolumab										
Day		1	15	29	1	15	29								
Immune Cell Subsets (Whole Blood) [18b]	Biomarker samples are collected on zolbetuximab treatment days and do not require a unique visit to the study site. If samples can be collected but central labs cannot receive samples, samples can be stored at sites until shipping is accepted again.	X	X	X				X							
Cryopreserved PBMC[18b]	Samples are collected on zolbetuximab treatment days and do not require a unique visit to the study site. If samples can be collected but central labs cannot receive samples, samples can be stored at sites until shipping is accepted again.	X	X	X				X							

Table continued on next page

**Critical Assessment**	**Alternate Approach(es)†**	**Critical Timepoint**						**Follow-up**							
		**Treatment Period (1 cycle = 42 days)**						**Zolbetuximab Study Drug Discontinuation Visit[28]**	**mFOLFOX6 and/or nivolumab Study Drug Discontinuation Visit[28]**	**Zolbetuximab 30-Day Safety Follow-up Visit[29]**	**mFOLFOX6 and/or nivolumab 30-Day Safety Follow-up Visit[30]**	**Zolbetuximab 90-Day Follow-up Visit[31]**	**mFOLFOX6 and/or nivolumab 90-Day Safety Follow-up Visit[32]**	**Post-treatment Follow-up[33]**	**Survival Follow-up (Cohort 4B only)[34]**
		**Cycles 1 to 4**<br>**zolbetuximab + mFOLFOX6 + nivolumab**			**Cycles ≥ 5**<br>**zolbetuximab + leucovorin or folinic acid + 5-FU + nivolumab**										
**Day**		**1**	**15**	**29**	**1**	**15**	**29**								
Genetic Immune Polymorphisms – (whole blood)[18c]	Biomarker samples are collected on zolbetuximab treatment days and do not require a unique visit to the study site. If samples can be collected but central labs cannot receive samples, samples can be stored at sites until shipping is accepted again.	X													
Whole Blood Sample for PGx (optional)[19]	Biomarker samples are collected on zolbetuximab treatment days and do not require a unique visit to the study site. If samples can be collected but central labs cannot receive samples, samples can be stored at sites until shipping is accepted again.	X													

Table continued on next page

**Critical Assessment**	**Alternate Approach(es)†**	**Critical Timepoint**						**Follow-up**							
		**Treatment Period (1 cycle = 42 days)**						**Zolbetuximab Study Drug Discontinuation Visit[28]**	**mFOLFOX6 and/or nivolumab Study Drug Discontinuation Visit[28]**	**Zolbetuximab 30-Day Safety Follow-up Visit[29]**	**mFOLFOX6 and/or nivolumab 30-Day Safety Follow-up Visit[30]**	**Zolbetuximab 90-Day Follow-up Visit[31]**	**mFOLFOX6 and/or nivolumab 90-Day Safety Follow-up Visit[32]**	**Post-treatment Follow-up[33]**	**Survival Follow-up (Cohort 4B only)[34]**
		**Cycles 1 to 4**<br><br>**zolbetuximab + mFOLFOX6 + nivolumab**			**Cycles ≥ 5**<br><br>**zolbetuximab + leucovorin or folinic acid + 5-FU + nivolumab**										
**Day**		**1**	**15**	**29**	**1**	**15**	**29**								
**Cardiac Safety**															
12-lead ECG[20]	ECGs allowed up to 4 days prior to treatment visits after C1D1 and can be done locally but must be available for review prior to treatment; if dosing visits on Day 15 or Day 29 are conducted at local facility, standard of care ECG monitoring would be acceptable; Study Treatment Discontinuation Visit and 30-Day Safety Follow Up Visit required only if clinically indicated	X	X	X											
**Radiology**															
Image Assessment[21]	At select visits, efficacy assessment using radiological examinations are required. Imaging assessment will be done locally but must be available for submission to central imaging vendor.	Every 8 (± 1) weeks from C1D1 for the first 56 weeks and then every 12 (± 2) weeks thereafter.													
Table continued on next page															

Critical Assessment	Alternate Approach(es)†	Critical Timepoint						Follow-up							
		Treatment Period (1 cycle = 42 days)						Zolbetuximab Study Drug Discontinuation Visit[28]	mFOLFOX6 and/or nivolumab Study Drug Discontinuation Visit[28]	Zolbetuximab 30-Day Safety Follow-up Visit[29]	mFOLFOX6 and/or nivolumab 30-Day Safety Follow-up Visit[30]	Zolbetuximab 90-Day Follow-up Visit[31]	mFOLFOX6 and/or nivolumab 90-Day Safety Follow-up Visit[32]	Post-treatment Follow-up[33]	Survival Follow-up (Cohort 4B only)[34]
		Cycles 1 to 4<br>zolbetuximab + mFOLFOX6 + nivolumab			Cycles ≥ 5<br>zolbetuximab + leucovorin or folinic acid + 5-FU + nivolumab										
**Day**		1	15	29	1	15	29								
**HRQoL**															
HRQoL[22]	Not required at Study Treatment Discontinuation visit; may be assessed and captured via phone contact; may be captured a day or 2 ahead of visit	X	X	X	X	X	X	X		X		X			
**Tissue Samples**															
On-Treatment Biopsy[24] (Required for Cohort 4B, optional for Cohort 4A)	Biomarker samples are collected on zolbetuximab treatment days and do not require a unique visit to the study site. If samples can be collected but central labs cannot receive samples, samples can be stored at sites until shipping is accepted again.	X													
Post-progression Tumor Sample (optional)[25]	Biomarker samples are collected on zolbetuximab treatment days and do not require a unique visit to the study site. If samples can be collected but central labs cannot receive samples, samples can be stored at sites until shipping is accepted again.							X							

Table continued on next page

Critical Assessment	Alternate Approach(es)†	Critical Timepoint						Follow-up							
		Treatment Period (1 cycle = 42 days)						Zolbetuximab Study Drug Discontinuation Visit[28]	mFOLFOX6 and/or nivolumab Study Drug Discontinuation Visit[28]	Zolbetuximab 30-Day Safety Follow-up Visit[29]	mFOLFOX6 and/or nivolumab 30-Day Safety Follow-up Visit[30]	Zolbetuximab 90-Day Follow-up Visit[31]	mFOLFOX6 and/or nivolumab 90-Day Safety Follow-up Visit[32]	Post-treatment Follow-up[33]	Survival Follow-up (Cohort 4B only)[34]
		Cycles 1 to 4<br>zolbetuximab + mFOLFOX6 + nivolumab			Cycles ≥ 5<br>zolbetuximab + leucovorin or folinic acid + 5-FU + nivolumab										
**Day**		1	15	29	1	15	29								
**Safety Assessment**															
Concomitant Medication[26]	Remote/Virtual Telemedicine Visits allowed for non-dosing visits. Please refer to protocol schedule of assessments.	X	X	X	X	X	X	X	X	X	X	X	X		
AEs/SAEs[27]	Remote/Virtual Telemedicine Visits allowed for non-dosing visits. Please refer to protocol schedule of assessments.	X	X	X	X	X	X	X	X	X	X	X	X		
Survival Follow-up[33]	Remote/Virtual Telemedicine Visits allowed for non-dosing visits. Please refer to protocol schedule of assessments.														X
Subject Contact[35]	Long Term Survival and Safety follow-up visits can be conducted via phone.									X		X		X	X

5-FU: fluorouracil; ADA: anti-drug antibody; AE: adverse event; BSA: body surface area; C: cycle; CLDN: claudin; CT: computerized tomography; D: day; eCRF: electronic case report form; ECG: electrocardiogram; ECOG: Eastern Cooperative Oncology Group; FFPE: formalin-fixed paraffin-embedded; GP: Global Pain; HER2: human epidermal growth factor receptor 2; HRQoL: health-related quality of life; HRU: Health Resource Utilization; ICF: informed consent form; INR: international normalized ratio; IRR: infusion related reaction; IV: intravenous; MRI: magnetic resonance imaging; ORR: objective response rate; PBMC: peripheral blood mononuclear cells; PET: positron emission tomography; PFS: progression-free survival; PGx: pharmacogenomics; PT: prothrombin time; PTT: partial thromboplastin time; SAE: serious adverse event; T4: thyroxine; TSH: thyroid-stimulating hormone.

Footnotes continued on next page

Laboratory tests will be performed predose according to the Schedule of Assessments and sent to a central laboratory for analysis. In case of multiple laboratory data within this period, the most recent data should be used.

† Alternative schedule of assessments only applies to subjects in Cohort 4A after tolerability assessment and all subjects in Cohort 4B.

1. *This footnote has been deleted.*
2. *This footnote has been deleted.*
3. *This footnote has been deleted.*
4. *This footnote has been deleted.*
5. All laboratory results should be available and reviewed by the investigator before starting any study treatment (zolbetuximab, mFOLFOX6, nivolumab).
6. Prophylactic antiemetics and, if needed, other premedications should be given according to institutional standards and the respective product package insert(s). Antiemetic premedication should be given at least 30 minutes prior to zolbetuximab administration. If one study drug is discontinued, antiemetic premedication should be given at least 30 minutes prior to each dose of zolbetuximab, mFOLFOX6 and nivolumab. It is recommended that the prophylactic antiemetic regimen include the following agents: NK-1 receptor blockers and 5-HT3 receptor blockers.
7. Zolbetuximab will be administered as a minimum 2-hour IV infusion. Zolbetuximab IV infusion may be interrupted or slowed down to manage toxicity.
8. Post-Infusion Observation Period: Following the subject's first dose of zolbetuximab, the subject must remain at the site facility for 2 hours post infusion. If AEs are observed during this time, infusion time should be extended and subjects should continue to be observed for 2 hours post infusion.
9. Nivolumab will be administered every 2 weeks at a dose of 240 mg. Zolbetuximab will be administered first, followed by nivolumab and then mFOLFOX6.
10. mFOLFOX6 will be administered every 2 weeks. mFOLFOX6 will be administered after zolbetuximab and nivolumab. Beginning at cycle 5, subjects may continue on 5-FU and leucovorin or folinic acid along with zolbetuximab and nivolumab for the remainder of the study per investigator's discretion.
11. Physical examination and other evaluations include height (at screening only), weight and ECOG performance status. The physical exam only needs to be repeated on C1D1 if clinically significant changes from screening (in the opinion of the investigator) are observed. Targeted (symptom-driven) physical exams should be conducted every 2 weeks on zolbetuximab visit days.
12. Vital signs (pulse, blood pressure, temperature) should be taken at the following time points:
    a. Predose at every visit.
    b. C1D1: Every 30 (± 10) minutes during zolbetuximab infusion.
    c. Subsequent zolbetuximab infusions: every 60 (± 10) minutes during zolbetuximab infusions if the subject did not develop any ≥ grade 2 AEs during the C1D1 zolbetuximab infusion or the Post-Infusion Observation Period. If the subject has an > grade 2 AE, then vital signs are to be taken be every 30 minutes, subsequently. If the next cycle is ≤ grade 1, then vital signs can be assessed every 60 minutes.
    d. Every 60 (±10) minutes post zolbetuximab infusion during the Post-Infusion Observation Period.
    e. Unscheduled if clinically indicated

Footnotes continued on next page

13. See [Section 5.4.3] for list of laboratory assessments. Laboratory tests must be sent to the central laboratory for analysis unless otherwise approved by the sponsor. For screening/eligibility laboratory assessments, see footnote 2.
    - Laboratory test results (central or local) will be reviewed by the investigator prior to any study treatment. Clinical significance of out-of-range laboratory findings is to be determined and documented by the investigator/subinvestigator who is a qualified physician.
    - Local laboratory results may be used for treatment decisions; however, central laboratory samples must also be drawn per protocol and sent to the central laboratory unless otherwise approved by the sponsor.
    - Central and local labs may be collected up to 48 hours prior to study treatment.
    - Holidays and weekends should be taken into account when scheduling these blood draws.
    - Additional assessments may be done centrally or locally to monitor AEs or as clinically indicated.
14. Urinalysis: Urinalysis should be performed at screening and is to be repeated if clinically indicated after starting study treatment. Urinalysis should also be performed for the zolbetuximab, mFOLFOX6 and nivolumab study drug discontinuation visits and at the zolbetuximab 30-day follow-up visit. Urinalysis tests will be sent to a central laboratory for analysis.
15. Ongoing evaluation should be continued for subjects who are receiving therapeutic anticoagulation according to local standard of care.
16. Serum Pregnancy Test: Will be collected for female subjects of childbearing potential only. Serum pregnancy tests are to be completed at screening. Subjects with elevated serum βHCG during screening and a demonstrated non-pregnant status through additional testing are eligible. Central laboratory must be used to confirm eligibility.
17. Urine Pregnancy Test: For female subjects of childbearing potential only. Urine pregnancy tests are to be completed at every visit prior to zolbetuximab administration. Urine pregnancy can be confirmed at a local laboratory.
18. Biomarker (serum, plasma, whole blood and cryopreserved PBMCs) samples should be taken within 48 hours prior to dosing:
    a. Samples will be collected predose at C1D1, C1D15, C1D29, C2D1, C2D29, C3D15, C4D1, C4D29 and then predose on D1 of every additional cycle through zolbetuximab study drug discontinuation.
    b. Samples will be collected predose at C1D1, C1D15, C1D29, C2D1, C2D29, C3D15, C4D1, C4D29 and zolbetuximab study discontinuation.
    c. Samples will be collected predose on C1D1.
19. For subjects who signed a separate ICF, an optional whole blood sample for PGx for exploratory biomarker analysis should be collected within 48 hours prior to dosing.

Footnotes continued on next page

20. A single ECG will be performed at the following time points:
    a. Screening
    b. Up to 48 hours prior to every oxaliplatin infusion (before any antiemetic treatment)†
    c. Up to 6 hours following completion of every oxaliplatin infusion†
    d. Zolbetuximab study drug discontinuation
    e. Zolbetuximab 30-day follow-up visit
    f. If clinically indicated

    †Local read only – do not transmit to central ECG laboratory.
21. Imaging assessments to be collected at screening and every 8 (± 1) weeks counting from C1D1 for the first 56 weeks, and then every 12 (± 2) weeks thereafter. Imaging assessments completed prior to consent, but within 28 days of first dose of study drug, may be used for screening. Imaging will include CT scans with contrast of the thorax, abdomen, and pelvis (if CT scan is medically not feasibly with contrast, MRI may be used for imaging). Bone scans (or focal X-ray) or brain imaging should be performed if metastatic disease is suspected. Disease must be evident by radiology; measurable lesions only. Same mode of imaging should be utilized throughout the study unless medical necessity requires change. CT scan performed with PET scan can be used if it is of quality, which allows accurate tumor measurement. Progression and tumor responses will be evaluated by investigator per RECIST 1.1. All imaging will be sent to a central independent radiographic review within 7 days.
22. HRQoL questionnaires and HRU are to be administered on zolbetuximab visit days before any drug treatment (or up to 48 hours prior to treatment) or other scheduled assessments are conducted and before the disease status is discussed with the subject. HRQoL will be measured by QLQ-C30, OG-25, GP and the EQ-5D questionnaires. The HRU questionnaire will not be administered at the screening visit. Questionnaires should only be administered on days when the subject receives zolbetuximab treatment. HRQoL and HRU questionnaires are not required at mFOLFOX6 and/or nivolumab treatment discontinuation visit, 30-day safety follow-up visit or 90-day safety follow-up visit. A combined visit can be completed if zolbetuximab is discontinued on the same day. Questionnaire completion will not be required if the subject is illiterate or the questionnaire is not available in the local language.
23. *This footnote has been deleted.*
24. Cohort 4B Only: Subjects are required to provide an on-treatment tumor specimen collected ± 15 days of the C2D1 visit. A minimum of 1 FFPE tumor tissue block (preferred) OR a minimum of 15 FFPE unstained sections are required. This is optional for Cohort 4A. If ≥ 15 slides cannot be provided, the sponsor should be contacted for further guidance.
25. For subjects who signed a separate ICF, an optional post-progression tumor sample for exploratory biomarker analysis should be collected following confirmation of radiographic disease progression and prior to commencement of subsequent anti-cancer therapy.
26. Concomitant medications will be collected from the time of main study main informed consent through 90 days following the last dose of study drug.
27. AEs and SAEs (regardless of causality) will be collected from the time of main informed consent through 90 days following the last dose of study drug.
28. Study Drug Discontinuation: Visit will occur within 7 days after the last dose or decision by the investigator to discontinue subject from treatment.
29. Zolbetuximab 30-day Safety Follow-up Visit should occur 30 days (± 7) after last dose of zolbetuximab.

Footnotes continued on next page

30. mFOLFOX6/nivolumab 30-day Safety Follow-up Visit should occur 30 days (± 7) after last dose of mFOLFOX6 (all components) and/or nivolumab.
    - For subjects that have discontinued mFOLFOX6 and nivolumab on the same day, the safety follow-up visit must be conducted in-person.
    - For subjects that have discontinued on mFOLFOX6 and nivolumab on different days, the safety follow-up visit for nivolumab must be conducted in-person, while the safety follow-up visit for mFOLFOX6 can be conducted via phone call.
31. Zolbetuximab 90-day Follow-up Visit should occur 90 days (+ 7) after last dose of zolbetuximab.
32. mFOLFOX6/nivolumab 90-day Safety Follow-up Visit should occur 90 days (+ 7) after the last dose of mFOLFOX6 (all components) and/or nivolumab.
    - For subjects that have discontinued mFOLFOX6 and nivolumab on the same day, the safety follow-up visit must be conducted in-person.
    - For subjects that have discontinued on mFOLFOX6 and nivolumab on different days, the safety follow-up visit for nivolumab must be conducted in-person, while the safety follow-up visit for mFOLFOX6 can be conducted via phone call.
33. Post-treatment Follow-up: if a subject discontinues study drug prior to radiographic disease progression as confirmed by the independent central reader, the subject should enter the post-treatment follow-up period and continue to undergo imaging assessments until radiographic progression is documented per the investigator, or the subject starts another cancer treatment, whichever occurs earlier.
34. Cohort 4B only: Survival follow-up by telephone contact is to be performed every 12 weeks ± 2 weeks for all subjects with proven radiographic disease progression or who left the study due to any other reason than death or withdrawal of consent. Subjects will be followed to collect survival status until subject death, withdrawal of consent, or study closure. Additional follow-up contacts may be required per sponsor request for analysis purposes.
35. Long Term and Survival Follow-up Period: Following radiographic disease progression on 1st line treatment or start of subsequent anti-cancer treatment, subjects will be followed in the Long-Term and Survival Follow-up Period per institutional guidelines, but not less frequently than every 12 weeks. Radiologic imaging will be done per standard of care and read locally. Survival Follow-up Period will continue until death (from any cause). All post-progression details including subsequent anticancer treatment and date and site of radiographic progression will be recorded on the eCRF. Subject contact by phone or other remote methods is sufficient during Long Team and Survival Follow-up.
36. Thyroid function to be assessed at day 1 of cycles 3, 5 and all odd cycles >5.

Table 38 Alternative Schedule of Assessments in Response to a Crisis, Cohort 5

**Critical Assessment**	**Alternate Approach(es)**	**Preoperative cycles 1 to 4 zolbetuximab + FLOT**		**Preoperative Follow-up**		**Surgery[1]**	**Postoperative cycles 5 to 8 zolbetuximab + FLOT[19]**		**Postoperative Follow-up or Early Discontinuation**			**Post-Treatment Follow-up[21]**	**Survival Follow-up[22]**
**Day**		**1**	**2[2]**	**7-14 days after last preop dose**	**30 days after last preop dose**	**0**	**1**	**2[2]**	**EOT[20]**	**EOT +30**	**EOT +90**		
Visit Window (business days)		C1: 0<br>C2-4: +2	+ 2		+ 7		+ 2	+ 2	+ 7	± 7	± 7		
**Study Drug Administration**													
Antiemetic Pretreatment[3]	Can be administered at home per standard of care	X	X[2]				X	X[2]					
Zolbetuximab[4]		X					X						
FLOT		X[2]	X[2]				X[2]	X[2]					
**Physical Examination/Assessments**													
Physical Examination[5]		X		X	X		X		X	X			
Weight[5]		X		X	X		X		X	X			
ECOG Performance Status[5]		X		X	X		X		X	X			
Vital Signs[6]		X					X		X	X			
HRQoL[7]		X		X	X		X		X	X	X	X	
**Laboratory Assessments**													
Biochemistry[8]		X					X		X	X			
Hematology[8]		X					X		X	X			

Table continued on next page

**Critical Assessment**	**Alternate Approach(es)**	**Preoperative cycles 1 to 4 zolbetuximab + FLOT**		**Preoperative Follow-up**		**Surgery[1]**	**Postoperative cycles 5 to 8 zolbetuximab + FLOT[19]**		**Postoperative Follow-up or Early Discontinuation**			**Post-Treatment Follow-up[21]**	**Survival Follow-up[22]**
**Day**		**1**	**2[2]**	**7-14 days after last preop dose**	**30 days after last preop dose**	**0**	**1**	**2[2]**	**EOT[20]**	**EOT +30**	**EOT +90**		
Visit Window (business days)		C1: 0<br>C2-4: +2	+ 2		+ 7		+ 2	+ 2	+ 7	± 7	± 7		
Urinalysis[9]		If Clinically Indicated							X	X			
Cytokine/Chemokine and/or Tryptase	If Clinically Indicated												
TSH and T4		X	If Clinically Indicated										
PT, PTT and INR[10]		If Clinically Indicated											
Urine Pregnancy Test[11]		X					X		X	X			
Pharmacokinetics of Zolbetuximab	See Table 12												
Anti-Drug Antibodies (immunogenicity)	See Table 12												
Exploratory Biomarkers (blood/plasma)[12]	See Table 13												
Whole Blood Sample for PGx (optional)[13]		X											
**Tissue Samples**													
Postoperative (Surgical) Sample[14]						X							

Table continued on next page

**Critical Assessment**	**Alternate Approach(es)**	**Preoperative cycles 1 to 4 zolbetuximab + FLOT**		**Preoperative Follow-up**		**Surgery[1]**	**Postoperative cycles 5 to 8 zolbetuximab + FLOT[19]**		**Postoperative Follow-up or Early Discontinuation**			**Post-Treatment Follow-up[21]**	**Survival Follow-up[22]**
**Day**		**1**	**2[2]**	**7-14 days after last preop dose**	**30 days after last preop dose**	**0**	**1**	**2[2]**	**EOT[20]**	**EOT +30**	**EOT +90**		
Visit Window (business days)		C1: 0<br>C2-4: +2	+ 2		+ 7		+ 2	+ 2	+ 7	± 7	± 7		
Post-progression Tumor Sample (optional)[15]	Biomarker samples are collected on zolbetuximab treatment days and do not require a unique visit to the study site. If samples can be collected but central labs cannot receive samples, samples can be stored at sites until shipping is accepted again.									X			
Diagnostic Laparoscopy (optional depending on institutional guidelines/standard-of-care)					X								
Table continued on next page													

**Critical Assessment**	**Alternate Approach(es)**	**Preoperative cycles 1 to 4 zolbetuximab + FLOT**		**Preoperative Follow-up**		**Surgery[1]**	**Postoperative cycles 5 to 8 zolbetuximab + FLOT[19]**		**Postoperative Follow-up or Early Discontinuation**			**Post-Treatment Follow-up[21]**	**Survival Follow-up[22]**
**Day**		**1**	**2[2]**	**7-14 days after last preop dose**	**30 days after last preop dose**	**0**	**1**	**2[2]**	**EOT[20]**	**EOT +30**	**EOT +90**		
Visit Window (business days)		C1: 0<br>C2-4: +2	+ 2		+ 7		+ 2	+ 2	+ 7	± 7	± 7		
**Radiology**													
Image Assessment	At select visits, efficacy assessment using radiological examinations are required. Imaging assessment can be done locally and if available, should be submitted to central imaging vendor. Independent central reading of locally obtained scans can be facilitated by sharing of	Imaging at restaging (after preoperative chemotherapy prior to surgery) and after a subject discontinues or completes all study treatment (every 3 [± 1] months during the first year, then every 4 [± 2] months during the second year in post-treatment follow-up period.											
Table continued on next page													

**Critical Assessment**	**Alternate Approach(es)**	**Preoperative cycles 1 to 4 zolbetuximab + FLOT**		**Preoperative Follow-up**		**Surgery[1]**	**Postoperative cycles 5 to 8 zolbetuximab + FLOT[19]**		**Postoperative Follow-up or Early Discontinuation**			**Post-Treatment Follow-up[21]**	**Survival Follow-up[22]**
**Day**		**1**	**2[2]**	**7-14 days after last preop dose**	**30 days after last preop dose**	**0**	**1**	**2[2]**	**EOT[20]**	**EOT +30**	**EOT +90**		
Visit Window (business days)		C1: 0<br>C2-4: +2	+ 2		+ 7		+ 2	+ 2	+ 7	± 7	± 7		
	Image Acquisition Guidelines from study site to local site if applicable. Investigational site will be requested to re-read the scan performed at local site. If investigational site read is not an option, the investigator should discuss the case with the local institution radiologist.												

Table continued on next page

**Critical Assessment**	**Alternate Approach(es)**	**Preoperative cycles 1 to 4 zolbetuximab + FLOT**		**Preoperative Follow-up**		**Surgery[1]**	**Postoperative cycles 5 to 8 zolbetuximab + FLOT[19]**		**Postoperative Follow-up or Early Discontinuation**			**Post-Treatment Follow-up[21]**	**Survival Follow-up[22]**
**Day**		**1**	**2[2]**	**7-14 days after last preop dose**	**30 days after last preop dose**	**0**	**1**	**2[2]**	**EOT[20]**	**EOT +30**	**EOT +90**		
Visit Window (business days)		C1: 0<br>C2-4: +2	+ 2		+ 7		+ 2	+ 2	+ 7	± 7	± 7		
**Safety Assessments**													
12-lead ECG[16]		X		X	X		X		X	X			
Concomitant Medication[17]	Remote/Virtual/ Telemedicine Visits allowed for non-dosing visits. Please refer to protocol schedule of assessments.	X	X	X	X	X	X	X	X	X	X		
AEs/SAEs[18]	Remote/Virtual/ Telemedicine Visits allowed for non-dosing visits. Please refer to protocol schedule of assessments.	X	X	X	X	X	X	X	X	X	X		
Survival Assessment													X

Footnotes appear on next page

AE: adverse event; βHCG: beta human chorionic gonadotropin; C: cycle; D: day; DPD: dihydropyrimidine dehydrogenase; ECG: electrocardiogram; ECOG: Eastern Cooperative Oncology Group; EOT: end of treatment; EQ-5D: EuroQOL five dimensions questionnaire; FFPE: formalin-fixed paraffin-embedded; FLOT: fluorouracil, leucovorin or folinic acid, oxaliplatin and docetaxel; GP: Global Pain; HRQoL: health-related quality of life; HRU: Health Resource Utilization; ICF: informed consent form; INR: international normalized ratio; IV: intravenous; OG-25: Oesophago-Gastric Module (EORTC QLQ-OG-25); PGx: pharmacogenomics; preop: preoperative; PT: prothrombin time; PTT: partial thromboplastin time; QLQ-C30: Quality of Life Questionnaire - Core Questionnaire; SAE: serious adverse event; TSH: thyroid-stimulating hormone

Laboratory tests will be performed predose according to the Schedule of Assessments and sent to a central laboratory for analysis. In case of multiple laboratory data within this period, the most recent data should be used.

1. Surgery to be performed 4-8 weeks after completion of preoperative chemotherapy.
2. For cycles 1 and 5, zolbetuximab will be administered on day 1 and FLOT will be administered on day 2. For cycles 2-4 and 6-8, FLOT may be administered following zolbetuximab on day 1 or can be administered on day 2 as per investigator's clinical judgment.
3. Prophylactic antiemetics and, if needed, other premedications should be given according to institutional standards and the respective product package insert(s) (FLOT). Antiemetic premedication should be given at least 30 minutes prior to zolbetuximab administration. If one study drug is discontinued or administered on different days, antiemetic premedication should be given at least 30 minutes prior to each dose of zolbetuximab and FLOT. It is recommended that the prophylactic antiemetic regimen include the following agents: NK-1 receptor blockers and 5-HT3 receptor blockers.
4. Zolbetuximab will be administered with a preoperative loading dose at cycle 1 and a postoperative loading dose at cycle 5. Zolbetuximab IV infusion may be interrupted or slowed down to manage toxicity. Refer to [Section 5.1.1.1] for zolbetuximab dose regimen and infusion rate details.
5. Physical examination and other evaluations include height (at screening only), weight and ECOG performance status. The physical exam only needs to be repeated on C1D1 if clinically significant changes from screening (in the opinion of the investigator) are observed. Targeted (symptom-driven) physical exams should be conducted every 2 week visit.
6. Vital signs (pulse, blood pressure, temperature) should be taken at the following time points:
    - Predose at every visit.
    - C1D1: Every 30 (± 10) minutes during zolbetuximab infusion.
    - Subsequent zolbetuximab infusions: every 60 (± 10) minutes during zolbetuximab infusions if the subject did not develop any ≥ grade 2 AEs during the C1D1 zolbetuximab infusion or the Post-Infusion Observation Period. If the subject has an ≥ grade 2 AE, then vital signs are to be taken be every 30 minutes, subsequently. If the next cycle is ≤ grade 1, then vital signs can be assessed every 60 minutes.
    - Every 60 (±10) minutes post zolbetuximab infusion during the Post-Infusion Observation Period.
    - Unscheduled if clinically indicated
7. HRQoL questionnaires and HRU are to be administered on zolbetuximab visit days before any drug treatment (or up to 48 hours prior to treatment) or other scheduled assessments are conducted and before the disease status is discussed with the subject. HRQoL will be measured by QLQ-C30, OG-25, GP and the EQ-5D questionnaires. The HRU questionnaire will not be administered at the screening visit. Questionnaires should only be administered on days when the subject receives zolbetuximab treatment. A combined visit can be completed if zolbetuximab is discontinued on the same day. Questionnaire completion will not be required if the subject is illiterate or the questionnaire is not available in the local language.

Footnotes continued on next page

8. See [Section 5.4.3] for list of laboratory assessments. Laboratory tests must be sent to the central laboratory for analysis.
    - Laboratory test results (central or local) will be reviewed by the investigator prior to any study treatment. Clinical significance of out-of-range laboratory findings is to be determined and documented by the investigator/subinvestigator who is a qualified physician.
    - Local laboratory results may be used for treatment decisions; however, central laboratory samples must also be drawn per protocol and sent to the central laboratory.
    - Central and local labs may be collected up to 48 hours prior to study treatment.
    - Holidays and weekends should be taken into account when scheduling these blood draws.
    - Additional assessments may be done centrally or locally to monitor AEs or as clinically indicated.
9. Urinalysis should be performed at screening and is to be repeated if clinically indicated after starting study treatment. Urinalysis should also be performed at the postoperative follow-up or early discontinuation visits (EOT and EOT +30). Urinalysis tests will be sent to a central laboratory for analysis.
10. Ongoing evaluation should be continued for subjects who are receiving therapeutic anticoagulation according to local standard of care.
11. Urine Pregnancy Test: For female subjects of childbearing potential only. Urine pregnancy tests are to be completed at every visit prior to zolbetuximab administration. Urine pregnancy can be confirmed at a local laboratory.
12. Biomarkers will be collected through the post-treatment follow-up period as indicated in [Table 13].
13. For subjects who signed a separate ICF, an optional whole blood sample for PGx for exploratory biomarker analysis should be collected within 48 hours prior to dosing.
14. Subjects are required to provide the postoperative (surgical) sample. A minimum of 1 FFPE tumor tissue block (preferred) OR a minimum of 15 FFPE unstained sections are required.
15. For subjects who signed a separate ICF, an optional post-progression tumor sample for exploratory biomarker analysis should be collected following confirmation of radiographic disease progression and prior to commencement of subsequent anti-cancer therapy. A minimum 1 FFPE tumor tissue block (preferred) OR a minimum of 15 FFPE unstained sections are required.
16. A single ECG will be performed at the time points shown.
17. Concomitant medications will be collected from the time of main study informed consent through 90 days following the last dose of study drug.
18. AEs and SAEs (regardless of causality) will be collected from the time of main informed consent through 90 days following the last dose of study drug, except for inpatient hospitalization for planned procedures as allowed per study (e.g., surgery for subjects in Cohort 5).
19. Postoperative treatments will begin 6-12 weeks after completion of surgery.
20. End of treatment visit will occur within 7 days after the last dose or decision by the investigator to discontinue subject from treatment. If zolbetuximab is discontinued prior to FLOT, an EOT visit should be conducted within 7 days after zolbetuximab discontinuation and within 7 days after FLOT discontinuation.
21. Subjects who discontinue or complete all study treatment will enter the post-treatment follow-up period for up to 2 years.
22. Following completion of the post-treatment follow-up period, disease recurrence (or radiographic disease progression) or the start of another anticancer therapy (whichever occurs first), subjects in Cohort 5 will enter the survival follow-up period for the remainder of the 3-year period following last dose of study treatment.

IMP SUPPLY

If any of the conditions outlined above in the Participants Procedures Assessment are met, one or all of the following mitigating strategies will be employed, as needed, to ensure continuity of IMP supply to the participants:

- Increase stock of IMP on site to reduce number of shipments required, if site space will allow.

DATA COLLECTION REQUIREMENTS

Additional data may be collected in order to indicate how participation in the study may have been affected by a crisis and to accommodate data collection resulting from alternate measures implemented to manage the conduct of the study and participant safety.

- Critical assessments for safety and efficacy based on study endpoints to be identified as missing or altered (performed virtually, at alternative locations, out of window, or other modifications) due to the crisis.

13 COORDINATING INVESTIGATOR'S SIGNATURE

A Phase 2 Study of Zolbetuximab (IMAB362) as Monotherapy and in Combination with Chemotherapy and/or Immunotherapy in Subjects with Metastatic or Locally Advanced Unresectable Gastric or Gastroesophageal Junction (GEJ) Adenocarcinoma and Locoregional Gastric or GEJ Adenocarcinoma Whose Tumors are Claudin (CLDN) 18.2-Positive

ISN/Protocol 8951-CL-0103

Version 8.0 Incorporating Substantial Amendment 7

23 May 2024

I have read all pages of this clinical study protocol for which Astellas is the sponsor. I agree that it contains all the information required to conduct this study.

Coordinating Investigator:

Signature: ____________________ ____________
<Insert name, department/affiliation, name of institution> Date (DD Mmm YYYY)

Printed Name: ____________________

Address: ____________________

____

STATISTICAL ANALYSIS PLAN

Version 3.0, dated August 22, 2024

A Phase 2 Study of Zolbetuximab (IMAB362) as Monotherapy and in Combination with Chemotherapy and/or Immunotherapy in Subjects with Metastatic or Locally Advanced Unresectable Gastric or Gastroesophageal Junction (GEJ) Adenocarcinoma and Locoregional Gastric or GEJ Adenocarcinoma Whose Tumors are Claudin (CLDN) 18.2-Positive

ISN/Protocol 8951-CL-0103
IND number: 129598

Sponsor:
Astellas Pharma Global Development, Inc. (APGD)
Northbrook, IL 60062

This confidential document is the property of the sponsor. No unpublished information contained in this document may be disclosed without prior written approval of the sponsor.

Table of Contents

I. LIST OF ABBREVIATIONS AND KEY TERMS 5

1 INTRODUCTION 8

2 STUDY OBJECTIVE(S) AND DESIGN 8

2.1 Study Objective(s) 8

2.1.1 Primary Objective 8

2.1.2 Secondary Objectives 8

2.1.3 Exploratory Objective 9

2.2 Study Design 9

2.3 Randomization 9

3 SAMPLE SIZE 9

4 ANALYSIS SETS 10

4.1 Full Analysis Set (FAS) 10

4.2 Safety Analysis Set (SAF) 10

4.3 Pharmacokinetics Analysis Set (PKAS) 10

4.4 Biomarker Analysis Set (BMAS) (Cohort 4B and 5 only) 11

4.5 DLT Evaluation Analysis Set (DEAS) 11

5 ANALYSIS VARIABLES 11

5.1 Efficacy Endpoints 11

5.1.1 Primary Efficacy Endpoint(s) 11

5.1.2 Secondary Efficacy Endpoint 11

5.2 Safety Variables 14

5.2.1 Definition of DLT 14

5.2.2 AE 14

5.2.3 Clinical Laboratory Variables 15

5.2.4 Vital Signs 16

5.2.5 12-lead electrocardiogram (ECG) 16

5.2.6 ECOG performance scores 16

5.2.7 Physical Examination 16

5.2.8 Other Safety Aspects 17

5.3 Pharmacokinetics Variables 17

5.4 Pharmacodynamics Variables 18

5.5 Other Variables 18

6 STATISTICAL METHODOLOGY 19
6.1 General Considerations 19
6.2 Study Population 20
6.2.1 Disposition of Subjects 20
6.2.2 Protocol Deviations 22
6.2.3 Demographic and Other Baseline Characteristics 22
6.2.4 Previous and Concomitant Medications 22
6.3 Study Drugs 23
6.3.1 Exposure 23
6.3.2 Relative Dose Intensity 24
6.4 Analysis of Efficacy 24
6.4.1 Analysis of Primary Endpoint(s) 24
6.4.2 Analysis of Secondary Endpoints 24
6.4.3 Analysis of Exploratory Endpoints 26
6.4.4 Imaging 26
6.5 Analysis of Safety 26
6.5.1 Dose Limiting Toxcicities 26
6.5.2 Adverse Events 26
6.5.3 AE of Special interest 28
6.5.4 Laboratory Assessments 29
6.5.5 Vital Signs 30
6.5.6 Electrocardiogram 31
6.5.7 Eastern Cooperative Oncology Group Performance Status 32
6.5.8 Pregnancies 32
6.6 Analysis of PK 32
6.6.1 Concentrations 32
6.6.2 Estimation of Pharmacokinetics Parameters 33
6.6.3 Statistical Analysis of Pharmacokinetics Parameters 33
6.6.4 Immunogenicity 33
6.7 Subgroups of Interest 33
6.8 Interim Analysis (and Early Discontinuation of the Clinical Study) 33
6.9 Additional Conventions 34
6.9.1 Missing Data 34
6.9.2 Outliers 34

6.9.3 Visit Windows 34
7 DOCUMENT REVISION HISTORY 34
8 REFERENCES 34
9 APPENDICES 35
9.1 EORTC QLQ-C30 questionnaire (version 3) 35
9.2 EORTC QLQ-30 Scoring 37
9.3 EORTC QLQ-OG25 questionnaire 38
9.4 EQ-5D-5L Scoring 40
9.5 Global Pain 42
9.6 Health Resource Utilization 43
9.7 Author and Approver Signatories 45

I. LIST OF ABBREVIATIONS AND KEY TERMS

List of Abbreviations

**Abbreviations**	**Description of abbreviations**
ADA	anti-drug antibody
ADCC	antibody-dependent cellular cytotoxicity
AE	Adverse Event
ALP	Alkaline Phosphatase
ALT	Alanine Transaminase
ASCM	Analysis Set Classification Meeting
AST	Aspartate Transaminase
AUC	Area Under the Concentration-Time Curve
AUC_{inf}	AUC from the time of dosing extrapolated to time infinity
AUC_{inf} (%extrap)	percentage of AUC_{inf} due to extrapolation from the last measurable concentration to time infinity
AUC_{last}	AUC from the time of dosing up to the time of the last measurable concentration
AUC_{tau}	AUC from the time of dosing to the start of the next dosing interval at multiple dose conditions
CRF	Case Report Form
CRM	Continual Reassessment Method
CS	Classification Specifications
CSR	Clinical Study Report
C_{max}	maximum concentration
C_{trough}	Concentration immediately prior to dosing at multiple dosing
DLT	dose-limiting toxicity
5-FU	Fluorouracil
H	High
ICH	International Conference on Harmonization
L	Low
LLN	Lower Limit of Normal
LOCF	Last Observation Carried Forward
MedDRA	Medical Dictionary for Regulatory Activities
N	Normal
PD	Pharmacodynamics
PD1-x	Protocol Deviation 1-x
BMAS	Biomarker Analysis Set
PK	Pharmacokinetics
PKAS	Pharmacokinetics Analysis Set

**Abbreviations**	**Description of abbreviations**
PT	Preferred Term
PFS	Progression Free Survial
QTc	QT Interval Corrected
QTcF	QT Interval Corrected by Fridericia's Correction formula
OS	Overall Survial
SAF	Safety Analysis Set
SAP	Statistical Analysis Plan
SAS	Statistical Analysis Software
SBP	Systolic Blood Pressure
SOC	System Organ Class
$t_{1/2}$	Terminal Elimination Half-Life
TEAE	Treatment Emergent Adverse Event
TLF	Tables, Listings and Figures
ULN	Upper Limit of Normal
t_{max}	Time of Maximum Concentration
V_{ss}	Volume of Distribution at Steady State after Intravenous Dosing
V_Z	Volume of Distribution after Intravenous Dosing during the Terminal Elimination Phase
WHO-DD	World Health Organization Drug Dictionary

List of Key Terms

**Terms**	**Definition of terms**
Baseline	Assessments of subjects as they enter a trial before they receive any treatment.
Endpoint	Variable that pertains to the efficacy or safety evaluations of a trial.
Enroll	To register or enter a subject into a clinical trial. NOTE: Once a subject has received the study drug, the clinical trial protocol applies to the subject.
Intervention	The drug, device, therapy or process under investigation in a clinical study that is believed to have an effect on outcomes of interest in a study. (e.g., health-related quality of life, efficacy, safety, pharmacoeconomics).
Investigational period	Period of time where major interests of protocol objectives are observed, and where the test drug or comparative drug (sometimes without randomization) is usually given to a subject, and continues until the last assessment after completing administration of the test drug or comparative drug.
Post investigational period	Period of time after the last assessment of the protocol. Follow-up observations for sustained adverse events and/or survival are done in this period.
Randomization	The process of assigning trial subjects to treatment or control groups using an element of chance to determine assignments in order to reduce bias.
Screening	A process of active consideration of potential subjects for enrollment in a trial.
Screen failure	Potential subject who did not meet 1 or more criteria required for participation in a trial.
Screening period	Period of time before entering the investigational period, usually from the time when a subject signs the consent until just before the test drug or comparative drug (sometimes without randomization) is given to a subject.
Study period	Period of time from the first site initiation date to the last site completing the study.
Variable	Any entity that varies; any attribute, phenomenon or event that can have different qualitative or quantitative values.

1 INTRODUCTION

This Statistical Analysis Plan (SAP) contains technical and detailed elaboration of the principal features of the analysis described in the protocol, and includes procedures for executing the statistical analysis to fulfil the objectives of the study.

The final SAP will be approved prior to database hard-lock.

Changes from the planned analyses in the SAP will be documented in the Clinical Study Report (CSR).

2 STUDY OBJECTIVE(S) AND DESIGN

2.1 Study Objective(s)

2.1.1 Primary Objective

The primary objective is to determine the ORR of zolbetuximab as a single agent as assessed by an independent central reader.

2.1.2 Secondary Objectives

- To evaluate the pharmacokinetics of zolbetuximab as a single agent, in combination with mFOLFOX6, in combination with pembrolizumab and in combination with mFOLFOX6 and nivolumab, and in combination with FLOT.
- To evaluate pharmacokinetics of oxaliplatin and 5-FU in combination with zolbetuximab
- To assess the safety and tolerability of zolbetuximab as a single agent, in combination with mFOLFOX6 (with or without nivolumab), in combination with pembrolizumab, and in combination with FLOT
- To assess the immunogenicity of zolbetuximab as a single agent, in combination with mFOLFOX6 (with or without nivolumab), in combination with pembrolizumab, and in combination with FLOT
- To evaluate health-related quality of life (HRQoL)
- To assess ORR of zolbetuximab in combination with pembrolizumab and in combination with mFOLFOX6 as assessed by an independent central reader
- To assess ORR of zolbetuximab as a single agent, in combination with mFOLFOX6 (with or without nivolumab) based on investigator assessment
- To evaluate disease control rate (DCR), duration of response (DOR) and progression free survival (PFS) of zolbetuximab as a single agent and in combination with mFOLFOX6 based on independent central reader assessment
- To evaluate DCR, DOR and PFS of zolbetuximab as a single agent and in combination with mFOLFOX6 (with or without nivolumab) based on investigator assessment
- To assess OS of zolbetuximab as a single agent, in combination with mFOLFOX6 and nivolumab, and in combination with FLOT
- Cohort 5: To evaluate antitumor activity of zolbetuximab and FLOT as measured by radiological response (restaging) and pathological response (ypTNM)
- Cohort 5: To assess disease-free survival (DFS) of zolbetuximab in combination with FLOT
- Cohort 5: To assess minimal residual disease and disease recurrence

2.1.3 Exploratory Objective

- To evaluate potential genomic and/or other biomarkers that may correlate with treatment outcome of zolbetuximab as a single agent, in combination with mFOLFOX6 (with or without nivolumab), and in combination with FLOT
- To assess the effects of zolbetuximab as a single agent and in combination with mFOLFOX6 (with or without nivolumab) on CLDN18.2 expression
- To assess the immunomodulatory effects of zolbetuximab as a single agent, in combination with mFOLFOX6 (with or without nivolumab), and in combination with FLOT
- To evaluate the pharmacokinetics of pembrolizumab in combination with zolbetuximab
- To evaluate the pharmacokinetics of nivolumab in combination with zolbetuximab and mFOLFOX6

2.2 Study Design

This is a phase 2, open-label, multi-arm, non-randomized, multicenter study to assess the antitumor activity of zolbetuximab, an IgG1 chimeric monoclonal antibody directed against CLDN18.2, in subjects with metastatic or locally advanced unresectable gastric or GEJ adenocarcinoma and locoregional gastric or GEJ adenocarcinoma whose tumors are CLDN18.2-positive. For each cohort, the study consists of the following periods: pre-screening, screening, treatment and follow-up for radiographic disease progression (or post-treatment follow-up for disease recurrence, which will be conducted for Cohort 5). In addition, there will be a survival follow-up period for Cohorts 1A, 4B and 5 subjects only. Up to 25 centers located in North America, Europe and Asia Pacific will participate in this study for Cohorts 1-4. At least 50% of the subjects enrolled in Cohorts 1A and 2 must be from the US or Europe to ensure the study is regionally balanced. Up to 5 centers in North America and Japan will participate in this study for Cohort 5.

Please refer to protocol for details of study design for each cohort.

2.3 Randomization

This is a non-randomized study.

3 SAMPLE SIZE

The sample size of 20 for Cohort 1A is not based on a strict statistical power calculation, but is expected to provide adequate early efficacy and safety information.

The sample size of 12 for Cohort 2 is to provide sufficient pharmacokinetic information of zolbetuximab, oxaliplatin and 5-FU.

The sample size of 12 for Cohort 3A is not based on a statistical power calculation but is expected to provide safety information to determine the tolerability of the dose level of interest.

The sample size of 12 for Cohort 4A is not based on a statistical power calculation, but is expected provide safety information to determine the tolerability of the dose level of interest.

The sample size of approximately 65 for Cohort 4B is not based on a strict statistical consideration. The sample size of approximately 65 is expected to yield 50 subjects with high CLDN18.2 expression. For these subjects with high CLDN18.2 expression, assuming an accrual period of 12 months and a follow-up period of 3 to 6 months and 20 to 25 PFS events, the sample size of 50 will provide 70.37% to 76.13% power to detect the difference in PFS with the assumption of a 12 -month median PFS vs 8.5 -month median PFS (zolbetuximab in combination of nivolumab and mFOLFOX6 vs zolbetuximab and mFOLFOX6) using a 1-sided 15% Type I error. It is assumed that the survival time distributions of both groups are approximated reasonably well by the Weibull distribution with a shape parameter of 1. v

The sample size of 12 for Cohort 5 is not based on a statistical power calculation but is expected to provide safety information to determine the tolerability of the dose level of interest.

4 ANALYSIS SETS

In accordance with International Conference on Harmonization (ICH) recommendations in guidelines E3 and E9, the following analysis sets will be used for the analyses.

The determination of whether subjects are included or excluded from the safety and efficacy analysis sets will be made prior to database hard-lock.

The number and percentage of subjects will be characterized for all treated subjects and by each analysis set.

4.1 Full Analysis Set (FAS)

The FAS consists of all subjects who were enrolled and received at least 1 dose of zolbetuximab and who have at least 1 post-treatment disease assessment. At least 1 post-treatment disease assessment is defined as, for at least one time point, for either local or central assessment, the tumor imaging overall response does not equal to NE (not evaluable).

The FAS will be used for summaries of all efficacy data except PFS and OS , as well as selected demographic and baseline characteristics.

4.2 Safety Analysis Set (SAF)

The Safety Analysis Set (SAF) consists of all subjects who received at least 1 dose of zolbetuximab. The SAF will be used for summaries of demographic and baseline characteristics, PFS, OS, and all safety and tolerability related variables.

4.3 Pharmacokinetics Analysis Set (PKAS)

The Pharmacokinetics Analysis Set (PKAS) consists of the subset of the SAF for which at least 1 concentration data is available for any analyte. Additional subjects may be excluded

from the PKAS at the discretion of the pharmacokineticist. The PKAS is used for all tables and graphical summaries of the pharmacokinetics data.

4.4 Biomarker Analysis Set (BMAS) (Cohort 4B and 5 only)

The Biomarker Analysis Set (BMAS) consists of the subset of the SAF for which at least 1 pre-treatment or 1 on-treatment biomarker measurement is available. Additional subjects may be excluded from the BMAS at the discretion of the sponsor. The BMAS is used for all tables and graphical summaries of the biomarker data.

4.5 DLT Evaluation Analysis Set (DEAS)

The DLT (dose-limiting toxicity) Evaluation Analysis Set (DEAS) is defined as all subjects in the SAF excluding subjects without a DLT who receive less than the prescribed dose in cycle 1, or do not complete cycle 1 evaluations for a reason other than a DLT (e.g., consent withdrawal).

5 ANALYSIS VARIABLES

5.1 Efficacy Endpoints

The primary efficacy endpoint is objective response rate (ORR) of zolbetuximab as a single agent as assessed by an independent central reader. The secondary efficacy endpoints include duration of response (DOR), disease control rate (DCR) and Progression-free survival (PFS) by Response Evaluation Criteria in Solid Tumors (RECIST) 1.1, Overall Survival (OS), HRQoL, as well as the cohort 5-specific endpoints of Clinical Response, Pathological Response, Disease-free Survival(DFS) and Minimal Residual Disease.

5.1.1 Primary Efficacy Endpoint(s)

The ORR is defined as the proportion of subjects with complete or partial response based on best overall response (BOR). The primary efficacy endpoint, will be assessed by an Independent Central Reader according to RECIST v1.1.

BOR is determined once all tumor response data for the subject is available. Subject’s best response on study will be classified based on RECIST v1.1 criteria by independent central reader for primary efficacy endpoint. Timepoint responses after start of new ACT will not be used in determining BOR. BOR For best overall response of stable disease (SD), SD must be documented as present at least once after study entry.

5.1.2 Secondary Efficacy Endpoint

5.1.2.1 Disease Control Rate (DCR)

The disease control rate is defined as the proportion of subjects with CR, PR or SD based on best overall response (BOR). Subject’s best response on study will be classified based on RECIST v1.1.When SD is believed to be best response, the assessment should be at least 35 days from first dose date for Cohort 1 and 3, at least 56 days from the first dose for Cohort 2 and at least 49 days from the first dose for Cohort 4. For those with non-measurable disease

per central review at baseline, non-CR/non-PD will be included in DCR instead of SD. DCR will be summarized using the same method used for ORR.Duration of Response (DOR)

DOR is defined as the time from the date of the first response CR/PR (whichever is first recorded) to the date of radiological progression/death or date of censoring. DOR will be derived for subjects with response as CR or PR. If a subject has not progressed, the subject will be censored at the date of last evaluable radiological assessment or at the date of first CR/PR if no later evaluable radiological assessment is available. Other censoring used for the PFS analysis (see Table 1) will apply to DOR too.

To apply the cut-off date to DOR is to exclude those tumor assessments after cut-off date and anti-cancer therapy date after cut-off date in the analysis.

5.1.2.3 Radiologic Pesponse and Pathological Response

Radiological response will include CR and PR.

Pathological response (ypTNM) will include ypCR, ypPR and MPR (major pathologic response).

5.1.2.4 Progression-free Survival (PFS)

PFS is defined as the time from the start of the study treatment until death from any cause or radiological disease progression, whichever occurs first, assessed according to RECIST 1.1.

The PFS algorithm will be as follow:

Table 1

**Situation**	**Date of Event or Censoring**	**Outcome**
No baseline imaging assessments	Start Date of the study treatment	Censored
No evaluable post-baseline imaging assessments, no death	Start Date of study treatment	Censored
**Subject did not receive new anti-cancer therapy (ACT):**		
Radiological PD documented per RECIST v1.1	Date of first radiological PD (defined as earliest of date of scan showing new lesion if PD is based on new lesion or date of last scan of target lesions if PD is based on increase in sum of diameters (SOD) of target lesions)	Event
No radiological PD, but death recorded on eCRF	Date of death	Event
Neither radiological PD nor death	Date of last radiological assessment	Censored
Table continued on next page		

Situation	Date of Event or Censoring	Outcome
**Subject received new anti-cancer therapy (ACT)*:**		
Radiological PD per RECIST v1.1 or death documented only after start of new ACT	Date of last radiological assessment before start of new ACT	Censored
Radiological PD documented per RECIST v1.1 before start of new ACT	Date of first radiological PD	Event
No radiological PD nor death	Date of last radiological assessment before start of new ACT	Censored
**Missed >=2 scheduled radiological assessments:**		
If radiological PD or death occurs after missing 2 or more scheduled radiological assessments**	Date of last radiological assessment	Censored

Note: PFS = date of event or censoring – start date of study treatment + 1. NE will be treated as missing for the derivation described this table.

*New ACT includes new anti-cancer surgery, radiotherapy, chemo, immunotherapy and on study tumor directed procedure after start of study treatment.

**If the first radiological assessment after subject missed >=2 imaging assessments is SD or better and it's confirmed that subject did not take any other ACT during the missing period, the following imaging assessments will be used rather than censored.

To apply the cut-off date to PFS is to exclude those tumor assessments after cut-off date and anti-cancer therapy date after cut-off date in the analysis.

5.1.2.5 Disease-free Survival

DFS is defined as the time from date of treatment start until the date of radiological disease recurrence or until death due to any cause, whichever is earliest.

5.1.2.6 Overall Survival

OS is defined as the time from the date of treatment start until the documented date of death from any cause. All deaths will be included, regardless of whether death occurred while the subject is still taking study drug or after the subject discontinue study drug. Subjects who are still alive at the time of analysis will be censored at the last day known to be alive.

Table 2 OS Definition

Situation	Date of Event or Censoring	Outcome
Death before analysis cutoff date	Date of death	Event
Last known alive date is before cutoff date	Last known alive date	Censored
Death after analysis cutoff date	Analysis cutoff date	Censored
Last known alive date is after cutoff date	Analysis cutoff date	Censored

OS = Date of Event or Censor – Start date of study treatment+1

5.1.2.7 HRQoL

HRQoL are measured by the Quality of Life Questionnaire:

- Core Questionnaire (QLQ-C30)
- Oesophago-Gastric Module (OG-25)
- Global Pain (GP)
- EuroQOL Five Dimensions Questionnaire (EQ-5D)
- Health Resource Utilization (HRU) questionnaires.

The EORTC-QLQ-C30 is a cancer-specific instrument consisting of 5 functional domain scales: physical, role, emotional, social and cognitive. The EORTC-QLQ-OG25 instrument evaluates GC- and GEJC-specific symptoms such as stomach discomfort, difficulties eating and swallowing and indigestion. The Global Pain instrument is a single assessment of overall pain. The EQ-5D-5L is a standardized instrument for use as a measure of health outcome, consisting of 6 items that cover 5 main domains (mobility, self-care, usual activities, pain/discomfort, and anxiety/depression) and a general visual analog scale for health status.

5.1.2.8 Minimal Residual Disease

Minimal residual disease is measured by ctDNA.

5.2 Safety Variables

Safety and tolerability as noted by: DLT, AEs, infusion related reaction (IRR), serious AEs (SAEs), Adverse Events of Possible Hepatic Origin, laboratory test results (Hematology, Biochemisty, urinalysis, Serum pregnancy test, Coagulation profile), electrocardiograms (ECGs), vital signs,ECOG performance status, and other safety aspects are the secondary endpoint of this study.

5.2.1 Definition of DLT

Please refer to protocol Section 2.2.1 for details on DLT definition.

5.2.2 AE

AE will be assessed by evaluation of the following variables:

- Treatment-emergent adverse events (TEAEs; frequency, severity, seriousness, and relationship to study drug)
  - TEAE is defined as an adverse event observed after starting administration of the study drug and within 30 days after the last dose of study drug.
  - If the adverse event occurs on Day 1 and the onset check box is marked “Onset after first dose of study drug” or the onset check box is left blank, then the adverse event will be considered treatment emergent.
  - If the adverse event occurs on Day 1 and the onset check box is marked “Onset before first dose of study drug”, then the adverse event will not be considered treatment emergent.
  - If a subject experiences an event both during the pre-investigational period and during the investigational period, the event will be considered as TEAE only if it has worsened in severity (i.e., it is reported with a new start date).

    - Any AEs with onset dates completely missing will be considered TEAEs in summaries. AEs with partially missing onset dates will be assumed TEAEs unless the available portion of the date indicates that the onset was strictly before start of study medication.
    - A drug-related TEAE is defined as any TEAE with possible relationship to study treatment as assessed by the investigator or with missing assessment of the causal relationship
- Serious adverse events (SAEs) include adverse events that are flagged as serious by the investigator on eCRF, or upgraded by the Sponsor based on review of the Sponsor's list of Always Serious terms.

5.2.3 Clinical Laboratory Variables

Below is a table of the laboratory tests that will be performed during the conduct of the study. Refer to the Schedule of Assessments for study visit collection dates.

Additional laboratory tests should be performed according to institutional standard of care. Clinical significance of out-of-range laboratory findings is to be determined and documented by the investigator or delegated sub-investigator who is a qualified physician.

**Panel/Assessment**	**Parameters to be Analyzed**
Hematology	Hematocrit (Hct)<br>Hemoglobin (Hgb)<br>Red blood cell count (RBC)<br>White blood cell count (WBC)<br>WBC differential (absolute)<br>Platelets<br>Mean corpuscular volume (MCV)<br>Mean corpuscular hemoglobin (MCH)<br>Mean corpuscular hemoglobin concentration (MCHC)
Biochemistry (fasting not required)	Sodium (Na)<br>Magnesium (Mg)<br>Creatine phosphokinase (CK)<br>Potassium (K)<br>Calcium (Ca)<br>Chloride (Cl)<br>Phosphate (P)<br>Creatinine (Cr)<br>Glucose (Gl)<br>Blood urea nitrogen (BUN)<br>Alkaline phosphatase (ALP)<br>Aspartate aminotransferase (AST)<br>Alanine aminotransferase (ALT)<br>Lactate dehydrogenase (LDH)<br>Bilirubin total (TBL) (total and direct)<br>Total protein (TP)<br>Albumin (Alb)<br>Bicarbonate (HCO3)<br>Thyroid stimulating hormone (TSH)<br>Free thyroxine (T4)<br>Creatinine Clearance
Table continued on next page	

Panel/Assessment	Parameters to be Analyzed
Urinalysis	Color<br>Clarity/turbidity<br>Specific Gravity<br>Nitrites<br>Leukocyte esterase<br>RBCs<br>WBCs<br>Protein<br>Glucose<br>pH<br>Occult blood<br>Bilirubin<br>Urobilinogen
Urine Pregnancy Test	Human chorionic gonadotropin (HCG)
Serum Pregnancy Test	HCG
Coagulation Profile	Partial Thromboplastin Time (PTT)<br>Prothrombin time (PT)<br>International normalized ratio (INR)
Grade 3 or 4 Infusion-related Reaction (IRR)	Cytokine/Chemokine†
Any reaction with features of anaphylaxis	Serum total tryptase
Dihydropyrimidine dehydrogenase (DPD) deficiency screening†	DPD deficiency alleles

5.2.4 Vital Signs

Vital signs will include systolic and diastolic blood pressure (mmHg), radial pulse (beats/min) and temperature.

5.2.5 12-lead electrocardiogram (ECG)

A single 12-lead ECG will be performed at screening, at the zolbetuximab study drug discontinuation visit, zolbetuximab 30-day follow-up visit and if clinically indicated. A single 12-lead ECG will also be performed and assessed locally prior to every oxaliplatin infusion (before any antiemetic treatment) and following completion of every oxaliplatin infusion. Triplicate ECG will be collected in Cohort 1A at the time points outlined in the Schedules of Assessments [Protocol Table 2] to evaluate the potential of zolbetuximab to induce QT/corrected QT (QTc) interval prolongation. Additional ECG may be performed based on medical history and investigator medical judgment. All ECGs for Cohort 1A and 2 will be read centrally except for ECGs performed prior to and following completion of every oxaliplatin infusion, in which case a local read will be acceptable. All ECGs for Cohorts 3A, 4 and 5 will require a local read.

5.2.6 ECOG performance scores

ECOG performance scores will be collected.

5.2.7 Physical Examination

Physical examination will include the observation and review of all body systems and weight; height is only required at screening.

5.2.8 Other Safety Aspects

The following safety measures will be summarized for zolbetuximab and FLOT (Cohort 5):

a. Percentage of subjects with surgical complications
b. Percentage of subjects with surgical mortality as defined by death within 30
c. days of surgery
d. Percentage of subjects able to complete preoperative chemotherapy
e. Percentage of subjects with perioperative mortality and morbidity at 30 days
f. and 90 days post last dose
g. Percentage of subjects able to start postoperative chemotherapy
h. Percentage of subjects able to complete postoperative chemotherapy

5.3 Pharmacokinetics Variables

Serum concentrations of zolbetuximab, pembrolizumab and nivolumab, and plasma concentrations of oxaliplatin (measured as total and free platinum) and 5-FU will be evaluated.

The following pharmacokinetics parameters will be provided for zolbetuximab, oxaliplatin, 5-FU and pembrolizumab as applicable:

- **Cohort 1A**
  - Cycle 1 Day 1: zolbetuximab AUC_{inf}, AUC_{inf}(%extrap), AUC_{last}, AUC_{tau}, C_{max}, t_{max}, $t_{1/2}$, t_{last}, CL, V_{ss} and V_z, as appropriate. Note: Cycle 2 Day 1 predose sample of zolbetuximab will be used as the 504 hour postdose sample of Cycle 1 Day 1 when calculating pharmacokinetics parameters.
  - Cycle 3 Day 1: zolbetuximab AUC_{last}, AUC_{tau}, C_{max}, t_{max}, t_{last} as appropriate. Note: Cycle 4 Day 1 predose sample of zolbetuximab will be used as the 504 hour postdose sample of Cycle 3 Day 1 when calculating pharmacokinetics parameters.
  - All applicable visits: zolbetuximab predose samples as C_{trough}
- **Cohort 2**

  Zolbetuximab:

  - Cycle 1 Day 3: zolbetuximab AUC_{inf}, AUC_{inf}(%extrap), AUC_{last}, AUC_{tau}, C_{max}, t_{max}, $t_{1/2}$, t_{last}, CL, V_{ss} and V_z, as appropriate. Note: Cycle 1 Day 22 predose sample of zolbetuximab will be used as the 456 hour postdose sample of Cycle 1 Day 3 when calculating pharmacokinetics parameters.
  - Cycle 2 Day 1: zolbetuximab AUC_{last}, AUC_{tau}, C_{max}, t_{max}, t_{last} as appropriate. Note: Cycle 2 Day 22 predose sample of zolbetuximab will be used as the 504 hour postdose sample of Cycle 2 Day 1 when calculating pharmacokinetics parameters.

- All applicable visits: zolbetuximab predose samples as C_{trough}

Oxaliplatin (measured as total and free platinum):

- Day 1 in Cycles 1 and 2: AUC_{inf}, AUC_{inf}(%extrap), AUC_{last}, C_{max}, t_{max}, $t_{1/2}$, t_{last}, CL and V_z, as appropriate.

5-FU:

- Day 1 in Cycles 1 and 2: AUC_{last}, C_{max}, t_{max}, t_{last}, as appropriate. Additional parameters (e.g., AUC_{5h}, AUC_{24h}, AUC_{48h}) may be calculated if deemed helpful to understand the data.

- **Cohort 3**

Zolbetuximab:

- Cohort 3A, Cycle 1 Day 1: zolbetuximab AUC_{inf}, AUC_{inf}(%extrap), AUC_{last}, AUC_{tau}, C_{max}, t_{max}, $t_{1/2}$, t_{last}, CL, V_{ss} and V_z, as appropriate. Note: Cycle 2 Day 1 predose sample of zolbetuximab will be used as the 504 hour postdose sample of Cycle 1 Day 1 when calculating pharmacokinetics parameters.
- Cohort 3A, all applicable visits: zolbetuximab predose samples as C_{trough}

Pembrolizumab:

- Cohort 3A, all applicable visits: Pembrolizumab predose and end-of-infusion concentrations

- **Cohort 4**

Zolbetuximab:

- Cohort 4A, Cycle 1 Day 1: zolbetuximab AUC_{inf}, AUC_{inf}(%extrap), AUC_{last}, AUC_{tau}, C_{max}, t_{max}, $t_{1/2}$, t_{last}, CL, V_{ss} and V_z, as appropriate. Note: Cycle 1 Day 15 predose sample of zolbetuximab will be used as the 336 hour postdose sample of Cycle 1 Day 1 when calculating pharmacokinetics parameters.
- Cohorts 4A and 4B, all applicable visits: zolbetuximab predose samples as C_{trough}

Nivolumab:

- Cohorts 4A and 4B, all applicable visits: Nivolumab predose and end-of-infusion concentrations

5.4 Pharmacodynamics Variables

Pharmacodynamics variables that are of interest may be explored. Variables for other exploratory biomarkers will be summarized in separate exploratory biomarker analysis plan.

5.5 Other Variables

- Changes in tumor expression of CLDN18.2
- Changes in immune-related biomarkers in tumor tissue and blood samples

- Immunogenicity of zolbetuximab as measured by the frequency of ADA positive subjects.
- Body Mass Index (BMI)

  BMI = weight (kg) / [height (m)]^2
- Duration of treatment
  Duration of treatment of a study drug will be calculated in days, using the following formula:
  (Date of last dose – date of first dose) + 1

  When the start or stop date is missing, then the exposure will be treated as missing.

- Number of infusions
  For the interruptions that involve overnight infusion, only one infusion will be counted
- Number of infusion entirely administered
  Number of infusions captured on dosing CRF with entire infusion administered checked as Yes.

- Infusion time (min/infusion)
- Infusion time captured on dosing CRF, calculated as (stop time – start time). Interruption time is included in the infusion time if the infusion were finished within one day; for interruptions that goes overnight, interruption time will not be includedPlanned Cumulative Dose
  Protocol specified planned cumulative dose

- Actual Cumulative Dose
  Sum of actual delivered dose captured on dosing CRF across the days when study drug was administered.

- Relative Dose Intensity (RDI)

  Actual Cumulative Dose
  ----------------------------- x 100
  Planned Cumulative Dose

6 STATISTICAL METHODOLOGY

6.1 General Considerations

In general, all data will be summarized by cohort (cohort 1,cohort 2, cohort 3A, cohort 4A, cohort 4B, and cohort 5) with descriptive statistics for continuous endpoints, and frequency and percentage for categorical endpoints, with the exception of demographic, disposition and baseline characteristics. Demographic, disposition and baseline characteristics will be summarized by cohort and overall. Kaplan-Meier estimates will be provided for time-to-event endpoints.

Baseline will be defined as the last observation prior to first dose, unless otherwise specified.

For continuous variables, descriptive statistics will include the number of subjects (n), mean, standard deviation, median, minimum and maximum. When needed, the use of other percentiles (e.g.,10%, 25%, 75% and 90%) will be mentioned in the relevant section. In addition, for summaries of PK data, descriptive statistics will include CV, geometric mean and geometric CV. Frequencies and percentages will be displayed for categorical data. Percentages by categories will be based on the number of subjects with no missing data, i.e., will add up to 100%.

Summaries will be presented by planned treatment group at the time of enrollment, unless specifically stated otherwise.

All data processing, summarization, and analyses will be performed using SAS® Version 9.3 or higher on Unix. Specifications for table, figures, and data listing formats can be found in the TLF specifications for this study.

Study day will be calculated in reference to the first dose date. For assessments conducted before the first dose, study day will be calculated as (assessment date – first dose date). For assessments conducted on or after the first dose, study day will be calculated as (assessment date – first dose date + 1).

For BOR, ORR , DOR and DCR, result for both confirmed and confirmed plus unconfirmed will be presented, Although confirmed is the primary endpoint.

For the definition of subgroups of interest, please refer to Section 6.7.

6.2 Study Population

6.2.1 Disposition of Subjects

The following subject data will be presented:

- Number of subjects screen failed
- Number of subjects with informed consent, discontinued before treatment and allocated to treatment , overall only;
- Number and percentage of subjects allocated to treatment in each analysis set, by cohort and overall;
- Number and percentage of subjects completed and discontinued zolbetuximab, by primary reason for treatment discontinuation for FAS and SAF, by cohort and overall ;
- Number and percentage of subjects completed and discontinued last component of mFOLFOX6, by primary reason for treatment discontinuation for FAS and SAF, for cohort 2, 4A, and 4B;
- Number and percentage of subjects completed and discontinued last component of Pembrolizumab, by primary reason for treatment discontinuation for FAS and SAF, for cohort 3A;

- Number and percentage of subjects completed and discontinued last component of Nivolumab by primary reason for treatment discontinuation for FAS and SAF, for cohort 4A and 4B;
- Number and percentage of subjects completed and discontinued last component of FLOT by primary reason for treatment discontinuation for FAS and SAF, for cohort 5;
- Number and percentage of subjects completed and discontinued the study at 30 day Follow up for zolbetuximab, by primary reason for study discontinuation for SAF by cohort and overall;
- Number and percentage of subjects completed and discontinued the study at 30 day Follow up for last component of mFOLFOX6, by primary reason for study discontinuation for SAF for cohort 2, 4A, and 4B;
- Number and percentage of subjects completed and discontinued the study at 30 day Follow up for last component of Pembrolizumab, by primary reason for study discontinuation for SAF for cohort 3A;
- Number and percentage of subjects completed and discontinued the study at 30 day Follow up for last component of Nivolumab, by primary reason for study discontinuation for SAF for cohort 4A and 4B; Number and percentage of subjects completed and discontinued the study at 30 day Follow up for last component of FLOT, by primary reason for treatment discontinuation for SAF for cohort 5;
- Number and percentage of subjects completed and discontinued the study at 90 day Follow up for zolbetuximab, by primary reason for study discontinuation for SAF by cohort and overall;
- Number and percentage of subjects completed and discontinued the study at 90 day Follow up for last component of mFOLFOX6, by primary reason for study discontinuation for SAF for cohort 2, 4A, and 4B;
- Number and percentage of subjects completed and discontinued the study at 90 day Follow up for last component of Pembrolizumab, by primary reason for study discontinuation for SAF for cohort 3A;
- Number and percentage of subjects completed and discontinued the study at 90 day Follow up for last component of Nivolumab, by primary reason for study discontinuation for SAF for cohort 4A and 4B;
- Number and percentage of subjects completed and discontinued the study at 90 day Follow up for last component of FLOT, by primary reason for treatment discontinuation for SAF for cohort 5;
- Number and percentage of subjects completed and discontinued the study at End of Post-Treatment Follow up, by primary reason for study discontinuation for SAF for cohort 1A, cohort 2 ,cohort 4 and cohort 5;
- Number and percentage of subjects completed and discontinued during survival follow up, by primary reason for post-study period discontinuation for SAF for cohort 1 ,cohort 4B and cohort 5;

6.2.2 Protocol Deviations

The number and percentage of subjects with the following protocol deviation criteria will be summarized for each criterion and overall, by cohort and total as well as by investigative site. Subjects deviating from a criterion more than once will be counted once for the corresponding criterion.

The unique identifiers will be as follows:

PD1 - Entered into the study even though they did not satisfy entry criteria,

PD2 - Developed withdrawal criteria during the study and was not withdrawn,

PD3 - Received wrong treatment or incorrect dose,

PD4 - Received excluded concomitant treatment.

6.2.3 Demographic and Other Baseline Characteristics

Demographic and other baseline characteristics will be summarized descriptively by cohort and total group for FAS.

Number and percentage of subjects allocated to treatment in each country and site will be presented by cohort and overall for the SAF.

Descriptive statistics for age, weight, body mass index (BMI) and height at study entry will be presented. Frequency tabulations for sex, ethnicity, race, tobacco history, age categories (<=65 years, >65 years and <=75 years, >75 years), tumor history, tumor location, CLDN18.2 categories (>=50 but <75% and >=75%) and HER2 status will be presented. This will be done for SAF and FAS by cohort and overall.

Medical history is coded in MedDRA, and will be summarized by System Organ Class (SOC) and Preferred Term (PT) as well as by PT alone, by cohort and overall for the SAF.

Baseline conditions are defined as those ongoing at the time of informed consent or arising following the time of informed consent and before the first dose of study drug. For ongoing medical conditions, Common Terminology Criteria for Adverse Events (NCI-CTCAE) grade will be provided in listing.

6.2.4 Previous and Concomitant Medications

Previous medications are coded with WHO-DD, and will be summarized by therapeutic subgroup (ATC 2nd level) and chemical subgroup (ATC 4th level) and preferred WHO name by cohort and overall for the SAF.

As with previous medication, concomitant medication will be summarized for each cohort and overall by therapeutic subgroup (ATC 2nd level) and chemical subgroup (ATC 4th level) and preferred WHO name for the SAF. Subjects taking the same medication multiple times will be counted once per medication and investigational period. A medication which can be classified into several chemical and/or therapeutic subgroups is presented in all chemical and therapeutic subgroups.

Previous medications are defined as medications that patients started prior to first administration of study medication. Concomitant medications are defined as any medications that patients took after the first dose of study medication and through 90 days from last dose of study drug. Medications that started prior to first administration of study drug and continued while study drug was given will be counted in both previous and concomitant medications.

6.2.4.1 Prior Cancer Therapy

Frequency tabulations of subjects with prior cancer therapy will be presented for SAF.

6.2.4.2 Prior Radiation Therapy

Frequency tabulations of subjects with prior radiation therapy will be presented for SAF.

6.2.4.3 Prior Procedures for Primary Cancer

Frequency tab ulations of subjects with prior procedures for primary cancer will be presented for SAF.

6.2.4.4 New Anti-Cancer therapy

New Anti-Cancer therapy will be listed.

6.3 Study Drugs

Study Drug exposure will be presented separately for each component of study drug in general.

6.3.1 Exposure

The following information on drug exposure will be presented for the SAF for zolbetuximab, Pembrolizumab, Nivolumab and each component of mFOLFOX6 and FLOT separately in general.

- Duration of Treatment
- Number of Infusions (Zolbetuximab, Pembrolizumab, Nivolumab,each component of mFOLFOX6 and FLOT, mFOLFOX6 and FLOT as a regimen)
- Average Infusion Time per Infusion
- Planned Cumulative Dose
- Actual Cumulative Dose
- Relative Dose intensity
- RDI categories: <50, 50 to 80 inclusive, >80
- Number of Dose Adjustments
- Number of Dose interruptions
- Reasons for dose adjustment
- Reasons for dose interruptions

Dose increase and dose reduction for zolbetuximab and nivolmab are not allowed. Number and percent of subject with dose adjustments or interruptions and reasons for dose adjustments, interruptions and delays will be presented when applicable.

6.3.2 Relative Dose Intensity

RDI will be examined for subjects in the SAF whose first and last days of treatment are known.

RDI will be summarized in two ways for the SAF:

- Descriptive statistics will be presented
- RDI will be categorized according to the following categories:
  - less than 50%
  - at least 50%, less or equal to 80%
  - greater than 80%
  - Unknown

6.4 Analysis of Efficacy

Efficacy analysis will be conducted on the FAS. Tumor related analyses are summarized based on RECIST 1.1.

6.4.1 Analysis of Primary Endpoint(s)

ORR with confirmed response will be summarized using exact 95% confidence interval for each cohort. Best overall response is determined once all tumor response data for the subject is available.

SAS code for constructing 95% confidence interval for ORR by Clopper-Pearson method:

```
proc freq;
   tables orr/binomial;
   exact binomial;
run;
```

6.4.2 Analysis of Secondary Endpoints

6.4.2.1 Disease Control Rate (DCR)

DCR will be summarized using the same method used for ORR.

6.4.2.2 Best Overall Response

BOR based on RECIST 1.1 will be summarized.

A confirmed response means that for CR and PR RECIST 1.1 criteria must be met again on confirmatory scans done at least 4 weeks after the date of the scan on which CR or PR was first observed.

Percent change in tumor size will be calculated for subjects with measurable disease at baseline. The percent change by time in tumor size will be visually presented using a spider plot. The best percent change in tumor size will be visually presented using a waterfall plot.

6.4.2.3 Duration of Response (DOR)

DOR will be calculated only for the subgroup of subjects with response CR/PR. The distribution of DOR will be estimated for each cohort using Kaplan-Meier methodology.

Median, 1st Quartile and 3rd Quartile of DOR and their 95% confidence interval will be provided. Kaplan-Meier plot for DOR will be provided.

6.4.2.4 Clinical Response and Pathological Response

For zolbetuximab and FLOT (Cohort 5), the clinical response to preoperative chemotherapy (timepoints include baseline tumor assessment [prior to study treatment] to before surgery and after completion/discontinuation of preoperative chemotherapy) will be summarized using the following:

1. Percentage of subjects with radiological response at restaging will be summarized using exact 95% confidence interval.
2. Percentage of subjects with pathological response (ypTNM) will be summarized using exact 95% confidence intervals.

6.4.2.5 Progression-free Survival

The survival curve and the median, 1st Quartile and 3rd Quartile of PFS will be estimated using Kaplan-Meier method and will be reported along with corresponding 95% confidence interval for each cohort. Kaplan-Meier plot for PFS will be provided.

In addition, PFS rate and its 95% confidence interval will be estimated at 3 months, 6 months, 9 months, and 12 months using Kaplan-Meier method for each cohort.

6.4.2.6 Disease-free Survival

DFS will be analyzed using the same method as PFS (i.e., Kaplan-Meier method) for each cohort.

6.4.2.7 Overall Survival

OS will be analyzed using the same method as PFS (i.e., Kaplan-Meier method) for each cohort.

In addition, OS rate and its 95% confidence interval will be estimated at 6 months, 9 months, 12 months and 18 month using Kaplan-Meier method foreach cohort with survival follow-up as part of the schedule of assessment.

6.4.2.8 HRQoL

HRQoL will be summarized by descriptive statistics by the QLQ-C30, OG-25, GP, EQ-5D and HRU questionnaires for each cohort. The change from baseline to final visit or early termination will also be summarized.

In addition, the EORTC QLQ-C30 and OG25 individual symptom scores will be categorized as the following:

a. For questions which have a 4 point scales with 1 being the best, and 4 being the worst, categorized as 1-2=”None/Slight”, 3=”Moderate”, 4= “Severe”.

b. For QLQ-C30 questions #29 and #30 which are 7-points overall ratings please classify as follows: 1-2=”Severe impairment”, 3-5=”Moderate impairment”, 6-7=”No or slight impairment”

The EQ-5D-5L scores (on 1-5 scale) will be categorized as: 1-2=”None/Slight”, 3=” Moderate”, 4-5=”Severe/Extreme”.

6.4.2.9 Minimal Residual Disease

Descriptive statistics will be used to summarize results and change from baseline by time point.

6.4.3 Analysis of Exploratory Endpoints

6.4.3.1 Tumor Expression of CLDN18.2

Tumor expression of CLDN18.2 measured by IHC in screening sample (prescreening and screening) will be summarized for each cohort.

6.4.3.2 Immune related biomarkers in tumor tissue and blood

Details will be provided in separate biomarker SAP.

6.4.3.3 Other Biomarker

Details will be provided in separate biomarker SAP.

6.4.4 Imaging

A listing of tumor imaging data will be provided.

6.5 Analysis of Safety

6.5.1 Dose Limiting Toxcicities

A DLT event, as defined in protocol Section 2.2, will be summarized by dose level using DEAS for cohort 3A, 4A and 5. Details of DLTs will be presented in listings and subject narratives. DLT rate will be presented for all DLT during DLT observation period for each dose level in cohort 3A, 4A and 5.

6.5.2 Adverse Events

AEs will be coded using the Medical Dictionary for Regulatory Activities (MedDRA) and graded using NCI-CTCAE. MedDRA Version used will be presented in the title of the related TLFs.

TEAE is defined as an AE observed after starting administration of the study treatment and within 30 days after the last dose of the last administered component of study treatment. Serious TEAE summaries include both investigator-assessed and Astellas upgraded SAEs.

Separate summaries for AE/SAE related to any component of the study drug and AE/SAE related to each component of study drug will be provided for Drug-related AE/SAE summaries.

An overview table will include the following details by cohort:

- Number and percentage of subjects with TEAEs,
- Number and percentage of subjects with drug related TEAEs,
- Number and percentage of subjects with serious TEAEs
- Number and percentage of subjects with serious drug related TEAEs
- Number and percentage of subjects with TEAEs leading to permanent discontinuation of any component of study drug and by component
- Number and percentage of subjects with drug related TEAEs leading to permanent discontinuation of any component of study drug and by component
- Number and percentage of subjects with TEAEs leading death
- Number and percentage of subjects with drug related TEAEs leading to death
- Number and percentage of subjects with Grade >=3 TEAEs
- Number and percentage of subjects with drug related Grade >=3 TEAEs

- Number of deaths.

The number and percentage of subjects with TEAEs, as classified by SOC and PT will be summarized by cohort. Summaries will be provided for:

- DLT within DLT observation period
- TEAEs
- Drug related TEAEs
- AEs collected after 30 days post last dose of study drug,
- Serious TEAEs,
- Serious AEs collected after 30 days post last dose of study drug,
- Drug-Related Serious TEAE
- TEAE Leading to Permanent Discontinuation of any component of study drug and by component
- Drug Related TEAE Leading to permanent Discontinuation of any component of study drug and by component
- TEAE Leading to Dose Interruption of any component of study drug and by component
- Drug-Related TEAE Leading to Dose Interruption of any component of study drug and by component
- TEAE with NCI-CTCAE >=3
- Drug related TEAE with NCI-CTCAE >=3
- TEAE Leading to Death
- Drug-Related TEAE Leading to Death
- Common TEAE: Frequently Reported (>=10% in at least one Cohort)
- Common TEAE excluding SAE that equal to or exceed a threshold 10% in at least one cohort

The number and percentage of subjects with TEAEs , Serious TEAE and TEAE leading to death, as classified by PT only, will be summarized by cohort.

AE summary tables will include subject counts as opposed to AE counts except for SAE and TEAE leading to death. SAE and TEAE leading to death summary table will include subject counts as well as AE counts. If a subject experiences more than one episode of a particular AE, that subject will be counted only once for that event. If a subject has more than one AE that code to the same preferred term, the subject will be counted only once for that preferred term. Similarly, if a subject has more than one AE within a body system, the subject will be counted only once in that body system.

TEAEs and the number and percentage of subjects with TEAEs, as classified by SOC and PT will also be summarized by NCI-CTCAE severity grade and by relationship to study drug If an adverse event changes in severity grade or relationship, then the subject will be counted only once with the worst severity grade and highest degree of relationship. Then, the adverse event will be presented in each category they were classified to. If a subject has an event more than once with missing severity grade and with non-missing severity grade, then the subject will be counted as the highest non-missing grade. If a subject has an event with all missing severity grade, then the subject will be counted under the missing category for severity grade. If a subject has an event more than once with missing relationship and with non-missing relationship, or if a subject has an event more than once with all missing relationship, the subject will be counted as “related”. Drug related TEAEs will be presented in a similar way by severity only.

All AEs, deaths, SAEs and withdrawals due to adverse events will be displayed in listings.

All AEs occurring during or after the subject has discontinued the study are to be followed up until resolved or judged to be no longer clinically significant, or until they become chronic to the extent that they can be fully characterized.

6.5.3 AE of Special interest

The list of adverse events of interest to be summarized may change during the course of the study due to ongoing pharmacovigilance. AE of special interest will include:

- Nausea or vomiting or abdominal pain based on PT terms
- Nausea or vomiting based on PT terms
- Hypersensitivity reactions based on HS SMQ
- Infusion-related reactions (IRR)
  - infusion-related reactions (IRRs) flagged by investigators
  - potential IRR defined as all AE that have a start date the same as a study treatment day
- Anemia
- Neutropenia

The number and percentage of subjects with AESI(AE of Special Interest), as classified by SOC and PT will be summarized. Summaries will be provided for:

- AESI
- Serious AESI
- AESI by NCI-CTCAE Grade

- AESI leading to permanent discontinuation
- AESI leading to dose interruption
- AESI leading to death

6.5.4 Laboratory Assessments

The baseline visit is the last measurement taken prior to initial study drug administration.

Quantitative clinical laboratory variables, i.e., hematology, biochemistry , urinalysis and coagulation profile will be summarized using mean, standard deviation, minimum, maximum and median by cohort at each visit. Additionally, a within-subject change will be calculated as the post-baseline measurement minus the baseline measurement and summarized in the same way.

Frequency tabulations of qualitative clinical laboratory variables for urine analysis as appropriate will be presented by cohort at each visit.

Laboratory results will be graded using NCI-CTCAE, where possible. NCI-CTCAE grade derivation will be using grade definition in CTCAE handbook. NCI-CTCAE grade of laboratory evaluations will be summarized by number and percentage of subjects for each visit. Shift tables of NCI-CTCAE grade change from baseline to worst post-baseline grade will also be presented. Parameters that have criteria available for both low and high values, i.e., hypo- and hyper-, will be summarized for both criteria separately. The same subject can be counted for both values if the subject has different laboratory values meeting each criterion. Baseline NCI-CTCAE grade is the last non-missing grade before first dose.

Potentially clinically significant values in liver enzymes and total bilirubin will be summarized. Details are provided in Section 6.5.4.1.

The list of laboratory parameters to be summarized may change during the course of the study due to ongoing pharmacovigilance. It will be finalized before the database hard lock.

Laboratory data will be displayed in listings.

6.5.4.1 Liver Safety Assessment

The liver safety assessments will be summarized by the following categories below based on the measurements from Alkaline Phosphatase (ALP), Alanine Transaminase (ALT), total bilirubin, Aspartate Transaminase (AST) and their combination are defined. The subject's highest value during the investigational period will be used.

Parameter	Criteria
ALT	> 3xULN<br>> 5xULN<br>> 10xULN<br>> 20xULN
AST	> 3xULN<br>> 5xULN<br>> 10xULN<br>> 20xULN
ALT or AST	> 3xULN<br>> 5xULN<br>> 10xULN<br>> 20xULN
Total Bilirubin	> 2xULN
ALP	> 1.5xULN
ALT and/or AST AND Total Bilirubin(*)	(ALT and/or AST > 3xULN) and total bilirubin > 2xULN
ALT and/or AST AND Total Bilirubin(*)	(ALT and/or AST > 3xULN) and total bilirubin > 2xULN and ALP<2x ULN

(*) Combination of values measured within same day or within 1 day apart

The number and percentage of subjects with potentially clinically significant values in liver enzyme and total bilirubin tests during the investigational period will be presented by cohort.

6.5.5 Vital Signs

The baseline visit is the last measurement taken prior to initial study drug administration.

Vital signs (systolic blood pressure, diastolic blood pressure, pulse rate and body temperature) will be summarized using mean, standard deviation, minimum, maximum and median by cohort, and by visit. Additionally, a within-subject change will be calculated per visit as the post-baseline measurement minus the baseline measurement and summarized by cohort and visit.

Tables for potentially clinically significant vital signs will be generated using baseline value and highest value obtained during treatment for each subject for each cohort.

A separate set of tables may be generated for the first 24 hours measurement

Vital signs data will be displayed in listings

The following potentially clinically significant criteria are defined for each parameter:

Vital Sign Variable	Criteria
SBP	≥180 mmHg AND ≥20 mmHg change from baseline
SBP	≤ 80 mmHg
DBP	≥105 mmHg AND ≥15 mmHg change from baseline
Pulse Rate	≥120 bpm AND ≥15 bpm change from baseline

6.5.6 Electrocardiogram

A single 12-lead ECG will be performed at screening, at the zolbetuximab study drug discontinuation visit, zolbetuximab 30-day follow-up visit and if clinically indicated. A single 12-lead ECG will also be performed and assessed locally prior to every oxaliplatin infusion (before any antiemetic treatment) and following completion of every oxaliplatin infusion. Triplicate ECG will be collected in Cohort 1A at the time points outlined in the Schedules of Assessments to evaluate the potential of zolbetuximab to induce QT/corrected QT (QTc) interval prolongation. All ECGs for Cohort 1A and 2 will be read centrally except for ECGs performed prior to and following completion of every oxaliplatin infusion, in which case a local read will be acceptable. All ECGs for Cohorts 3A and 4 will require a local read.

For triplicate ECGs, the three values of each ECG parameter within a time point for a subject will be averaged before used in analysis and summaries.

ECG variables (including triplicate and single ECGs) will be summarized using mean, standard deviation, minimum, maximum and median for SAF for each cohort at each treatment visit and time point, including changes from baseline.

Number and percentage of subjects with normal and abnormal results for the overall interpretation will be tabulated by cohort at each treatment visit and time point for centrally assessed data. And Number and percentage of subjects with normal, abnormal-not clinically significant and abnormal-clinical significant results for the interpretation will be tabulated by cohort at each treatment visit and time point for locally assessed data. A shift analysis table showing shift in overall ECG interpretation from baseline to each time point will be provided. For triplicate ECGs, the worst of the three overall ECG interpretations will be used as the time-specific overall ECG interpretation for a subject. Percent of subjects on different kind of abnormality will also be reported.

The QT interval corrected by Fridericia's Correction formula (QTcF interval) will be summarized using frequency tables for values of clinical importance using the range criteria below.

	**QTcF Interval Criteria Value (msec)**
Normal	≤ 450
Borderline	> 450 to <=480
Prolonged	> 480 to <=500
Clinically significant	> 500

QTcF interval: Fridericia-corrected QT interval

The QTcF interval will also be summarized by the frequencies of subjects with a change from baseline of clinical importance using the criteria identified below.

**Variable**	**Change from Baseline**
QTcF Interval (msec)	<0<br>>=0 to <=30<br>>30 to <=60<br>> 60

QTcF interval: Fridericia-corrected QT interval

All ECG data (individual replicates and the averages) will be displayed in listings.

Effects of serum concentrations of zolbetuximab on ΔQTcF (defined as the mean change from baseline in QTcF) will be assessed. Analysis for relationship between zolbetuximab concentrations and QTc will be performed by, or under the direct auspices of, Pharmacokinetics, Modeling and Simulation, Astellas. The report will be provided in a separate memo.

6.5.7 Eastern Cooperative Oncology Group Performance Status

Summary statistics (number and percent of subjects) for each category of the ECOG performance status at each assessment will be provided. The change from baseline to final visit or early termination will also be summarized. Negative change scores indicate an improvement. Positive scores indicate a decline in performance. Shift tables of ECOG performance status change from baseline to worst post-baseline grade will also be presented.

6.5.8 Pregnancies

A detailed listing of all pregnancies will be provided.

6.6 Analysis of PK

Descriptive statistics will include the number of subjects (n), mean, SD, coefficient of variation (CV), geometric mean, geometric CV, median, minimum, maximum. For the pharmacokinetics parameters t_{max} and $t_{last,}$ only n, median, minimum and maximum will be calculated.

6.6.1 Concentrations

Serum concentrations of zolbetuximab, pembrolizumab and nivolumab, and plasma concentrations of oxaliplatin (measured as total and free platinum) and 5-FU will be listed and summarized using descriptive statistics by scheduled time point for each cohort and cycle as applicable. Standard graphics including mean serum or plasma concentration-time profiles,

overlay (spaghetti) plots and individual subject serum or plasma concentration-time profiles will be produced.

6.6.2 Estimation of Pharmacokinetics Parameters

Noncompartmental analysis will be used for the calculation of serum and plasma pharmacokinetics parameters using Phoenix version 6.3 or higher (Certara L.P., 100 Overlook Center Suite 101, Princeton, NJ 08540, US).

Serum pharmacokinetics parameters of zolbetuximab and plasma parameters of oxaliplatin (measured as total and free platinum) and 5-FU will be listed and summarized using descriptive statistics for each cohort and cycle as applicable.

6.6.3 Statistical Analysis of Pharmacokinetics Parameters

To evaluate potential effect of zolbetuximab on the pharmacokinetics of oxaliplatin and 5-FU in Cohort 2, the geometric least-squares mean ratio (cycle 2 day 1 vs. cycle 1 day 1) and associated 90% confidence intervals will be provided for C_{max} and AUCs of total platinum, free platinum and 5-FU for Cohort 2. If deemed appropriate, dose-normalized C_{max} and AUCs will be calculated and used in the assessment.

[Steady-state assessment]

Serum trough concentrations of zolbetuximab (C_{trough}) measured over time will be plotted, and the visual assessment of steady-state will be conducted. Similar plots and assessment may be done for pembrolizumab and nivolumab.

6.6.4 Immunogenicity

Immunogenicity of zolbetuximab will be summarized using the frequency of ADA positive subjects for each cohort.

The potential relationship between zolbetuximab immunogenicity and zolbetuximab pharmacokinetics, efficacy and safety profiles in subjects may be assessed.

6.7 Subgroups of Interest

Primary and secondary efficacy endpoint will be summarized by cohort for the subgroups defined on the basis of the categorized variables listed below:

Grouping variable	Subgroups
Gastrectomy	Yes/No
Tumor type	Diffuse/Intestinal/Mixed or other
CLDN 18.2	High/Intermediate

6.8 Interim Analysis (and Early Discontinuation of the Clinical Study)

No interim analysis will be performed.

6.9 Additional Conventions

6.9.1 Missing Data

Every effort will be made to resolve missing or incomplete dates for adverse events and concomitant medications. If a partial date cannot be resolved, the most conservative imputation methods will be used to complete the missing information. As a general rule, if the month or year is missing, imputation should be avoided if possible. More details on date imputation, if needed, would be placed in the TLF specifications.

6.9.2 Outliers

All values will be included in the analyses.

6.9.3 Visit Windows

Not applicable. Nominal visits will be used in the by visit summary. Values from unscheduled visits will be included in the summary of extreme cases (e.g., summary of worst post-baseline, summary of minimum post-baseline, summary of maximum post-baseline). For efficacy endpoints, all values (scheduled and unscheduled) will be included in the analysis.

7 DOCUMENT REVISION HISTORY

Version	Date	Changes	Comment/rationale for change
1.00	21 May 2018	NA	Document finalized
1.1	15 April 2020	To add cohort 3	Protocol amendment
2.0	15 April 2021	Added cohort 4	Protocol amendment
3.0	22 Augest 2024	Revision of cohort 4B and adding cohort 5	Protocol amendment 7, version 8.

8 REFERENCES

Hwang IK, Shih WJ, De Cani JS. Group sequential designs using a family of type I error probability spending functions. Stat Med. 1990 Dec; 9(12):1439-45.

Zhou H, Lee JJ, Yuan Y. BOP2: Bayesian optimal design for phase II clinical trials with simple and complex endpoints. Stat Med. 2017;36(21):3302-14.

9 APPENDICES

9.1 EORTC QLQ-C30 questionnaire (version 3)

		Not at All	A Little	Quite a Bit	Very Much
1.	Do you have any trouble doing strenuous activities, like carrying a heavy shopping bag or a suitcase?	1	2	3	4
2.	Do you have any trouble taking a long walk?	1	2	3	4
3.	Do you have any trouble taking a short walk outside of the house?	1	2	3	4
4.	Do you need to stay in bed or a chair during the day?	1	2	3	4
5.	Do you need help with eating, dressing, washing yourself or using the toilet?	1	2	3	4

During the past week:

		Not at All	A Little	Quite a Bit	Very Much
6.	Were you limited in doing either your work or other daily activities?	1	2	3	4
7.	Were you limited in pursuing your hobbies or other leisure time activities?	1	2	3	4
8.	Were you short of breath?	1	2	3	4
9.	Have you had pain?	1	2	3	4
10.	Did you need to rest?	1	2	3	4
11.	Have you had trouble sleeping?	1	2	3	4
12.	Have you felt weak?	1	2	3	4
13.	Have you lacked appetite?	1	2	3	4
14.	Have you felt nauseated?	1	2	3	4
15.	Have you vomited?	1	2	3	4
16.	Have you been constipated?	1	2	3	4

Please go on to the next page

**During the past week:**	**Not at All**	**A Little**	**Quite a Bit**	**Very Much**
17. Have you had diarrhea?	1	2	3	4
18. Were you tired?	1	2	3	4
19. Did pain interfere with your daily activities?	1	2	3	4
20. Have you had difficulty in concentrating on things, like reading a newspaper or watching television?	1	2	3	4
21. Did you feel tense?	1	2	3	4
22. Did you worry?	1	2	3	4
23. Did you feel irritable?	1	2	3	4
24. Did you feel depressed?	1	2	3	4
25. Have you had difficulty remembering things?	1	2	3	4
26. Has your physical condition or medical treatment interfered with your family life?	1	2	3	4
27. Has your physical condition or medical treatment interfered with your social activities?	1	2	3	4
28. Has your physical condition or medical treatment caused you financial difficulties?	1	2	3	4

For the following questions please circle the number between 1 and 7 that best applies to you

29. How would you rate your overall health during the past week?

1	2	3	4	5	6	7
Very poor						Excellent

30. How would you rate your overall quality of life during the past week?

1	2	3	4	5	6	7
Very poor						Excellent

9.2 EORTC QLQ-30 Scoring

Scoring the EORTC QLQ-C30 version 3.0

Table 1: Scoring the QLQ-C30 version 3.0

	Scale	Number of items	Item range*	**Version 3.0** Item numbers	Function scales
**Global health status / QoL**					
Global health status/QoL (revised)†	QL2	2	6	29, 30	
**Functional scales**					
Physical functioning (revised)†	PF2	5	3	1 to 5	F
Role functioning (revised)†	RF2	2	3	6, 7	F
Emotional functioning	EF	4	3	21 to 24	F
Cognitive functioning	CF	2	3	20, 25	F
Social functioning	SF	2	3	26, 27	F
**Symptom scales / items**					
Fatigue	FA	3	3	10, 12, 18	
Nausea and vomiting	NV	2	3	14, 15	
Pain	PA	2	3	9, 19	
Dyspnoea	DY	1	3	8	
Insomnia	SL	1	3	11	
Appetite loss	AP	1	3	13	
Constipation	CO	1	3	16	
Diarrhoea	DI	1	3	17	
Financial difficulties	FI	1	3	28	

* *Item range* is the difference between the possible maximum and the minimum response to individual items; most items take values from 1 to 4, giving *range* = 3.

† (revised) scales are those that have been changed since version 1.0, and their short names are indicated in this manual by a suffix "2" – for example, PF2.

For all scales, the *RawScore*, *RS*, is the mean of the component items:

$$RawScore = RS = (I_1 + I_2 + ... + I_n)/n$$

Then for **Functional scales**:

$$Score = \left\{1 - \frac{(RS-1)}{range}\right\} \times 100$$

and for **Symptom scales / items** and **Global health status / QoL:**

$$Score = \{(RS-1)/range\} \times 100$$

Examples:

Emotional functioning

$$RawScore = (Q_{21} + Q_{22} + Q_{23} + Q_{24})/4$$
$$\text{EF } Score = \{1 - (RawScore - 1)/3\} \times 100$$

Fatigue

$$RawScore = (Q_{10} + Q_{12} + Q_{18})/3$$
$$\text{FA } Score = \{(RawScore - 1)/3\} \times 100$$

9.3 EORTC QLQ-OG25 questionnaire

EORTC QLQ – OG25

Patients sometimes report that they have the following symptoms or problems. Please indicate the extent to which you have experienced these symptoms or problems during the past week. Please answer by circling the number that best applies to you.

During the past week:	Not at all	A little	Quite a bit	Very much
31. Have you had problems eating solid foods?	1	2	3	4
32. Have you had problems eating liquidised or soft foods?	1	2	3	4
33. Have you had problems drinking liquids?	1	2	3	4
34. Have you had trouble enjoying your meals?	1	2	3	4
35. Have you felt full up too quickly after beginning to eat?	1	2	3	4
36. Has it taken you a long time to complete your meals?	1	2	3	4
37. Have you had difficulty eating?	1	2	3	4
38. Have you had acid indigestion or heartburn?	1	2	3	4
39. Has acid or bile coming into your mouth been a problem?	1	2	3	4
40. Have you had discomfort when eating?	1	2	3	4
41. Have you had pain when you eat?	1	2	3	4
42. Have you had pain in your stomach area?	1	2	3	4
43. Have you had discomfort in your stomach area?	1	2	3	4
44. Have you been thinking about your illness?	1	2	3	4
45. Have you worried about your health in the future?	1	2	3	4
46. Have you had trouble with eating in front of other people?	1	2	3	4
47. Have you had a dry mouth?	1	2	3	4
48. Have you had problems with your sense of taste?	1	2	3	4
49. Have you felt physically less attractive as a result of your disease or treatment?	1	2	3	4

Please go on to the next page

**During the past week:**	**Not at all**	**A little**	**Quite a bit**	**Very much**
50. Have you had difficulty swallowing your saliva?	1	2	3	4
51. Have you choked when swallowing?	1	2	3	4
52. Have you coughed?	1	2	3	4
53. Have you had difficulty talking?	1	2	3	4
54. Have you worried about your weight being too low?	1	2	3	4
55. Answer this question only if you lost any hair: If so, were you upset by the loss of your hair?	1	2	3	4

9.4 EQ-5D-5L Scoring

2. Scoring the EQ-5D-5L descriptive system

The EQ-5D-5L descriptive system should be scored, for example, as follows:

Under each heading, please tick the ONE box that best describes your health TODAY

**MOBILITY**	
I have no problems in walking about	✔
I have slight problems in walking about	☐
I have moderate problems in walking about	☐
I have severe problems in walking about	☐
I am unable to walk about	☐
**SELF-CARE**	
I have no problems washing or dressing myself	☐
I have slight problems washing or dressing myself	✔
I have moderate problems washing or dressing myself	☐
I have severe problems washing or dressing myself	☐
I am unable to wash or dress myself	☐
**USUAL ACTIVITIES** *(e.g. work, study, housework, family or leisure activities)*	
I have no problems doing my usual activities	☐
I have slight problems doing my usual activities	☐
I have moderate problems doing my usual activities	✔
I have severe problems doing my usual activities	☐
I am unable to do my usual activities	☐
**PAIN / DISCOMFORT**	
I have no pain or discomfort	☐
I have slight pain or discomfort	☐
I have moderate pain or discomfort	☐
I have severe pain or discomfort	✔
I have extreme pain or discomfort	☐
**ANXIETY / DEPRESSION**	
I am not anxious or depressed	☐
I am slightly anxious or depressed	☐
I am moderately anxious or depressed	☐
I am severely anxious or depressed	☐
I am extremely anxious or depressed	✔

Levels of perceived problems are coded as follows:

✔ ☐ ☐ ☐ ☐	Level 1 is coded as a '1'
☐ ✔ ☐ ☐ ☐	Level 2 is coded as a '2'
☐ ☐ ✔ ☐ ☐	Level 3 is coded as a '3'
☐ ☐ ☐ ✔ ☐	Level 4 is coded as a '4'
☐ ☐ ☐ ☐ ✔	Level 5 is coded as a '5'

This example identifies the health state '12345'.

NB: There should be only ONE response for each dimension

*NB: **Missing values** can be coded as '9'.*

*NB: **Ambiguous values** (e.g. 2 boxes are ticked for a single dimension) should be treated as missing values.*

- We would like to know how good or bad your health is TODAY.
- This scale is numbered from 0 to 100.
- 100 means the <u>best</u> health you can imagine.
  0 means the <u>worst</u> health you can imagine.
- Mark an X on the scale to indicate how your health is TODAY.
- Now, please write the number you marked on the scale in the box below.

YOUR HEALTH TODAY = ☐

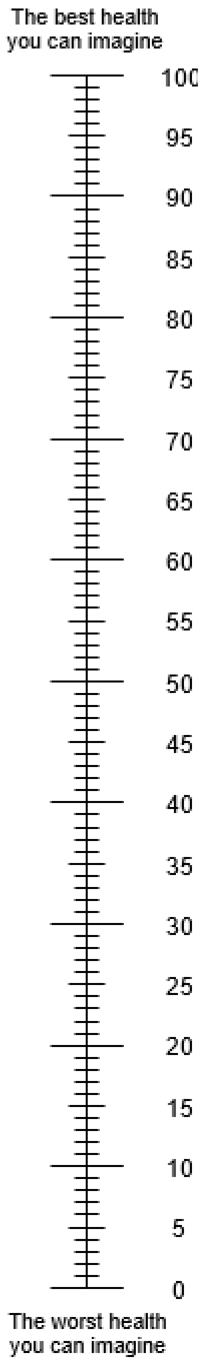

9.5 Global Pain

The Global Pain item is below (the eCOA team can format with radial dials or check boxes)

Please rate your pain by selecting the one number that best describes your pain at its worst in the last 24 hours

0	1	2	3	4	5	6	7	8	9	10
No Pain										Pain as bad as you can imagine

9.6 Health Resource Utilization

1. Since your last study visit, have you had any visits to the emergency room (ER)?

   ☐ No (if no, go to 4)
   ☐ Yes (if yes, go to 2)

2. Since your last visit, how many emergency room visits have you had?
   ____

3. For each emergency room visit please complete the following:

	Result in a Hospital Admission (more than a 24 hour stay)?	Length of stay in hospital (number of days)
ER Visit 1	Yes/No	
ER Visit 2	Yes/No	
ER Visit 3	Yes/No	

4. Since your last study visit, have you had any hospital admissions (more than a 24 hour stay) that occurred <u>without</u> first going to the emergency room (ER)?

   ☐ No (if no, go to 7)
   ☐ Yes (if yes, go to 5)

5. How many hospital admissions (more than 24 hour stay; without previous ER transferal)? ____________

6. For each hospital admission (more than 24 hour stay; without previous ER transferal) visit please complete the following:

	Length of stay in hospital (number of days)
Hospital Visit 1	
Hospital Visit 2	
Hospital Visit 3	

7. Since your last study visit, have you had any visits to a general practitioner (primary care physician)?

☐ No (if no, go to 9)
☐ Yes (if yes, go to 8)

8. How many visits have you had to a general practitioner (primary care physician)? ___________

9. Since your last study visit, did you have any visits to a specialist physician (e.g., oncologist, rheumatologist, endocrinologist, orthopedic surgeon, etc.)?

☐ No
☐ Yes (if yes, go to 10)

How many visits have you had to a specialist physician (e.g., oncologist, rheumatologist, endocrinologist, orthopedic surgeon, etc.)?

9.7 Author and Approver Signatories

(E-signatures are attached at end of document)

PPD

PPD

PPD
