# Supplementary material for: First-line zolbetuximab plus mFOLFOX6 and nivolumab in unresectable CLDN18.2-positive gastric or gastroesophageal junction adenocarcinoma: a phase 2 trial
Source: Nat Med. 2026 Mar 16;32(6):2182–90. doi: 10.1038/s41591-026-04306-9 (PMC13278966; doi:10.1038/s41591-026-04306-9)
Supplement: Supplementary file 2 — Reporting Summary [file 41591_2026_4306_MOESM2_ESM.pdf]

Corresponding author(s): Dr Kohei Shitara
Dr Samuel J. Klempner

Last updated by author(s): Feb 20, 2026

Reporting Summary

Nature Portfolio wishes to improve the reproducibility of the work that we publish. This form provides structure for consistency and transparency in reporting. For further information on Nature Portfolio policies, see our Editorial Policies and the Editorial Policy Checklist.

Statistics

For all statistical analyses, confirm that the following items are present in the figure legend, table legend, main text, or Methods section.

n/a	Confirmed	
☐	☒	The exact sample size (n) for each experimental group/condition, given as a discrete number and unit of measurement
☒	☐	A statement on whether measurements were taken from distinct samples or whether the same sample was measured repeatedly
☐	☒	The statistical test(s) used AND whether they are one- or two-sided<br>*Only common tests should be described solely by name; describe more complex techniques in the Methods section.*
☒	☐	A description of all covariates tested
☒	☐	A description of any assumptions or corrections, such as tests of normality and adjustment for multiple comparisons
☐	☒	A full description of the statistical parameters including central tendency (e.g. means) or other basic estimates (e.g. regression coefficient) AND variation (e.g. standard deviation) or associated estimates of uncertainty (e.g. confidence intervals)
☒	☐	For null hypothesis testing, the test statistic (e.g. F, t, r) with confidence intervals, effect sizes, degrees of freedom and P value noted<br>*Give P values as exact values whenever suitable.*
☒	☐	For Bayesian analysis, information on the choice of priors and Markov chain Monte Carlo settings
☒	☐	For hierarchical and complex designs, identification of the appropriate level for tests and full reporting of outcomes
☒	☐	Estimates of effect sizes (e.g. Cohen's d, Pearson's r), indicating how they were calculated

Our web collection on statistics for biologists contains articles on many of the points above.

Software and code

Policy information about availability of computer code

Data collection	Medidata Rave Electronic Data Capture (EDC) 2025.2.0 was used to collect the study data.
Data analysis	Statistical data analyses were performed with SAS® version 9.4.

For manuscripts utilizing custom algorithms or software that are central to the research but not yet described in published literature, software must be made available to editors and reviewers. We strongly encourage code deposition in a community repository (e.g. GitHub). See the Nature Portfolio guidelines for submitting code & software for further information.

Data

Policy information about availability of data

All manuscripts must include a data availability statement. This statement should provide the following information, where applicable:

- Accession codes, unique identifiers, or web links for publicly available datasets
- A description of any restrictions on data availability
- For clinical datasets or third party data, please ensure that the statement adheres to our policy

The data underlying this manuscript can be requested from the study sponsor, Astellas. Details for how researchers may request access to information from Astellas-sponsored clinical trials can be found at https://www.clinicaltrials.astellas.com/transparency/.

Subject to compliance with the applicable laws and regulations relevant to protection of personal data, Astellas provides a platform (www.vivli.org) where

researchers may request access to participant-level data, trial-level data, and protocols from Astellas-sponsored clinical trials with a medicinal product conducted in patients that are completed after January 1, 2010. Access to this data is granted for medicinal products and indications approved in any country after the request has been reviewed and approved by an independent panel of experts ("Scientific Review Board") based on scientific merit and the qualifications of the researcher. Requestors must be affiliated with an accredited research institution, have relevant education and qualifications, disclose conflicts of interest, submit a detailed research proposal and statistical analysis plan, include a qualified statistician on the team, and sign a Data Use Agreement before access is granted.

Access is given by Astellas after review and approval by the Scientific Review Board and execution of a data sharing agreement. If certain data elements cannot be made available due to privacy, ethical, or regulatory limitations, this will be communicated to the requester. Before participant-level data is shared, it is anonymized to respect the rights of the clinical trial patients to privacy and to protection of their personal health information. Anonymized data will be shared in the Vivli secure research environment. On average, it takes a few months to access data after submitting a request, but the timeline can vary based on the number of data contributors, studies involved, and the requester's responsiveness to comments.

Further details on Astellas' data sharing criteria and process for requesting access can be found on the Astellas Member Page on www.vivli.org.

Research involving human participants, their data, or biological material

Policy information about studies with human participants or human data. See also policy information about sex, gender (identity/presentation), and sexual orientation and race, ethnicity and racism.

Reporting on sex and gender

Sex was collected as a demographic variable and recorded in the electronic case report form. Only biological sex (male or female) was captured; gender identity was not collected in this study. The study was a single-arm trial and was not designed to evaluate treatment effects by sex or gender. Therefore, no sex- or gender-based stratification or subgroup analyses were performed. Overall numbers by sex are provided in Table 1 of the submitted manuscript.

Reporting on race, ethnicity, or other socially relevant groupings

Race was collected as a demographic variable in the electronic case report form (eCRF) using predefined categories: White, Black or African American, Asian, American Indian or Alaska Native, Native Hawaiian or Other Pacific Islander, and Other). Race was summarized descriptively as part of the baseline characteristics in Table 1 of the manuscript. No analyses were performed that adjusted for or compared outcomes by race, and race was not used as a proxy for any other social or socioeconomic variables. As this was a single-arm study with descriptive analyses only, no control for confounding variables based on race was undertaken.

Population characteristics

Covariate-relevant population characteristics are summarized in Table 1 of the manuscript.

Recruitment

Participants were identified mainly at clinical study sites as new patients presenting or through review of patient files by the site staff. Patients were screened and enrolled between February 2, 2022, and September 5, 2022, for cohort 4A and between May 29, 2023, and October 9, 2024, for cohort 4B.
Study sites and their principal investigators included:

Hopital Morvan - CHU Brest, Jean Philippe Metges
Hopital Haut Leveque, Denis Smith, Comite de Protection des Personnes Sud-Est 1 CHU de Saint-Etienne – Hopital de Bellevue
Hopital Europeen Georges Pompidou, Aziz Zaanan
Seconda Universita degli Studi di Napoli, AOU, Ferdinando De Vita
SO S.Chiara, AOU Pisana, Gianluca Masi, Comitato Etico Degli IRCCS Istituto Europeo di Oncologia e Centro Cardiologico Monzino
Istituto Oncologico Veneto, Sara Lonardi
Istituto Europeo di Oncologia, Nicola Fazio
National Cancer Center Hospital East, Kohei Shitara, National Cancer Central Institutional Review Board
National Cancer Center Hospital, Hirokazu Shoji, National Cancer Central Institutional Review Board
The Cancer Institute Hospital of JFCR, Kensei Yamaguchi, Cancer Institute Hospital of JFCR Institutional Review Board
Seoul National University Hospital, Tae Yong Kim, Seoul National University College of Medicine / Seoul National University Hospital Institutional Review Board
Seoul National University Bundang Hospital, Keun Wook Lee, Seoul National University Bundang Hospital institutional review Board
China Medical University Hospital, Li Yuan Bai, Research Ethics Committee, China Medical University & Hospital

Ethics oversight

The protocol and all amendments were approved by the appropriate ethics committee or institutional review board at each participating institution. Patients provided written informed consent before participating in the trial. All the authors attest that the trial was conducted in accordance with the Declaration of Helsinki and the standards of Good Clinical Practice.

Note that full information on the approval of the study protocol must also be provided in the manuscript.

Field-specific reporting

Please select the one below that is the best fit for your research. If you are not sure, read the appropriate sections before making your selection.

☒ Life sciences ☐ Behavioural & social sciences ☐ Ecological, evolutionary & environmental sciences

For a reference copy of the document with all sections, see nature.com/documents/nr-reporting-summary-flat.pdf

Life sciences study design

All studies must disclose on these points even when the disclosure is negative.

Sample size	The sample size of cohort 4A was not based on a statistical power calculation but was expected to provide safety information to determine the tolerability of the dose level of interest. The expected sample size of approximately 65 patients for cohort 4B was not based on strict statistical consideration but was expected to yield 50 patients whose tumors had high CLDN18.2 expression based on population prevalence estimates. For patients with high CLDN18.2 expression, assuming an accrual period of 12 months and a follow-up period of 3–6 months and 20–25 PFS events, the sample size of 50 was expected to provide 70.37%– 76.13% power to detect the difference in PFS with the assumption of a 12-month median PFS for the triplet therapeutic regimen versus an 8.5-month median PFS (zolbetuximab plus nivolumab and mFOLFOX6 versus zolbetuximab and mFOLFOX6) using a 1-sided 15% Type I error. It was assumed that the survival time distributions of both groups were approximated reasonably well by the Weibull distribution with a shape parameter of 1 (exponential distribution).
Data exclusions	For time-to-event endpoints such as PFS and OS, we applied a ”censoring rule” to accommodate the available information on survival so there were no specific missing value issues. Detailed censoring rules, including the handling of intercurrent events, were pre-specified in the Statistical Analysis Plan, which is provided as supplementary material with the manuscript.
Replication	Not performed
Randomization	This was a single-arm study with no allocation to different experimental groups. Therefore, randomization was not applicable. As no group comparisons were planned, covariate control or adjustment was not required.
Blinding	This was an open-label, single-arm study. Therefore, blinding was not applicable.

Reporting for specific materials, systems and methods

We require information from authors about some types of materials, experimental systems and methods used in many studies. Here, indicate whether each material, system or method listed is relevant to your study. If you are not sure if a list item applies to your research, read the appropriate section before selecting a response.

Materials & experimental systems

n/a	Involved in the study	
☒	☐	Antibodies
☒	☐	Eukaryotic cell lines
☒	☐	Palaeontology and archaeology
☒	☐	Animals and other organisms
☐	☒	Clinical data
☒	☐	Dual use research of concern
☒	☐	Plants

Methods

n/a	Involved in the study	
☒	☐	ChIP-seq
☒	☐	Flow cytometry
☒	☐	MRI-based neuroimaging

Clinical data

Policy information about clinical studies

All manuscripts should comply with the ICMJE guidelines for publication of clinical research and a completed CONSORT checklist must be included with all submissions.

Clinical trial registration	The trial was registered at ClinicalTrials.gov (NCT03505320).
Study protocol	The study protocol and statistical analysis plan were provided as supplementary materials with the manuscript.
Data collection	Tumor response was assessed by imaging at screening, and every 8 (± 1) weeks counting from C1D1 for the first 56 weeks, and then every 12 (± 2) weeks thereafter. Survival was assessed every 12 weeks ± 2 weeks for all subjects. Patients completed health-related quality of life assessments, including QLQ-C30, OG-25, GP and the EQ-5D questionnaires on days when the subject receives zolbetuximab treatment, at study treatment discontinuation, and 30 and 90 days following study treatment discontinuation. Adverse events, graded according to the National Cancer Institute Common Terminology Criteria for Adverse Events version 4.03, were evaluated throughout the trial and for 90 days following study treatment discontinuation.
Outcomes	The Statistical Analysis Plan specifies the overall study-level primary objective as the assessment of ORR for single-agent zolbetuximab based on independent central review. However, the present manuscript reports results specifically from Cohort 4. For Cohort 4, all efficacy outcomes including ORR, DCR, DOR, and PFS based on investigator assessment according to RECIST 1.1, as well as OS were prespecified as cohort-level secondary endpoints. In addition, safety and tolerability were evaluated as prespecified secondary endpoints for Cohort 4.
